# Molecular-level insight into the multiple mechanistic pathways in iron-catalysed alkene dimerisation

**DOI:** 10.1039/d5sc07490h

**Published:** 2025-10-29

**Authors:** Joseph H. P. Cockcroft, Annabel Flook, Patrick J. Boaler, Gary S. Nichol, Jarle Holt, Joost Smit, Jennifer A. Garden, Stephen P. Thomas

**Affiliations:** a EaStCHEM School of Chemistry, Joseph Black Building, The University of Edinburgh, David Brewster Road Edinburgh EH9 3FJ UK J.Garden@ed.ac.uk stephen.thomas@ed.ac.uk; b Johnson Matthey, Technology Centre Princeton Drive Stockton-on-Tees TS17 6PY UK

## Abstract

As the least expensive, least toxic and most abundant of the first-row transition metals, iron catalysis underpins the future of sustainable synthesis. Yet the mechanistic understanding remains limited, particularly for pathways involving low oxidation-state intermediates. The reductive dimerisation of alkenes is a prime example, with very few iron-catalysed examples reported and with no in-depth mechanistic analyses. As simple, 1,2-disubstituted alkenes, methyl crotonates can be selectively dimerised to 2-ethylidene-3-methylpentanedioates with two stereogenic units. This rare non-arene example of a C(sp^2^)–H functionalisation offers a platform for molecular-level understanding of broad scope iron-catalysed C–H functionalisation. In-depth mechanistic studies of this dimerisation, including the speciation of [(dmpe)_2_FeH_2_] through a combination of X-ray diffraction, kinetic analysis and *in situ* NMR monitoring, has uncovered hidden pathways that show this “simple” dimerisation is in fact a mechanistically complex system.

## Abstract 2

Mechanistic investigations of the iron-catalysed methyl crotonate dimerisation using *in situ NMR* monitoring and crystallisation of reaction intermediates showed light-mediated and base-mediated pathways operate dependent on the reaction conditions.

## Graphical abstract

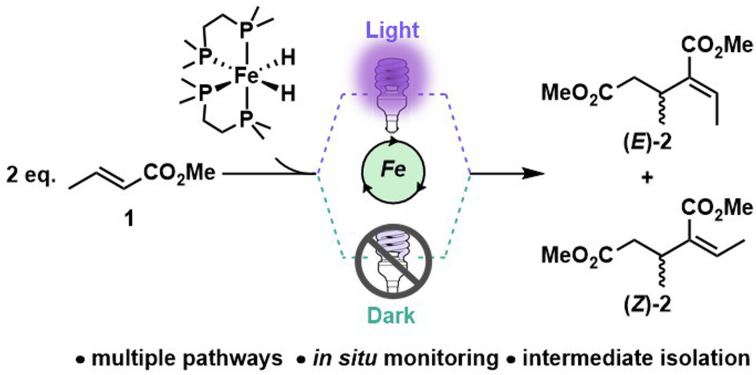

## Introduction

Alkenes are pivotal building blocks across small molecules and macromolecules, with key applications in commodity chemicals alongside polyolefin and polyacrylate materials.^1–4^ With increasing limitations on the use and supply of precious metals,^5^ it is vital that the chemical transformation of alkenes transitions to Earth-abundant metal catalysts. While the Earth-abundant metal-catalysed polymerisations of alkenes have been well-established,^6,7^ the linear and cyclic dimerisation of alkenes remains underexplored.^8–12^ Mechanistic understanding of Earth-abundant metal-catalysed functionalisation reactions is key to designing more efficient catalysts yet has been limited so far by difficulties in observing and isolating low oxidation-state intermediates. This in turn limits the industrial scalability of Earth-abundant metal-catalysed processes, presenting a key scientific challenge.

Methyl crotonate 1 is an alkene monomer that, unlike its methyl methacrylate isomer, does not readily polymerise under radical or anionic conditions.^13,14^ Unlike the dimerisation of similar alkenes,^9,15^ the iron-catalysed dimerisation of methyl crotonate 1 to (*E*),(*Z*)-2-ethylidene-3-methylpentanedioate 2 is limited to a single report from Komiya and co-workers which used [(dmpe)_2_FeH_2_] (dmpe = 1,2-bis(dimethylphosphino)ethane) 3. [(dmpe)_2_FeH_2_] 3, which has been shown to exist in the *cis*-configuration in solution by ^1^H NMR spectroscopy^16^ (see also SI S2.2 for further details) presumably following *cis*–*trans* isomerisation which may or may not be light mediated, has exhibited high reactivity for the metalation and onward reaction of C(sp)–H,^17^ C(sp^2^)–H^18^ and even C(sp^3^)–H^19^ bonds.^20–22^ Although some mechanistic studies have been reported for iron-catalysed C–H functionalisation reactions using [(dmpe)_2_FeH_2_] 3,^21,23,24^ the proposed short-lived iron(0) intermediates are notoriously difficult to identify, characterise and isolate, and this is further complicated when simultaneous irradiation and analysis is required.

**Scheme 1:**
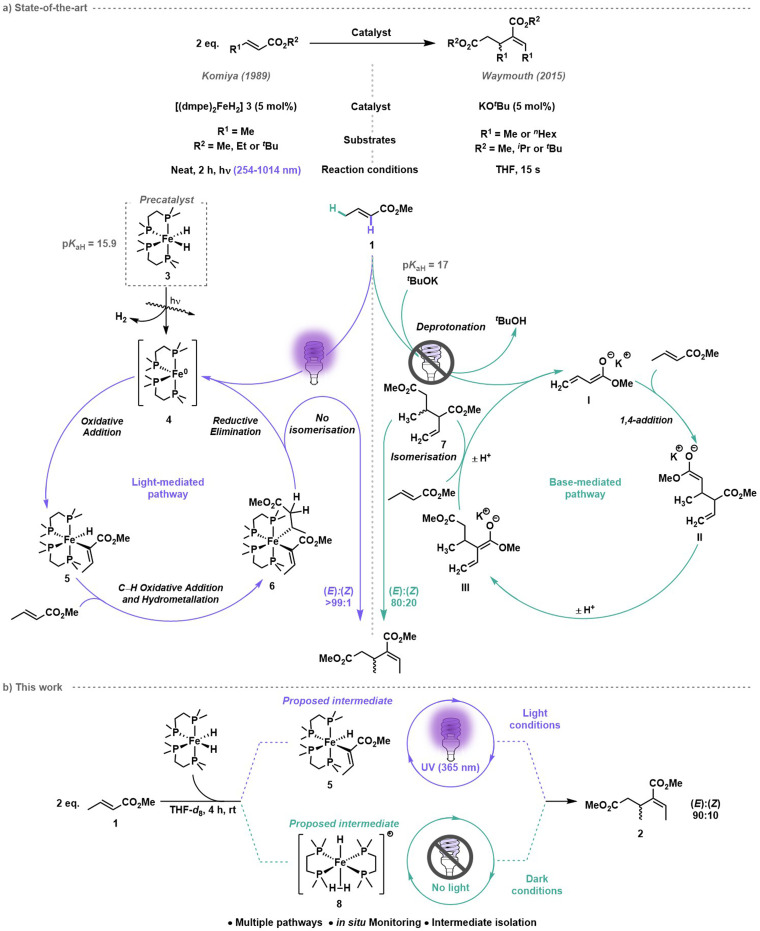
(a) Crotonate dimerisation by iron-catalysed (Komiya and co-workers) and base-catalysed (Waymouth and co-workers) methods. (b) This work: mechanistic investigation of methyl crotonate dimerisation using [(dmpe)_2_FeH_2_] 3 as pre-catalyst under light and dark conditions.

Komiya and co-workers proposed that the first step of the mechanism for iron-catalysed crotonate dimerisation was the photoirradiation of [(dmpe)_2_FeH_2_] 3 to trigger reductive elimination of dihydrogen to form [(dmpe)_2_Fe^0^] 4 (Scheme 1a, left).^26^ Subsequent oxidative addition of the alkenyl α-C(sp^2^)–H bond to the ester formed an iron monohydrido–monoalkenyl species [(dmpe)_2_FeH(CH_3_CH=CCO_2_Me)] 5, in analogy to tetrakis(phosphine) ruthenium complexes undergoing C(sp^2^)–H alkenyl insertion of methacrylates as reported by Yamamoto, Ibers and co-workers.^25^ Insertion of a second methyl crotonate 1 into [(dmpe)_2_FeH(CH_3_CH=CCO_2_Me)] 5 generated the hydrometallation product, iron monoalkyl–monoalkenyl species [(dmpe)_2_Fe(CH_3_CH=CCO_2_Me)(CH_3_CH–CH_2_CO_2_Me)] 6. Reductive elimination reformed [(dmpe)_2_Fe^0^] 4 and a >99 : 1 (*E*) : (*Z*) mixture of 2-ethylidene-3-methylpentanedioate 2.^26^

Waymouth and co-workers reported the base-catalysed reductive dimerisation of alkyl crotonates without any photoirradiation using potassium *tert*-butoxide to achieve high yields and 80 : 20 (*E*) : (*Z*) stereoselectivity.^27^ Using methyl crotonate as an example, Waymouth proposed δ-C–H deprotonation (C(sp^3^)–H bond) gave the (extended) enolate I (Scheme 1a, right). 1,4-Addition of I to another molecule of methyl crotonate 1 gave an anionic dimer II, which underwent proton transfer to give the conjugated enolate III, which deprotonated a third methyl crotonate 1 to regenerate enolate I as well as forming dimethyl 2-ethenyl-3-methylpentanedioate 7. The terminal alkene of dimethyl 2-ethenyl-3-methylpentanedioate 7 then underwent isomerisation to the conjugated alkene, to give (*E*),(*Z*)-2-ethylidene-3-methylpentanedioate 2. Importantly, the mechanisms proposed by Komiya^26^ and Waymouth^27^ involve different intermediate species, yet the p*K*_aH_ of [(dmpe)_2_FeH_2_] 3 (15.9)^28,29^ and p*K*_aH_ of potassium *tert*-butoxide (17)^30^ are similar. This raises an important question: does [(dmpe)_2_FeH_2_] 3 act solely as a precursor that requires photoirradiation to form the active catalyst [(dmpe)_2_Fe^0^] 4, or is [(dmpe)_2_FeH_2_] 3 itself catalytically active through a base-mediated pathway? Herein, we report a molecular-level investigation of methyl crotonate dimerisation with [(dmpe)_2_FeH_2_] 3 as the catalyst. A combination of kinetic studies and in-depth NMR reaction monitoring under light and dark conditions, with a focus on tracking the different iron species formed *in situ*, have revealed the hidden complexity of this seemingly simple crotyl dimerisation.^31,32^

## Results and discussion

Investigations of the dimerisation of 98% *trans*-diastereomer methyl crotonate 1 began by testing different wavelengths of photoirradiation and catalytic activity (Table 1). Use of wavelengths between 254 and 1014 nm (5 mol% [(dmpe)_2_FeH_2_] 3, neat conditions, 4 hours) showed no significant difference in either yield (75–89%) or stereoselectivity (≥85 : 15 (*E*) : (*Z*)) of 2-ethylidene-3-methylpentanedioate 2 across all wavelengths (Table 1, entries 1–4). The lower selectivity obtained compared to Komiya's conditions (Scheme 1a, left) could be attributed to a base-mediated pathway (Scheme 1a, right). Dark conditions (entry 5) gave a very similar yield and selectivity, generating 75% yield of (*E*),(*Z*)-2-ethylidene-3-methylpentanedioate 2 with 87 : 13 (*E*) : (*Z*) stereoselectivity under otherwise identical conditions. This indicated that in the absence of photoirradiation [(dmpe)_2_FeH_2_] 3 would act as a base, initiating the base-mediated pathway. However, the reaction was significantly slower than with Waymouth's KO^*t*^Bu system (4 h *vs.* 15 seconds), hinting that the reaction may not solely follow a base-mediated pathway under dark conditions. Subsequent variable time normalised analysis (VTNA) studies showed that the reaction was half-order in [(dmpe)_2_FeH_2_] 3 under light and dark conditions,^32^ indicative of potentially a dimeric iron species. Similar first-row transition metal-catalysed functionalisation reactions have noted that non-integer catalyst reaction orders indicate an off-cycle dimeric species which dissociates to two on-cycle (monomeric) species, or that the active catalyst is dimeric.^33–35^ The potential for both light-mediated and base-mediated pathways, and the associated multitude of equilibria and reaction pathways, would account for the observation of [(dmpe)_2_FeH_2_] 3 being half-order in the reaction.^33^ As the light-mediated and base-mediated pathways involve the reaction of different C–H bonds of methyl crotonate (Scheme 1a), two deuterated analogues of methyl crotonate 1 were synthesised to probe the rate-limiting step *via* analysis of kinetic isotope effects (KIEs). For the light-mediated cycle, alkene C–H deuterium isotopologue *d*_1_-1 featured an alkenyl (CO_2_Me)C(sp^2^)–D bond, to investigate whether the oxidative addition of crotonate 1 to form [(dmpe)_2_FeH(CH_3_CH=CCO_2_Me)] 5 was rate-limiting. The second deuterated substrate, tailored for the base-mediated pathway, featured an alkenyl CD_3_ unit *d*_3_-1 to probe the formation of the crotyl-enolate I and [(dmpe)_2_Fe(H)H_2_]^+^8 (see SI S8.1 for further details). In both instances, KIEs of 1 were observed, indicating that light-mediated oxidative addition and base-mediated CH_3_ deprotonation of 1 were not the rate-limiting steps in either reaction.

**Table 1:** Initial trials to investigate the wavelength range under which [(dmpe)_2_FeH_2_] 3 catalyses methyl crotonate dimerisation

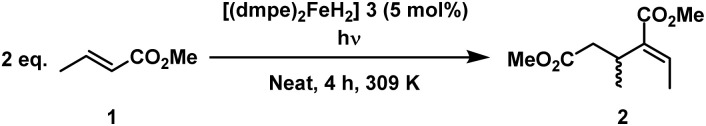				
Entry	Light source	Wavelength range (nm)	Yield of 2^a^ (%)	(*E*) : (*Z*)
1	Hg light	254–1014	87	88 : 12
2	UV-B	280–315	85	86 : 14
3	UV-A	315–400	78	85 : 15
4	Blue light	400–500	89	86 : 14
5	No light	n/a	75	87 : 13

a All conversions were measured by ^1^H NMR spectroscopy *versus* a 1 M trimethoxybenzene standard solution in diethyl ether. Conversion refers to the amount of 2-ethylidene-3-methylpentanedioate 2 formed from methyl crotonate 1. Reactions were left for 4 hours directly in front of a light source; for testing dark conditions, a box was placed over the sample.

**Fig. 1:**
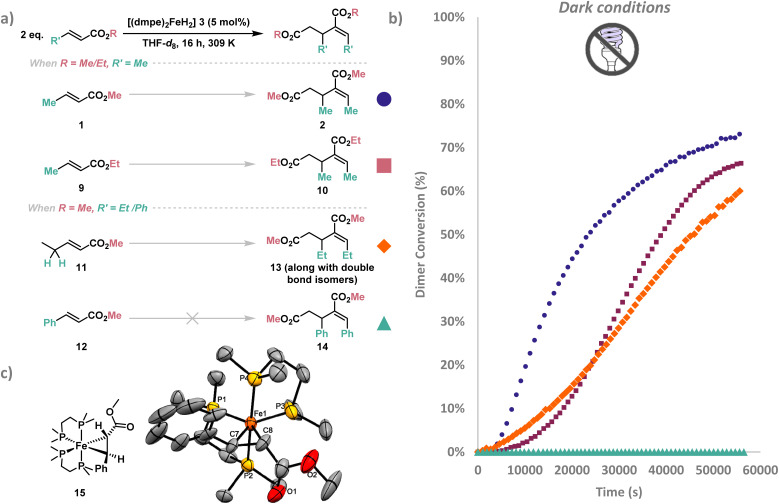
(a) i*n situ* Monitoring of crotyl substrates 1, 9, 11 and 12 under dark conditions with [(dmpe)_2_FeH_2_] 3 (5 mol%) in THF-*d*_8_ at 309 K were converted to dimers 2, 10, 13 and 14 respectively. The substrates selected either had a different alkyl ester (
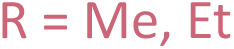
) or different substituents at the vinylogous position (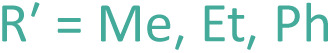). Product 13 was formed along with double bond isomers as the major products (see SI S9 SI Fig. 45–47 for additional details). (b) The dimer conversion (%) over time (s) across 16 hours under dark conditions. Dimer conversion (%) was calculated by integrating ^1^H resonances corresponding to starting material and dimer products, then computing (dimers/(dimers + starting material)) × 100%. (c) ORTEP plot of [Fe(PhCH=CHCO_2_Me)(dmpe)_2_] 15, hydrogens have been omitted for clarity and X-ray ellipsoids have been set at 50% probability.

Given the prominence of the base-mediated pathway, the dimerisation of other crotyl substrates was investigated under dark conditions (Fig. 1). To investigate the influence of changing the ester substituent from a methyl to an ethyl group, the dimerisation of ethyl crotonate 9 was investigated under dark conditions for 16 hours at 309 K (see SI S9 SI Fig. 42–44 for more details). Under the same reaction conditions used for methyl crotonate 1, ethyl crotonate 9 gave a lower conversion of 66% to diethyl 2-ethylidene-3-methylglutarate 10 with 95 : 5 (*E*) : (*Z*) selectivity in 16 hours (*vs.* 75% conversion of methyl crotonate 1 to 2-ethylidene-3-methylpentanedioate 2 with 87 : 13 (*E*) : (*Z*) stereoselectivity (Table 1, entry 5)). As the p*K*_a_ of the methyl C–H bond of 1 and 9 are near-identical,^36^ this indicates that the larger ester group may destabilise or inhibit the formation of an intermediate, leading to a slower rate of dimer formation.

Two chain-extended analogues of methyl crotonate were also investigated (see SI S9 SI Fig. 45–49 for more details); methyl (*E*)-2-pentenoate 11 and methyl cinnamate 12 (Fig. 1a). These substrates were selected because the CH_3_ site deprotonated in the base-mediated pathway to form crotyl enolate I (Scheme 1a, right) was replaced with a CH_2_Me group (*i.e.* a more sterically encumbered C(sp^2^)–H bond with fewer available protons), and a phenyl group (no available protons). The dimerisation of methyl (*E*)-2-pentenoate 11 showed a similar rate to that of its structural isomer, ethyl crotonate 9, however the major dimer species observed were double bond isomers of (*E*)- and (*Z*)-2-(1-propenyl)-3-ethylglutaric acid dimethyl ester 13 (see SI S9, SI Fig. 46 for further details) similar to findings reported by Waymouth.^27^ As all products formed from methyl (*E*)-2-pentenoate 11 dimerisation contain an internal alkene, there is no thermodynamic favourability to isomerise to the expected product (*E*),(*Z*)-2-(1-propenyl)-3-ethylglutaric acid dimethyl ester 13. This contrasts from the isomerisation pathway observed with methyl crotonate 1 which isomerises to the more thermodynamically stable internal conjugated alkene (Scheme 1a, right). Methyl cinnamate 12, which has no allylic C–H bonds, showed no observable dimerisation after 16 hours in the absence of light irradiation, providing further support for the base-mediated pathway where suitably acidic C–H bonds are present.

Although no dimerisation of methyl cinnamate 12 was recorded under dark conditions, exposure of the same sample to UV irradiation (365 nm) gave rapid conversion to the dimer (*E*),(*Z*)-2-benzylidene-3-phenyl-glutaric acid dimethyl ester 14. This indicated that only the redox Fe^0^/Fe^II^ reductive dimerisation pathway was operating for methyl cinnamate 12 (Scheme 1a, left). Accordingly, ^31^P{^1^H} NMR spectroscopy showed that the metallacyclopropane iron species [(dmpe)_2_Fe(PhCH=CHCO_2_Me)] 15 formed steadily to become the major species after 2 hours. Red crystals deposited from pentane at −35 °C were analysed by single crystal X-ray crystallography and identified as complex 15 (Fig. 1c, see SI S7.1 for more details). [(dmpe)_2_Fe(PhCH=CHCO_2_Me)] 15 was confirmed to be a catalytically active intermediate when used as a catalyst (5 mol%) with methyl crotonate 1 and exposed to UV (365 nm) irradiation for 1 hour, giving the continued formation of (*E*),(*Z*)-2-ethylidene-3-methylpentanedioate 2, in addition to small amounts of other dimers (see SI S9, SI Fig. 77).

**Fig. 2:**
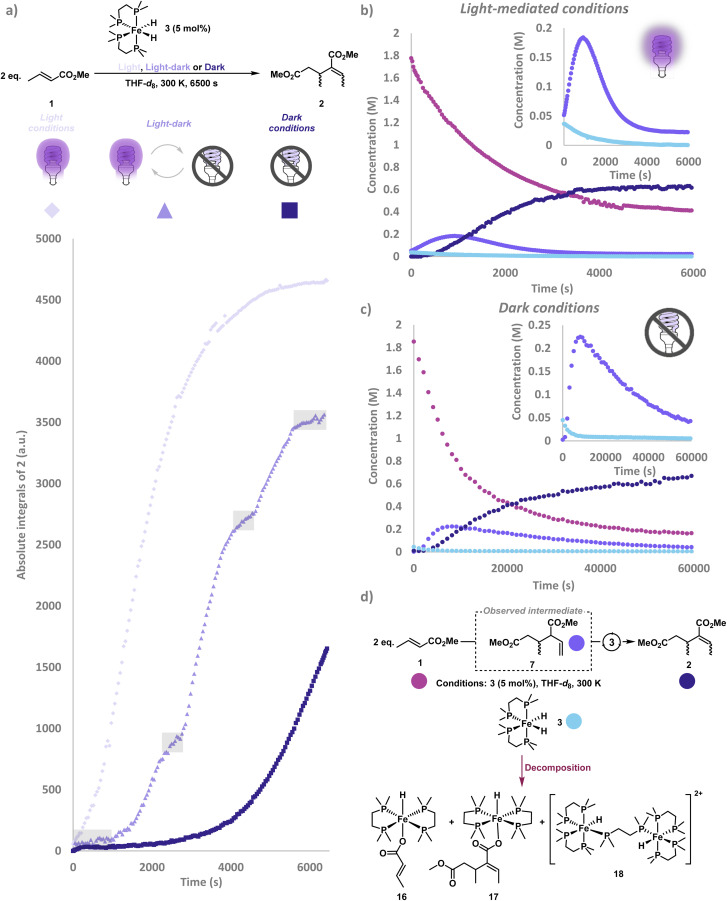
(a) Methyl crotonate 1 dimerisation under light (lilac diamond), light–dark (purple triangle) and dark (blue square) conditions. Boxes in light grey are for the light–dark experiment only, representing time points taken where the light source was turned off. Absolute integrals acquired from ^1^H NMR spectroscopy with post-acquisition signal-averaging.^38^ (b) Light-mediated methyl crotonate 1 (pink) dimerisation to (*E*),(*Z*)-2-ethylidene-3-methylpentanedioate 2 (blue) by [(dmpe)_2_FeH_2_] 3 pre-catalyst (light blue) showing the formation of intermediate dimethyl 2-ethenyl-3-methylpentanedioate 7 (purple) is iron-catalysed. Absolute integrals acquired from ^1^H NMR spectroscopy with post-acquisition signal-averaging.^38^ (c) Methyl crotonate 1 (pink) dimerisation to (*E*),(*Z*)-2-ethylidene-3-methylpentanedioate 2 (blue) under dark conditions which shows that the intermediate dimethyl 2-ethenyl-3-methylpentanedioate 7 (purple) is not light-dependent. Absolute integrals acquired from ^1^H NMR spectroscopy. (d) Decomposition of [(dmpe)_2_FeH_2_] 3 to *trans*-[(dmpe)_2_FeH(CH_3_CH=CH–COO^−^)] 16, *trans*-[(dmpe)_2_FeH(CH_3_O_2_CC_6_H_10_COO^−^)] 17 and [{(dmpe)_2_FeH}_2_(μ-dmpe)]^2+^18.

Using methyl crotonate 1 as a benchmark to gain insight into the iron species formed in the light and dark pathways, dimerisation by [(dmpe)_2_FeH_2_] 3 was monitored using *in situ* LED ^1^H and ^31^P{^1^H} NMR spectroscopy^37^ over a 2 hours period (Fig. 2a) with post-acquisition FID processing.^38^ A light–dark experiment (Fig. 2a) showed that upon irradiation a large increase in the rate of product formation occurred, whilst stopping irradiation (dark) showed a slower rate of conversion. This suggests that the two mechanisms operate in tandem in the presence of light, with the base-mediated pathway as the major mechanism in the absence of light. Even after complete consumption of [(dmpe)_2_FeH_2_] 3 in the light–dark reaction, a rapid increase in the absolute integrals of product (*E*),(*Z*)-2-ethylidene-3-methylpentanedioate 2 was observed upon re-exposure to UV (365 nm) irradiation. This suggested that light was not only required for pre-catalyst activation (*via* reductive elimination of dihydrogen from [(dmpe)_2_FeH_2_] 3 to form [(dmpe)_2_Fe^0^] 4), but also a step on the catalytic cycle: hydrometallation of a second equivalent of methyl crotonate 1 and/or reductive elimination of (*E*),(*Z*)-2-ethylidene-3-methylpentanedioate 2. ^31^P{^1^H} NMR spectroscopic analysis revealed an intermediate that increased in absolute concentration under light conditions but remained constant under dark conditions. Four distinct ^31^P{^1^H} NMR resonances suggested that this *cis*-configured intermediate was either the oxidative addition product [(dmpe)_2_FeH(CH_3_CH=CCO_2_Me)] 5 (Scheme 1a) or a metallacyclopropane intermediate similar to [(dmpe)_2_Fe(PhCH=CHCO_2_Me)] 15 (Fig. 1c).

An intermediate was visible by ^1^H NMR spectroscopy under both light and dark conditions, which had diagnostic geminal alkene signals consistent with a diastereomeric mixture of dimethyl 2-ethenyl-3-methylpentanedioate 7 (Scheme 1a, right and Fig. 2a). As dimethyl 2-ethenyl-3-methylpentanedioate 7 is not on the oxidative pathway, its presence under light conditions confirms that the base-mediated pathway operates under both light and dark conditions. This also aligns with the lower diastereoselectivity observed here compared to that of Komiya and co-workers; the base-mediated pathway lowering the (*E*) : (*Z*) ratio. Under both light and dark conditions, no induction period for the formation of dimethyl 2-ethenyl-3-methylpentanedioate 7 was observed (see SI S4.5 SI Fig. 4 and 5 for further details). Dimethyl 2-ethenyl-3-methylpentanedioate 7 peaked in absolute concentration at roughly 1000 and 10000 seconds under light and dark conditions respectively. Rapid conversion of dimethyl 2-ethenyl-3-methylpentanedioate 7 to (*E*),(Z)-2-ethylidene-3-methylpentanedioate 2 was observed throughout the experiment until dimethyl 2-ethenyl-3-methylpentanedioate 7 was a minor species by ^1^H NMR spectroscopy (Fig. 2b and c).

It is highly likely that the isomerisation of dimethyl 2-ethenyl-3-methylpentanedioate 7 into (*E*),(Z)-2-ethylidene-3-methylpentanedioate 2 was iron-catalysed: no isomerisation occurred when [(dmpe)_2_FeH_2_] 3 was consumed and only decomposition products *trans*-[(dmpe)_2_FeH(CH_3_CH=CH–COO^−^)] 16, *trans*-[(dmpe)_2_FeH(CH_3_O_2_CC_6_H_10_COO^−^)] 17 and [{(dmpe)_2_FeH}_2_(μ-dmpe)]^2+^18 were identified by ^1^H–^31^P HMBC spectroscopy (Fig. 2d, see SI S5.2–5.4 and S7.2–7.3 for further details). [{(dmpe)_2_FeH}_2_(μ-dmpe)]^2+^18 has previously been observed as a decomposition product of [(dmpe)_2_FeH_2_] 3 upon irradiation.^21,39^*trans*-[(dmpe)_2_FeH(CH_3_O_2_CC_6_H_10_COO^−^)] 17 was not catalytically active in the dimerisation of methyl crotonate, presumably as this species is unable to re-enter the catalytic cycle (see SI S9 SI Fig. 85 for more details).^40^ Under dark conditions a small amount of [(dmpe)_2_FeH_2_] 3 was present at the end of the reaction which correlates with the gradual decrease in the concentration of intermediate dimethyl 2-ethenyl-3-methylpentanedioate 7 observed.

**Scheme 2:**
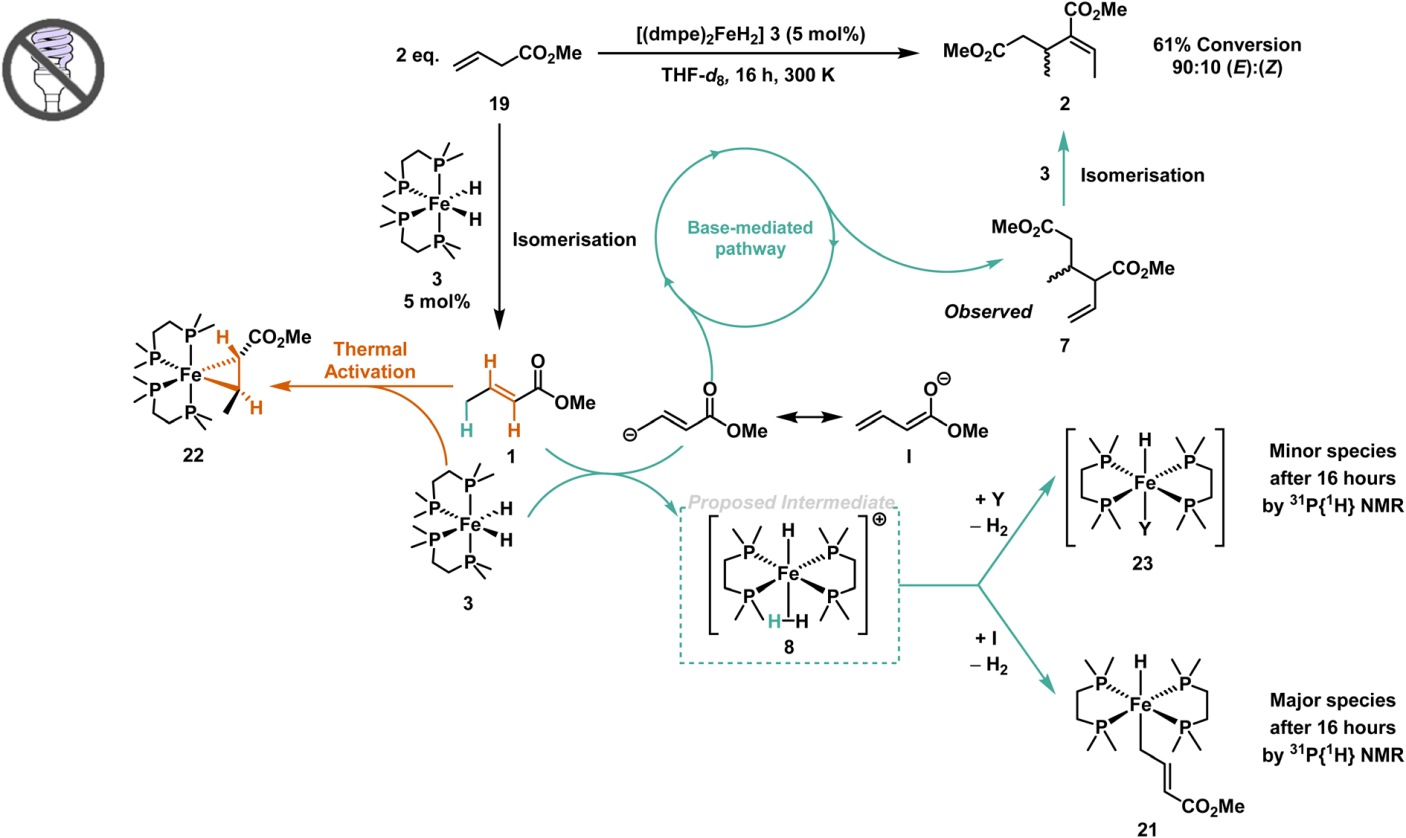
Isomerisation of methyl 3-butenoate 19 to methyl crotonate 1 by [(dmpe)_2_FeH_2_] 3. Methyl crotonate 1 can then be dimerised by the base-mediated pathway to give the dimer intermediate dimethyl 2-ethenyl-3-methylpentanedioate 7 which itself can be isomerised by [(dmpe)_2_FeH_2_] 3 to give (*E*),(*Z*)-2-ethylidene-3-methylpentanedioate 2. ^31^P NMR spectroscopic analysis of the reaction shows that the major iron species is *trans*-[(dmpe)_2_FeH(CH_2_CH=CHCO_2_Me)] 21, with minor amounts of [(dmpe)_2_FeHY] 23. Minor amounts of [(dmpe)_2_Fe(η^2^-1)] 22 by thermal activation of [(dmpe)_2_FeH_2_] 3 were also observed.

To further probe whether the isomerisation of dimethyl 2-ethenyl-3-methylpentanedioate 7 to (*E*),(*Z*)-2-ethylidene-3-methylpentanedioate 2 was catalysed by [(dmpe)_2_FeH_2_] 3, the terminal alkene isomer of 1, methyl 3-butenoate 19, was investigated. Under light-mediated conditions, 19 was completely isomerised to methyl crotonate 1 by [(dmpe)_2_FeH_2_] 3 (5 mol% loading), with 50% conversion of methyl crotonate 1 to 2-ethylidene-3-methylpentanedioate 2 (90 : 10 (*E*) : (*Z*)) after 2 hours (Scheme 2, see SI S9 SI Fig. 67 for further details). Iron-alkenyl complexes identified by ^1^H and ^31^P{^1^H} NMR spectroscopy (see SI S9 SI Fig. 69 for further details) showed conversion of [(dmpe)_2_FeH_2_] 3 to the demethylation complex *trans*-[(dmpe)_2_FeH(CH_3_CH=CH–COO^−^)] 16 in addition to the proposed complex *trans*-[(dmpe)_2_FeH(CH=CHCH_2_CO_2_Me)] 20 which showed a pentet hydride resonance and a single ^31^P NMR resonance similar to previously reported iron monohydrido monoalkyl complexes.^21^

Under dark conditions, reacting methyl 3-butenoate 19 with [(dmpe)_2_FeH_2_] 3 (5 mol% catalyst loading) gave complete isomerisation of methyl 3-butenoate 19 to methyl crotonate 1 after 4 hours (Scheme 2 and see SI S9 SI Fig. 70–72), whilst control reactions without [(dmpe)_2_FeH_2_] 3 showed trace isomerisation (<2%) of methyl 3-butenoate 19 after 16 hours (see SI S9 SI Fig. 73). Dimerisation of methyl crotonate 1 to (*E*),(*Z*)-2-ethylidene-3-methylpentanedioate 2 under dark conditions by [(dmpe)_2_FeH_2_] 3 was also observed (61% conversion, 90 : 10 (*E*) : (*Z*), 16 hours). Baseline peaks congruent with intermediate dimethyl 2-ethenyl-3-methylpentanedioate 7 were observed by ^1^H NMR spectroscopy, suggesting that [(dmpe)_2_FeH_2_] 3 facilitated isomerisation of 7 to (*E*),(*Z*)-2-ethylidene-3-methylpentanedioate 2. These observations show that [(dmpe)_2_FeH_2_] 3 can promote the isomerisation of a terminal alkene to an internal alkene; both methyl 3-butenoate 19 to methyl crotonate 1 and dimethyl 2-ethenyl-3-methylpentanedioate 7 to (*E*),(*Z*)-2-ethylidene-3-methylpentanedioate 2.

^31^P NMR spectroscopic analysis of methyl 3-butenoate 19 with [(dmpe)_2_FeH_2_] 3 under dark conditions showed speciation of [(dmpe)_2_FeH_2_] 3 into one major species after 16 hours, identified as *trans*-[(dmpe)_2_FeH(CH_2_CH=CHCO_2_Me)] 21 (see SI S9 SI Fig. 87–89 for further details). ^1^H–^31^P HMBC spectroscopy showed that the Fe–H resonance (*δ* −25.9 ppm) correlated with a singlet ^31^P NMR resonance, indicative of a *trans*-geometry. The ^1^H NMR spectrum showed diagnostic resonances at *δ* 0.5 ppm and *δ* 7.0 ppm, which coupled to each other with a relative ratio of 2 : 1. Based on these distinctive chemical shifts, the relative integrals and the coupling patterns, these resonances were assigned to the Fe–C*H*_2_C*H*=CHCO_2_Me unit of *trans*-[(dmpe)_2_FeH(CH_2_CH=CHCO_2_Me)] 21. Data congruent with the proposed base-mediated intermediate, *trans*-[(dmpe)_2_FeH(H_2_)]^+^8, was not observed at any point nor was hydrogen gas observed, which is attributed to the low concentration of catalyst.

When *trans*-[(dmpe)_2_FeH(CH_2_CH=CHCO_2_Me)] 21 (see SI S5.5 for more details) was used as a catalyst for methyl crotonate dimerisation, approximately 21% conversion to (*E*),(*Z*)-2-ethylidene-3-methylpentanedioate 2 was observed under dark conditions after 16 hours (see SI S9 SI Fig. 90 for more details). ^31^P{^1^H} NMR data across the same time period showed that *trans*-[(dmpe)_2_FeH(CH_2_CH=CHCO_2_Me)] 21 was gradually converted to *trans*-[(dmpe)_2_FeH(CH_3_CH=CH–COO^−^)] 16. Subsequent exposure of the same sample to irradiation (365 nm) for 20 minutes led to rapid conversion of methyl crotonate 1 to (*E*),(*Z*)-2-ethylidene-3-methylpentanedioate 2, suggesting that *trans*-[(dmpe)_2_FeH(CH_2_CH=CHCO_2_Me)] 21 can undergo light-mediated reductive elimination of methyl crotonate to give [(dmpe)_2_Fe^0^] 4.

Under dark conditions, two additional minor iron species were also identified by ^1^H–^31^P HMBC and ^1^H–^13^C HMBC spectroscopy as [(dmpe)_2_Fe(η^2^-1)] 22 (see SI S5.6 for further details) and [(dmpe)_2_FeHY] 23 (see SI S5.7 for further details). [(dmpe)_2_Fe(η^2^-1)] 22 was present in minor amounts, presumably formed by thermal activation of [(dmpe)_2_FeH_2_] 3.^41^ Four chemically inequivalent ^31^P NMR resonances were observed which did not correspond to any hydride resonance, and were tentatively assigned as the metallacyclopropane complex [(dmpe)_2_Fe(η^2^-1)] 22. *trans*-[(dmpe)_2_FeHY] 23 was present as a minor iron species which increased in absolute intensity across 16 hours. Full assignment of *trans*-[(dmpe)_2_FeHY] 23 was not possible: it was initially assumed that Y was enolate I, however no matching peaks in the ^1^H NMR spectra were found to support this.^42–44^ On the basis of the kinetic data, it was proposed that *trans*-[(dmpe)_2_FeHY] 23 is either a short-lived intermediate or a coordination complex which does not play a major role in methyl crotonate dimerisation.

**Fig. 3:**
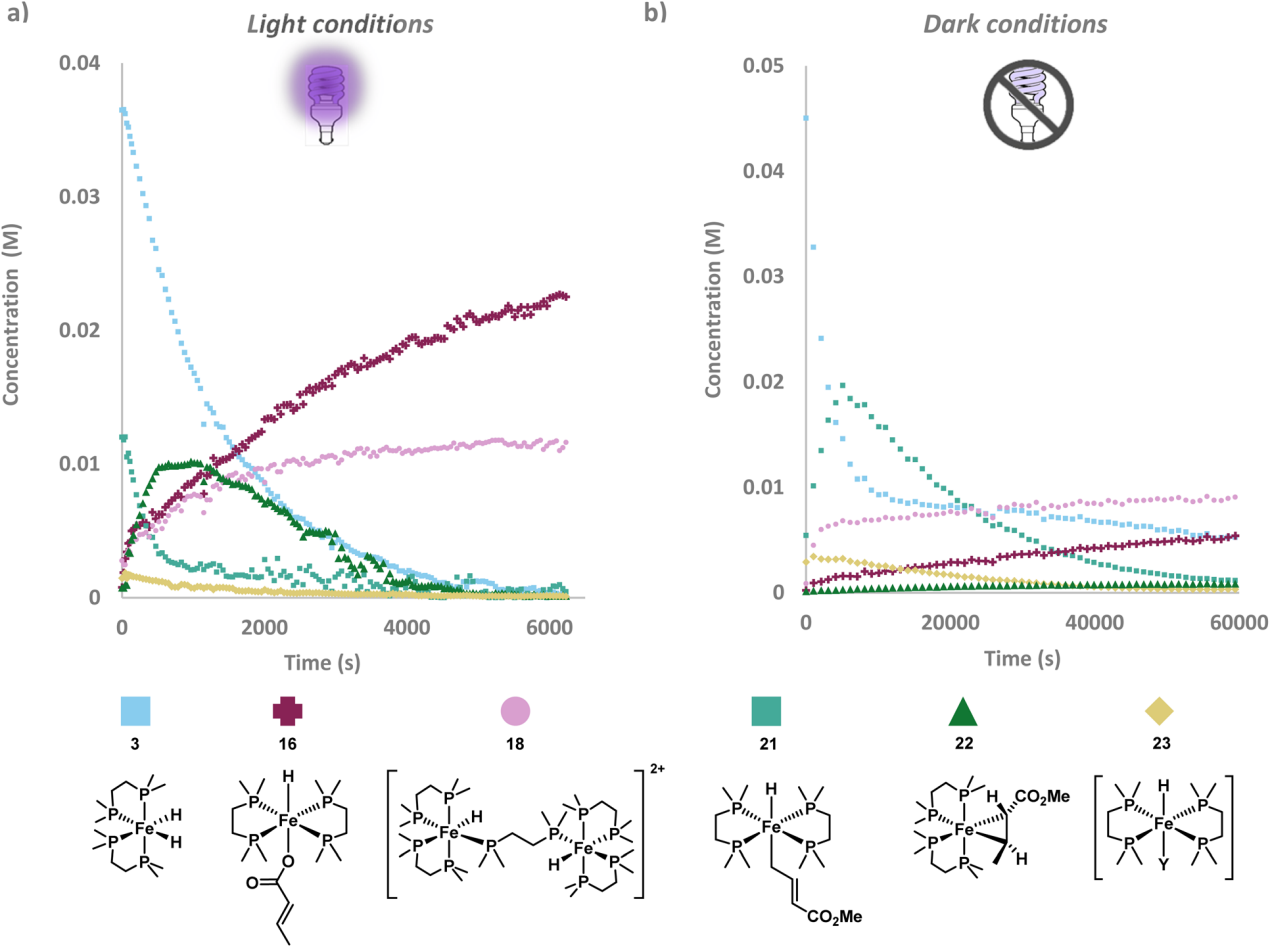
(a) *In situ* monitoring by ^31^P{^1^H} NMR spectroscopy with post-acquisition FID processing showing different iron species formed under light conditions of methyl crotonate 1 dimerisation.^38^ (b) *In situ* monitoring by ^31^P{^1^H} NMR spectroscopy showing different iron species formed under dark conditions.

**Fig. 4:**
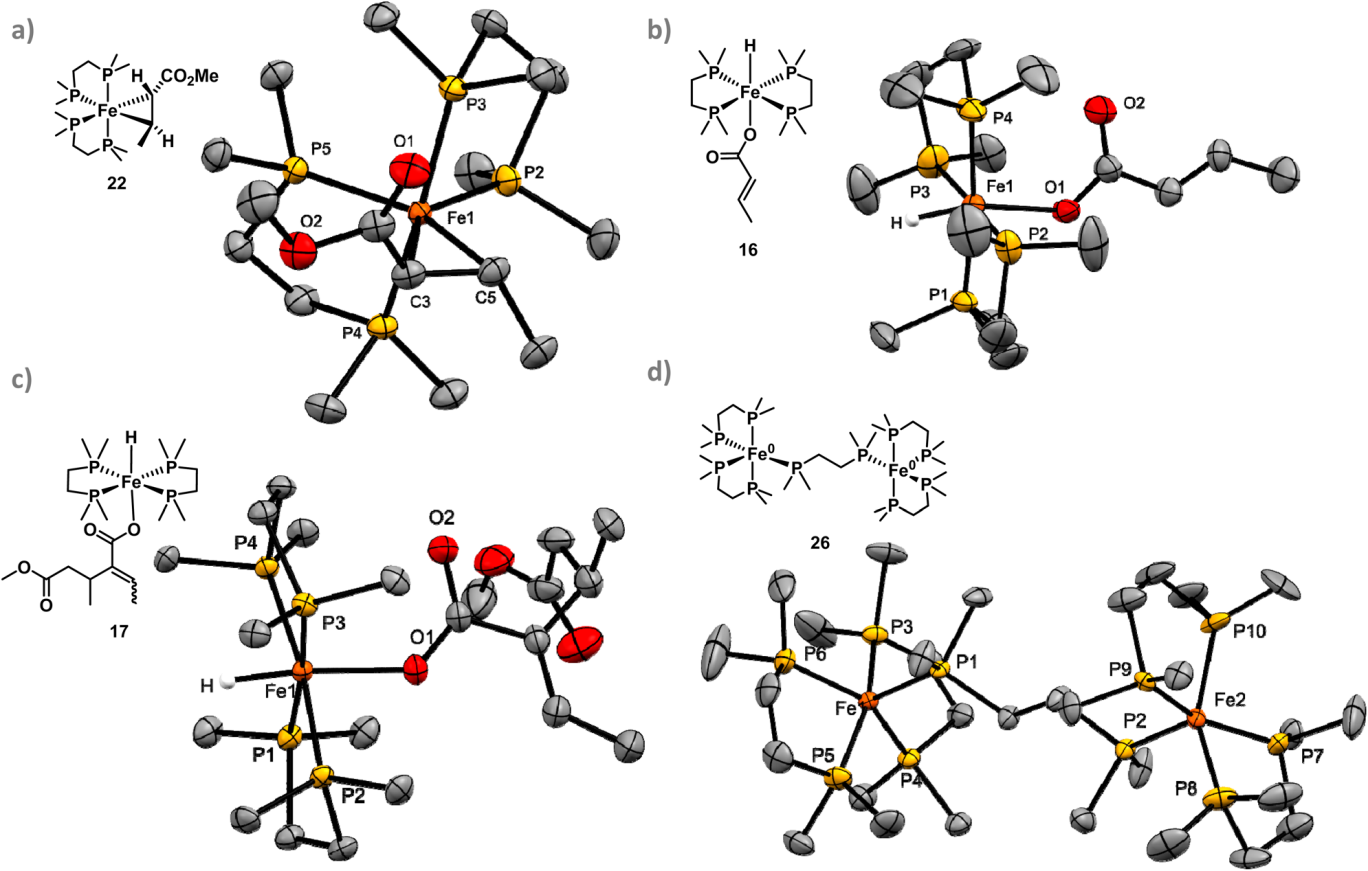
ORTEP plots of (a) [(dmpe)_2_Fe(η^2^-1)] 22. (b) *Trans*-[(dmpe)_2_FeH(CH_3_CH=CH–COO^−^)] 16. (c) *Trans*-[(dmpe)_2_FeH(CH_3_O_2_CC_6_H_10_COO^−^)] 17. (d) [(dmpe)_5_Fe_2_] 26. Iron, carbon, phosphorus, oxygen and hydride atoms on the molecular structures are coloured orange, grey, yellow, red and white respectively. Hydrogens have been omitted for clarity and thermal ellipsoids have been set at 50% probability.

^31^P{^1^H} NMR spectra obtained during *in situ* monitoring of methyl crotonate 1 under light and dark dimerisation conditions both showed several iron species (Fig. 3). Under irradiation, a majority of the pre-catalyst [(dmpe)_2_FeH_2_] 3 was rapidly consumed presumably to give [(dmpe)_2_Fe^0^] 4, which was not observed in accordance with previous reports.^45^ Under irradiation, a majority of [(dmpe)_2_FeH_2_] 3 was converted to [(dmpe)_2_Fe(η^2^-1)] 22 after 20 minutes by ^31^P{^1^H} NMR spectroscopy. Extensive isolation studies of [(dmpe)_2_Fe(η^2^-1)] 22 gave single yellow crystals suitable for X-ray diffraction obtained from a saturated solution of pentane at −35 °C (see SI S7.4 for further details). Analysis of [(dmpe)_2_Fe(η^2^-1)] 22 confirmed an η^2^-alkene coordination and a C3–C5 bond length of 1.448(2) Å (Fig. 4a); this is significantly longer than similar uncoordinated activated alkenes with bond lengths of 1.32–1.37 Å.^46,47^ The Dewar–Chatt–Duncanson model suggests that the bond elongation is more typical of C(sp^3^) character, however the bond angles varied between 110 and 120° suggesting significant disruption of the double bond and that both alkenyl carbons lie between C(sp^2^) and C(sp^3^) hybridisation. Comparing the metallocyclopropane Fe–C or C–C bonds in [(dmpe)_2_Fe(η^2^-1)] 22 and [(dmpe)_2_Fe(PhCH=CHCO_2_Me)] 15 shows no significant difference in bond lengths or bond angles, indicating the metallocyclopropane carbons are between C(sp^2^) and C(sp^3^) hybridisation in both cases. This suggests that the same type of metallacyclopropane intermediate forms from [(dmpe)_2_FeH_2_] 3 and methyl crotonate 1 or methyl cinnamate 12. [(dmpe)_2_Fe(η^2^-1)] 22 was assumed to be the major metallacyclopropane product as no [(dmpe)_2_FeH(CH_3_CH=CCO_2_Me)] 5 or hydrometallation product [(dmpe)_2_Fe(CH_3_CH=CCO_2_Me)(CH_3_CH_2_–CHCO_2_Me)] 6 (Scheme 1) was observed by ^31^P{^1^H} NMR. Under dark conditions, minor peaks of [(dmpe)_2_Fe(η^2^-1)] 22 were observed to increase throughout monitoring, suggesting thermal activation of methyl crotonate 1 by [(dmpe)_2_FeH_2_] 3.^41^ It is possible that a catalytically active species was formed by deprotonation of the allylic methyl group of [(dmpe)_2_Fe(η^2^-1)] 22 by [(dmpe)_2_FeH_2_] 3, as iron coordination can dramatically reduce the p*K*_a_ of the C(sp^3^)–H bond.^41,48,49^ However, the higher rate of reaction under light conditions suggests that [(dmpe)_2_Fe(η^2^-1)] 22 is predominantly consumed in the light-dependent pathway (Scheme 1a). Under dark conditions, the absolute concentration of [(dmpe)_2_Fe(η^2^-1)] 22 is highest at the end of the reaction which suggests that it is not a catalytically active species in this case. Further ^31^P{^1^H} NMR spectroscopic analysis showed rapid conversion from [(dmpe)_2_FeH_2_] 3 to [(dmpe)_2_Fe(η^2^-1)] 22 upon irradiation in the presence of methyl crotonate 1, with [(dmpe)_2_Fe(η^2^-1)] 22 being the major catalytically active species present after 1000 seconds, before gradually being consumed. ^1^H NMR spectroscopic analysis showed the gradual formation of (*E*),(*Z*)-2-ethylidene-3-methylpentanedioate 2 across the same period (see SI S9 SI Fig. 92 for further details), providing further support for the catalytic activity of [(dmpe)_2_Fe(η^2^-1)] 22 under light-mediated conditions.

Decomposition products were also identified: [{(dmpe)_2_FeH}_2_(μ-dmpe)]^2+^18 was characterised using a diagnostic resonance at *δ* 14.4 ppm in the ^31^P{^1^H} NMR spectrum, which matched reports from Field and co-workers.^39^ Absolute integrals assigned to [{(dmpe)_2_FeH}_2_(μ-dmpe)]^2+^18 rose rapidly until approximately 2000 seconds under both light and dark conditions, and increased more gradually thereafter (Fig. 3). The second decomposition product, which was the dominant iron species observed by ^31^P{^1^H} NMR at the end of the irradiated reaction, was found to be *trans*-[(dmpe)_2_FeH(CH_3_CH=CH–COO^−^)] 16, which was confirmed by single crystal X-ray diffraction (Fig. 4b). This indicated that ester demethylation of methyl crotonate 1 by [(dmpe)_2_Fe^0^] 4 was a significant decomposition pathway of catalytic species.^24,50^ While the demethylation mechanism remains unclear, *trans*-[(dmpe)_2_FeCl_2_] activated by sodium 2-ethylhexanoate has been reported to demethylate methoxy-substituted arenes,^24^ and iron-containing enzymes such as cytochrome P450 are known to demethylate other substrates, including lignin.^51,52^ Here, the significant formation of *trans*-[(dmpe)_2_FeH(CH_3_CH=CH–COO^−^)] 16 showed no major hindrance on the rate of reaction, suggesting that at 5 mol% catalyst loading there was sufficient [(dmpe)_2_FeH_2_] 3 present for the reaction to proceed. Crotonic acid 24 was observed in trace amounts suggesting that some *trans*-[(dmpe)_2_FeH(CH_3_CH=CH–COO^−^)] 16 decomposed to elemental iron. Separate investigations reacting [(dmpe)_2_FeH_2_] 3 with crotonic acid 24 yielded a red solution containing a complex mixture of iron complexes. Red crystals characterised by ^1^H–^31^P HMBC and ^1^H–^13^C HMBC were identified as *trans*-[(dmpe)_2_Fe(CH_3_CH=CH–COO^−^)_2_] 25 (see SI S6.5 and S9 SI Fig. 78–80). Complex 25 was catalytically inactive in the presence of methyl crotonate 1, in the presence and absence of irradiation (for further details see SI S9, SI Fig. 81).^53^ During *in situ* monitoring of methyl crotonate 1 dimerisation, no spectroscopic resonances corresponding to *trans*-[(dmpe)_2_Fe(CH_3_CH=CH–COO^−^)_2_] 25 were observed, suggesting that catalyst decomposition to *trans*-[(dmpe)_2_FeH(CH_3_CH=CH–COO^−^)] 16, *trans*-[(dmpe)_2_FeH(CH_3_O_2_CC_6_H_10_COO^−^)] 17 and [{(dmpe)_2_FeH}_2_(μ-dmpe)]^2+^18 was more likely.

Further investigations were conducted by irradiating [(dmpe)_2_FeH_2_] 3 in the presence of product (*E*),(*Z*)-2-ethylidene-3-methylpentanedioate 2 to test if competitive insertion to [(dmpe)_2_Fe^0^] 4 was inhibiting the reaction. Single crystals of *trans*-[(dmpe)_2_FeH(CH_3_O_2_CC_6_H_10_COO^−^)] 17 (Fig. 4c) deposited from a saturated pentane solution at −35 °C confirmed that demethylation of the conjugated ester in (*E*),(*Z*)-2-ethylidene-3-methylpentanedioate 2 was preferred over either C(sp^2^)–H oxidative addition or metallacyclopropane formation. This is attributed to the increased steric bulk of trisubstituted alkene (*E*),(*Z*)-2-ethylidene-3-methylpentanedioate 2 disfavouring the formation of an Fe(ii) metallocycle compared to disubstituted alkenes methyl crotonate 1 and methyl cinnamate 12 to form ([(dmpe)_2_Fe(PhCH=CHCO_2_Me)] 15 and [(dmpe)_2_Fe(η^2^-1)] 22, Fig. 1c and 4a respectively).

Under both light and dark conditions, *trans*-[(dmpe)_2_FeH(CH_2_CH=CHCO_2_Me)] 21 and *trans*-[(dmpe)_2_FeHY] 23 were observed by ^1^H–^31^P HMBC spectroscopy (Fig. 3a and b). Under light conditions *trans*-[(dmpe)_2_FeH(CH_2_CH=CHCO_2_Me)] 21 was at its highest concentration (0.01 M) at the start of the reaction before being rapidly consumed, whilst under dark conditions *trans*-[(dmpe)_2_FeH(CH_2_CH=CHCO_2_Me)] 21 grew in absolute concentration to 0.02 M at approximately 5000 seconds before gradually being consumed. *trans*-[(dmpe)_2_FeHY] 23 was present as a minor iron species in both light and dark conditions, with the highest absolute intensity observed at the start of the experiment and steadily decreasing to trace amounts after approximately 3000 seconds under light conditions. This suggests that *trans*-[(dmpe)_2_FeHY] 23 was either a short-lived intermediate or a coordination complex which does not play a significant role in methyl crotonate dimerisation.

Irradiation of a solution of [(dmpe)_2_FeH_2_] 3 in THF-*d*_8_ without methyl crotonate 1 at 365 nm for 11 days gave a mixture of iron(0) complexes similar to those reported by Tolman and co-workers.^54^ Specifically, [(dmpe)_5_Fe_2_] 26 and [(dmpe)_3_Fe] 27 (Scheme 4) were the major species present; the structure of the former was confirmed by single crystal X-ray diffraction (Fig. 4d and see SI S7.5, for more details).^55^ Addition of methyl crotonate 1 at 5 mol% catalyst loading in the absence of light saw immediate and complete conversion to (*E*),(*Z*)-2-ethylidene-3-methylpentanedioate 2 with 88 : 12 (*E*) : (*Z*) selectivity (see SI S9 SI Fig. 65 for more details). ^31^P{^1^H} NMR spectroscopic analysis showed that after methyl crotonate 1 addition, [(dmpe)_5_Fe_2_] 26 and [(dmpe)_3_Fe] 27 were completely consumed and only known decomposition products [{(dmpe)_2_FeH}_2_(μ-dmpe)]^2+^18 and [(dmpe)_3_FeH]^+^28 remained, making it highly likely that [(dmpe)_5_Fe_2_] 26 and [(dmpe)_3_Fe] 27 dimerise methyl crotonate 1 through a base-mediated pathway.^21,39^ It is important to note that neither [(dmpe)_5_Fe_2_] 26 and/or [(dmpe)_3_Fe] 27 were observed in the experiments containing [(dmpe)_2_FeH_2_] 3, methyl crotonate 1 and THF-*d*_8_, suggesting that these species are immediately consumed or do not form at all under reaction conditions where substrate and catalyst are added together.

**Scheme 3:**
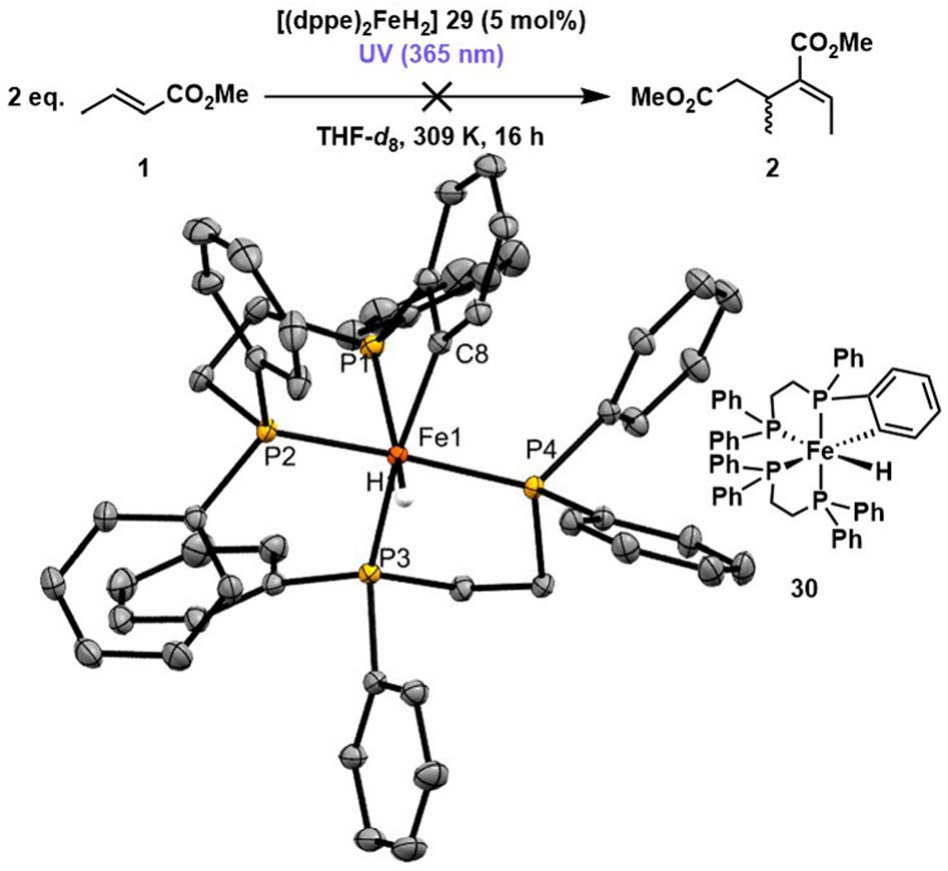
Using [(dppe)_2_FeH_2_] 29 as catalyst for methyl crotonate 1 dimerisation under UV (365 nm) irradiation for 16 hours yielded no (*E*),(*Z*)-2-ethylidene-3-methylpentanedioate 2. Crystals suitable for X-ray crystallography were grown from the reaction mixture at −35 °C and identified as [(dppe)FeH(Ph_2_PCH_2_CH_2_PPh(C_6_H_4_))] 30. Iron, carbon and phosphorus atoms on the molecular structures are coloured orange, grey and yellow respectively. Hydrogens have been omitted for clarity and thermal ellipsoids have been set at 50% probability.

**Scheme 4:**
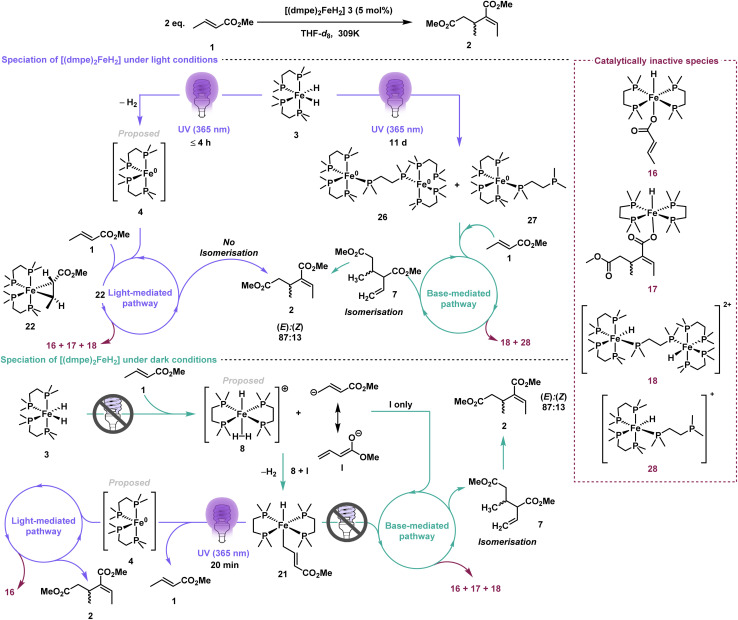
Speciation of [(dmpe)_2_FeH_2_] 3 into multiple structures dependent on the reaction conditions, with potential thermal activation in addition to the light-dependent and light-independent pathways.

In addition to investigating the substrate p*K*_a_, the catalyst p*K*_aH_ was varied using the bis(diphenylphosphinoethane) analogue [(dppe)_2_FeH_2_] 29, which has a lower p*K*_aH_ than [(dmpe)_2_FeH_2_] 3 (12.4 *versus* 15.9, respectively)^28^ (Scheme 3). [(dppe)_2_FeH_2_] 29 (5 mol%) did not catalyse the dimerisation of methyl crotonate 1 under photoirradiation for 1 hour, as there was no observable formation of (*E*),(*Z*)-2-ethylidene-3-methylpentanedioate 2 by ^1^H NMR spectroscopy. ^31^P{^1^H} NMR spectra and X-ray diffraction studies confirmed a novel crystal morphology of [(dppe)FeH(Ph_2_PCH_2_CH_2_PPh(C_6_H_4_))] 30 (Scheme 3), where a phenyl group has undergone C(sp^2^)–H insertion into Fe^0^ (see SI S7.6 for more details).^56^ Further irradiation overnight caused no change beyond some decomposition of [(dppe)_2_FeH_2_] 29 (5 mol%) to free dppe and an unidentified decomposition species. These observations give a clear indication that photoirradiation of [(dppe)_2_FeH_2_] 29 reductively eliminates dihydrogen, and that the resultant Fe^0^ species can undergo subsequent reaction with the dppe ligand. It is worth noting that a conformer of [(dppe)FeH(Ph_2_PCH_2_CH_2_PPh(C_6_H_4_))] 30 has been studied by Ackermann and co-workers in the context of C–H alkenylation.^56,57^ No reactivity was observed when methyl crotonate 1 and [(dppe)_2_FeH_2_] 29 were reacted under dark conditions, with only starting materials observed by NMR spectroscopy. The discovery of [(dppe)FeH(Ph_2_PCH_2_CH_2_PPh(C_6_H_4_))] 30 and the lack of reaction with methyl crotonate 1 give a valuable mechanistic insight that steric and ligand interactions play a significant role, rather than solely the p*K*_a_ determining the rate of reaction.^56^ This insight correlates with the studies probing the effect of steric bulk on the crotonate substrate (Fig. 1), and is reinforced by the fact that the phenyl C(sp^2^)–H bond of [(dppe)_2_FeH_2_] 29 was more labile than an alkenyl C(sp^2^)–H of 1, differing to when [(dmpe)_2_FeH_2_] 3 was used as the pre-catalyst.

All evidence pointed to a more complex mechanism than Komiya and co-workers' original proposal of the photoactivation of [(dmpe)_2_FeH_2_] 3 followed by C–H oxidative addition (Scheme 1).^26^ [(dmpe)_2_FeH_2_] 3 can speciate into multiple complexes under either light-mediated or base-mediated conditions (Scheme 4). In addition, [(dmpe)_2_FeH_2_] 3 could be thermally activated, giving small quantities of [(dmpe)_2_Fe(η^2^-1)] 22. Beyond the thermal pathway, both the base-mediated and the light-mediated pathways can dimerise methyl crotonate 1 to (*E*),(*Z*)-2-ethylidene-3-methylpentanedioate 2. It is worth noting that a possible explanation for the lower stereoselectivity observed here (*cf.* that reported by Komiya) supports the simultaneous occurrence of both light- and base-mediated pathways. Furthermore, irradiation of [(dmpe)_2_FeH_2_] 3 in the absence of methyl crotonate 1 gave [(dmpe)_5_Fe_2_] 26 and [(dmpe)_3_Fe] 27. This contrasts from the formation of [(dmpe)_2_Fe(η^2^-1)] 22 generated when [(dmpe)_2_FeH_2_] 3 and methyl crotonate 1 are irradiated together and shows that different iron species are formed depending on the order of addition of reagents.

## Conclusions

Mechanistic investigations have shown that methyl crotonate 1 can be selectively dimerised to (*E*),(*Z*)-2-ethylidene-3-methylpentanedioate 2 with [(dmpe)_2_FeH_2_] 3, incorporating both light-mediated and base-mediated mechanisms. Monitoring by NMR spectroscopy showed that other crotyl derivatives, including those with vinylogous C–H bonds of different p*K*_a_s, could also be dimerised by [(dmpe)_2_FeH_2_] 3 under dark conditions with the exception of methyl cinnamate, which highlights the role of the base-mediated pathway. Isolation of several key iron intermediates, in addition to observation of other iron species *in situ*, has delivered valuable insight into the behaviour of [(dmpe)_2_FeH_2_] 3 under thermal and photoirradiation reaction conditions and in the presence of an alkene. Varying the catalyst to [(dppe)_2_FeH_2_] 29 demonstrated how both the steric bulk and the basicity of iron hydride complexes influences the balance between light-mediated and base-mediated pathways. Gaining mechanistic insight into both light- and base-mediated pathways can be used to enhance catalyst performance, for example by revealing the high catalytic activity of Fe^0^ complexes such as [(dmpe)_5_Fe_2_] 26 in converting methyl crotonate 1 into (*E*),(*Z*)-2-ethylidene-3-methylpentanedioate 2. Overall, these studies highlight how iron–phosphorous complexes offer multiple possible pathways for C–H functionalisations and open the door to using non-light activated methods for this transformation, which remain extremely rare.

## Author contributions

J. A. Garden and S. P. Thomas: conceptualisation, supervision, writing and editing. J. H. P. Cockcroft: synthesis, characterisation, and writing. A. Flook and P. J. Boaler: *in situ* monitoring by NMR spectroscopy. G. S. Nichol: single crystal X-ray analyses and the refinement of the structures. J. Holt and J. Smit for supervision (JHPC) and productive conversations. All authors have given approval to the final version of the manuscript.

## Conflicts of interest

The authors have no conflicts of interest to declare.

## Supplementary Material

SC-OLF-D5SC07490H-s001

SC-OLF-D5SC07490H-s002

## Data Availability

CCDC 2453128–2453133 contain the supplementary crystallographic data for this paper.^58*a*–*f*^ Supplementary information: synthesis details, characterisation data for compounds including NMR spectroscopy, mass spectrometry, and single crystal X-ray diffraction data. See DOI: https://doi.org/10.1039/d5sc07490h.
