# Supplementary material for: Molecular-level insight into the multiple mechanistic pathways in iron-catalysed alkene dimerisation
Source: Chem Sci. 2025 Oct 29;16(47):22394–406. doi: 10.1039/d5sc07490h (PMC12571196; doi:10.1039/d5sc07490h)
Supplement: SC-016-D5SC07490H-s001 [file SC-016-D5SC07490H-s001.pdf]

Supplementary Information (SI) for Chemical Science.
This journal is © The Royal Society of Chemistry 2025

Molecular-level insight into the multiple mechanistic pathways in iron-catalysed alkene dimerisation

Joseph H. P. Cockcroft[a], Annabel Flook[a], Patrick Boaler[a], Gary S. Nichol[a], Jarle Holt[b], Joost Smit[b], Jennifer A. Garden[a]*, and Stephen P. Thomas[a]*

[a]EaStCHEM School of Chemistry, Joseph Black Building, The University of Edinburgh, David Brewster Road, Edinburgh, EH9 3FJ

[b]Johnson Matthey, Technology Centre, Princeton Drive, Stockton-on-Tees, TS17 6PY

Corresponding Authors: j.garden@ed.ac.uk, stephen.thomas@ed.ac.uk

Supplementary information

Contents

S1 General experimental ... 4

S1.1 Reaction setup ... 4

S1.2 Solvents ... 4

S1.3 NMR spectroscopy ... 4

S1.6 Chemicals ... 5

S1.7 Chromatography ... 5

S1.8 X-ray crystallography ... 5

S1.9 Equipment setup ... 7

S2 Pre-catalyst synthesis ... 9

S2.1 [(dmpe)$_2$FeCl$_2$] ... 9

S2.2 [(dmpe)$_2$FeH$_2$] **3** ... 9

S3 Deuterated substrate synthesis ... 10

S3.1 Acetaldehyde-*2,2,2-d$_3$* ... 10

S3.2 Methoxycarbonylmethyltriphenylphosphonium bromide ... 10

S3.3 Methyl (triphenylphosphoranylidene)acetate ... 11

S3.4 Methyl (triphenylphosphoranylidene)acetate-*d$_1$* ... 11

S3.5 Methyl crotonate-*d*$_3$ ... 12

S3.6 Methyl crotonate-*d*$_1$ ... 12

S3.7 (*E*),(*Z*)-Dimethyl 2-ethylidene-3-methylpentanedioate **2** ... 13

S3.8 (*E*),(*Z*)-Diethyl 2-ethylidene-3-methylglutarate **10** ... 13

S3.9 (*E*),(*Z*)-2-(1-propenyl)-3-ethylglutaric acid dimethyl ester **13** ... 14

S4 General procedure for crotonate dimerisation experiments ... 15

S4.1 Using different light wavelengths to dimerise methyl crotonate ... 15

S4.2 *ex situ* General procedure for reaction monitoring ... 15

S4.3 General procedure for variable time normalised analysis (VTNA) ... 16

S4.4 General procedure for crotyl substrate dimerisation ... 17

S4.5 General procedure for *in situ* monitoring ... 17

S4.6 General procedure for 16-hour no-light dimerisation experiments ... 20

S4.7 General procedure for control experiments using methyl 3-butanoate **19** ... 20

S5 Iron species identified throughout *in situ* monitoring ... 21

S5.1 [(dmpe)$_2$Fe(PhCH=CHCO$_2$Me)] **15** ... 21

S5.2 *trans*-[(dmpe)$_2$FeH(CH$_3$CH=CH-COO$^-$)] **16** ... 21

S5.3 *trans*-[(dmpe)$_2$FeH(CH$_3$O$_2$CC$_6$H$_{10}$COO$^-$)] **17** ... 22

S5.4 [{(dmpe)$_2$FeH}$_2$(μ-dmpe)$_2$]$^{2+}$ **18** ... 22

S5.5 *trans*-[(dmpe)$_2$FeH(CH$_2$CH=CHCO$_2$Me)] **21** ... 22

S5.6 [(dmpe)$_2$Fe(η^2-1)] **22** ... 23

S5.7 *trans*-[(dmpe)$_2$FeHY] **23** ... 23

S5.8 [(dmpe)$_5$Fe$_2$] **26** ... 23

S5.9 [(dmpe)$_3$Fe] **27** ... 23

S6 Determining whether species observed *in situ* are catalytically active 24
S6.1 [(dmpe)$_2$Fe(PhCH=CHCO$_2$Me)] **15** 24
S6.2 *trans*-[(dmpe)$_2$FeH(CH$_3$O$_2$CC$_6$H$_{10}$COO$^-$)] **17** 24
S6.3 *trans*-[(dmpe)$_2$FeH(CH$_2$CH=CHCO$_2$Me)] **21** 24
S6.4 [(dmpe)$_2$Fe(η^2-1)] **22** (formed *in situ*) 25
S6.5 *trans*-[(dmpe)$_2$Fe(CH$_3$CH=CH-COO$^-$)$_2$] **25** 25
S7 Crystallographic data 27
S7.1 [(dmpe)$_2$Fe(PhCH=CHCO$_2$Me)] **15** 27
S7.2 *trans*-[(dmpe)$_2$FeH(CH$_3$CH=CH−COO$^-$)] **16** 28
S7.3 *trans*-[(dmpe)$_2$FeH(CH$_3$O$_2$CC$_6$H$_{10}$COO$^-$)] **17** 30
S7.4 [(dmpe)$_2$Fe(η^2-1)] **22** 31
S7.5 [(dmpe)$_5$Fe$_2$] **26** 33
S7.6 [(dppe)FeH(Ph$_2$PCH$_2$CH$_2$PPh(C$_6$H$_5$)] **30** 34
S8 Initial results 35
S8.1 Initial kinetic analysis comparing proteo- and deutro-substrates 35
S8.2 Variable time normalisation analysis (VTNA) 36
S9 NMR spectra 40
S10 References 124

S1 General Experimental

S1.1 Reaction Setup

All air and moisture sensitive manipulations were carried out using standard vacuum line and Schlenk techniques, or in a Vigor glovebox containing a purified argon atmosphere (≤0.1 ppm H_2O/O_2).

S1.2 Solvents

All solvents for air- and moisture sensitive techniques were obtained from an anhydrous solvent system (Innovative Technology) and stored over 4Å molecular sieves. Reaction solvents tetrahydrofuran (THF) (Fisher, HPLC grade) and ether (Et_2O) (Fisher, BHT stabilized ACS grade) were dried by percolation through two columns packed with neutral alumina under a positive pressure of argon. Reaction solvent toluene (ACS grade) was dried by percolation through a column packed with neutral alumina and a column packed with Q5 reactant (supported copper catalyst for scavenging oxygen) under a positive pressure of argon. All glassware was cleaned using base (KOH, ^{i}PrOH) then acid (HCl aq) baths. All reported reaction temperatures correspond to external bath temperatures. Room temperature (rt) was approximately 22 °C.

S1.3 NMR Spectroscopy

^{1}H, ^{2}H, ^{13}C and ^{31}P NMR spectra were recorded on Bruker Avance III 400 and 500 MHz spectrometers. High resolution magic angle spinning was acquired using a TXI-HRMAS probe, optimised using a KBr standard. Chemical shifts are reported in parts per million (ppm). ^{1}H and ^{13}C NMR spectra were referenced to the residual deuterated solvent peak (^{1}H residual peaks in ppm, ^{13}C residual peaks in ppm) $CHCl_3$ (7.27 ppm, 77.00 ppm); C_6D_6 (7.16 ppm, 128.06 ppm) D_2O (4.79 ppm); THF-d_8 (3.58 and 1.72 ppm, 67.21 and 25.31 ppm).[1] Multiplicities are indicated by app. (apparent), br. (broad), s (singlet), d (doublet), t (triplet), q (quartet), quin. (quintet), sext. (sextet), sept. (septet). Coupling constants, J, are reported in Hertz and rounded to the nearest 0.1 Hz. Integration is provided and assignments are indicated. NMR spectra for *in situ* monitoring were acquired on a Bruker Ascend 400 MHz NMR spectrometer fitted with a broadband direct-detect Cryoprobe Prodigy. ^{31}P T_1 measurements were determined using FLIPS.[1] Using the approximated monitoring time of 2 h and T_1 max = 5 s for the reacting species, NMR parameters for reaction monitoring were determined using the method laid out by Flook and Lloyd-Jones.[2]

S1.4 Mass Spectroscopy

All HRMS was performed by the Scottish Instrumentation and Resource Centre for Advanced Mass Spectrometry (SIRCAMS) of the University of Edinburgh using either electron impact (EI) or electrospray ionisation (ESI) techniques. Accurate masses are calculated using the most abundant isotopes of each element

S1.5 Infrared Spectroscopy

All ATR-IR was performed on a Nicole Summit X (Serial No. BGA2311781) with a Diamond crystal, a KBr beamsplitter and a DTGS KBr detector. All runs were scanned 16 times and calibrated against a background scan.

S1.6 Chemicals

All reagents were purchased from Sigma Aldrich, Alfa Aesar, Acros Organics, Tokyo Chemical Industries UK, and Apollo Scientific or synthesised within the laboratory. Chloroform, benzene-d_6 and deuterium oxide were purchased from Sigma-Aldrich and stored over 4Å molecular sieves. THF-d_8 was dried using sodium benzophenone ketyl, distilled and stored over 4Å molecular sieves. Iron(II) chloride was purchased from Strem Chemicals Inc. (UK); anhydrous iron chloride, 98% (product number 93-2631. Lot 19226800, 44.00000% Fe, expect 44.059%). *Trans*-methyl crotonate 98% was purchased from Fisher Chemicals Inc., distilled and stored over 4Å molecular sieves. Ethyl crotonate, methyl (*E*)-2-pentenaote and methyl (*2E*)-4-methylpent-2-enaote were purchased from Fluorochem Ltd., dried over calcium hydride, distilled and stored over 4Å molecular sieves. Acetaldehyde (99%) was purchased from Fisher Chemicals Inc. and distilled before each use. Crotonic acid was purchased from Sigma Aldrich and sublimed under vacuum at 40 °C for 1 hour before each use. Triphenylphosphine and methyl bromoacetate were purchased from Sigma-Aldrich and used without further purification. Methyl 3-butanoate was purchased from Fluorochem, sparged and stored under 4Å molecular sieves before use.

S1.7 Chromatography

Column chromatography was carried out on a Teledyne ISCO CombiFlash NextGen 300+ using RediSep Rf normal phase silica flash columns (12, 25, 40, or 80 g; 20-40 microns). Substrates were purified using PET ether and EtOAc on a gradient of 100:0 to 0:100 with flow rates of 10-110 mL min^{-1} depending on the size of column and ΔR_f. Further experimental and refinement details are given in the CIF files.

S1.8 X-ray crystallography

For [$(dmpe)_2Fe(\eta^2$-**1**)] **22**: A suitable crystal was selected and mounted on a MITIGEN holder in Paratone oil. on a Rigaku Oxford Diffraction SuperNova diffractometer. The crystal was kept at a steady T = 120.00 K during data collection. The structure was solved with the ShelXS[3] solution program using heavy methods and by using Olex2 1.5-beta as the graphical interface.[4] The model was refined with ShelXL 2018/3[5] using full matrix least squares minimisation on $\boldsymbol{F^2}$.

For [$(dmpe)_2Fe(PhCH{=}CHCO_2Me)$] **15,** *trans*-[$(dmpe)_2FeH(CH_3O_2CC_6H_{10}COO^-)$] **17** and *trans*-[$(dmpe)_5Fe_2$] **26**: A suitable crystal was selected and mounted on a MITIGEN holder in perfluoro-ether oil on a XtaLAB Synergy, Single source at home/near, HyPix-Arc 100 diffractometer. The crystal was kept at a steady T = 100.00(10) K during data collection. The structure was solved with the ShelXS solution program[3] using direct methods and by using Olex2 1.5-beta as the

graphical interface.[4] The model was refined with ShelXL 2018/3[5] using full matrix least squares minimisation on $\boldsymbol{F}^2$. Further experimental and refinement details are given in the CIF files.

trans-[$(dmpe)_2FeH(CH_3CH{=}CH\text{-}COO^-)$] **16** and [$(dppe)FeH(Ph_2PCH_2CH_2PPh(C_6H_4))$] **30**: A suitable crystal was selected and mounted on a MITIGEN holder in Paratone oil on a Bruker D8 VENTURE diffractometer. The crystal was kept at a steady T = 100.00 K during data collection. The structure was solved with the ShelXT 2018/2[6] solution program using dual methods and by using Olex2 1.5-beta as the graphical interface.[4] The model was refined with ShelXL 2018/3[5] using full matrix least squares minimisation on $\boldsymbol{F}^2$.

S1.9 Equipment setup

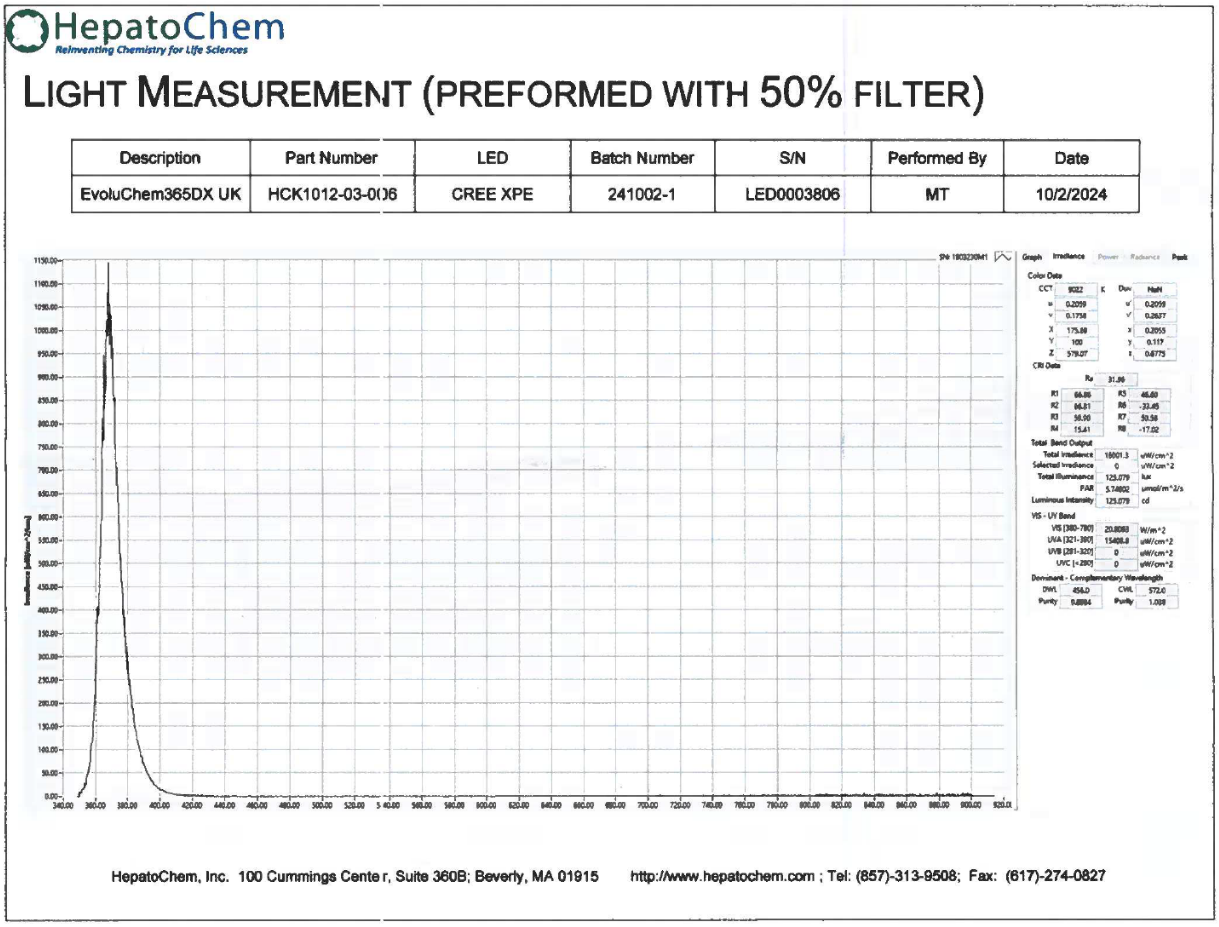

Supplementary Figure 1 – Emission spectra of HepatoChem P301-30-1 365 nm.

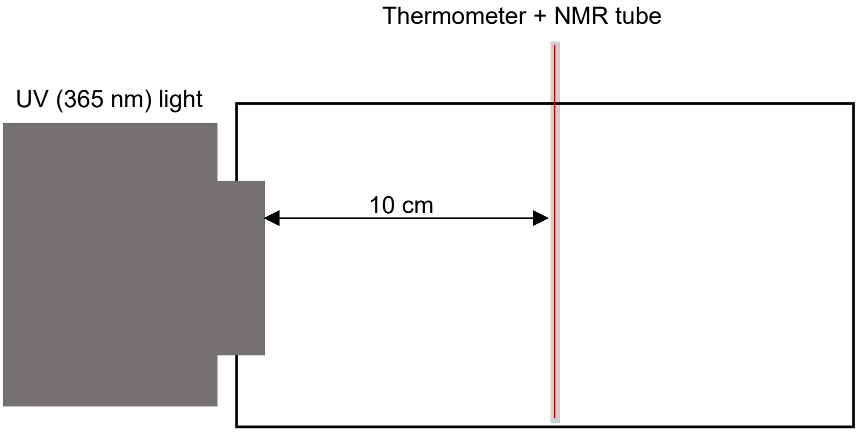

Supplementary Figure 2 – Illustration of the reaction monitoring setup used for kinetic analysis and VTNA.

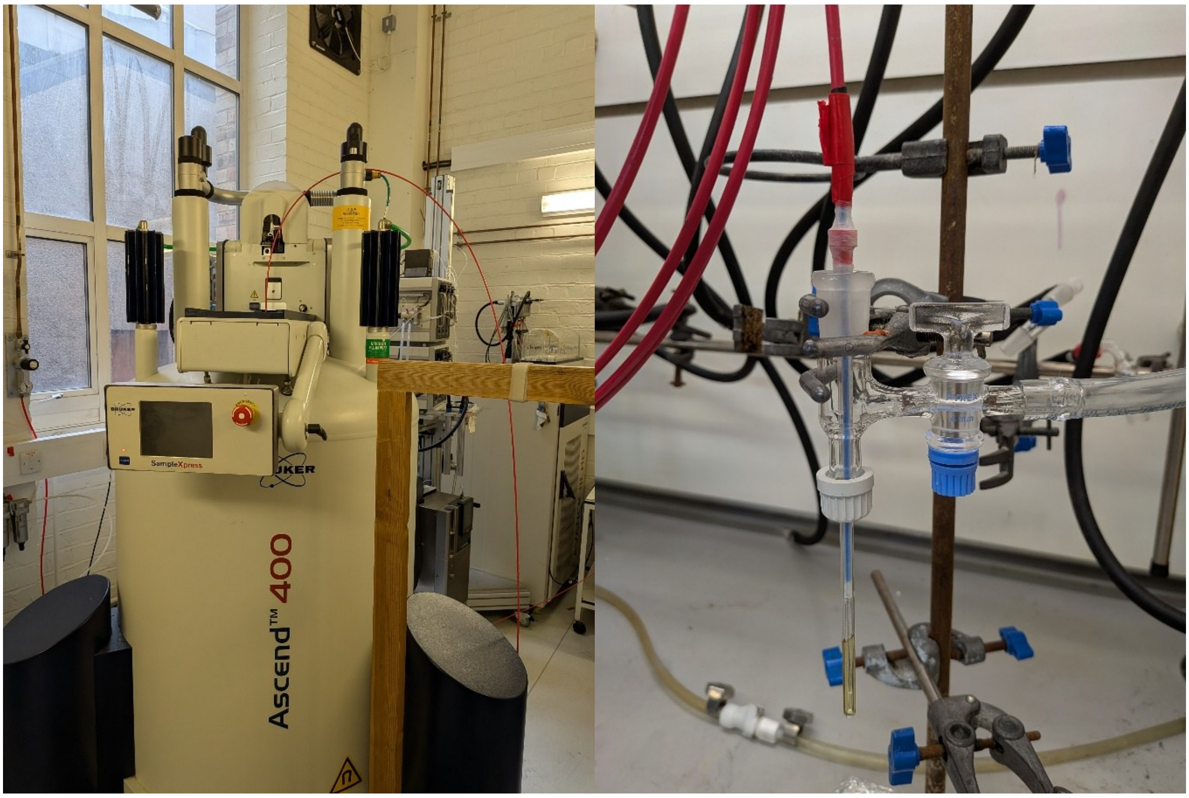

Supplementary Figure 3 – NMR setup used for ^{1}H and $^{31}P\{^{1}H\}$ NMR *in situ* monitoring

S2 Pre-catalyst Synthesis

S2.1 [$(dmpe)_2FeCl_2$]

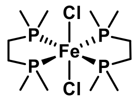

By modification of the method reported by Colbran and co-workers,[7] 1,2-bis(dimethylphosphino)ethane (0.500 g, 3.33 mmol, 2 eq.) was added to a solution of anhydrous iron dichloride (0.210 g, 1.67 mmol, 1 eq.) in anhydrous THF (10 mL) under an Ar atmosphere and the solution left to stir for 16 h at room temperature. The resultant solution was then filtered and the solvent removed *in vacuo.* The resultant crude solid was extracted and recrystallized with toluene to give [$(dmpe)_2FeCl_2$] as green plate crystals (0.550 g, 1.19 mmol, 77%).

^{1}H NMR (400 MHz, THF-d_8) δ 2.18 (s, 8H), 1.42 (s, 24H).

^{31}P{^{1}H} NMR (202 MHz, $CDCl_3$) δ 59.0 (s).

Spectroscopic data were in accordance with those previously reported.[7]

S2.2 [$(dmpe)_2FeH_2$] 3

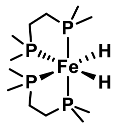

By modification of a procedure reported by Darcel and co-workers,[8] a lithium triethylborohydride solution (1.7 M in THF, 0.91 mL, 1.5 mmol, 2.2 equiv.) was added dropwise to a stirred solution of [$FeCl_2(dmpe)_2$] (300 mg, 0.70 mmol, 1 eq.) in anhydrous diethyl ether (10 mL). The mixed was stirred for 2 hours with an observed colour change to yellow/orange. Conversion was monitored by ^{1}H and ^{31}P NMR to ensure full conversion of the [$(dmpe)_2FeHCl$] intermediate. The solvent was removed *in vacuo* and the resultant solid extracted with pentane (3 x 10 mL). The solution was evaporated to dryness *in vacuo*. The crude product was sublimated (40 – 80 °C, 1 x 10^{-2} mbar for several hours) to yield [$(dmpe)_2FeH_2$] as a pale-yellow precipitate in high purity (149 mg, 0.440 mmol, 64%).

^{1}H NMR (500 MHz, THF-d_8) δ 1.75 – 1.58 (m, 4H), 1.40 (s, 8H), 1.33 (s, 8H), 1.23 (d, J = 4.9 Hz, 6H), 1.19 (d, J = 5.8 Hz, 6H), −14.13 (ddq, J = 58.2, 38.6, 19.3 Hz, 2H). *Hydride region only*

^{31}P{^{1}H} NMR (202 MHz, THF-d_8) δ 77.2 (t, J = 26.7 Hz), 67.5 (t, J = 26.7 Hz).

^{13}C NMR (126 MHz, THF-d_8) δ 35.5, 32.7, 27.0, 23.0, 22.3.

Spectroscopic data were in accordance with those previously reported.[8]

S3 Deuterated substrate synthesis

S3.1 Acetaldehyde-*2,2,2-d3*

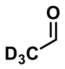

According to a procedure reported by Seeger and co-workers,[9] acetaldehyde (4.46 mL, 101 mmol, 1 eq.) was added to a tube with a stirred solution of pyridine (0.64 mL) and D_2O (14.9 mL, 0.826 mol, 8.18 eq.). The tube was sealed and heated to 60 ℃ for 72 hours. The mixture was then distilled and the process repeated twice more to give acetaldehyde-2,2,2-d_3 (1.68 g, 33.4 mmol, 33%) as a colourless liquid which showed 98% D-incorporation by ^{1}H NMR spectroscopy.

^{1}H NMR: (500 MHz, $CDCl_3$) δ 9.80 (s, 1H) 2.17 (m, <1H, *trace protons*).

^{2}H NMR (77 MHz, CH_2Cl_2) δ 2.15 (s).

^{13}C NMR (126 MHz, CD_2Cl_2) δ 197.5, 30.2 (sept, J = 19.7 Hz).

Spectroscopic data were in accordance with those previously reported.[9]

S3.2 Methoxycarbonylmethyltriphenylphosphonium bromide

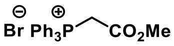

According to a procedure reported by Lugtenburg and co-workers,[10] methyl bromoacetate (6.0 mL, 63 mmol, 1 eq.) was added dropwise to a stirred solution of triphenylphosphine (16.5 g, 62.8 mmol, 1 eq.) in ethyl acetate (100 mL) and left overnight. The white suspension was then filtered and washed with pentane (3 x 10 mL) and dried under vacuum for 4 hours to give methoxycarbonylmethyltriphenylphosphonium bromide (24.3 g, 58.5 mmol, 93%) as a white amorphous solid.

^{1}H NMR (500 MHz, $CDCl_3$) δ 7.92-7.88 (dd, J = 13.2, 1.3 Hz, 6H), 7.79-7.76 (tq, J = 7.5, 1.2 Hz, 3H), 7.69-7.65 (dt, J = 8.0, 3.7 Hz, 6H), 5.68 (d, J = 13.6 Hz, 2H), 3.60 (s, 3H).

^{13}C NMR (126 MHz, $CDCl_3$) δ 165.5, 135.3, 134.3 (d, J = 10.9 Hz), 130.4 (d, J = 13.3 Hz), 118.2 (d, J = 89.3 Hz), 53.6, 33.5 (d, J = 58.4 Hz).

^{31}P{^{1}H} NMR (202 MHz, $CDCl_3$) δ 20.3 (s).

Spectroscopic data were in accordance with those previously reported.[11]

S3.3 Methyl (triphenylphosphoranylidene)acetate

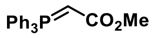

According to a procedure reported by Lugtenburg and co-workers,[10] methoxycarbonylmethyltriphenylphosphonium bromide (12.8 g, 30.8 mmol, 1 eq.) was dissolved in dichloromethane (50 mL) and placed in a 250 mL separating funnel. To the funnel was added 2 M NaOH solution (100 mL) and the biphasic mixture vigorously shaken. The aqueous layer was then washed with CH_2Cl_2 (4 x 25 mL) and the organic layers combined, dried with $MgSO_4$ and filtered. The solvent was then removed *in vacuo* to give methyl (triphenylphosphoranylidene)acetate in two rotameric forms as a white amorphous solid (8.35 g, 25.0 mmol, 81%).

^{1}H NMR (500 MHz, $CDCl_3$) δ 7.80 – 7.36 (m, 15H), 3.57 (75%) + 3.30 (25%) (s,3H), *ylide C−H not observed.*

^{13}C NMR (126 MHz, $CDCl_3$) δ 171.9, 133.0 (d, *J* = 10.0 Hz), 132.0, 128.8 (d, *J* = 12.2 Hz), 127.8 (d, *J* = 91.9 Hz), 49.9, 29.6 (d, *J* = 129.2 Hz).

^{31}P{^{1}H} NMR (202 MHz, $CDCl_3$) δ 18.0 (s), 16.5 (s).

Spectroscopic data were in accordance with those previously reported.[10]

S3.4 Methyl (triphenylphosphoranylidene)acetate-*d$_1$*

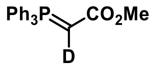

According to a modification of a procedure reported by Kishida and co-workers,[12] methyl(triphenylphosphoranylidene)acetate (5 g, 15.0 mmol, 1 eq.) was dissolved in dichloromethane (20 mL) and left to stir for 10 minutes. D_2O was then added (2.0 mL, 110 mmol, 7.4 eq.) and the mixture stirred vigorously overnight at room temperature. The mixture was then placed in a separating funnel and the organic layer extracted. The aqueous layer was then washed with additional dichloromethane (3 x 10 mL), the organic layers combined and the solvent was removed *in vacuo*. The process was repeated four times to give methyl (triphenylphosphoranylidene)acetate-*d$_1$* (2.54 g, 7.57 mmol, 50%) in two rotameric forms as a white amorphous solid which showed 99% D-incorporation by ^{1}H NMR spectroscopy.

^{2}H NMR (500 MHz, C_6D_6) δ 2.91 (s).

^{13}C NMR (126 MHz, C_6D_6) δ 172.4 (d, *J* = 14.6 Hz), 133.4 (d, *J* = 10.0 Hz), 132.4 (d, *J* = 10.0 Hz), 131.9, 128.9 (d, *J* = 12.0 Hz), 128.7 (d, *J* = 12.0 Hz), 49.9 (ap d, *J* = 3.3 Hz), 29.5 (dt, *J* = 128.5, 24.7 Hz).

^{31}P{^{1}H} NMR (202 MHz, C_6D_6) δ 18.2 (s), 16.2 (s).

Spectroscopic data were in accordance with those previously reported.[12]

S3.5 Methyl crotonate-d_3

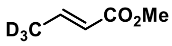

According to a modification of a procedure by Kingston and co-workers,[13] methyl (triphenylphosphoranylidene)acetate (11.96 g, 35.70 mmol, 1.07 eq.) in pentane (20 mL) and cooled to 0 °C. Acetaldehyde-*2,2,2-d_3* (1.58 g, 33.6 mmol, 1 eq.) was added dropwise and the mixture allowed to warm up to room temperature. The mixture was sealed and left to stir overnight at room temperature. The crude mixture was then vacuum distilled and the solvent was removed *in vacuo* to give methyl crotonate-d_3 (1.42 g, 13.7 mmol, 41%) as a colourless liquid which showed 93% D-incorporation by ^{1}H NMR spectroscopy.

^{1}H NMR (500 MHz, C_6D_6) δ 6.87 (dsept, *J* = 15.6, 0.9 Hz, 1H), 5.77 (d, *J* = 15.4 Hz, 1H), 3.41 (s, 3H).

^{2}H NMR (92 MHz, C_6D_6) δ (*cis*-isomer) 1.93 (d, *J* = 1.1 Hz), (*trans*-isomer) 1.22 (d, *J* = 1.0 Hz).

^{13}C NMR (126 MHz, C_6D_6) δ 169.1, 131.9 (t, *J* = 24.0 Hz), 131.0 (t, *J* = 24.1 Hz), 53.6, 23.3 (sept, *J* = 19.2 Hz).

Spectroscopic data were in accordance with those previously reported.[13]

S3.6 Methyl crotonate-d_1

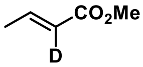

According to a modification of a procedure reported by Kingston and co-workers,[13] methyl (triphenylphosphoranylidene)acetate-d_1 (3.40 g, 10.13 mmol, 1 eq.) was suspended in pentane (20 mL) and cooled to 0 °C. Acetaldehyde (1 mL, 17.9 mmol, 1.8 eq.) was added dropwise and the mixture allowed to warm up to room temperature. The mixture was sealed and left to stir overnight at room temperature. The crude mixture was then vacuum distilled and the solvent was removed *in vacuo* to give methyl crotonate-d_1 (0.566g g, 5.60 mmol, 55%) as a colourless liquid, which showed 95% D-incorporation by ^{1}H NMR spectroscopy.

^{1}H NMR (601 MHz, C_6D_6) 6.86 (qt, *J* = 7.0, 2.4 Hz, 1H), 3.40 (s, 3H), 1.28 (d, *J* = 7.0 Hz, 3H)

^{13}C NMR (151 MHz, C_6D_6) 166.2, 144.0, 122.2 (t, *J* = 22.3 Hz), 50.7, 17.3

Spectroscopic data were in accordance with those previously reported.[13]

S3.7 (*E*),(*Z*)-Dimethyl 2-ethylidene-3-methylpentanedioate 2

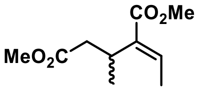

According to a modification of a procedure reported by Waymouth and co-workers,[14] potassium *tert*-butoxide (53 mg, 0.47 mmol, 5 eq.) was suspended in THF (2 mL) and methyl crotonate (0.944 g, 9.43 mmol, 100 eq.) was added dropwise (*Warning: exothermic reaction*). The mixture went from a cloudy colourless solution to a dark clear yellow solution after 15 seconds. The mixture was quenched with dilute HCl (10 mL, 1 M in H_2O) and the crude product extracted using diethyl ether. The mixture was then dried over Na_2SO_4, filtered and the solvent removed *in vacuo*. Flash column chromatography (10% ethyl acetate: 90% hexanes by volume) afforded 2-ethylidene-3-methylpentanedioate **2** (800 mg, 4.00 mmol, 85%) as a colourless oil in an 80:20 (*E*):(*Z*) isomeric ratio.

^{1}H NMR (601 MHz, C_6D_6) of (*E*):(*Z*) isomer mixture: (*E*)-isomer δ 6.75 (q, *J* = 7.2 Hz, 1H), 3.38 (s, 3H), 3.31 (s, 3H), 3.12 (h, *J* = 7.0 Hz, 1H), 2.83 (dd, *J* = 15.6, 8.1 Hz, 1H), 2.56 (dd, *J* = 15.6, 6.8 Hz, 1H), 1.55 (d, *J* = 7.3 Hz, 3H), 1.19 (d, *J* = 7.2 Hz, 3H).

(*Z*)-isomer 5.70 (dq, *J* = 7.2, 1.0 Hz, 1H), 3.65 (h, J = 6.1 Hz, 1H), 3.38 (s, 3H), 3.34 (s, 3H), 2.51 (dd, *J* = 15.4, 6.4 Hz, 1H), 2.25 (dd, *J* = 15.4, 8.2 Hz, 1H), 1.78 (d, *J* = 7.2 Hz, 3H), 1.06 (d, *J* = 7.0 Hz, 3H).

^{13}C NMR (151 MHz, C_6D_6) of (*E*):(*Z*) isomer mixture: (*E*)-isomer δ 172.8, 166.9, 138.0, 136.1, 50.9, 50.9, 39.5, 29.5, 19.1, 13.8.

(*Z*)-isomer 172.2, 167.9, 137.2, 134.1, 51.0, 50.7, 40.8, 34.9, 19.9, 15.7.

HRMS (ESI$^+$) m/z Calcd for $C_{10}H_{17}O_4$ 201.1121 $[M+H]^+$; Found 201.1114 (−3.68 ppm).

Spectroscopic data were in accordance with those previously reported.[14]

S3.8 (*E*),(*Z*)-Diethyl 2-ethylidene-3-methylglutarate 10

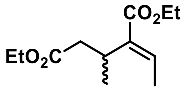

According to a modification of a procedure reported by Waymouth and co-workers,[14] a Schlenk flask was charged with potassium *tert*-butoxide (45.5 mg, 0.406 mmol, 5 eq.) in THF (2 mL) and ethyl crotonate (0.918 g, 8.06 mmol, 100 eq.) added dropwise (*Warning: exothermic reaction*). The mixture went from cloudy colourless → yellow → orange → brown solution after 15 seconds. The mixture was quenched with dilute HCl (10 mL, 1 M in H_2O) and the crude product extracted using diethyl ether. The mixture was then dried over Na_2SO_4, filtered and the solvent removed *in vacuo*. Flash column chromatography (10% ethyl acetate: 90% hexanes by volume) afforded (*E*), (*Z*)- diethyl 2-ethylidene-3-methylglutarate **10** (469 mg, 2.05 mmol, 51%) as a colourless oil in an 80:20 (*E*):(*Z*) isomeric ratio.

^{1}H NMR (601 MHz, $CDCl_3$) of (*E*):(*Z*) isomer mixture: (*E*)-isomer δ 6.81 (q, *J* = 7.2 Hz, 1H), 4.17 (q, *J* = 7.1 Hz, 2H), 4.14 – 4.05 (m, 3H), 3.29 (sept, *J* = 7.1 Hz, 1H), 2.71 (dd, *J* = 15.3, 7.9 Hz, 1H), 2.57 (dd, *J* = 15.3, 7.2 Hz, 1H), 1.84 (d, *J* = 7.2 Hz, 3H), 1.29 (t, *J* = 7.1 Hz, 3H), 1.23 – 1.19 (m, 5H).

(*Z*)-isomer δ 5.94 (dq, *J* = 7.2, 1.0 Hz, 1H), 4.23 (ddd, *J* = 17.9, 7.3, 0.4 Hz, 2H), 3.03 (sept, *J* = 7.2 Hz, 1H), 2.55 (dd, *J* = 15.3, 6.8 Hz, 3H), 2.33 (dd, *J* = 15.1, 8.3 Hz, 1H), 1.90 (dd, *J* = 7.1, 0.8 Hz, 3H), 1.32 (t, *J* = 7.0 Hz, 3H), 1.23 (t, *J* = 7.1 Hz, 3H), 1.12 (d, *J*= 6.9 Hz, 3H).

^{13}C NMR (151 MHz, $CDCl_3$) of (*E*):(*Z*) isomer mixture: (*E*)-isomer δ 173.1, 167.2, 137.9, 136.0, 60.3, 60.2, 39.8, 29.3, 19.2, 14.4, 14.4, 14.1.

***(Z)*-isomer** δ 172.6, 168.2, 137.1, 133.6, 60.4, 60.3, 41.1, 34.9, 28.2, 19.9, 15.7, 14.4.

HRMS (ESI$^+$) m/z Calcd for $C_{12}H_{20}O_4Na$ 251.1254 $[M+Na]^+$; Found 251.1252 (−0.72 ppm).

ATR-IR 2980, 2936, 2907, 2876, 1732, 1705, 1640 cm^{-1}.

S3.9 (*E*),(*Z*)-2-(1-propenyl)-3-ethylglutaric acid dimethyl ester 13

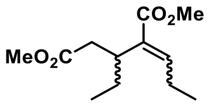

According to a modification of a procedure reported by Waymouth and co-workers,[14] a Schlenk flask was charged with potassium *tert*-butoxide (45.5 mg, 0.406 mmol, 5 eq.) in THF (2 mL) and methyl (*E*)-2-pentenaote (1.039 g, 8.11 mmol, 100 eq.) was added dropwise (*Warning: exothermic reaction*). The mixture went from cloudy colourless → yellow solution after 15 seconds. The mixture was quenched with dilute HCl (10 mL, 1 M in H_2O) and the crude product extracted using diethyl ether. The mixture was then dried over Na_2SO_4, filtered and the solvent removed *in vacuo*. Flash column chromatography (10% ethyl acetate: 90% hexanes by volume) afforded (*E*), (*Z*)-2-(1-propenyl)-3-ethylglutaric acid dimethyl ester **13** (550 mg, 2.41 mmol, 60%) as a colourless oil in an 87:13 (*E*):(*Z*) isomeric ratio.

^{1}H NMR (601 MHz, C_6D_6) of (*E*):(*Z*) isomer mixture: (*E*)-isomer δ 6.81 (t, *J* = 7.5 Hz, 1H), 3.70 (s, 3H), 3.61 (s, 3H), 3.10 – 2.95 (m, 1H), 2.72 (dd, *J* = 15.3, 8.5 Hz, 1H), 2.59 (dd, *J* = 15.3, 6.4 Hz, 1H), 2.26 (ddt, *J* = 11.4, 7.5, 3.8 Hz, 2H), 1.68 – 1.60 (m, 2H), 1.05 (t, *J* = 7.5 Hz, 3H), 0.93 – 0.77 (m, 3H).

(*Z*)-isomer δ 5.81 (td, *J* = 7.5, 0.7 Hz, 1H), 3.73 (s, 3H), 3.66 (s, 3H), 3.51 – 3.40 (m, 1H), 2.54 (dd, *J* = 15.3, 7.9 Hz, 1H), 2.45 (dd, *J* = 15.2, 7.0 Hz, 1H), 2.40 (td, *J* = 10.2, 5.0 Hz, 2H), 1.56 (dqd, *J* = 13.2, 7.5, 5.7 Hz, 2H), 0.99 (t, *J* = 7.5 Hz, 3H), 0.90 (t, *J* = 7.5 Hz, 3H).

^{13}C NMR (151 MHz, C_6D_6) of (*E*):(*Z*) isomer mixture: (*E*)-isomer δ 173.6, 167.6, 147.0, 132.0, 51.5, 51.4, 38.6, 36.8, 26.3, 21.9, 13.5, 12.5.

(*Z*)-isomer δ 174.1, 168.6, 142.8, 133.1, 51.6, 51.6, 39.2, 35.2, 26.9, 25.0, 14.1, 11.0.

HRMS (ESI$^+$) m/z Calcd for $C_{12}H_{20}O_4Na$ 251.1254 $[M+Na]^+$; Found 251.1254 (+0.08 ppm).

ATR-IR 2954, 2876, 1731, 1434, 1150 cm^{-1}.

S4 General procedure for crotonate dimerisation experiments

S4.1 Using different light wavelengths to dimerise methyl crotonate

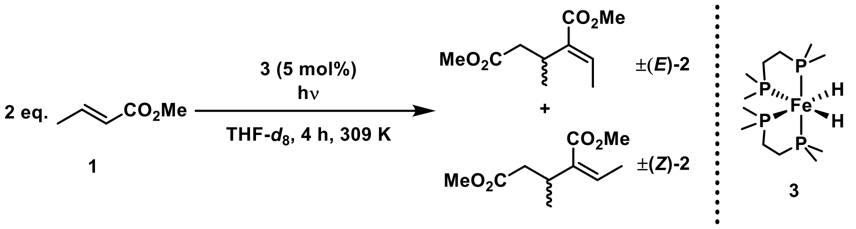

An NMR tube was charged with $[(dmpe)_2FeH_2]$ (7.0 mg, 0.020 mmol, 1 eq.) and *trans*-methyl crotonate (207 µL, 1.96 mmol, 100 eq.). This was immediately placed in front of a light source and irradiated for 4 hours, after which the tube was opened to air. 1,3,5-Trimethoxybenzene (0.1 mmol, 0.1 mL, 1 M in Et_2O) was added and the suspension filtered through silica to give a clear pale-yellow solution.

Spectroscopic data were in accordance with those previously reported.[14]

S4.2 *ex situ* General procedure for reaction monitoring

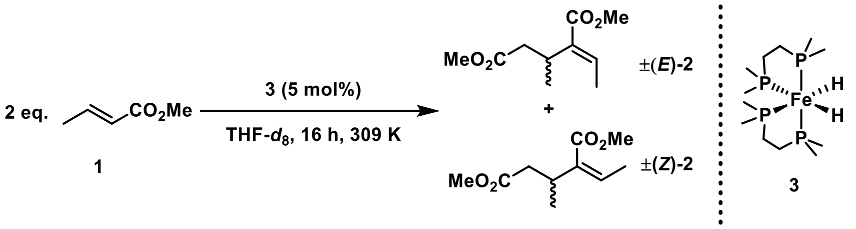

In a vial, $[(dmpe)_2FeH_2]$ (16.9 mg, 0.047 mmol, 5 eq.) was dissolved in THF-d_8 (0.4 mL) before *trans*-methyl crotonate – d_x (100 µL, 0.944 mmol, 100 eq.) was added. The mixture was transferred to an NMR tube and immediately placed in front of a light source and irradiated. For light experiments, the tube was irradiated and ^1H NMR spectra taken every 4 minutes for 1 hour. For non-light experiments, the NMR tube was placed in the spectrometer and ^1H NMR spectra taken every 5 minutes for 2 hours.

Spectroscopic data were in accordance with those previously reported.[14]

S4.3 General procedure for variable time normalised analysis (VTNA)

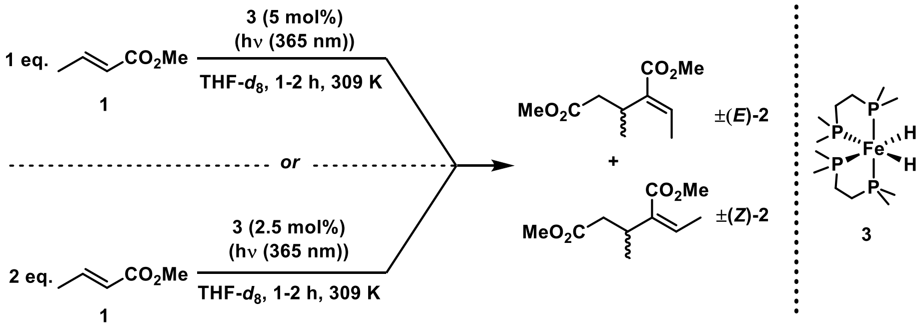

For half [(dmpe)$_2$FeH$_2$]: In a vial, [(dmpe)$_2$FeH$_2$] (8.45 mg, 0.0235 mmol, 2.5 eq.) was dissolved in THF-d_8 (0.4 mL) before *trans*-methyl crotonate (100 µL, 0.944 mmol, 100 eq.) was added. The mixture was transferred to an NMR tube and immediately placed in front of a light source and irradiated. For light experiments, the tube was irradiated and ^{1}H NMR spectra taken every 4 minutes for 1 hour. For non-light experiments, the NMR tube was placed in the spectrometer and ^{1}H NMR spectra taken every 5 minutes for 2 hours. Note that for VTNA experiments, only proteo-methyl crotonate was used.

For half methyl crotonate: In a vial, [(dmpe)$_2$FeH$_2$] (16.9 mg, 0.047 mmol, 5 eq.) was dissolved in THF-d_8 (0.4 mL) before *trans*-methyl crotonate (50 µL, 0.472 mmol, 50 eq.) was added. The mixture was transferred to an NMR tube and immediately placed in front of a light source and irradiated. For light experiments, the tube was irradiated and ^{1}H NMR spectra taken every 4 minutes for 1 hour. For non-light experiments, the NMR tube was placed in the spectrometer and ^{1}H NMR spectra taken every 5 minutes for 2 hours. Note that for VTNA experiments, only proteo-methyl crotonate was used.

Spectroscopic data were in accordance with those previously reported.[14]

S4.4 General procedure for crotyl substrate dimerisation

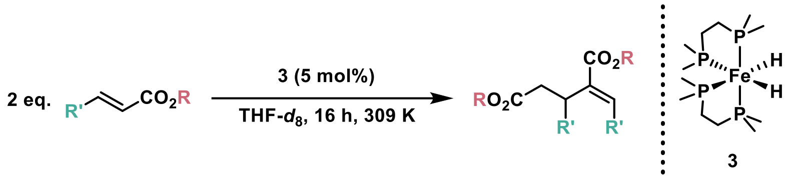

In a vial, [(dmpe)$_2$FeH$_2$] (16.9 mg, 0.0470 mmol, 5 eq.) was dissolved in THF-*d$_8$* (0.4 mL) before ethyl crotonate **9** (136 mg, 0.944 mmol, 100 eq.), methyl *(E)*-2-pentenoate **11** (136 mg, 0.944 mmol, 100 eq.) or methyl cinnamate **12** (153 mg, 0.944 mmol, 100 eq.) was added. The mixture was transferred to a *J*-Youngs NMR tube and immediately placed in the NMR spectrometer. The sample was then monitored in the dark using a periodically timed experiment whereby ^{1}H, ^{31}P{^{1}H} and ^{1}H-^{31}P HMBC spectra were recorded every 15 minutes continuously for 16 hours.

Spectroscopic data of (*E*), (*Z*)-diethyl 2-ethylidene-3-methylglutarate **10** and (*E*), (*Z*)-2-(1-propenyl)-3-ethylglutaric acid dimethyl ester **13** were in accordance with **S3.8** and **S3.9**.

S4.5 General procedure for *in situ* monitoring

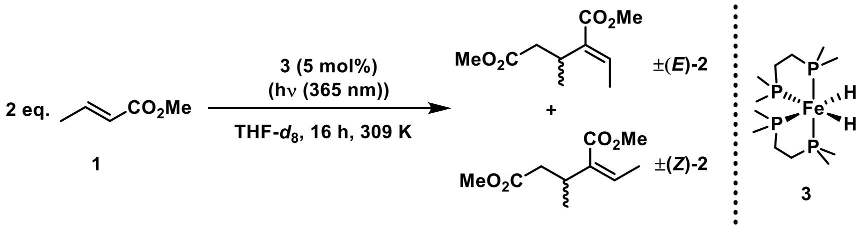

In a vial, [(dmpe)$_2$FeH$_2$] (8.4 mg, 0.024 mmol, 5 eq.) was dissolved in THF-*d$_8$* (0.2 mL) before *trans*-methyl crotonate (50 µL, 0.47 mmol, 100 eq.). The mixture was then transferred to a *J*-Youngs tube. The LED-NMR setup was designed by Gschwind[15] and set up as described by Ben-Tal and Lloyd-Jones,[3] with 365 nm LED and the assembled NMR tube was placed inside the NMR spectrometer. The spectrometer was tuned to both ^{1}H and ^{31}P nuclei and shimmed prior to irradiation. The data was recorded as interleaved ^{31}P{^{1}H} and ^{1}H spectra. For the light-on experiment, irradiation was started at the measurement of the first spectrum and remained constant throughout the experiment. The dark experiment was assembled as with the light-on experiment, but no irradiation was carried out. Irradiation was turned on/off for the light/dark experiment. The spectra were phased and a baseline corrected and each peak integrated. A moving average of size 8 was applied during processing and a total reaction spectrum was generated for both the light-on and dark experiments.[2]

Spectroscopic data were in accordance with those previously reported.[14]

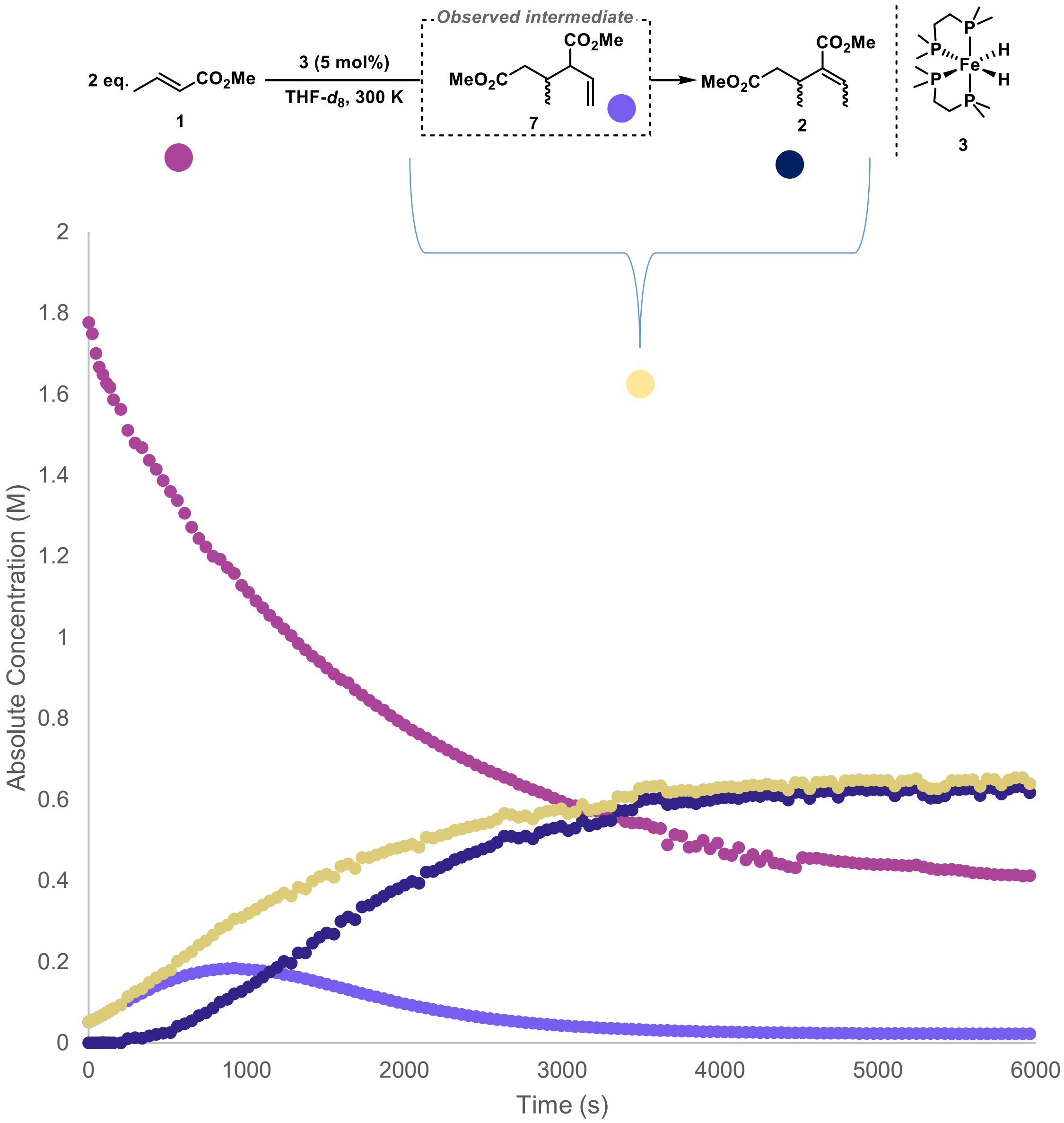

Supplementary Figure 4 – *in situ* Monitoring under light conditions for 6000 seconds showing conversion of methyl crotonate **1** (pink) to (*E*),(*Z*)-dimethyl 2-ethylidene-3-methylpentanedioate **2** (blue) *via* the intermediate dimethyl 2-ethenyl-3-methylpentanedioate **7** (purple). The addition of the absolute concentrations of **7** + **2** (yellow) shows that the initial observed induction period seen in the formation of (*E*),(*Z*)-dimethyl 2-ethylidene-3-methylpentanedioate **2** is explained by a build-up in dimethyl 2-ethenyl-3-methylpentanedioate **7**.

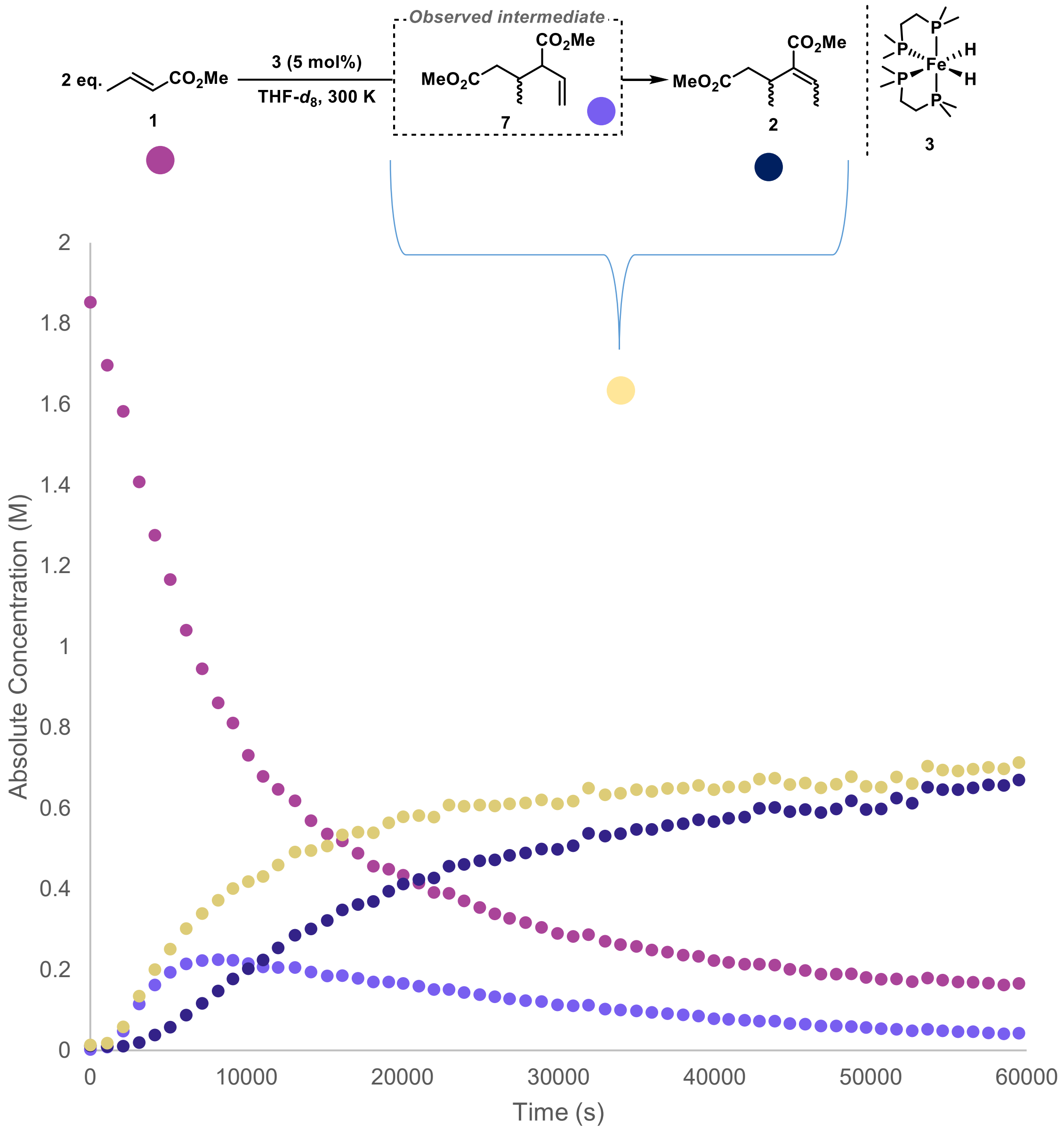

Supplementary Figure 5 – *in situ* Monitoring under no-light conditions for 60000 seconds showing conversion of methyl crotonate **1** (pink) to (*E*),(*Z*)-dimethyl 2-ethylidene-3-methylpentanedioate **2** (blue) *via* the intermediate dimethyl 2-ethenyl-3-methylpentanedioate **7** (purple). The addition of the absolute concentrations of **7** + **2** (yellow) shows that the initial observed induction period seen in the formation of (*E*),(*Z*)-dimethyl 2-ethylidene-3-methylpentanedioate **2** is explained by a build-up in dimethyl 2-ethenyl-3-methylpentanedioate **7**.

S4.6 General procedure for 16-hour no-light dimerisation experiments

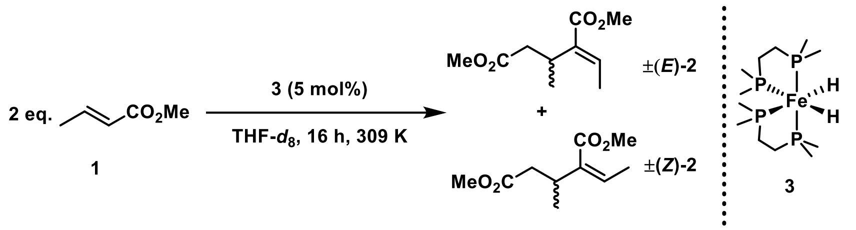

In a vial, [(dmpe)$_2$FeH$_2$] (16.9 mg, 0.0470 mmol, 5 eq.) was dissolved in THF-*d$_8$* (0.4 mL) before methyl crotonate (100 µL, 0.944 mmol, 100 eq.) was added. The mixture was transferred to a *J*-Youngs NMR tube and immediately placed in the NMR spectrometer. The sample was then monitored in the dark using a periodically timed experiment whereby ^{1}H, ^{31}P{^{1}H} and ^{1}H-^{31}P HMBC spectra were recorded every 15 minutes continuously for 16 hours.

Spectroscopic data were in accordance with those previously reported.[14]

S4.7 General procedure for control experiments using methyl 3-butanoate 19

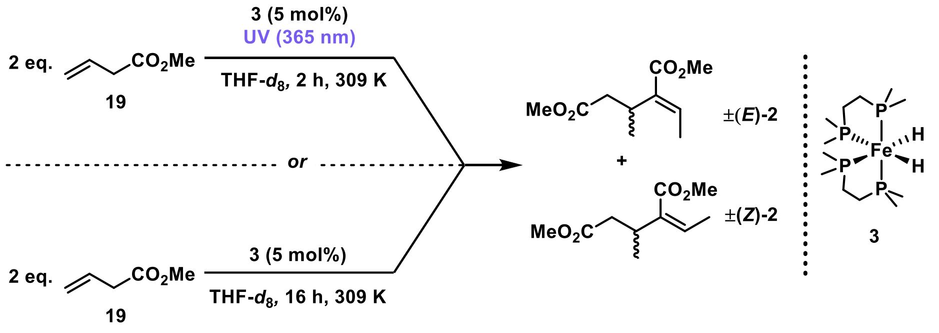

In a vial, [(dmpe)$_2$FeH$_2$] (16.9 mg, 0.0470 mmol, 5 eq.) was dissolved in THF-*d$_8$* (0.4 mL) before methyl-3-butanoate (100 µL, 0.944 mmol, 100 eq.) was added.

UV: The mixture was transferred to a *J*-Youngs NMR tube and irradiated for 2 hours.

No UV: The mixture was transferred to a *J*-Youngs NMR tube and immediately placed in the NMR spectrometer. The sample was then monitored in the dark by ^{1}H, ^{31}P{^{1}H} and ^{1}H-^{31}P HMBC spectra were recorded across 16 hours.

Spectroscopic data were in accordance with those previously reported.[14]

S5 Iron species identified throughout *in situ* monitoring

S5.1 [(dmpe)$_2$Fe(PhCH=CHCO$_2$Me)] 15

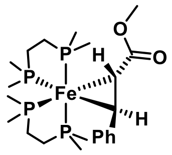

In a vial, [(dmpe)$_2$FeH$_2$] (16.9 mg, 0.0470 mmol, 5 eq.) was dissolved in THF-d_8 (0.4 mL) before methyl cinnamate **12** (153 mg, 0.944 mmol, 100 eq.) was added. The mixture was transferred to a *J*-Youngs NMR tube and irradiated for 2 hours. Orange block-shaped crystals suitable for X-ray diffraction were grown from a concentrated pentane solution at −35 °C.

^{1}H NMR (500 MHz, THF-d_8) δ 7.07 (d, *J* = 7.6 Hz, 2H), 6.87 (t, *J* = 7.5 Hz, 2H), 6.69 (t, *J* = 7.2 Hz, 1H), 3.42 (s, 3H), 3.40 – 3.35 (m, 1H), 2.23 (q, *J* = 9.1 Hz, 1 H), 1.61 (d, *J* = 6.8 Hz, 4H), 1.53 – 1.51 (m, 4H), 1.49 (d, *J* = 6.6 Hz, 4H), 1.22 (d, *J* = 5.2 Hz, 4H), 1.19 – 1.15 (m, 6H), 1.09 (d, *J* = 5.6 Hz, 4H), 0.89 (t, *J* = 7.0 Hz, 3H), 0.49 (d, *J* = 3.5 Hz, 3H).

^{31}P{^{1}H} NMR (202 MHz, THF-d_8) δ 62.7 – 61.7 (m), 53.1 (td, *J* = 36.9, 5.6 Hz), 51.3 (td, *J* = 44.4, 5.6 Hz).

^{13}C NMR (126 MHz, THF-d_8) δ 182.5, 156.0, 126.7, 119.9, 39.7, 39.5, 35.0, 34.1, 33.6, 32.7, 31.0, 25.4, 24.2, 23.3, 22.1, 18.6, 18.2, 14.4, 13.5.

S5.2 *trans*-[(dmpe)$_2$FeH(CH$_3$CH=CH-COO$^-$)] 16

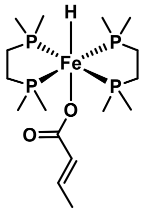

^{1}H NMR (500 MHz, C$_6$D$_6$) δ 6.56 (dd, *J* = 14.9, 7.2 Hz, 1H), 5.99 (d, *J* = 15.2 Hz, 1H), 2.23 – 2.14 (m, 4H), 1.56 – 1.48 (m, 4H), 1.38 (s, 12H), 1.09 (s, 12H). *CH$_3$ not observed.*

^{31}P NMR (202 MHz, C$_6$D$_6$) δ 72.1 (d, *J* = 37.2 Hz).

^{13}C NMR (126 MHz, C$_6$D$_6$) δ 137.7, 31.5, 22.6, 14.8. *Not all ^{13}C NMR resonances identified.*

S5.3 *trans*-[$(dmpe)_2FeH(CH_3O_2CC_6H_{10}COO^-)$] 17

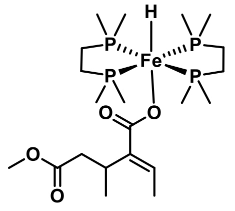

^{1}H NMR (500 MHz, THF-*d*$_8$) δ 5.89 (q, *J* = 7.2 Hz, 1H), 3.47 (s, 3H), 2.95 (h, *J* = 7.0 Hz, 1H), 2.57 (t, *J* = 6.9 Hz, 2H), 2.28 – 2.20 (m, 4H), 1.72 – 1.65 (m, 4H), 1.54 (d, *J* = 7.1 Hz, 3H), 1.32 (q, *J* = 3.5 Hz, 12H), 1.27 (s, 12H), 1.02 (d, *J* = 6.9 Hz, 3H), −33.69 (p, *J* = 49.0 Hz, 1H).

^{31}P{^{1}H} NMR (202 MHz, THF-*d*$_8$) δ 72.5 – 72.1 (m).

^{13}C NMR (126 MHz, THF-*d*$_8$) δ 173.1, 171.7, 142.8, 125.9, 39.7, 31.5, 29.6, 22.3, 18.8, 14.6, 12.5. *P**C**H$_2$ carbons not observed.*

S5.4 [$\{(dmpe)_2FeH\}_2(\mu\text{-}dmpe)_2]^{2+}$ 18

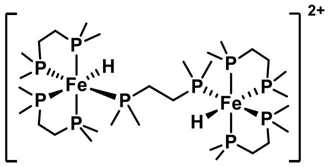

^{1}H NMR (500 MHz, THF-*d*$_8$) (*hydride region only*) δ −17.7 (pd, *J* = 55.6, 18.2 Hz).

^{31}P{^{1}H} NMR (202 MHz, THF-*d*$_8$) δ 67.1 (dd, *J* = 44.7, 31.6 Hz), 13.9 (dt, *J* = 38.5, 9.0 Hz).

Spectroscopic data were in accordance with those previously reported.[16]

S5.5 *trans*-[$(dmpe)_2FeH(CH_2CH{=}CHCO_2Me)$] 21

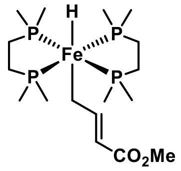

In a vial, [$(dmpe)_2FeH_2$] (16.9 mg, 0.0470 mmol, 5 eq.) was dissolved in THF-*d*$_8$ (0.4 mL) before methyl 3-butanoate (100 µL, 0.944 mmol, 100 eq.) was added. The mixture was transferred to a *J*-Youngs NMR tube and analysed periodically across 16 hours. The sample was transferred to a vial and placed in a glovebox freezer at –35 °C overnight to yield an orange suspension which was filtered and washed with pentane (2 x 1 mL). *trans*-[$(dmpe)_2FeH(CH_2CH{=}CHCO_2Me)$] **21** (2.0 mg, 0.00438 mmol, 9%) was obtained as an orange amorphous powder.

^{1}H NMR (500 MHz, THF-*d*$_8$) δ 7.03 (dt, *J* = 14.1, 10.6 Hz, 1H), 4.57 (d, *J* = 14.1 Hz, 1H), 3.41 (s, 3H), 1.87 – 1.82 (m, 4H), 1.60 (t, *J* = 7.5 Hz, 4H), 1.41 (s, 12H), 1.26 (s, 12H), 0.55 (dp, *J* = 10.2, 5.1 Hz, 2H), -24.85 (p, *J* = 50.0 Hz, 1H).

^{31}P{^{1}H} NMR (202 MHz, THF-*d*$_8$) δ 71.7 (d, *J* = 10.6 Hz).

^{13}**C NMR (126 MHz, THF-*d*$_8$)** δ 168.1, 164.9, 92.4, 48.0, 31.0, 25.9, 24.4, 14.6.

S5.6 [(dmpe)$_2$Fe(η^2-1)] 22

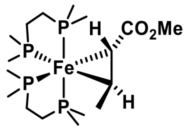

^{1}**H NMR (500 MHz, THF-*d*$_8$)** δ 3.39 (s), 1.71 (d, *J* = 6.7 Hz), 1.62 (m), 1.28 (d, *J* = 6.4 Hz), 1.21 (s), 1.12 (d, *J* = 6.9 Hz), 1.10 (d, *J* = 5.9 Hz). *Not all ^{1}H NMR resonances observed.*

^{31}**P{^{1}H} NMR (202 MHz, THF-*d*$_8$)** δ 63.4 (dtd, *J* = 62.8, 40.6, 31.1 Hz), 62.4 (ddd, *J* = 115.4, 54.0, 29.6 Hz), 59.5 (ddd, *J* = 54.2, 43.7, 6.6 Hz), 50.0 (ddd, *J* = 44.0, 29.4, 6.8 Hz).

^{13}**C NMR (126 MHz, THF-*d*$_8$)** δ 182.2, 34.3, 31.5, 30.8, 25.9, 24.9, 22.6, 22.1, 18.4, 16.9. *Not all ^{13}C NMR resonances observed.*

S5.7 *trans*-[(dmpe)$_2$FeHY] 23

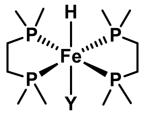

^{1}**H NMR (500 MHz, THF-*d*$_8$) (*hydride region only*)** δ 1.82*, 1.48*, 1.29*, −13.1 (p, *J* = 59.0 Hz, 1H).

*^{1}H NMR resonances assigned by ^{1}H−^{31}P HMBC NMR spectroscopy but overlap with other signals therefore the splitting and integration could not be determined.

^{31}**P{^{1}H} NMR (202 MHz, THF-*d*$_8$)** δ 69.2 (d, *J* = 12.0 Hz)

S5.8 [(dmpe)$_5$Fe$_2$] 26

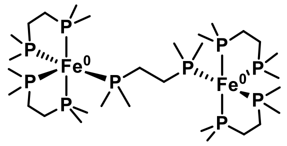

^{31}**P NMR (202 MHz, THF-*d*$_8$)** δ 60.8 (d, *J* = 10.7 Hz), 7.63 (p, *J* = 9.5 Hz).

Spectroscopic data were in accordance with those previously reported.[17, 18]

S5.9 [(dmpe)$_3$Fe] 27

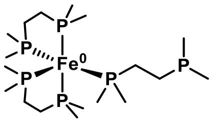

^{31}**P{^{1}H} NMR (202 MHz, THF-*d*$_8$)** δ 60.9 (d, *J* = 10.4 Hz), 10.5 (dq, *J* = 19.5, 10.3 Hz), −48.9 (br s).

Spectroscopic data were in accordance with those previously reported.[17]

S6 Determining whether species observed *in situ* are catalytically active

S6.1 [(dmpe)$_2$Fe(PhCH=CHCO$_2$Me)] 15

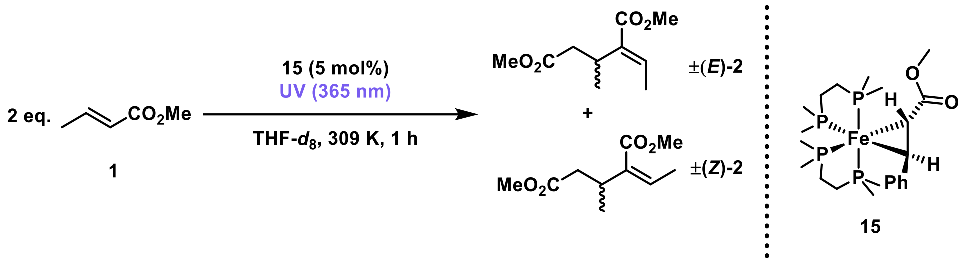

In an NMR tube, [(dmpe)$_2$Fe(PhCH=CHCO$_2$Me)] **15** (2.4 mg, 0.0046 mmol, 5 eq.) was dissolved in THF-d_8 (0.3 mL) and methyl crotonate **1** (9.27 mg, 0.093 mmol, 100 eq.) was added. The mixture was then exposed to UV (365 nm) and monitored *in situ* (using the general procedure described in **S4.5**) by ^{1}H and ^{31}P{^{1}H} NMR spectroscopy for 1 hour (see **Supplementary Figure 76** for more details).

S6.2 *trans*-[(dmpe)$_2$FeH(CH$_3$O$_2$CC$_6$H$_{10}$COO$^-$)] 17

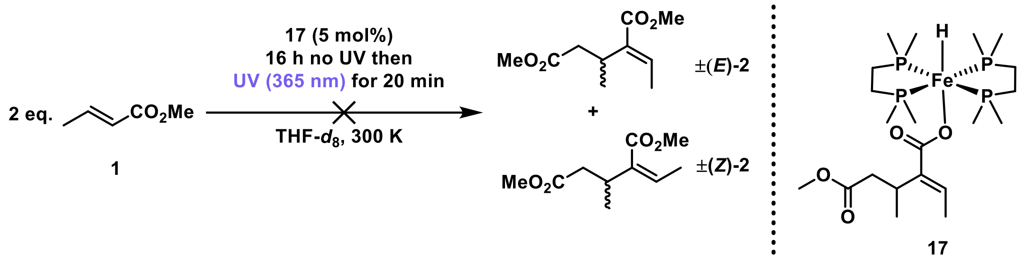

In an NMR tube, *trans*-[(dmpe)$_2$FeH(CH$_3$O$_2$CC$_6$H$_{10}$COO$^-$)] **17** (2.0 mg, 0.00369 mmol, 5 eq.) was dissolved in THF-d_8 (0.3 mL) and methyl crotonate **1** (7.38 mg, 0.0738 mmol, 100 eq.) was added. The mixture was then monitored *in situ* (see General Procedure in **S4.6** for more details) by ^{1}H and ^{31}P{^{1}H} NMR spectroscopy for 16 hours, then exposed to UV (365 nm) for 20 minutes and analysed by ^{1}H and ^{31}P{^{1}H} NMR spectroscopy (see **Supplementary Figures 85-86** for more details).

S6.3 *trans*-[(dmpe)$_2$FeH(CH$_2$CH=CHCO$_2$Me)] 21

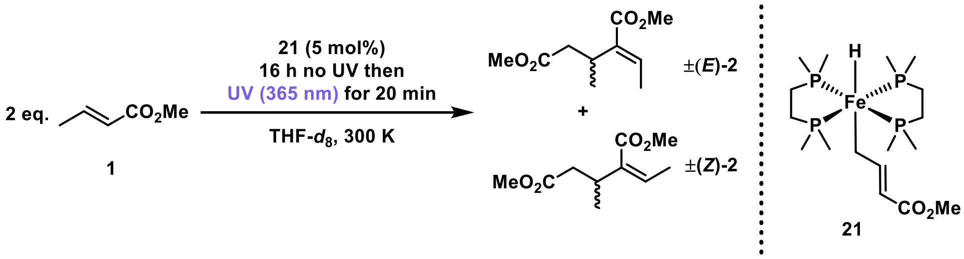

In an NMR tube, *trans*-[(dmpe)$_2$FeH(CH$_2$CH=CHCO$_2$Me)] **21** (2.0 mg, 0.00438 mmol, 5 eq.) was dissolved in THF-d_8 (0.3 mL) and methyl crotonate **1** (8.78 mg, 0.0877 mmol, 100 eq.) was added. The mixture was then monitored *in situ* (see General Procedure in **S4.6** for more details) by ^{1}H and ^{31}P{^{1}H} NMR spectroscopy for 16 hours, then exposed to UV (365 nm) for 20 minutes

and analysed by ^{1}H and $^{31}P\{^{1}H\}$ NMR spectroscopy (see **Supplementary Figure 90** for more details).

S6.4 [(dmpe)$_2$Fe(η^2-1)] 22 (formed *in situ*)

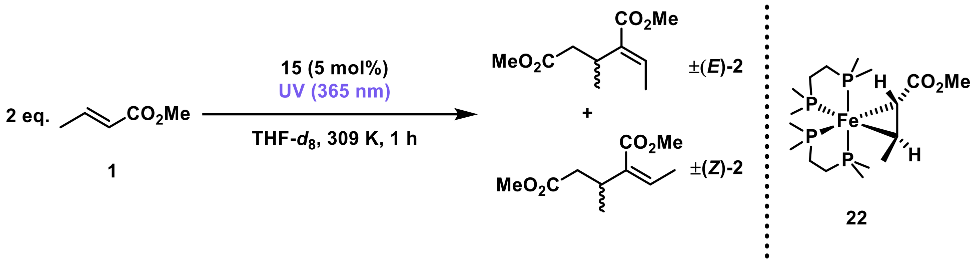

In an NMR tube, [(dmpe)$_2$FeH$_2$] **3** (16.9 mg, 0.047 mmol, 10 eq.) was dissolved in THF-*d*$_8$ (0.3 mL) and methyl crotonate **1** (47.2 mg, 0.235 mmol, 100 eq.) was added. The mixture was then exposed to UV (365 nm) for 10 minutes before the UV source was switched off and the sample monitored *in situ* under dark conditions for an additional 10 minutes (see **S4.5** for more details) by ^{1}H and $^{31}P\{^{1}H\}$ NMR spectroscopy. The sample was removed from the NMR spectrometer and an additional amount of methyl crotonate **1** (47.2 mg, 0.235 mmol, 100 eq.) was added. The sample was then left for 10 minutes under dark conditions before being exposed to UV (365 nm) for a further 30 minutes (see **Supplementary Figures 92-93** for more details).

S6.5 *trans*-[(dmpe)$_2$Fe(CH$_3$CH=CH-COO$^-$)$_2$] 25

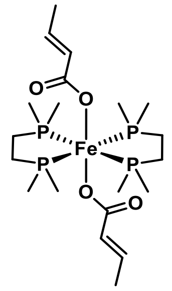

In a vial, [(dmpe)$_2$FeH$_2$] **3** (16.9 mg, 0.0470 mmol, 1 eq.) was dissolved in THF (0.5 mL) before crotonic acid (8.1 mg, 0.094 mmol, 2 eq.) was added. The mixture instantly changed colour from pale yellow to a dark red. Red block-shaped crystals of [(dmpe)$_2$Fe(CH$_3$CH=CH-COO$^-$)$_2$] **25** were grown from pentane at −35 °C however the molecular structure could not be determined by X-ray diffraction due to dmpe ligand disorder.

^{1}H NMR (500 MHz, THF-*d*$_8$) δ 5.65 (dq, J = 13.6, 6.8 Hz, 2H), 4.91 (dd, J = 15.3, 1.9 Hz, 2H), 2.53 (dq, J = 6.9, 4.0 Hz, 8H), 1.43 (dd, J = 6.8, 1.7 Hz, 6H), 1.29 (s, 24H).

$^{31}P\{^{1}H\}$ NMR (202 MHz, THF-*d*$_8$) δ 64.2 (s).

^{13}C NMR (126 MHz, THF-*d*$_8$) δ 173.3, 131.6, 128.5, 29.8 (t, J = 12.6 Hz), 16.0, 13.4 (p, J = 5.2 Hz).

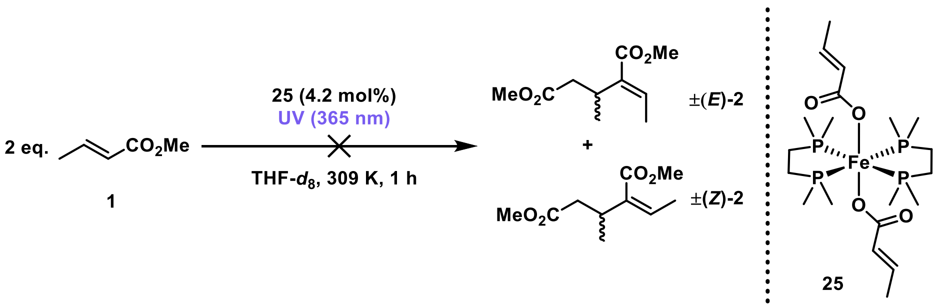

In an NMR tube, [(dmpe)$_2$Fe(CH$_3$CH=CH-COO$^-$)$_2$] **25** (1.6 mg, 0.00304 mmol, 1 eq.) was dissolved in THF-d_8 (0.3 mL) and methyl crotonate **1** (7.24 mg, 0.072 mmol, 24 eq.) was added. The mixture was then exposed to UV (365 nm) and monitored *in situ* (see **S4.5** for more details) by ^{1}H and ^{31}P{^{1}H} NMR spectroscopy for 1 hour (see **Supplementary Figures 78-81** for more details).

S7 Crystallographic data

S7.1 [(dmpe)$_2$Fe(PhCH=CHCO$_2$Me)] 15

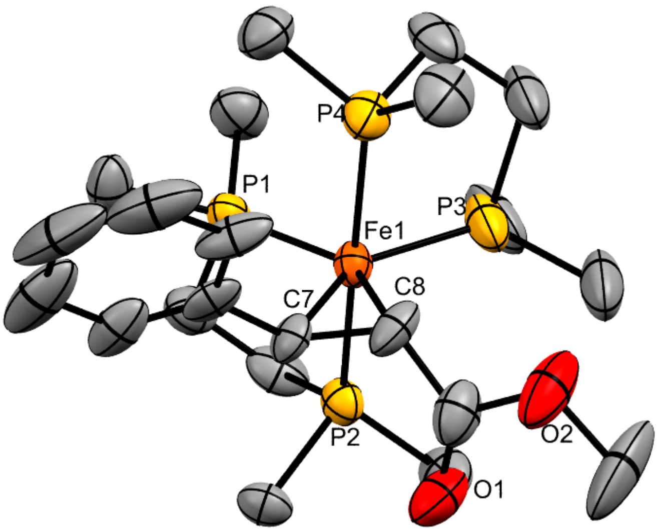

Formula	$C_{22}H_{42}O_2P_4Fe$
$D_{calc.}$/ g cm^{-3}	1.315
μ/mm^{-1}	7.051
Formula Weight	518.28
Colour	translucent dark orange
Shape	plate-shaped
Size/mm^3	0.08×0.05×0.02
T/K	100.00(10)
Crystal System	monoclinic
Space Group	$P2_1/c$
a/Å	9.1635(6)
b/Å	15.4314(11)
c/Å	18.7645(13)
α/°	90
β/°	99.482(6)
γ/°	90
V/Å^3	2617.1(3)
Z	4
Z'	1
Wavelength/Å	1.54184
Radiation type	Cu K_α
Θ_{min}/°	3.729
Θ_{max}/°	50.433
Measured Refl's.	19769
Indep't Refl's	2737
Refl's I≥2 σ(I)	2446
R_{int}	0.0929
Parameters	271
Restraints	0
Largest Peak	0.412
Deepest Hole	-0.507
GooF	1.145
wR_2 (all data)	0.2024
wR_2	0.1989
R_1 (all data)	0.0822
R_1	0.0762

Experimental. Single translucent dark orange plate-shaped crystals were recrystallised from a mixture of THF-d_8 and pentane at −35 °C by slow evaporation. A suitable crystal with dimensions 0.08 × 0.05 × 0.02 mm^3 was selected and mounted on a MITIGEN holder in Paratone oil on a XtaLAB Synergy R, HyPix-Arc 100 diffractometer. The crystal was kept at a steady T = 100.00(10) K during data collection. The structure was solved with the ShelXS (Sheldrick, 2008) solution program using heavy methods and by using Olex2 1.5-beta (Dolomanov et al., 2009) as the graphical interface. The model was refined with ShelXL 2018/3 (Sheldrick, 2015) using full matrix least squares minimisation on $\boldsymbol{F^2}$.

Crystal Data. $C_{22}H_{42}O_2P_4Fe$, M_r = 518.28, monoclinic, $P2_1/c$ (No. 14), a = 9.1635(6) Å, b = 15.4314(11) Å, c = 18.7645(13) Å, β = 99.482(6)°, α = γ = 90°, V = 2617.1(3) Å^3, T = 100.00(10) K, Z = 4, Z' = 1, μ(Cu K_α) = 7.051, 19769 reflections measured, 2737 unique (R_{int} = 0.0929) which were used in all calculations. The final wR_2 was 0.2024 (all data) and R_1 was 0.0762 (I≥2 σ(I)).

_refine_special_details: The data set resolution was cut at 1 Å during final refinement due to large values of R(merge) at high angle. A disorder model was attempted for the benzyl ring.

S7.2 *trans*-[(dmpe)$_2$FeH($CH_3CH=CH-COO^-$)] **16**

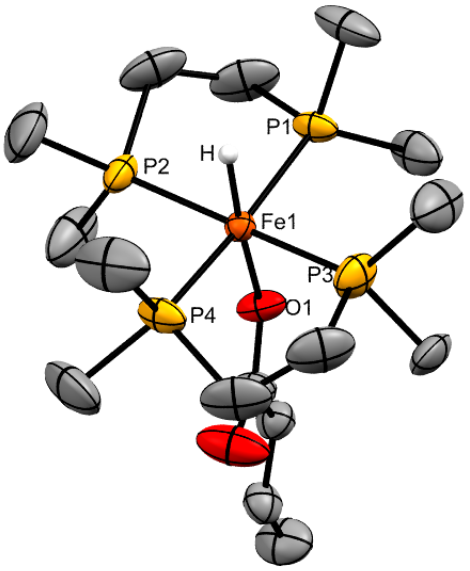

Experimental. Single orange block-shaped crystals were recrystallised at −35 °C from a solution of pentane by slow evaporation. A suitable crystal with dimensions 0.47 × 0.29 × 0.28 mm^3 was selected and mounted on a mitegen tip in Paratone oil. on a Bruker D8 Venture diffractometer. The crystal was kept at a steady *T* = 100.00 K during data collection. The structure was solved with the ShelXT 2018/2 (Sheldrick, 2018) solution program using dual methods and by using Olex2 1.5-beta (Dolomanov et al., 2009) as the graphical interface. The model was refined with ShelXL 2019/3 (Sheldrick, 2015) using full matrix least squares minimisation on $\boldsymbol{F}^2$.

Formula	$C_{16}H_{38}FeO_2P_4$
$D_{calc.}$/ g cm^{-3}	1.294
μ/mm^{-1}	0.952
Formula Weight	442.19
Colour	orange
Shape	block-shaped
Size/mm^3	0.47×0.29×0.28
T/K	100.00
Crystal System	monoclinic
Space Group	$P2_1/c$
a/Å	14.5261(4)
b/Å	8.9654(3)
c/Å	17.4240(5)
α/°	90
β/°	90.007(2)
γ/°	90
V/Å^3	2269.17(12)
Z	4
Z'	1
Wavelength/Å	0.71073
Radiation type	MoK_α
Θ_{min}/°	2.272
Θ_{max}/°	28.298
Measured Refl's.	133142
Indep't Refl's	5639
Refl's I≥2 σ(I)	5395
R_{int}	0.0495
Parameters	403
Restraints	956
Largest Peak	0.614
Deepest Hole	-0.453
GooF	1.093
wR_2 (all data)	0.0974
wR_2	0.0949
R_1 (all data)	0.0384
R_1	0.0359

Crystal Data. $C_{16}H_{38}FeO_2P_4$, M_r = 442.19, monoclinic, $P2_1/c$ (No. 14), a = 14.5261(4) Å, b = 8.9654(3) Å, c = 17.4240(5) Å, β= 90.007(2)°, α= γ = 90°, *V* = 2269.17(12) Å^3, *T* = 100.00 K, *Z* = 4, *Z'* = 1, $\mu(MoK_a)$ = 0.952, 133142 reflections measured, 5639 unique (R_{int} = 0.0495) which were used in all calculations. The final wR_2 was 0.0974 (all data) and R_1 was 0.0359 (I≥2 *s*(I)).

_refine_special_details: The clean diffraction pattern has apparent orthorhombic metric symmetry. However, systematic absence exceptions for 2(1) screw axes do not unambiguously fit any common orthorhombic space group in the sense that the third, necessary twofold axis is not clear. Solution in with ShelXT, with the -y -a switches set to allow the software to explore the full range of orthorhombic space groups, does not yield a clean solution (space group P2(1)2(1)2(1) is chosen, but the structure is not stable). With this in mind, the crystal system symmetry was reduced and the data set re-integrated. The beta angle refines to 90.007(2) degrees. Space group determination is quite clear and the initial ShelXT solution identifies the iron centre and the phosphorus atoms. Successive difference maps established that the all ligands (apart from hydride) are disordered over two positions. Refinement of the occupancies with different free variable established that the equatorial phosphine ligand disorder ratio (approx 3:1) is different from the axial ligand disorder ratio (approx 1:1). Only the iron centre and the hydride are not disordered. Twin law -1 0 0 / 0 -1 0 / 0 0 1 and refined twin scale factor 0.4632(13) handles the pseudo-orthorhombic metric symmetry. Geometric and displacement ellipsoid restraints were applied globally.

S7.3 *trans*-[(dmpe)$_2$FeH($CH_3O_2CC_6H_{10}COO^-$)] 17

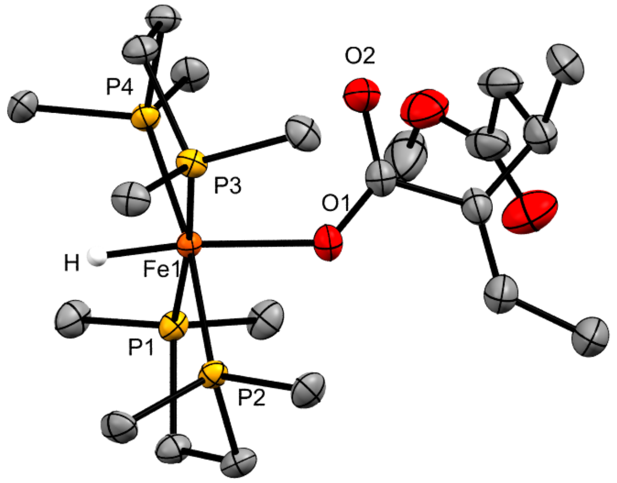

Experimental. Single clear yellow block-shaped crystals were grown at −35 °C from a concentrated solution of pentane and THF-d_8. A suitable crystal with dimensions 0.18 × 0.05 × 0.02 mm^3 was selected and mounted on a XtaLAB Synergy, Single source at home/near, HyPix-Arc 100 diffractometer. The crystal was kept at a steady T = 100.01(10) K during data collection. The structure was solved with the ShelXT 2018/2 (Sheldrick, 2018) solution program using dual methods and by using Olex2 1.5-beta (Dolomanov et al., 2009) as the graphical interface. The model was refined with ShelXL 2018/3 (Sheldrick, 2015) using full matrix least squares minimisation on $\boldsymbol{F}^2$.

Crystal Data. $C_{21}H_{46}FeO_4P_4$, M_r = 542.31, monoclinic, $P2_1/n$ (No. 14), a = 9.18470(10) Å, b = 20.6020(3) Å, c = 14.6513(3) Å, β = 92.907(2)°, $\alpha = \gamma$ = 90°, V = 2768.80(8) Å^3, T = 100.01(10) K, Z = 4, Z' = 1, μ(Mo K$_\alpha$) = 0.799, 64221 reflections measured, 7806 unique (R_{int} = 0.0509) which were used in all calculations. The final wR_2 was 0.1032 (all data) and R_1 was 0.0467 (I≥2 σ(I)).

Formula	$C_{21}H_{46}FeO_4P_4$
$D_{calc.}$/ g cm^{-3}	1.301
μ/mm^{-1}	0.799
Formula Weight	542.31
Colour	clear yellow
Shape	block-shaped
Size/mm^3	0.18×0.05×0.02
T/K	100.01(10)
Crystal System	monoclinic
Space Group	$P2_1/n$
a/Å	9.18470(10)
b/Å	20.6020(3)
c/Å	14.6513(3)
α/°	90
β/°	92.907(2)
γ/°	90
V/Å^3	2768.80(8)
Z	4
Z'	1
Wavelength/Å	0.71073
Radiation type	Mo K$_\alpha$
Θ_{min}/°	1.977
Θ_{max}/°	30.648
Measured Refl's.	64221
Indep't Refl's	7806
Refl's I≥2 σ(I)	6225
R_{int}	0.0509
Parameters	327
Restraints	12
Largest Peak	0.529
Deepest Hole	-0.474
GooF	1.141
wR_2 (all data)	0.1032
wR_2	0.0975
R_1 (all data)	0.0664
R_1	0.0467

_refine_special_details: There is disorder about C15 interpreted as the presence of two diasteroisomers. This was handled with PART -1/-2, geometric and displacement ellipsoid similarity restraints.

S7.4 [(dmpe)$_2$Fe(η^2-1)] 22

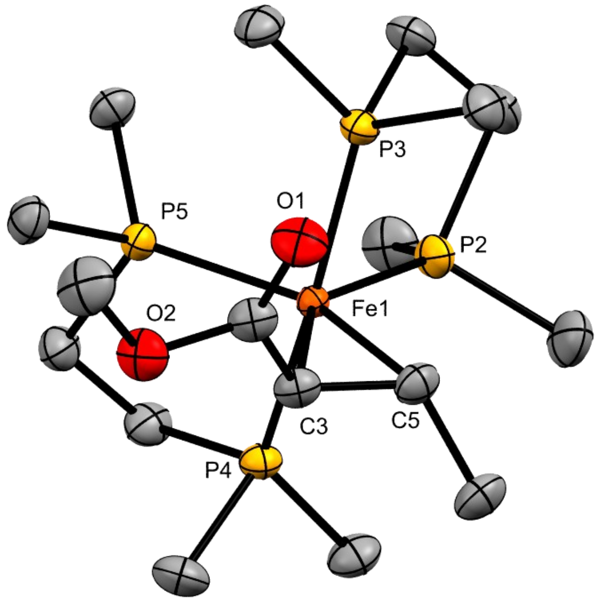

Formula	$C_{17}H_{40}FeO_2P_4$
$D_{calc.}$/ g cm^{-3}	1.353
μ/mm^{-1}	8.160
Formula Weight	456.246
Colour	translucent pale orange
Shape	block-shaped
Size/mm^3	0.12×0.09×0.05
T/K	120.00(10)
Crystal System	orthorhombic
Space Group	*Pbca*
a/Å	16.86988(18)
b/Å	15.39782(16)
c/Å	17.24096(19)
α/°	90
β/°	90
γ/°	90
V/Å^3	4478.50(8)
Z	8
Z'	1
Wavelength/Å	1.54184
Radiation type	Cu K$_\alpha$
Θ_{min}/°	4.66
Θ_{max}/°	76.12
Measured Refl's.	67317
Indep't Refl's	4655
Refl's I≥2 σ(I)	4156
R_{int}	0.0638
Parameters	577
Restraints	202
Largest Peak	0.8780
Deepest Hole	-0.2885
GooF	1.0802
wR_2 (all data)	0.0749
wR_2	0.0713
R_1 (all data)	0.0331
R_1	0.0280

Experimental. Single translucent pale orange block-shaped crystals were recrystallised from pentane at −35 °C by slow evaporation. A suitable crystal with dimensions 0.12 × 0.09 × 0.05 mm^3 was selected and mounted on a MITIGEN holder in Paratone oil. on a Rigaku Oxford Diffraction SuperNova diffractometer. The crystal was kept at a steady T = 120.00(10) K during data collection. The structure was solved with the ShelXS (Sheldrick, 2008) solution program using direct methods and by using Olex2 1.5-beta (Dolomanov et al., 2009) as the graphical interface. The model was refined with olex2.refine 1.5-beta (Bourhis et al., 2015) using full matrix least squares minimisation on F^2.

Crystal Data. $C_{17}H_{40}FeO_2P_4$, M_r = 456.246, orthorhombic, *Pbca* (No. 61), a = 16.86988(18) Å, b = 15.39782(16) Å, c = 17.24096(19) Å, $\alpha = \beta = \gamma = 90°$, V = 4478.50(8) Å^3, T = 120.00(10) K, Z = 8, Z' = 1, μ(Cu K$_a$) = 8.160, 67317 reflections measured, 4655 unique (R_{int} = 0.0638) which were used in all calculations. The final wR_2 was 0.0749 (all data) and R_1 was 0.0280 (I≥2 σ(I)).

_olex2_refine_details: Refinement using NoSpherA2, an implementation ofNOn-SPHERical Atom-form-factors in Olex2.Please cite:F. Kleemiss et al. Chem. Sci. DOI 10.1039/D0SC05526C - 2021NoSpherA2 implementation of HAR makes use oftailor-made aspherical atomic form factors calculatedon-the-fly from a Hirshfeld-partitioned electron density (ED) - not fromspherical-atom form factors.The ED is calculated from a gaussian basis set single determinant SCFwavefunction - either Hartree-Fock or DFT using selected funtionals - for a fragment of the crystal.This fragment can be embedded in an electrostatic crystal field by employing cluster chargesor modelled using implicit solvation models, depending on the software used.The following options were used: SOFTWARE: ORCA 5.0 PARTITIONING: NoSpherA2 INT ACCURACY: Normal METHOD: R2SCAN BASIS SET: def2-TZVP CHARGE: 0 MULTIPLICITY: 1 DATE: 2024-09-13_16-22-09

S7.5 [(dmpe)$_5$Fe$_2$] 26

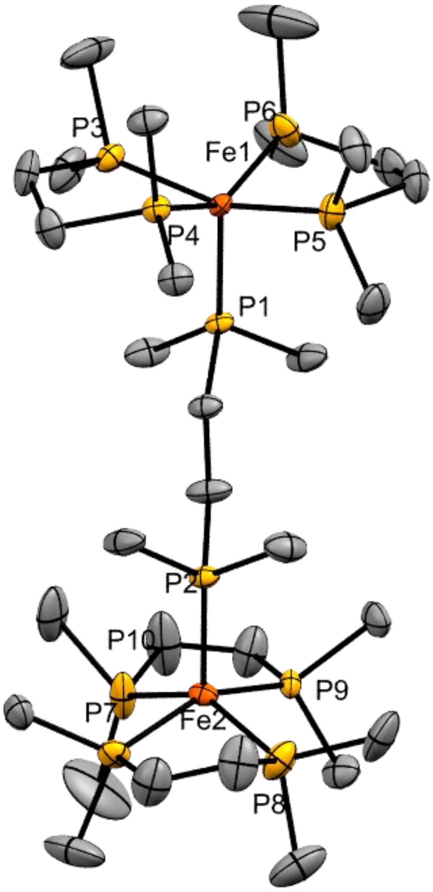

Experimental. Single translucent pale orange block-shaped crystals were recrystallised from THF-d_8 by spontaneous crystallization from 365 nm photoirradiation at room temperature over 24 hours. A suitable crystal with dimensions 0.10 × 0.06 × 0.04 mm^3 was selected and mounted on a MITIGEN holder in Paratone oil on a XtaLAB Synergy, Single source at home/near, HyPix-Arc 100 diffractometer. The crystal was kept at a steady T = 100.00(10) K during data collection. The structure was solved with the ShelXS (Sheldrick, 2008) solution program using direct methods and by using Olex2 1.5-beta (Dolomanov et al., 2009) as the graphical interface. The model was refined with ShelXL 2019/3 (Sheldrick, 2015) using full matrix least squares minimisation on $\boldsymbol{F}^2$.

Crystal Data. $C_{30}H_{80}Fe_2P_{10}$, M_r = 862.34, monoclinic, $P2_1/n$ (No. 14), a = 16.3811(4) Å, b = 13.8289(3) Å, c = 19.7524(5) Å, β□= 90.480(2)°, α = γ = 90°, V = 4474.39(19) Å^3, T = 100.00(10) K, Z = 4, Z' = 1, μ(Mo K$_\alpha$) = 1.026, 56573 reflections measured, 9144 unique (R_{int} = 0.0584) which were used in all calculations. The final wR_2 was 0.2123 (all data) and R_1 was 0.0816 (I≥2 σ(I)).

Formula	$C_{30}H_{80}Fe_2P_{10}$
$D_{calc.}$/ g cm^{-3}	1.280
μ/mm^{-1}	1.026
Formula Weight	862.34
Colour	translucent pale orange
Shape	block-shaped
Size/mm^3	0.10×0.06×0.04
T/K	100.00(10)
Crystal System	monoclinic
Space Group	$P2_1/n$
a/Å	16.3811(4)
b/Å	13.8289(3)
c/Å	19.7524(5)
α/°	90
β/°	90.480(2)
γ/°	90
V/Å^3	4474.39(19)
Z	4
Z'	1
Wavelength/Å	0.71073
Radiation type	Mo K$_\alpha$
Θ_{min}/°	2.815
Θ_{max}/°	26.372
Measured Refl's.	56573
Indep't Refl's	9144
Refl's I≥2 σ(I)	7075
R_{int}	0.0584
Parameters	585
Restraints	1321
Largest Peak	3.189
Deepest Hole	-0.667
GooF	1.074
wR_2 (all data)	0.2123
wR_2	0.1990
R_1 (all data)	0.1019
R_1	0.0816

S7.6 [(dppe)FeH($Ph_2PCH_2CH_2PPh(C_6H_5)$)] 30

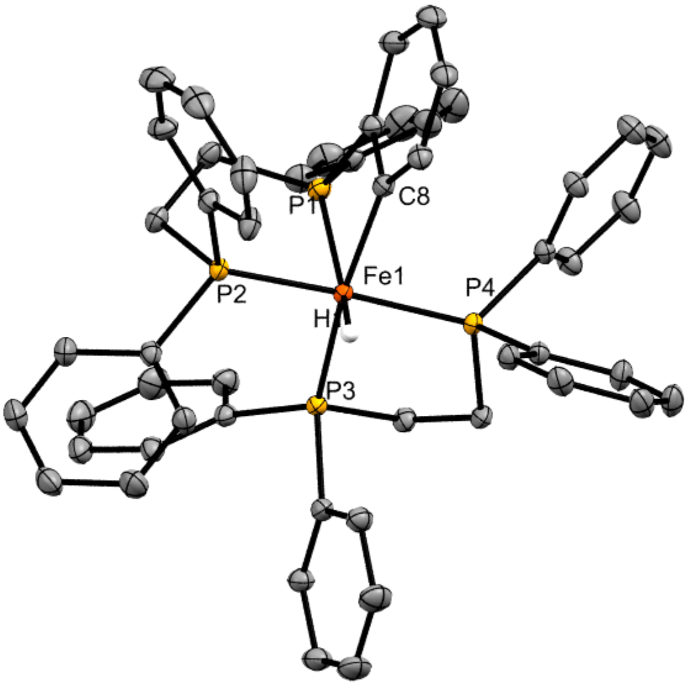

Formula	$C_{116.5}H_{126}Fe_2P_8$
$D_{calc.}$/ g cm^{-3}	1.276
μ/mm^{-1}	0.476
Formula Weight	1885.63
Colour	orange
Shape	block-shaped
Size/mm^3	0.34×0.19×0.09
T/K	100.10
Crystal System	triclinic
Space Group	P-1
a/Å	11.0746(9)
b/Å	20.0081(17)
c/Å	22.2287(19)
α/°	88.129(3)
β/°	86.550(3)
γ/°	87.443(3)
V/Å^3	4909.4(7)
Z	2
Z'	1
Wavelength/Å	0.71073
Radiation type	MoK_α
Θ_{min}/°	2.211
Θ_{max}/°	33.171
Measured Refl's.	671176
Indep't Refl's	37409
Refl's I≥2 σ(I)	33176
R_{int}	0.0466
Parameters	1222
Restraints	7
Largest Peak	0.845
Deepest Hole	-0.529
GooF	1.033
wR_2 (all data)	0.0820
wR_2	0.0783
R_1 (all data)	0.0358
R_1	0.0300

Experimental. The material was recrystallised from a saturated solution of pentane and methyl crotonate. A suitable crystal with dimensions 0.34 × 0.19 × 0.09 mm^3 was selected and mounted on a MITIGEN holder in Paratone oil. on a Bruker D8 Venture diffractometer. The crystal was kept at a steady T = 100.10 K during data collection. The structure was solved with the ShelXS (Sheldrick, 2008) solution program using heavy methods and by using Olex2 1.5-beta (Dolomanov et al., 2009) as the graphical interface. The model was refined with ShelXL 2019/3 (Sheldrick, 2015) using full matrix least squares minimisation on $\boldsymbol{F}^2$.

Crystal Data. $C_{116.5}H_{126}Fe_2P_8$, M_r = 1885.63, triclinic, P-1 (No. 2), a = 11.0746(9) Å, b = 20.0081(17) Å, c = 22.2287(19) Å, α = 88.129(3)°, β = 86.550(3)°, γ = 87.443(3)°, V = 4909.4(7) Å^3, T = 100.10 K, Z = 2, Z' = 1, μ(MoK_α) = 0.476, 671176 reflections measured, 37409 unique (R_{int} = 0.0466) which were used in all calculations. The final wR_2 was 0.0820 (all data) and R_1 was 0.0300 (I≥2 σ(I)).

_refine_special_details: Disordered pentane refined with geometric restraints. Fe-bound H atoms identified from a difference map and freely refined.

S8 Initial results

S8.1 Initial kinetic analysis comparing proteo- and deutro-substrates

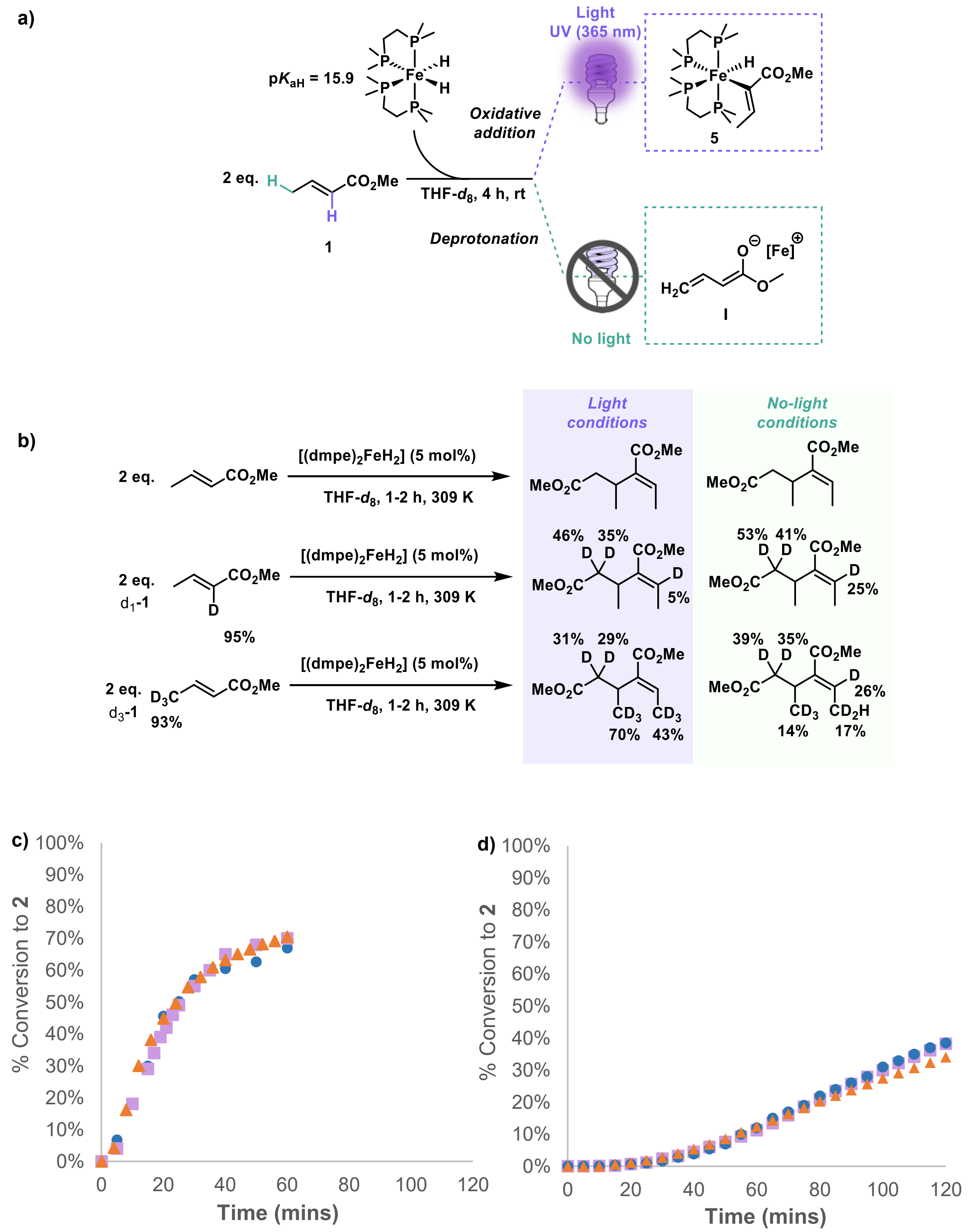

Supplementary Figure 6 – (a) Different products expected from the dimerisation of **1** (major expected product only shown for clarity) (b) % Deuterium incorporation for each position is calculated from the ^{1}H NMR resonances relative to the methyl ester resonance of **2** at δ 3.31 ppm *e.g.* 70% deuterium incorporation of a CD_3 unit indicates that on average, this position contains 2 deuteriums and 1 proton. N.B. (E),(Z)-2-ethylidene-3-methylpentanedioate **2** can contain 2 deuterium atoms when synthesised from d_1-**1**, 6 deuterium atoms when synthesised from d_3-**1**. The lower-than-maximum deuterium incorporation indicates that significant deuterium scrambling occurs). (c) Kinetics of methyl crotonate 1 dimerisation with UV (365 nm) for 1 hour, data points collected every 4 minutes. (d) Kinetics of the dimerisation of methyl crotonate **1** in no light for 2 hours, data points collected every 5 minutes. Product conversion is measured relative to starting material, as no other products are formed in quantifiable amounts.

S8.2 Variable time normalisation analysis (VTNA)

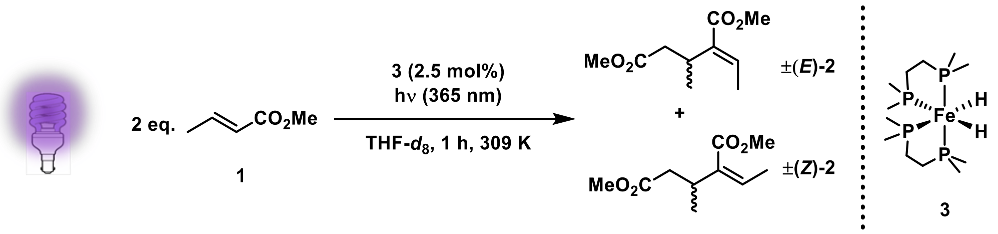

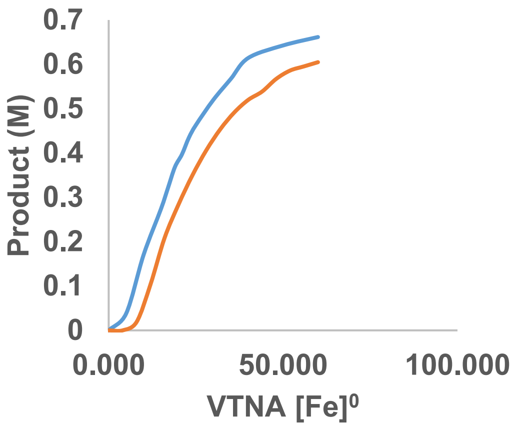

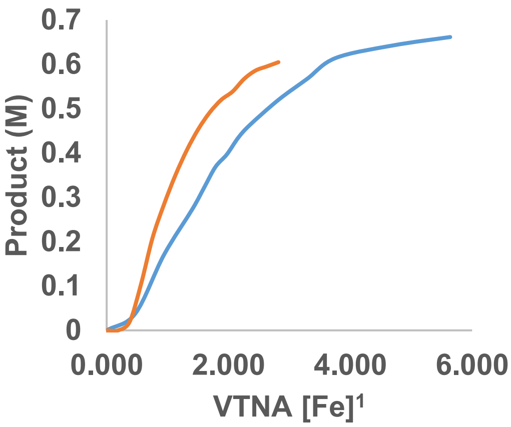

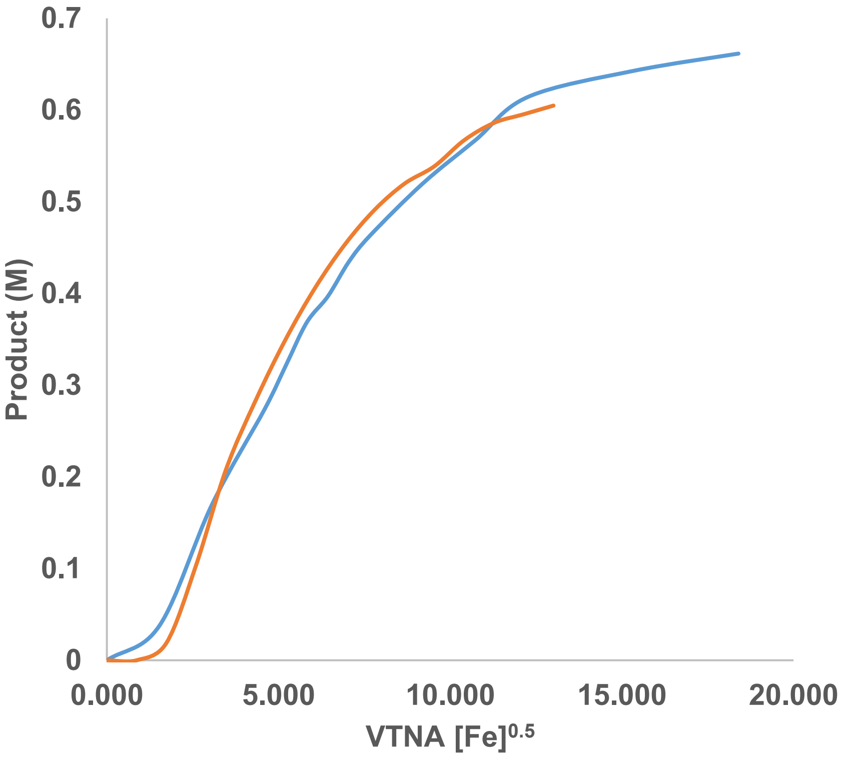

Supplementary Figure 7 – VTNA analysis halving the catalyst loading of $[FeH_2(dmpe)_2]$ **3** to 2.5 mol% under light conditions.

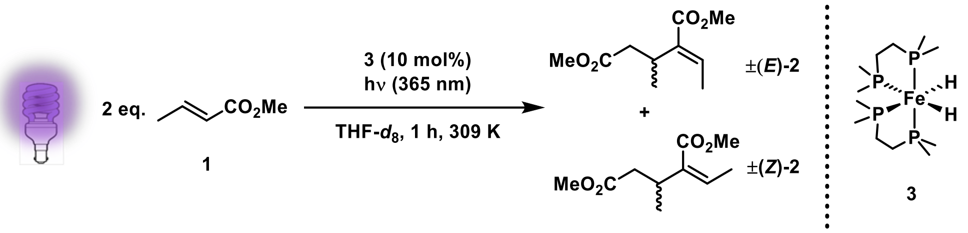

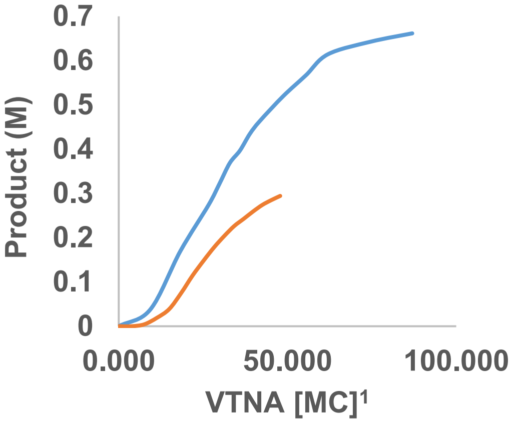

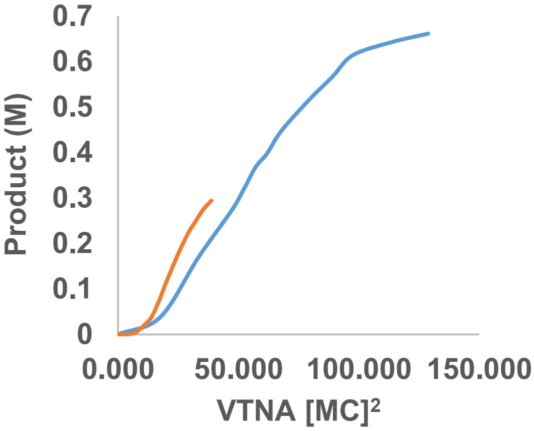

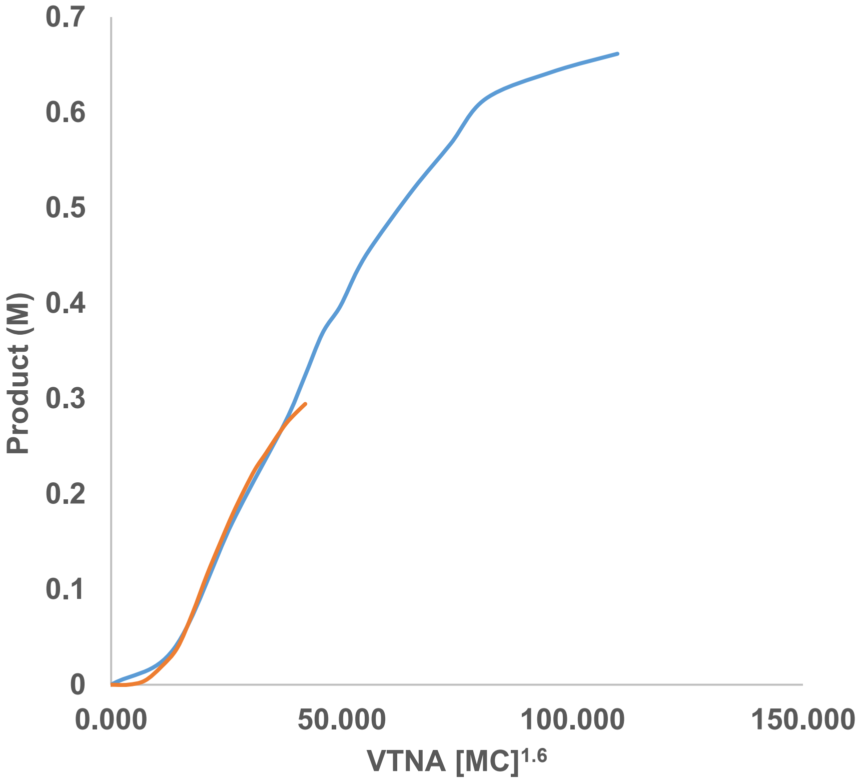

Supplementary Figure 8 – VTNA analysis halving the concentration of methyl crotonate **1** under light conditions.

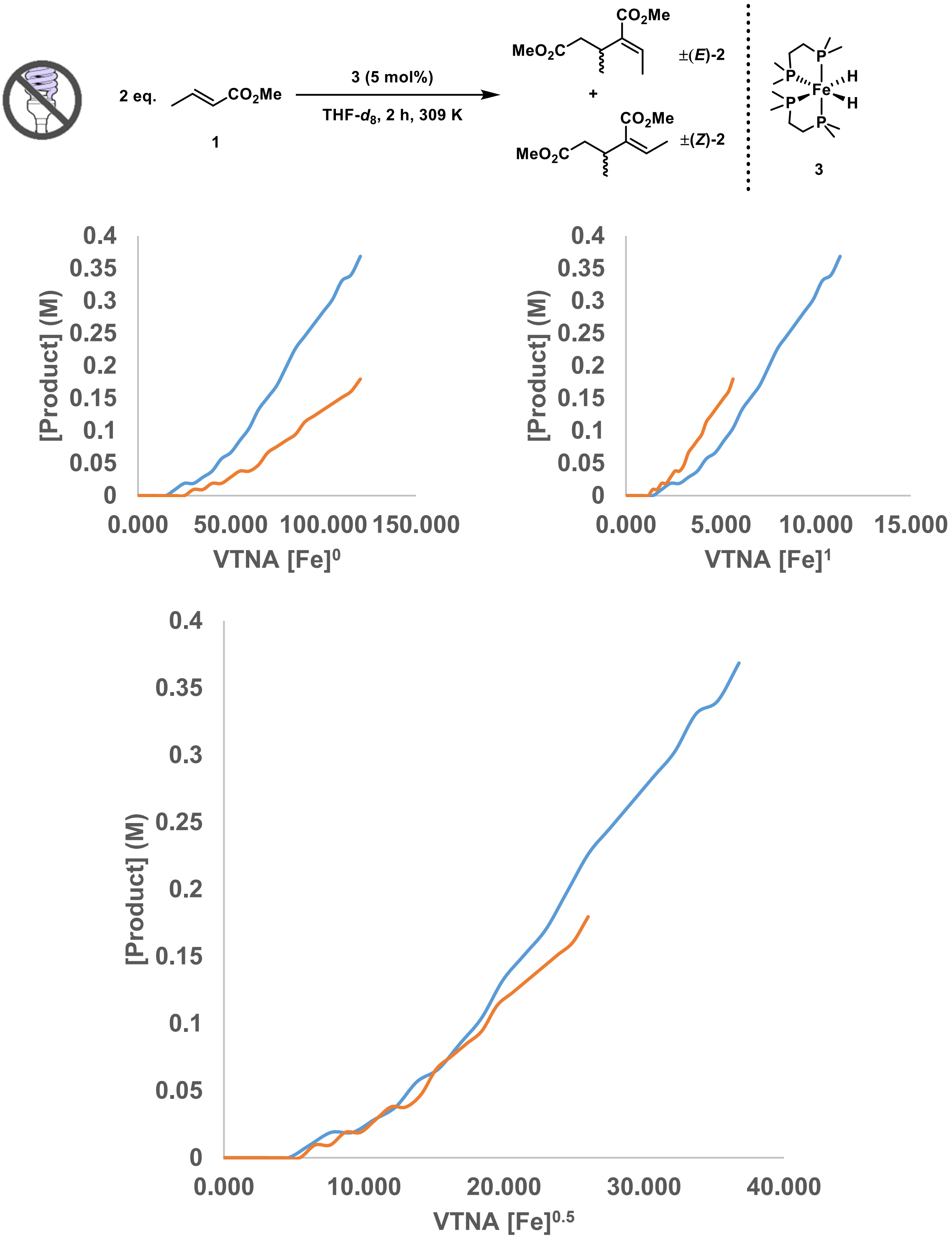

Supplementary Figure 9 – VTNA analysis halving the catalyst loading of $[FeH_2(dmpe)_2]$ **3** to 2.5 mol% under dark conditions.

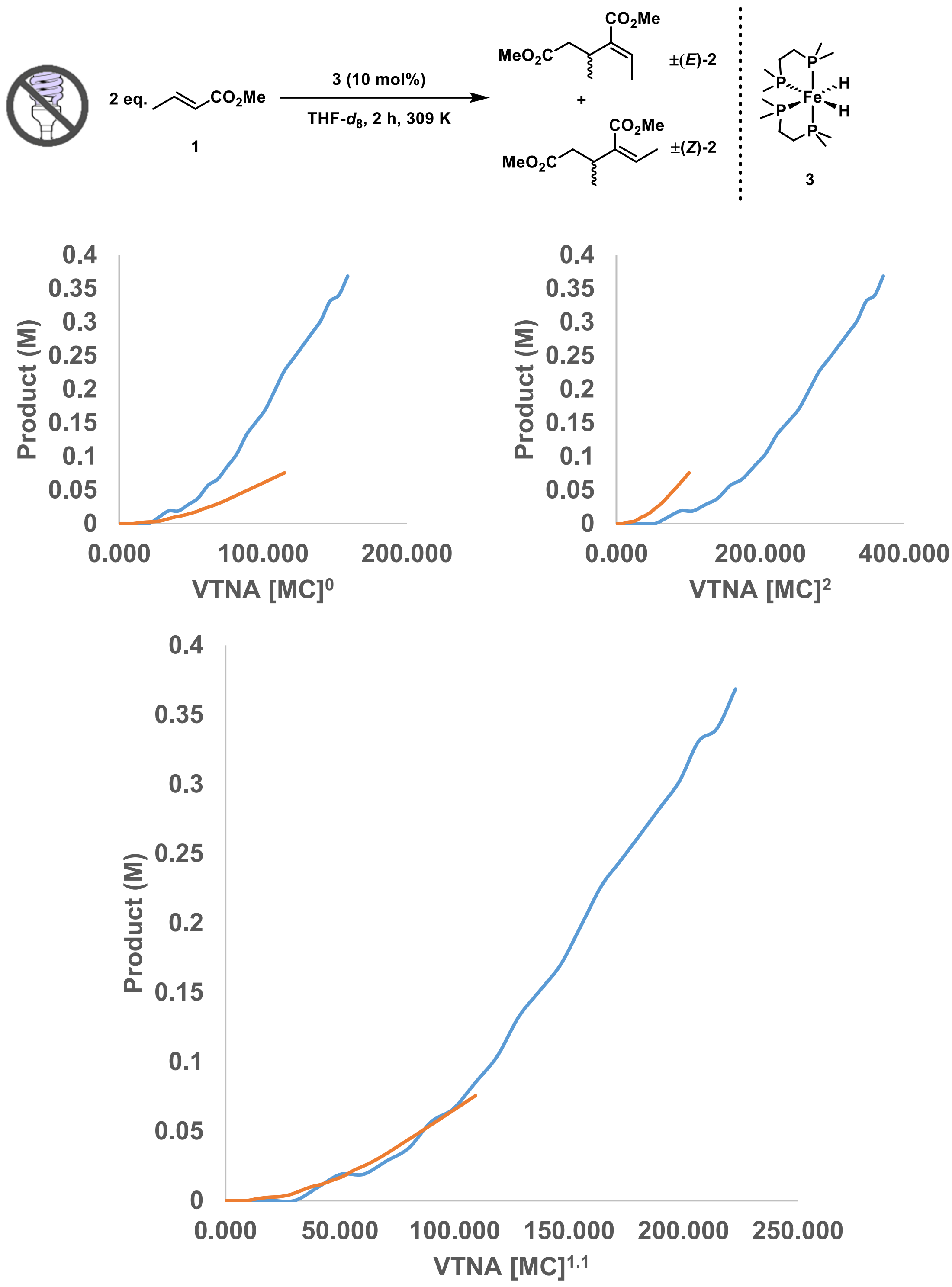

Supplementary Figure 10 – VTNA analysis halving the concentration of methyl crotonate **1** under dark conditions.

S9 NMR spectra

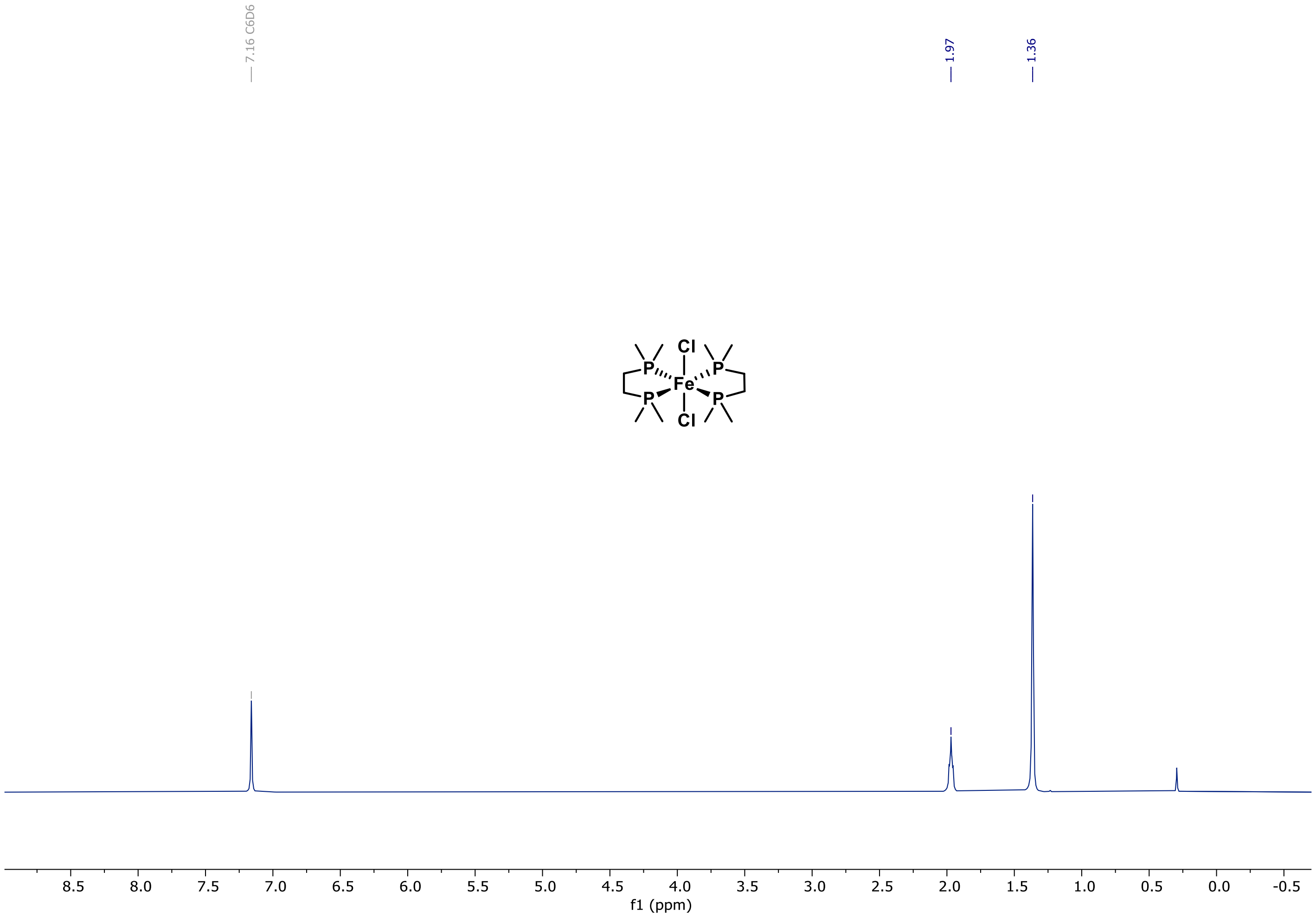

Supplementary Figure 11 – ^{1}H NMR (500MHz, C_6D_6) spectrum of [$(dmpe)_2FeCl_2$].

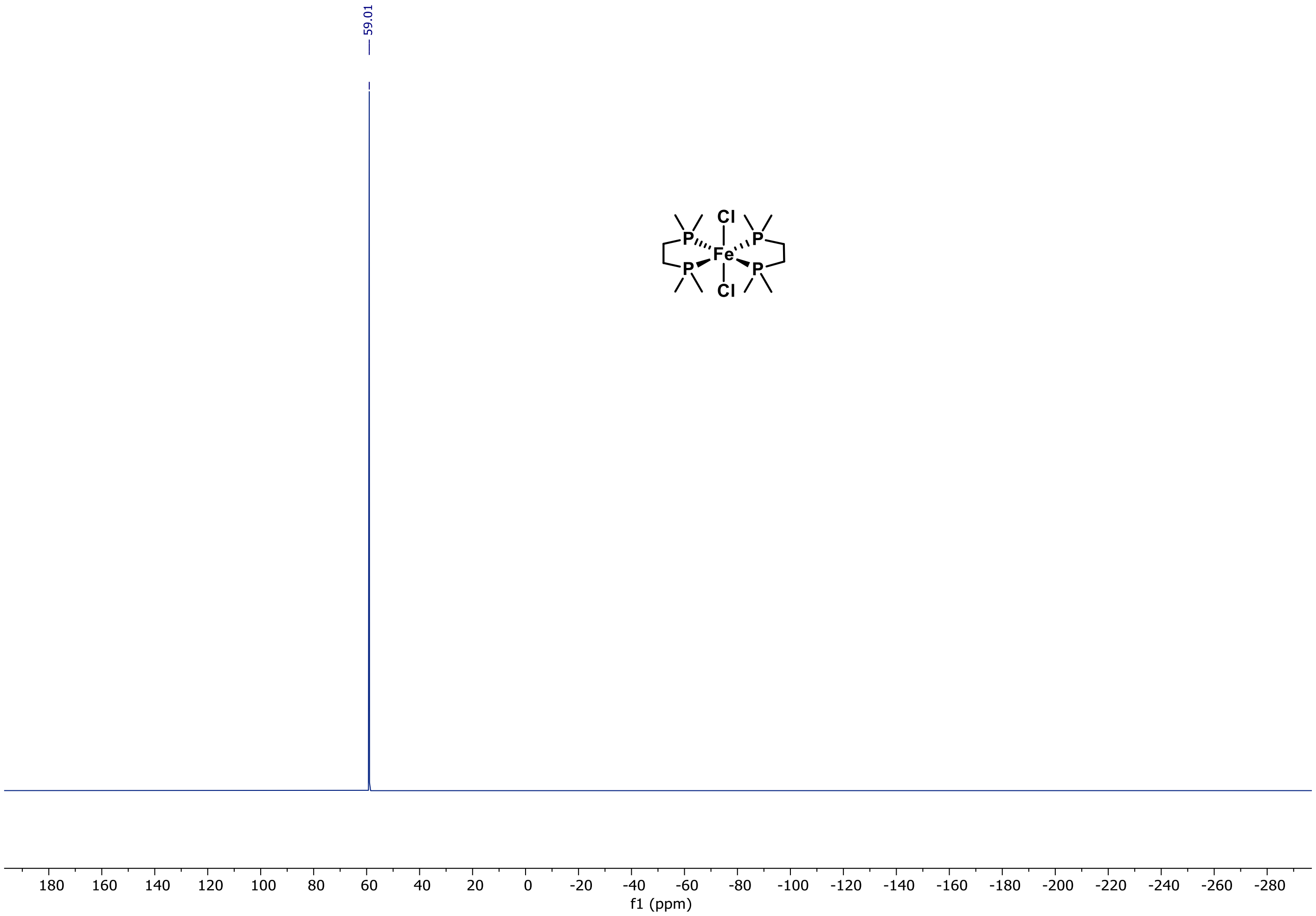

Supplementary Figure 12 – $^{31}P\{^1H\}$ NMR (202 MHz, C_6D_6) spectrum of $[(dmpe)_2FeCl_2]$.

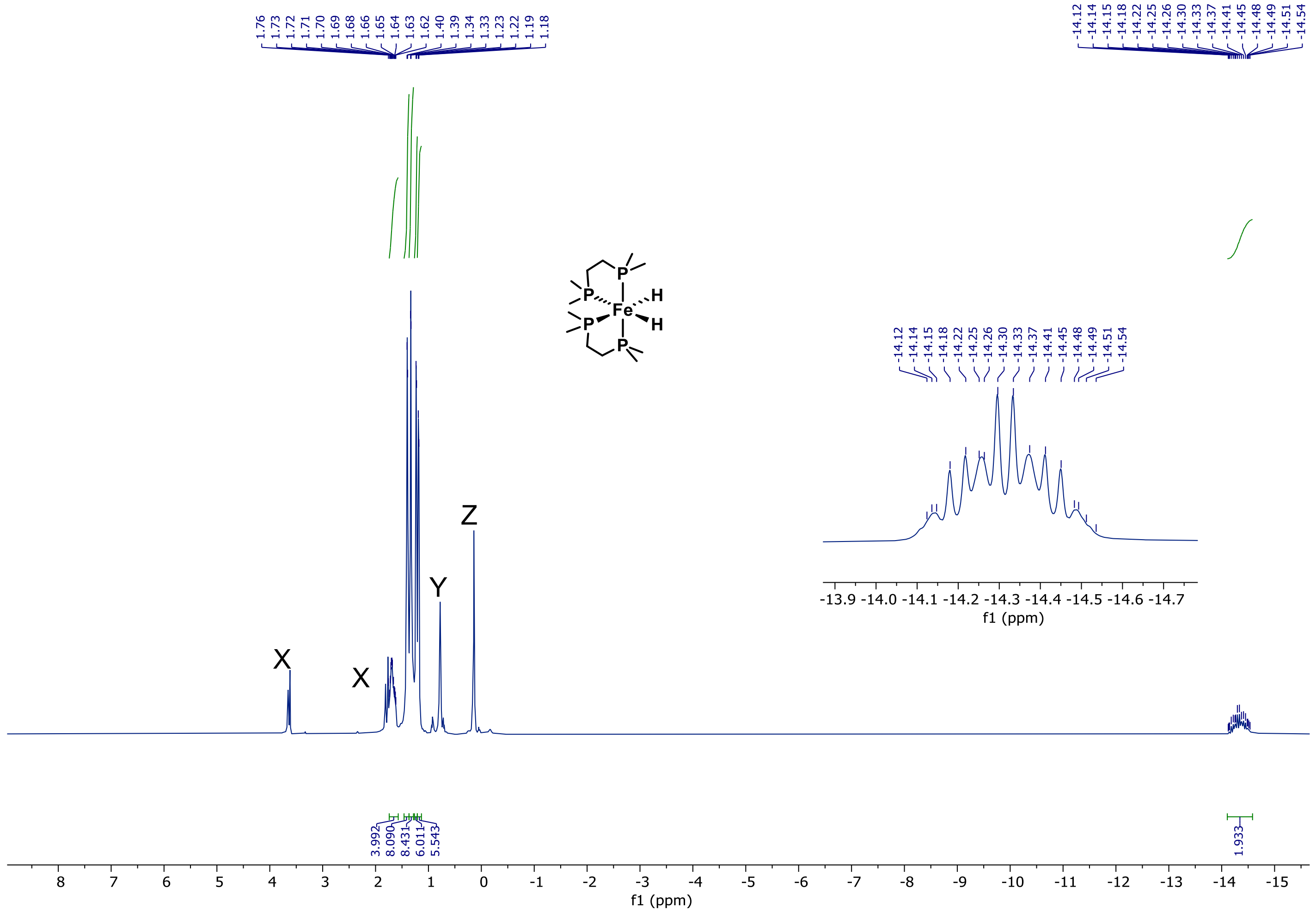

Supplementary Figure 13 – ^{1}H NMR (500 MHz, THF-d_8) spectrum of [(dmpe)$_2$FeH$_2$] **3**. The signals denoted by X are trace THF from the synthesis of [(dmpe)$_2$FeH$_2$] **3** as well as residual proteo-THF signals from THF-d_8. The signal denoted by Y is pentane, whilst the signal denoted by Z is residual grease from the solvent.

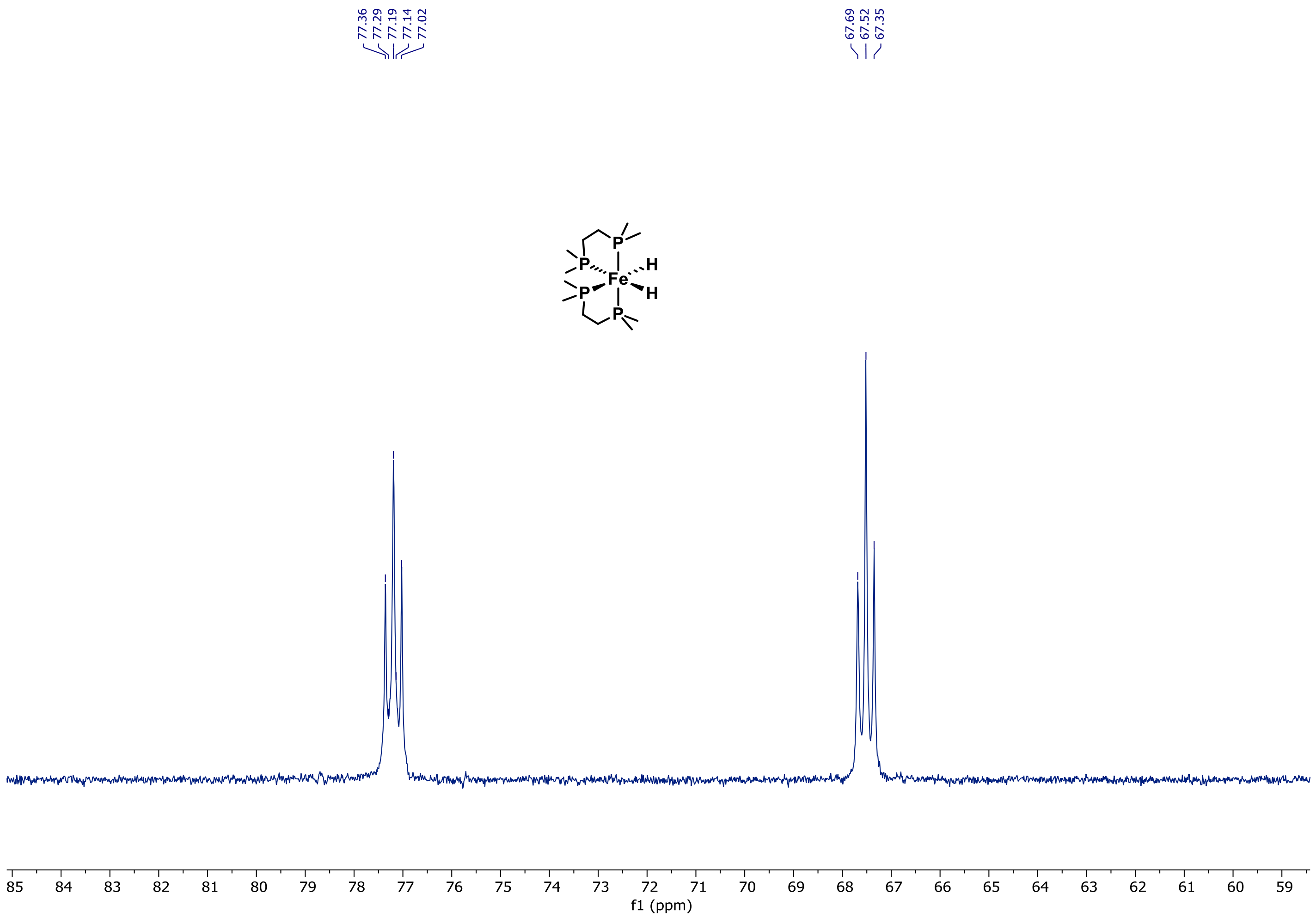

Supplementary Figure 14 – $^{31}P\{^{1}H\}$ NMR (202 MHz, THF) spectrum of $[(dmpe)_2FeH_2]$ **3**.

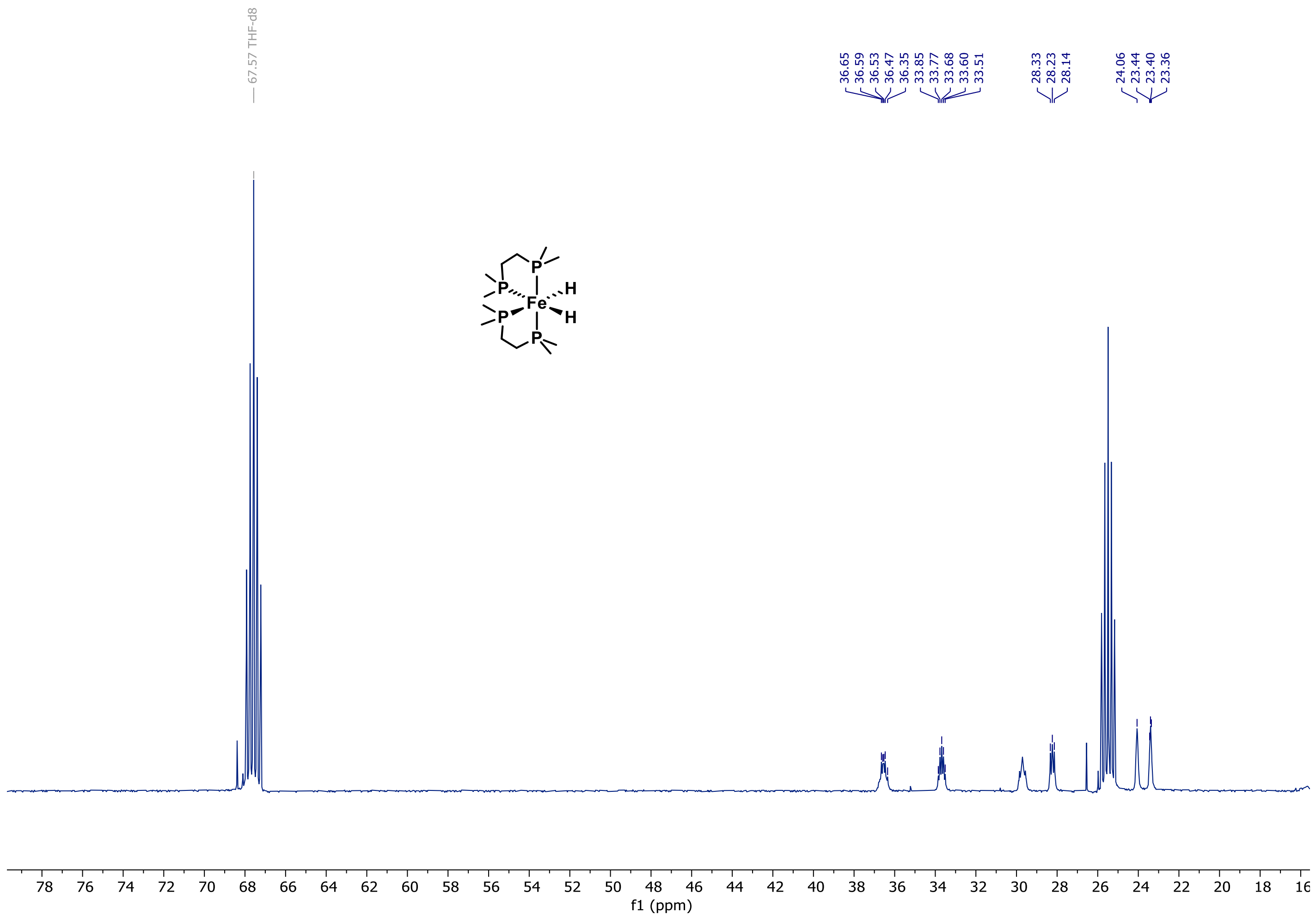

Supplementary Figure 15 – ^{13}C NMR (126 MHz, THF-d_8) spectrum of [(dmpe)$_2$FeH$_2$] **3**.

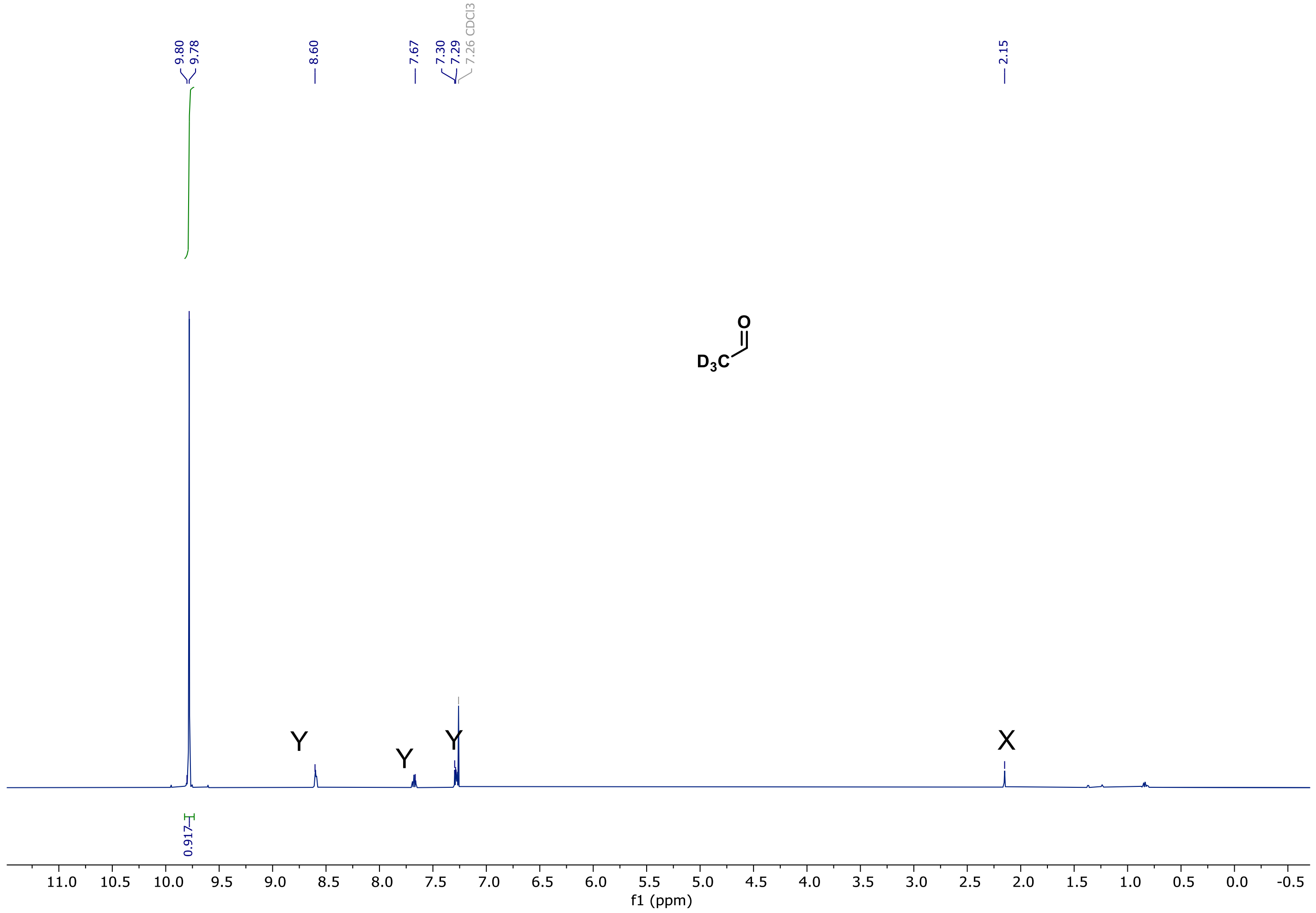

Supplementary Figure 16 – ^{1}H NMR (500MHz, $CDCl_3$) spectrum of acetaldehyde-d_3 (98% D-incorporation) The signal denoted by X is residual proteo-acetaldehyde, whilst the signals denoted by Y are trace amounts of pyridine which are removed in the next step of the synthesis.

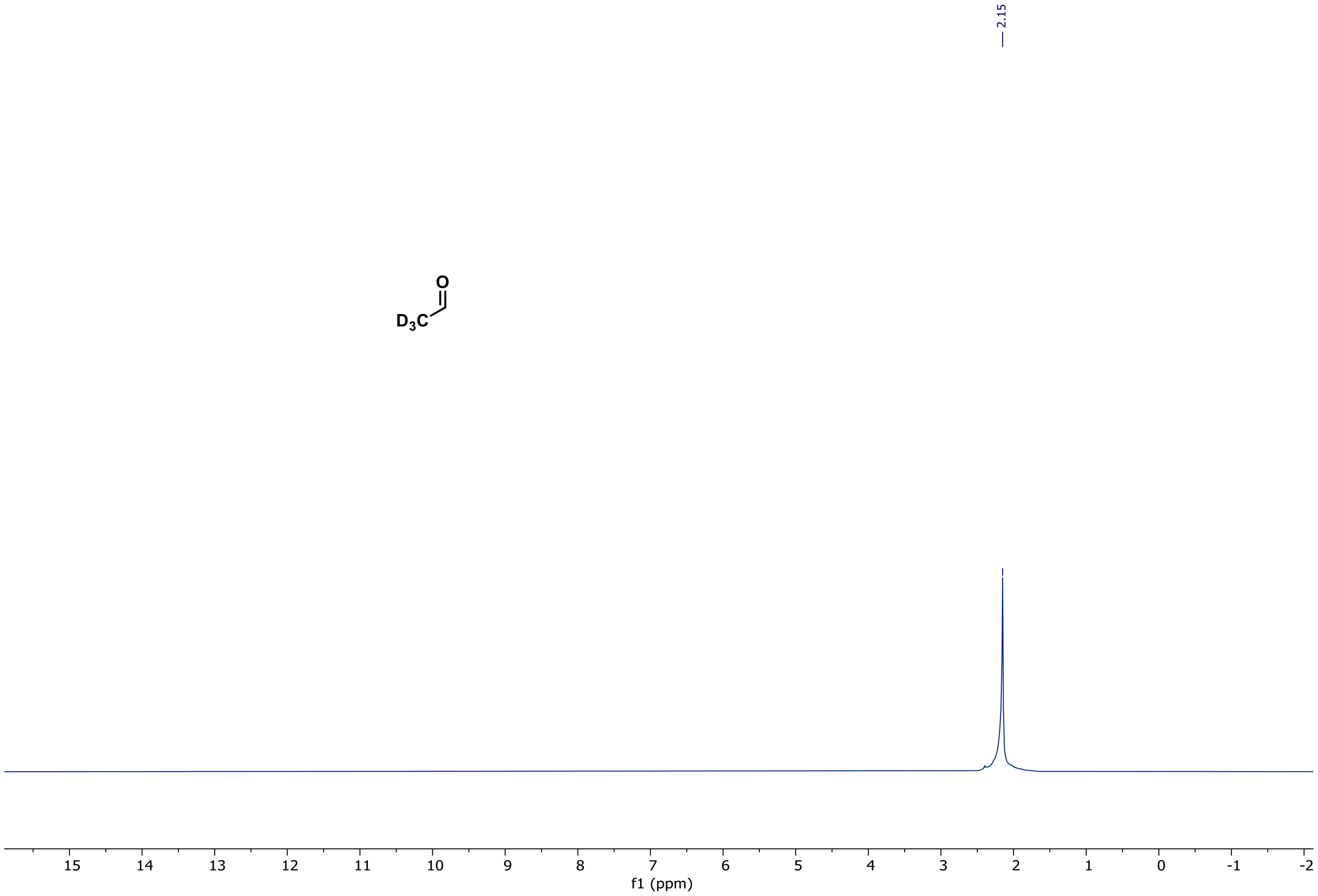

Supplementary Figure 17 – ^{2}H NMR (77 MHz, CH_2Cl_2) spectrum of acetaldehyde-d_3.

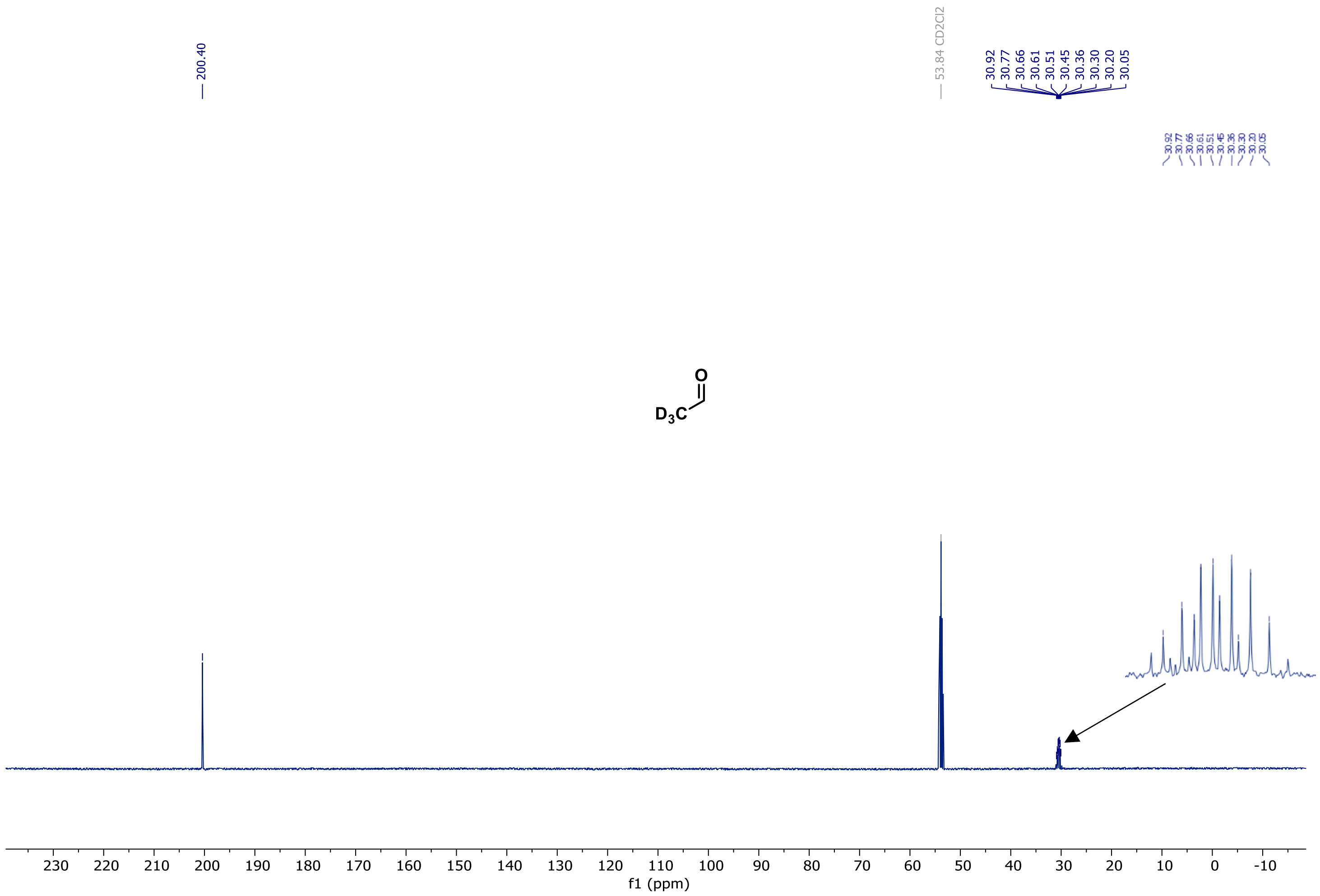

Supplementary Figure 18 – ^{13}C NMR (126 MHz, CD_2Cl_2) spectrum of acetaldehyde-*d*$_3$. The signal at δ 30 ppm contains some partially deuterated ($CD_{3-x}H_xC{=}O$) acetaldehyde.

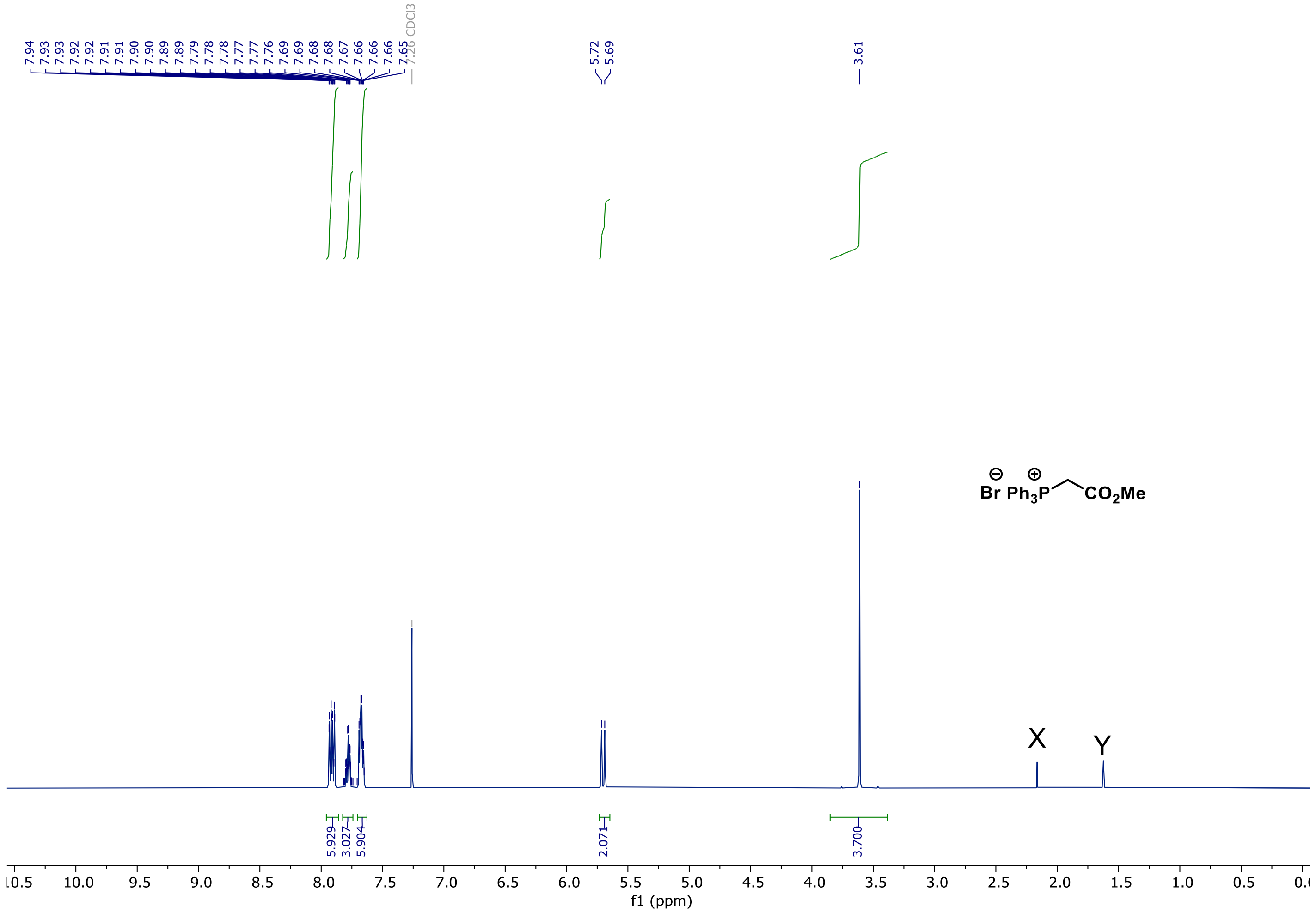

Supplementary Figure 19 – ^{1}H NMR (500 MHz, $CDCl_3$) spectrum of methoxycarbonylmethyltriphenylphosphonium bromide. The signal denoted by X is trace acetone, whilst the signal denoted by Y is trace water from the chloroform.

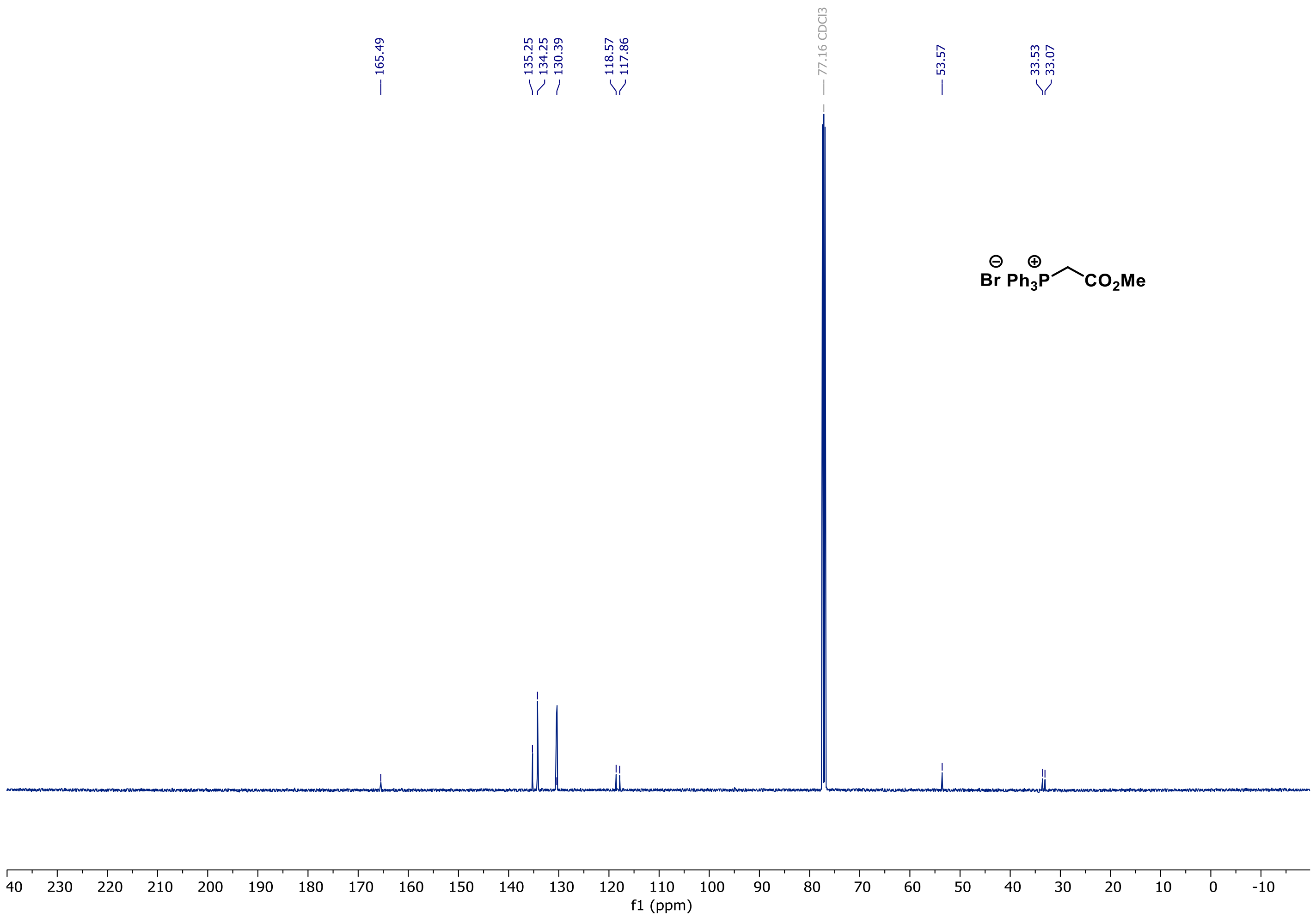

Supplementary Figure 20 – ^{13}C NMR (126 MHz, $CDCl_3$) spectrum of methoxycarbonylmethyltriphenylphosphonium bromide.

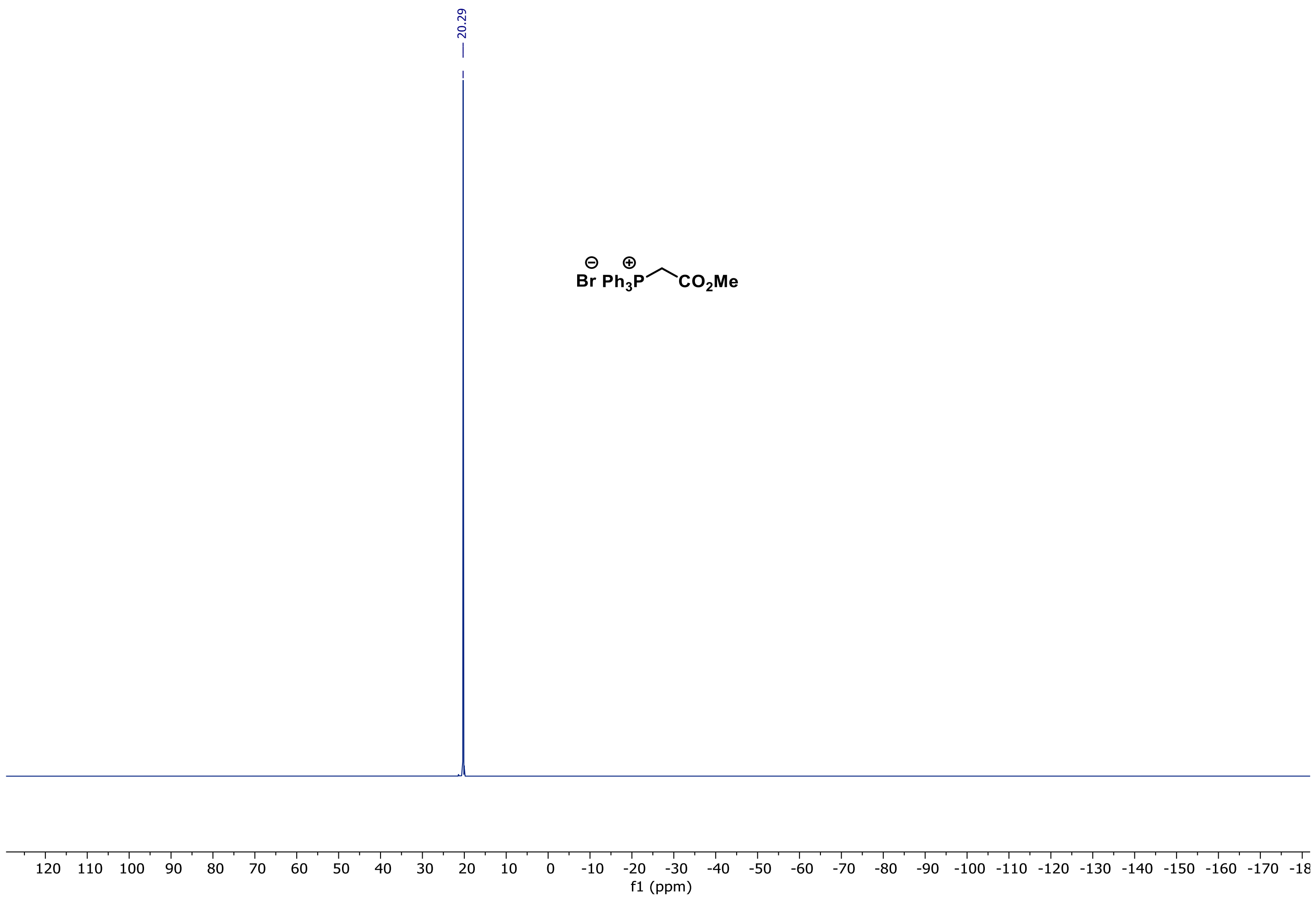

Supplementary Figure 21 – $^{31}P\{^{1}H\}$ NMR (202 MHz, $CDCl_3$) spectrum of methoxycarbonylmethyltriphenylphosphonium bromide.

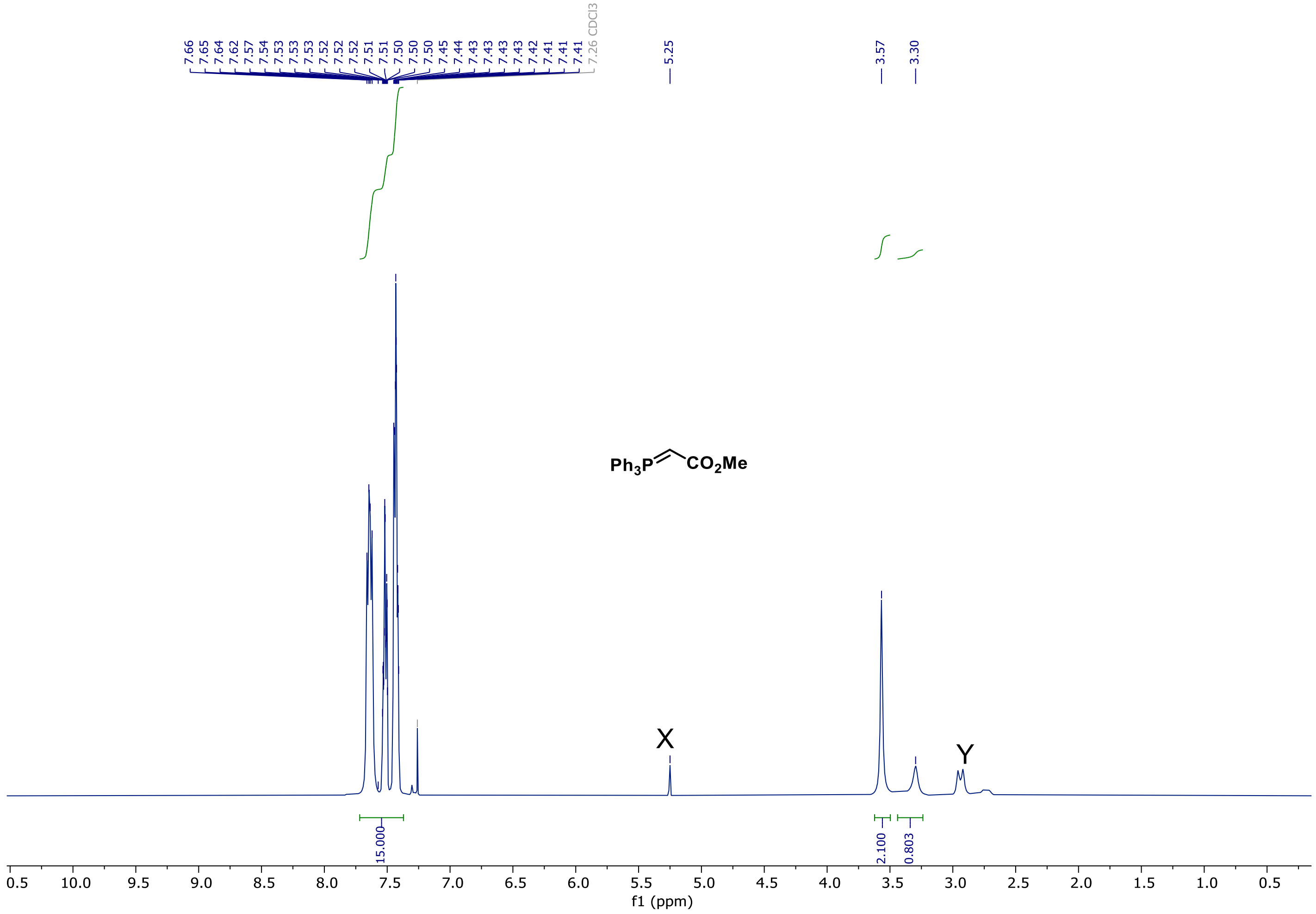

Supplementary Figure 22 – ^{1}H NMR (500 MHz, $CDCl_3$) of methyl (triphenylphosphoranylidene)acetate. The signal denoted by X is trace CH_2Cl_2 from the synthesis, whilst the signal denoted by Y are unknown contaminants.

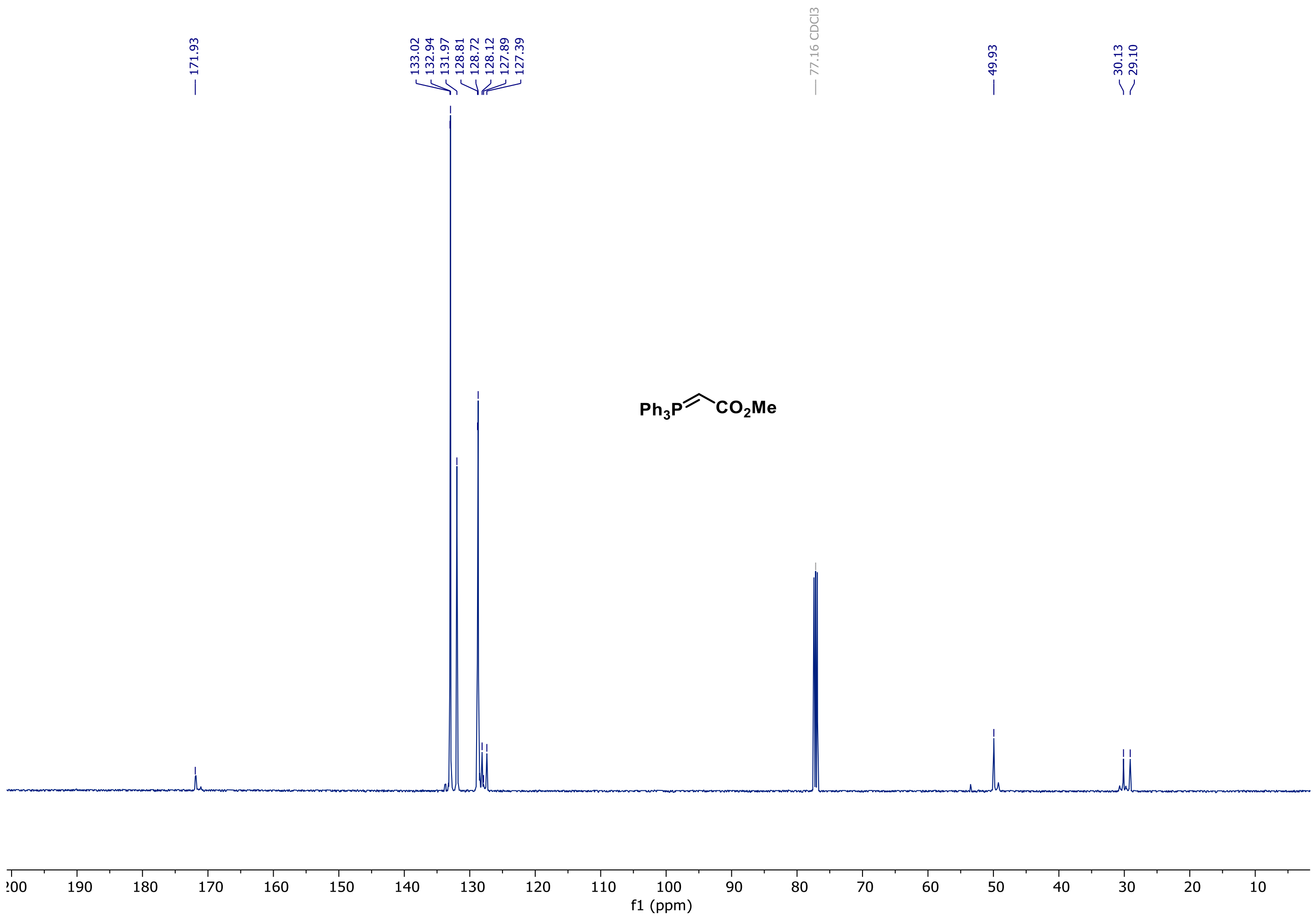

Supplementary Figure 23 – ^{13}C NMR (126 MHz, $CDCl_3$) spectrum of methyl (triphenylphosphoranylidene)acetate.

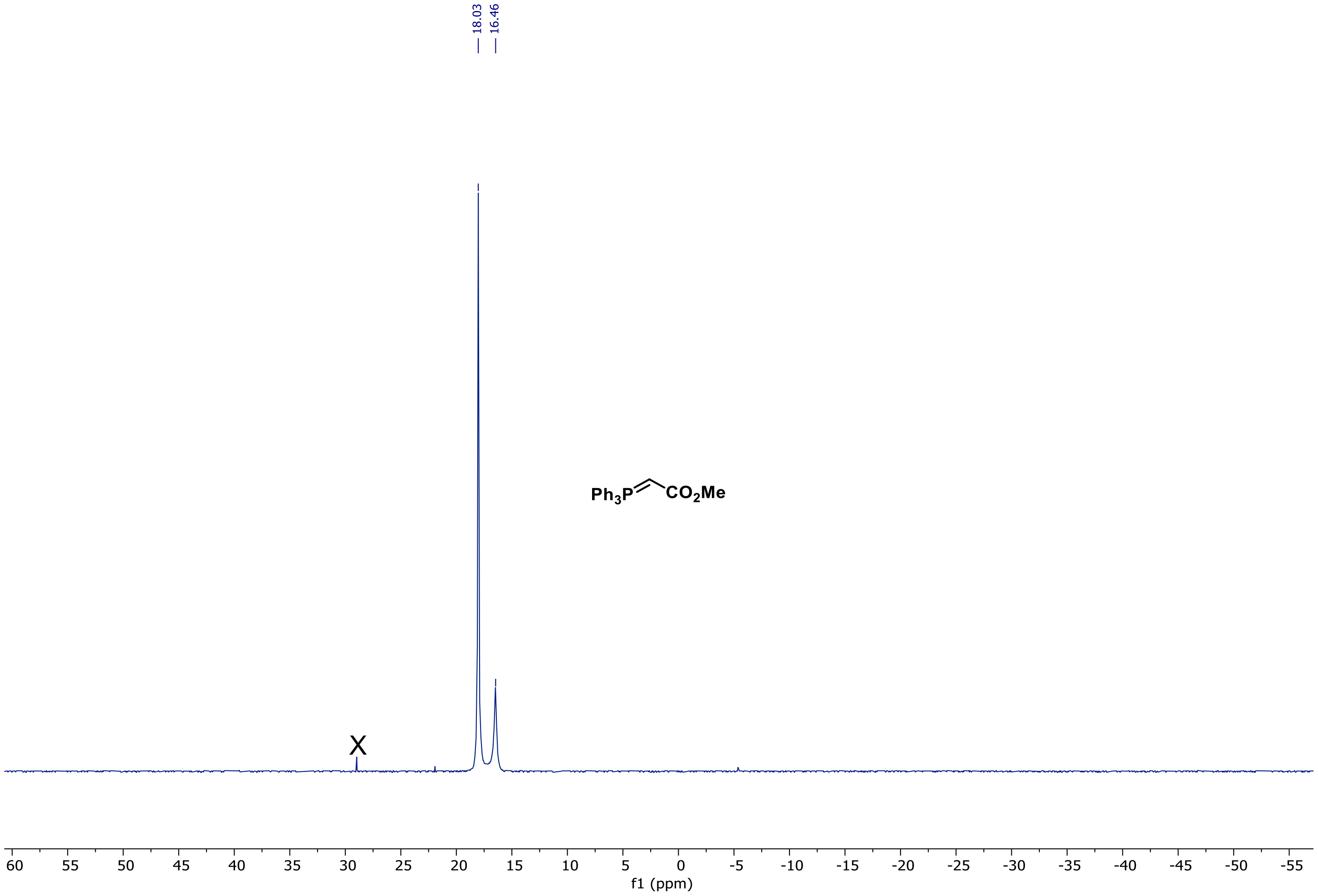

Supplementary Figure 24 – $^{31}P\{^1H\}$ NMR (202 MHz, $CDCl_3$) spectrum of methyl (triphenylphosphoranylidene)acetate. The signal denoted by X is trace triphenylphosphine oxide, a decomposition product from the ylide.

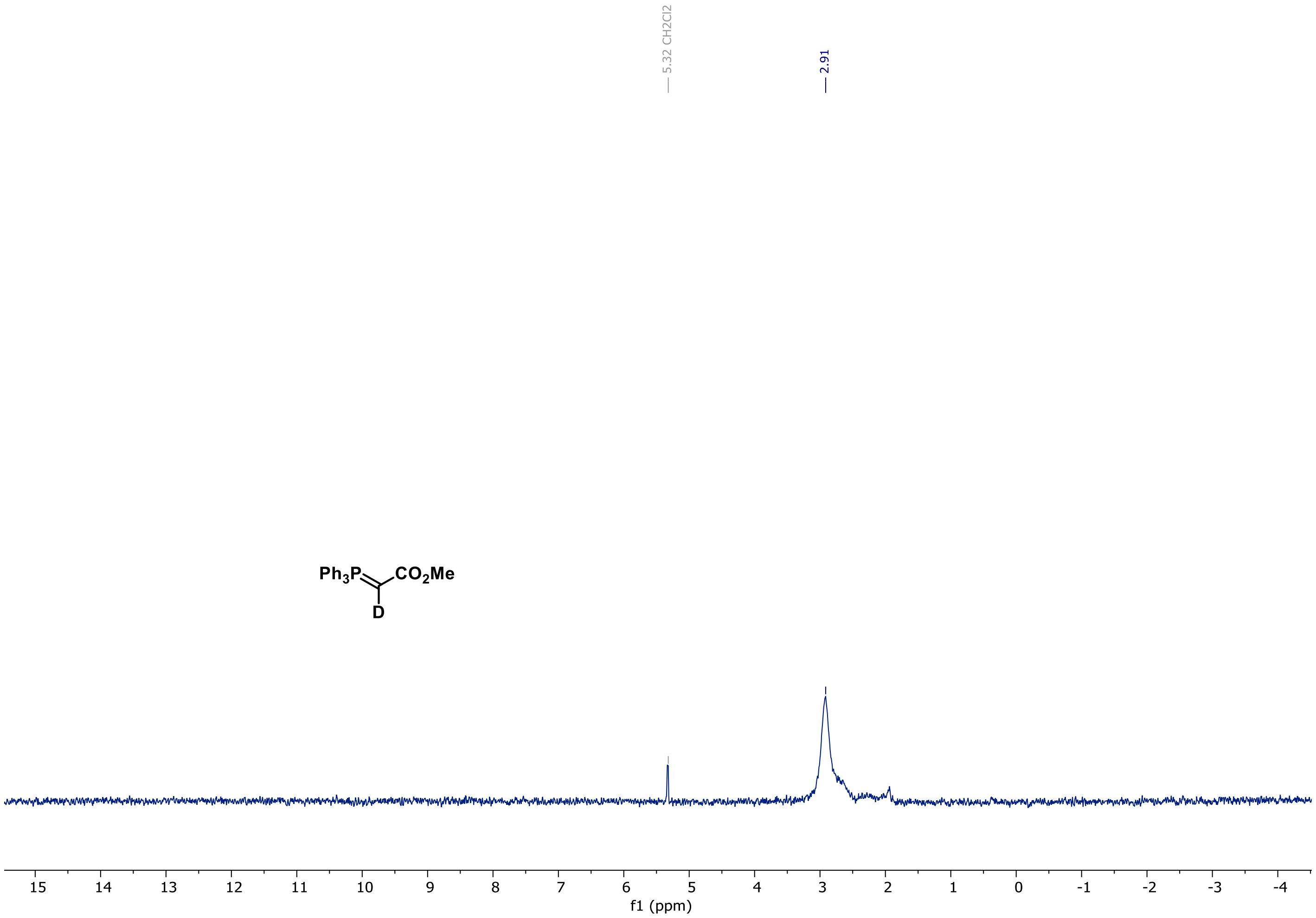

Supplementary Figure 25 – ^{2}H NMR spectrum (500 MHz, CH_2Cl_2) of methyl (triphenylphosphoranylidene)acetate-d_1.

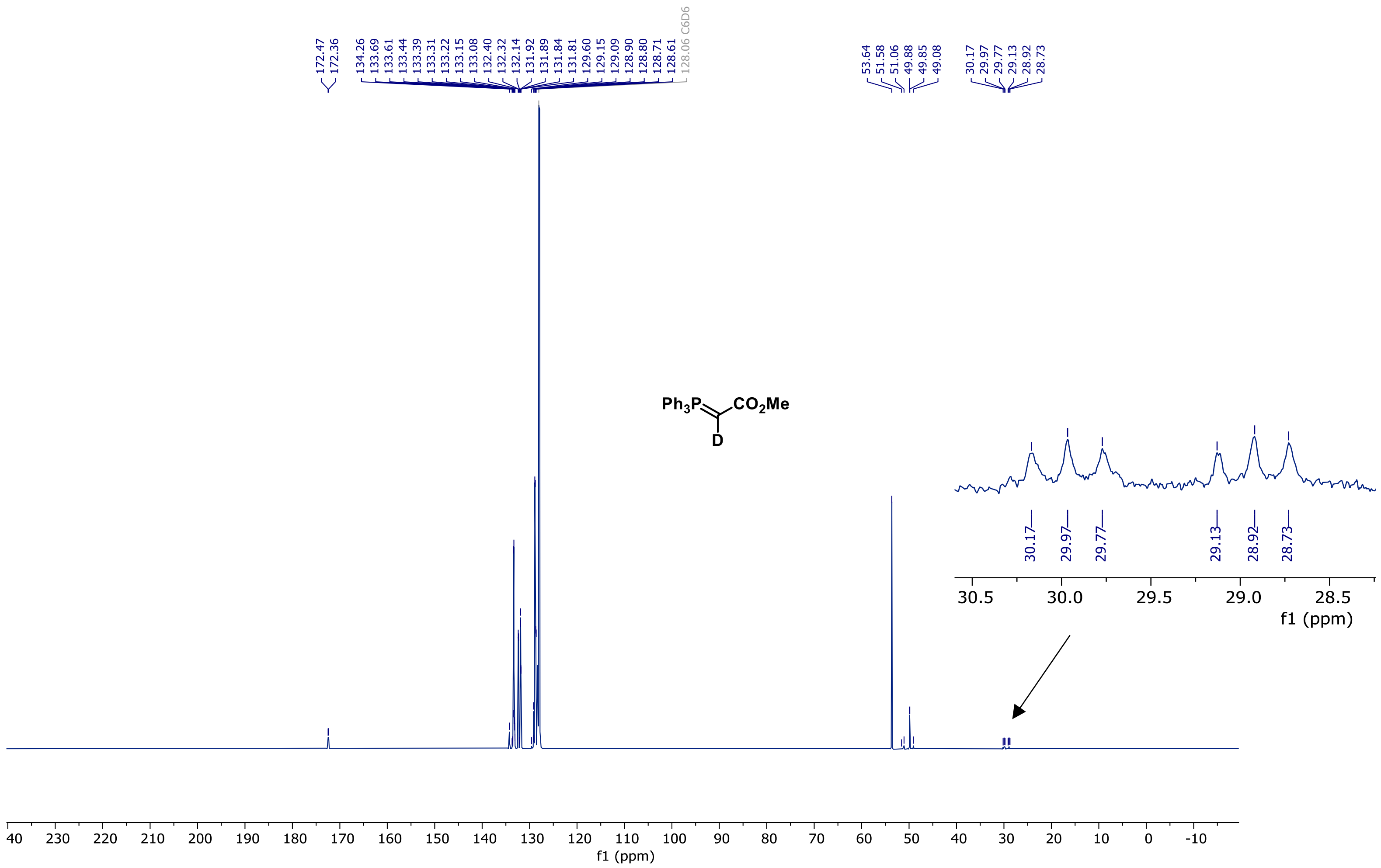

Supplementary Figure 26 – ^{13}C NMR (126 MHz, C_6D_6) of methyl (triphenylphosphoranylidene)acetate-d_1.

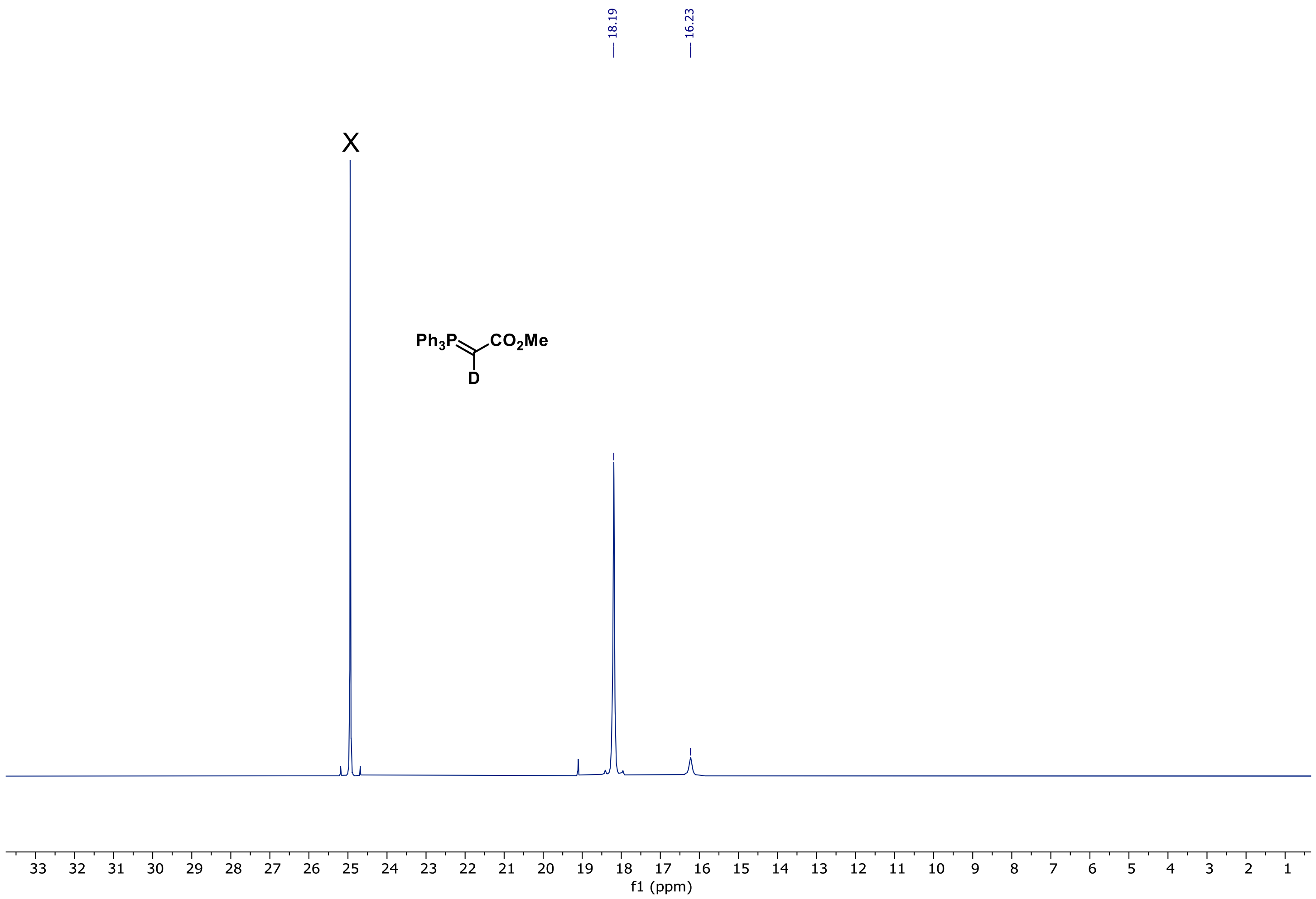

Supplementary Figure 27 – $^{31}P\{^{1}H\}$ NMR (202 MHz, C_6D_6) of methyl (triphenylphosphoranylidene)acetate-*d₁*. The signal denoted by X is triphenylphosphine oxide, which is removed in the next step of the synthesis.

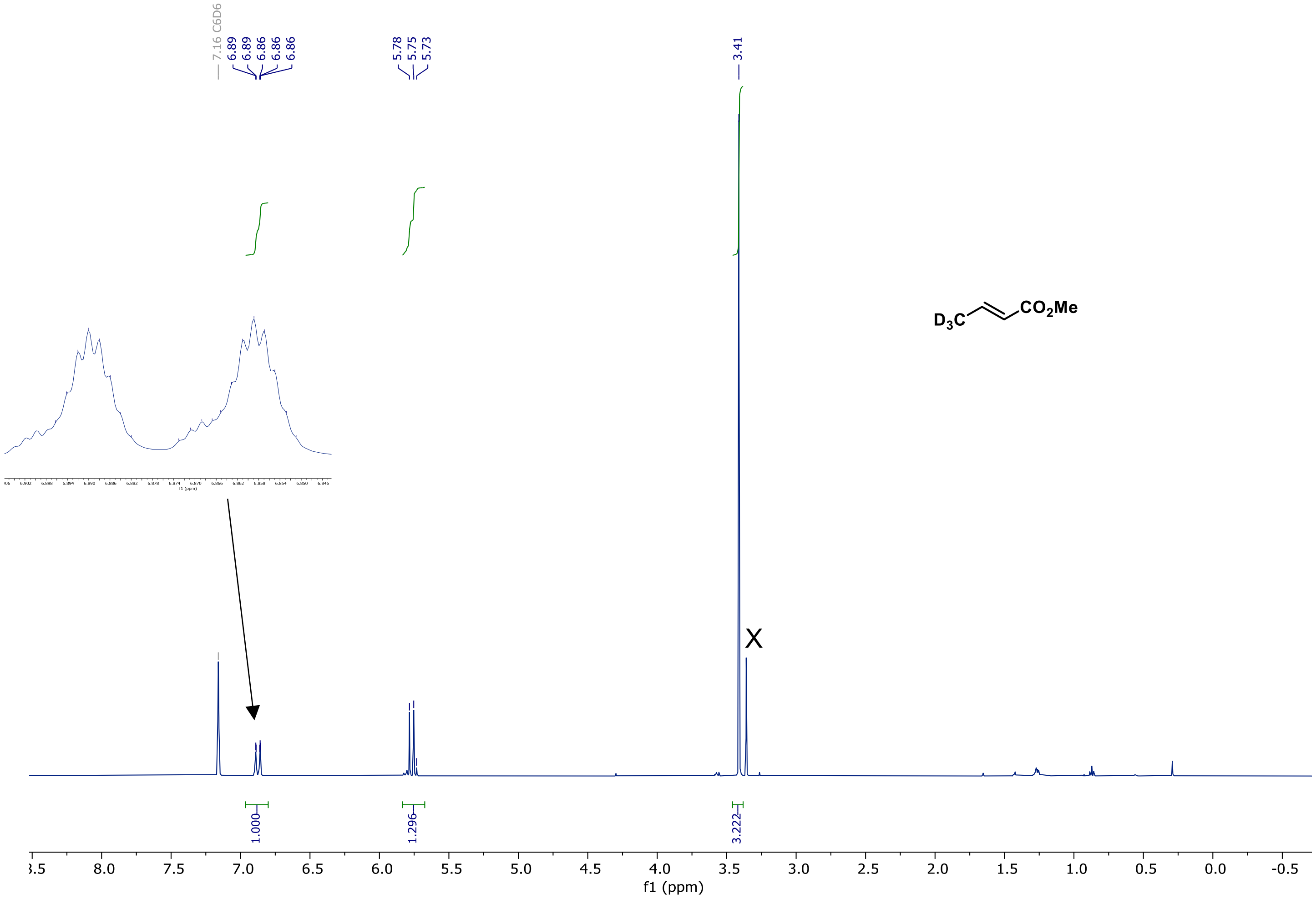

Supplementary Figure 28 – ^{1}H NMR (500 MHz, C_6D_6) spectrum of methyl crotonate-*d*3. The signal denoted by X is trace amounts of partially deuterated methyl crotonate.

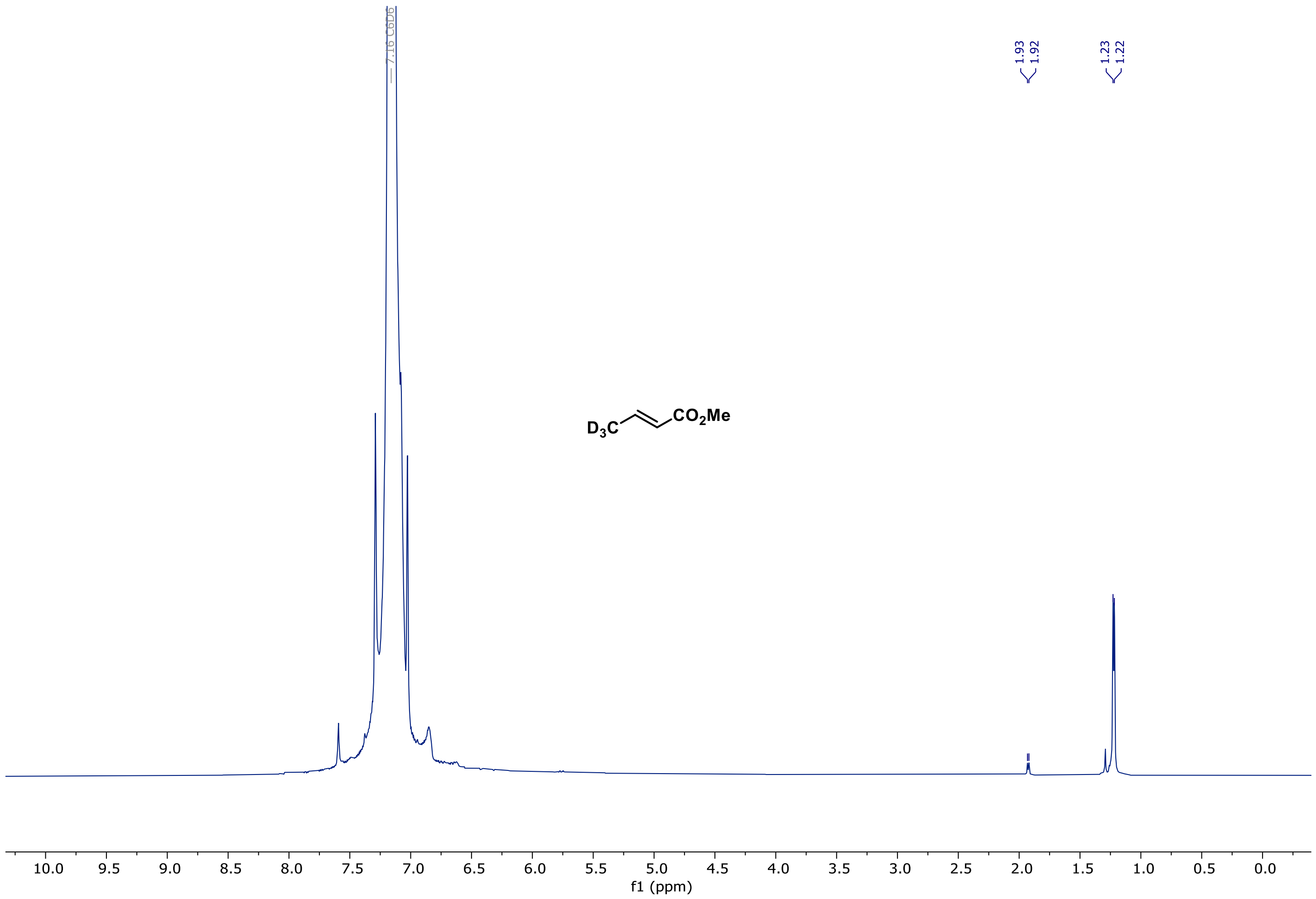

Supplementary Figure 29 – ^{2}H NMR (92 MHz, C_6D_6) spectrum of methyl crotonate-d_3.

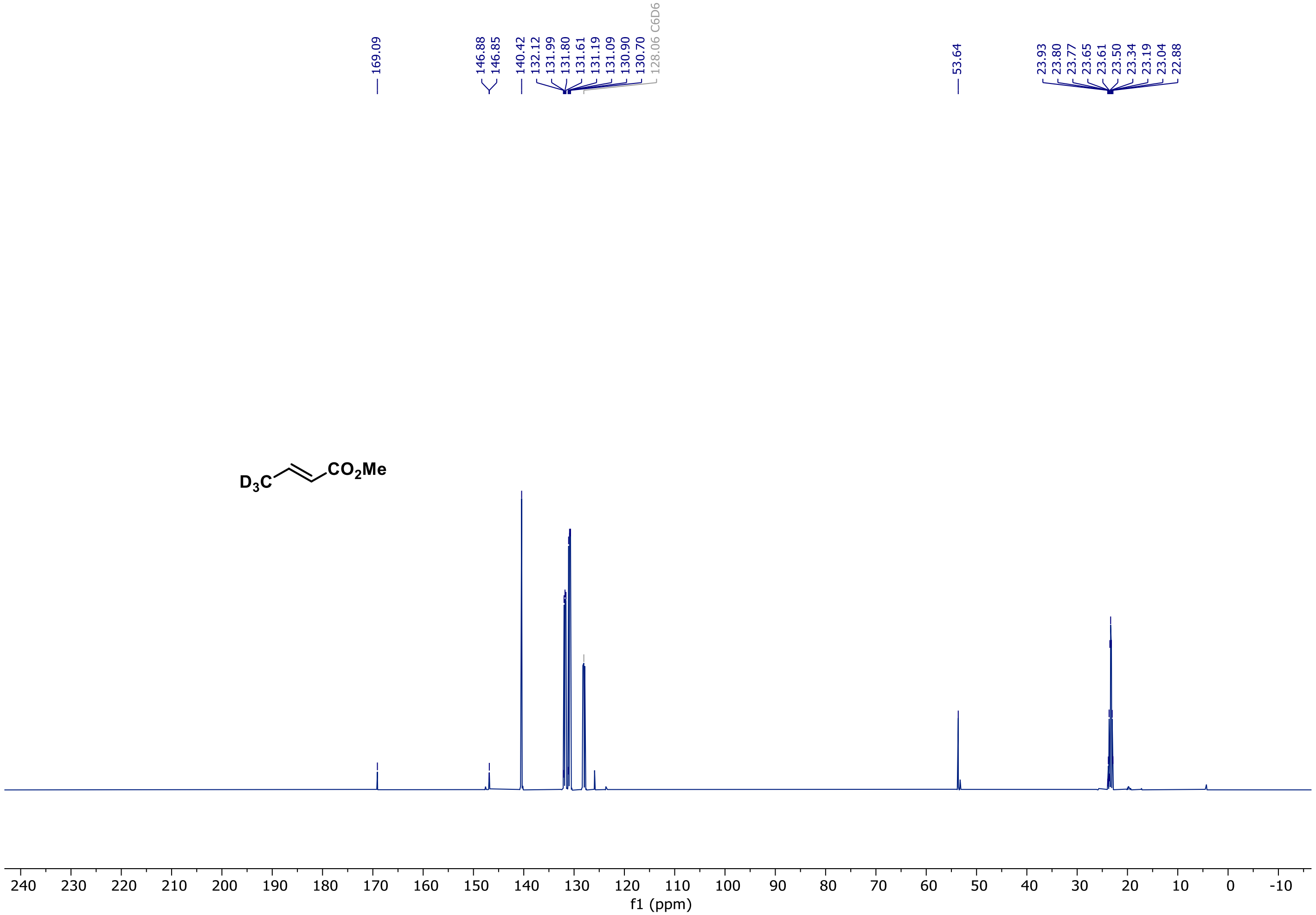

Supplementary Figure 30 – ^{13}C NMR (126 MHz, C_6D_6) spectrum of methyl crotonate-d_3.

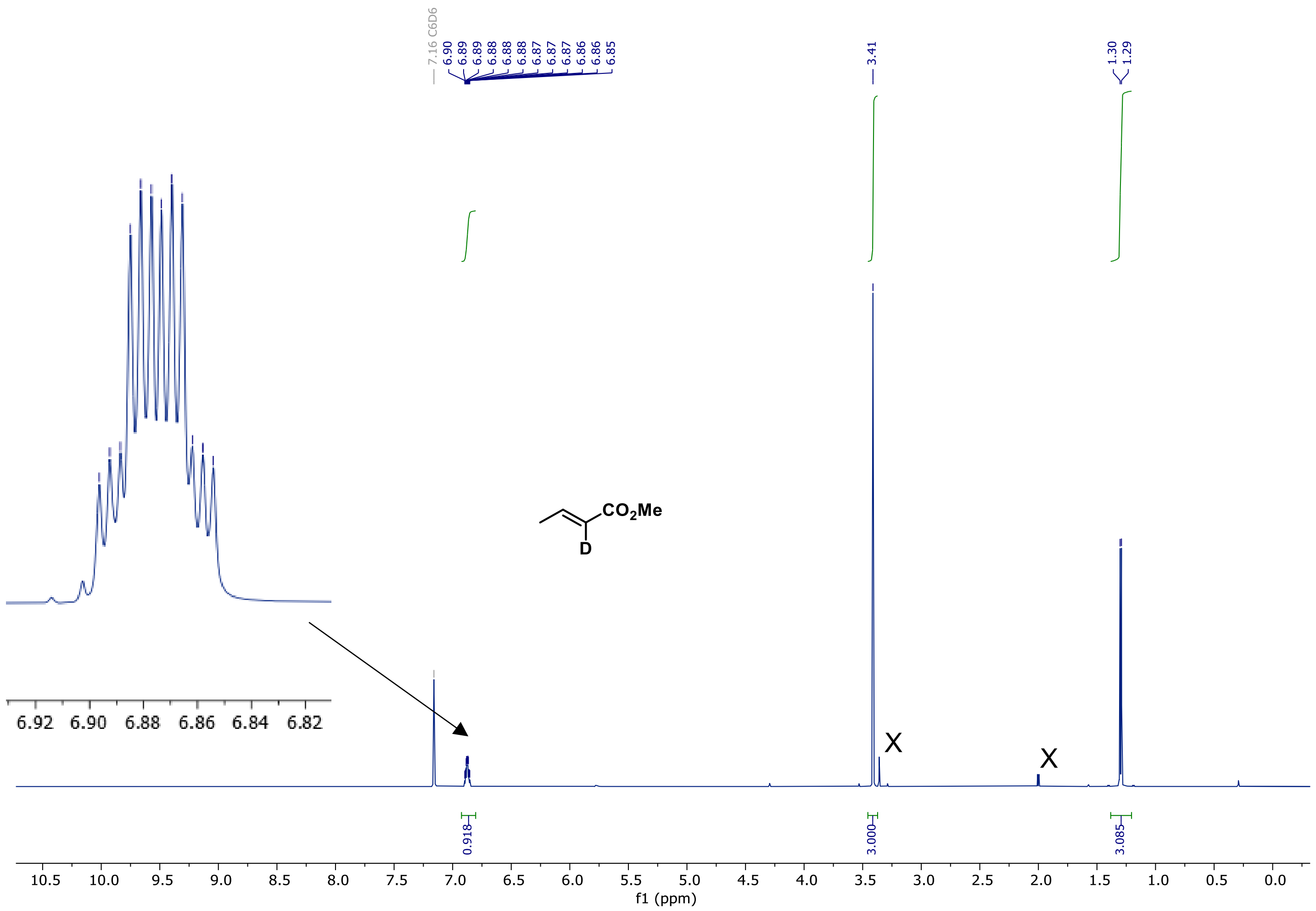

Supplementary Figure 31 – ^{1}H NMR (601 MHz, C_6D_6) spectrum of methyl crotonate-d_1. The signals denoted by X are trace amounts of proteo-methyl crotonate.

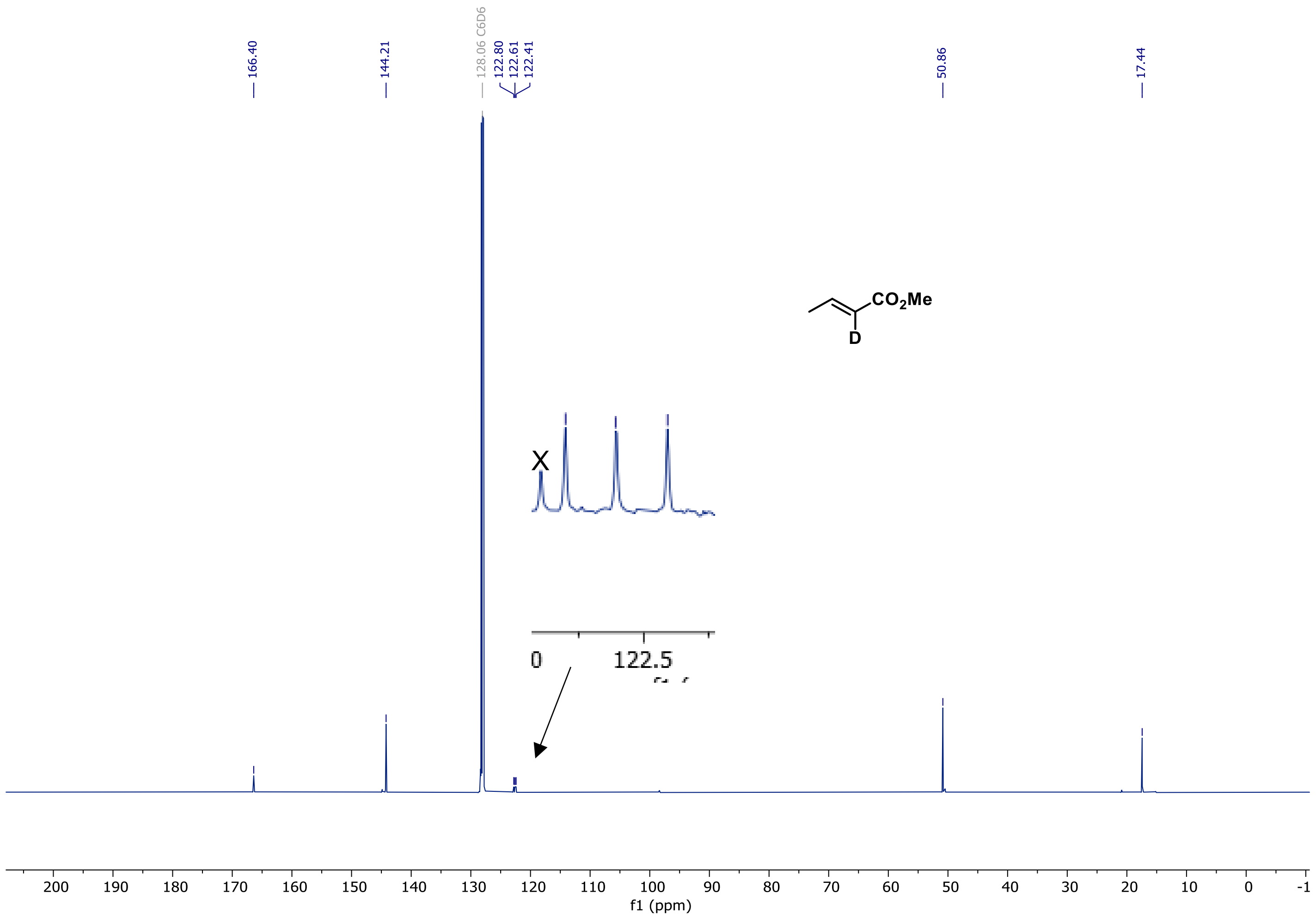

Supplementary Figure 32 – ^{13}C NMR spectrum (126 MHz, C_6D_6) of methyl crotonate-d_1. The signal denoted by X is trace amounts of proteo-methyl crotonate.

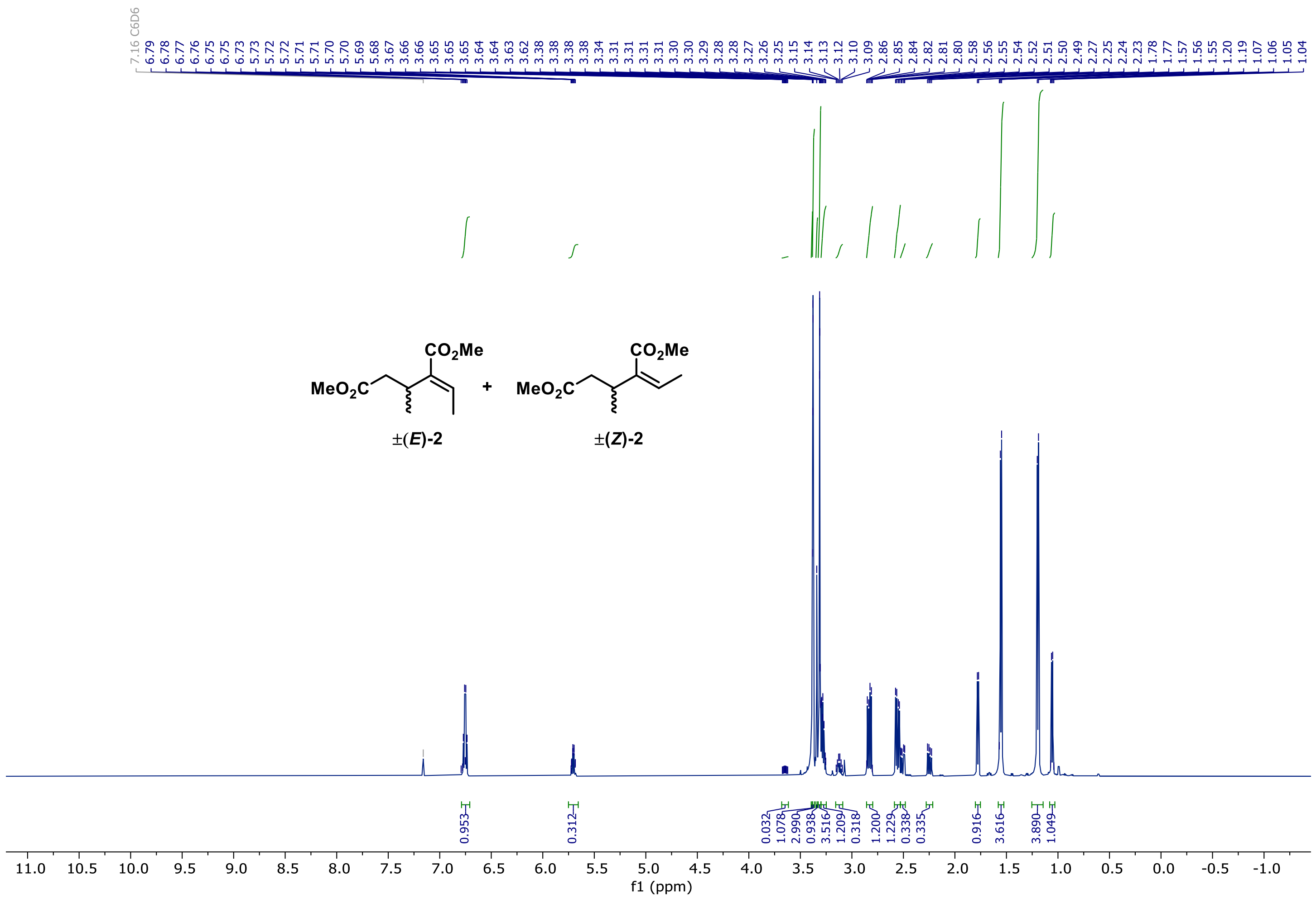

Supplementary Figure 33 – ^{1}H NMR (500 MHz, C_6D_6) spectrum of (*E*):(*Z*)-dimethyl 2-ethylidene-3-methylpentanedioate **2**.

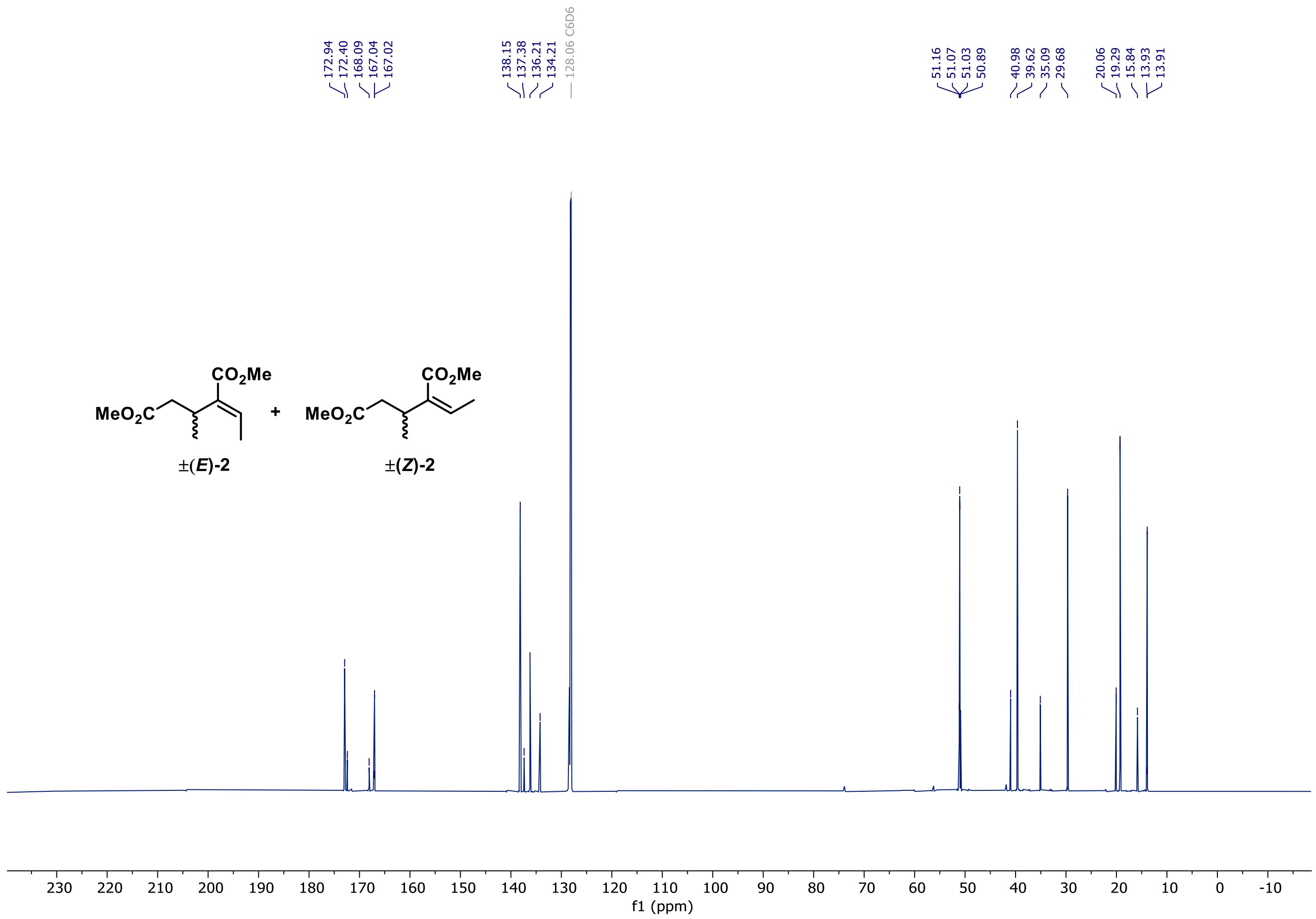

Supplementary Figure 34 – ^{13}C NMR (126 MHz, C_6D_6) spectrum of (*E*):(*Z*)-dimethyl 2-ethylidene-3-methylpentanedioate **2**.

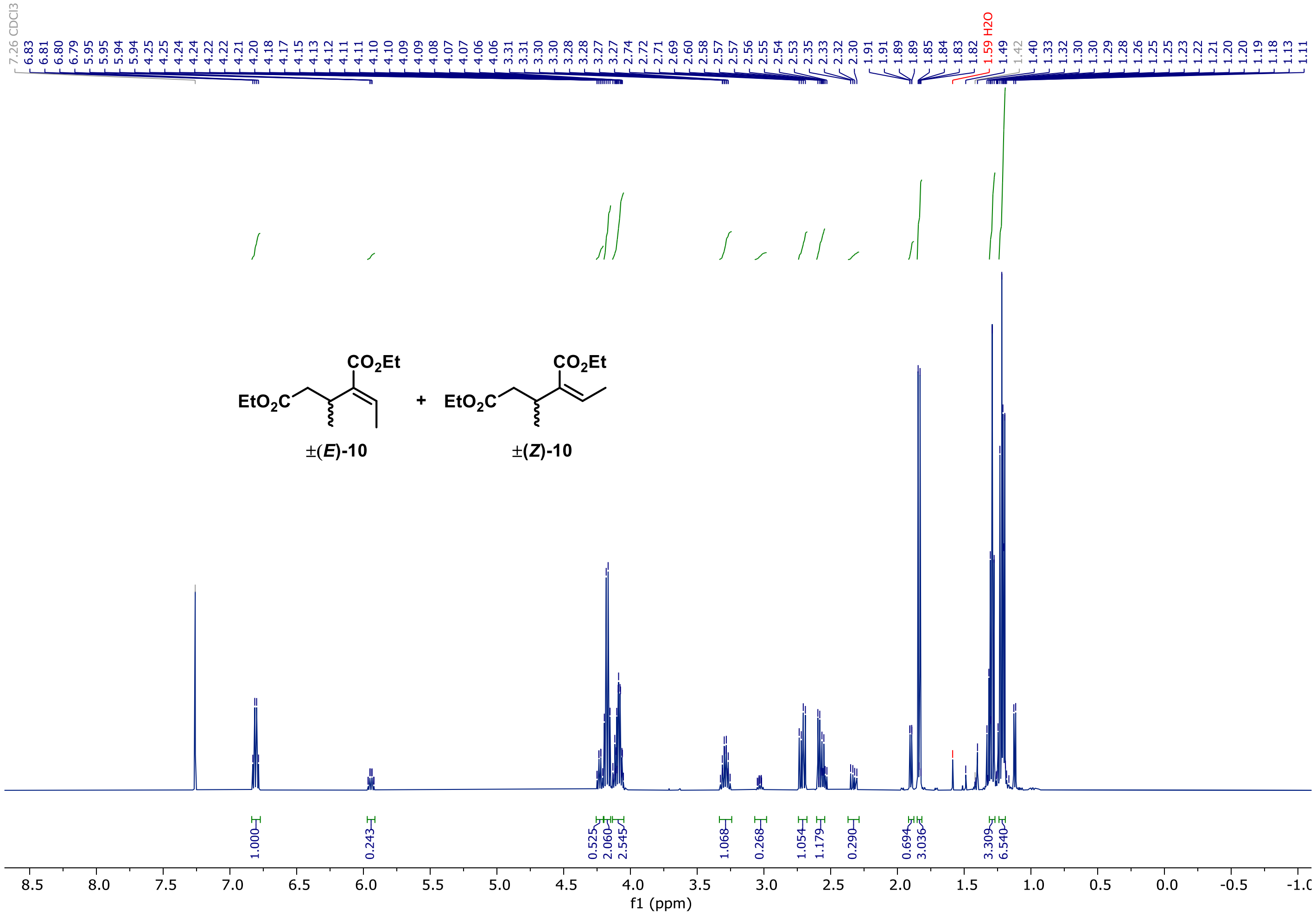

Supplementary Figure 35 – ^{1}H NMR (500 MHz, $CDCl_3$) spectrum of (*E*), (*Z*)- diethyl 2-ethylidene-3-methylglutarate **10**.

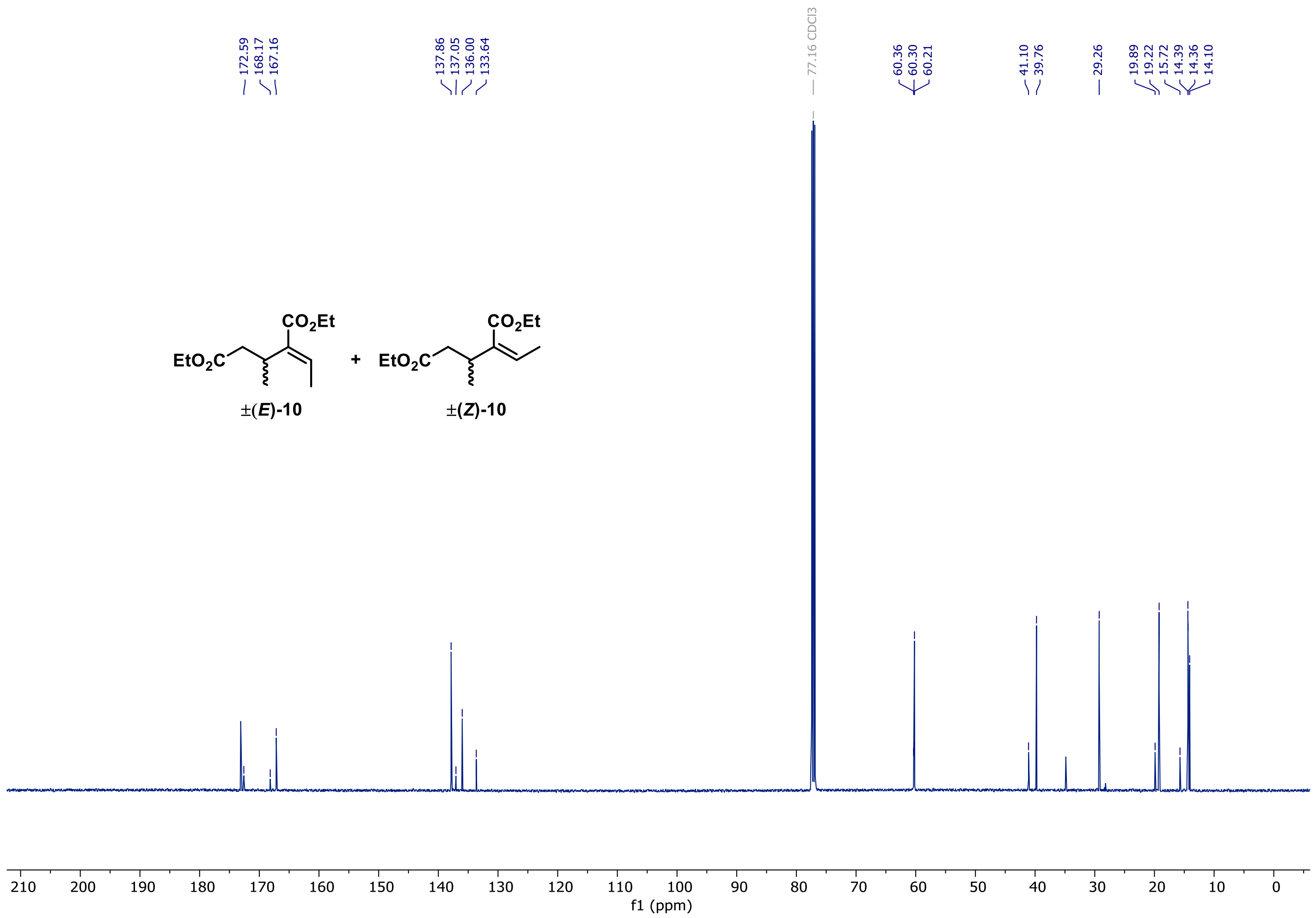

Supplementary Figure 36 – ^{13}C NMR (126 MHz, $CDCl_3$) spectrum of ^{1}H NMR spectrum of (*E*), (*Z*)- diethyl 2-ethylidene-3-methylglutarate **10**.

13

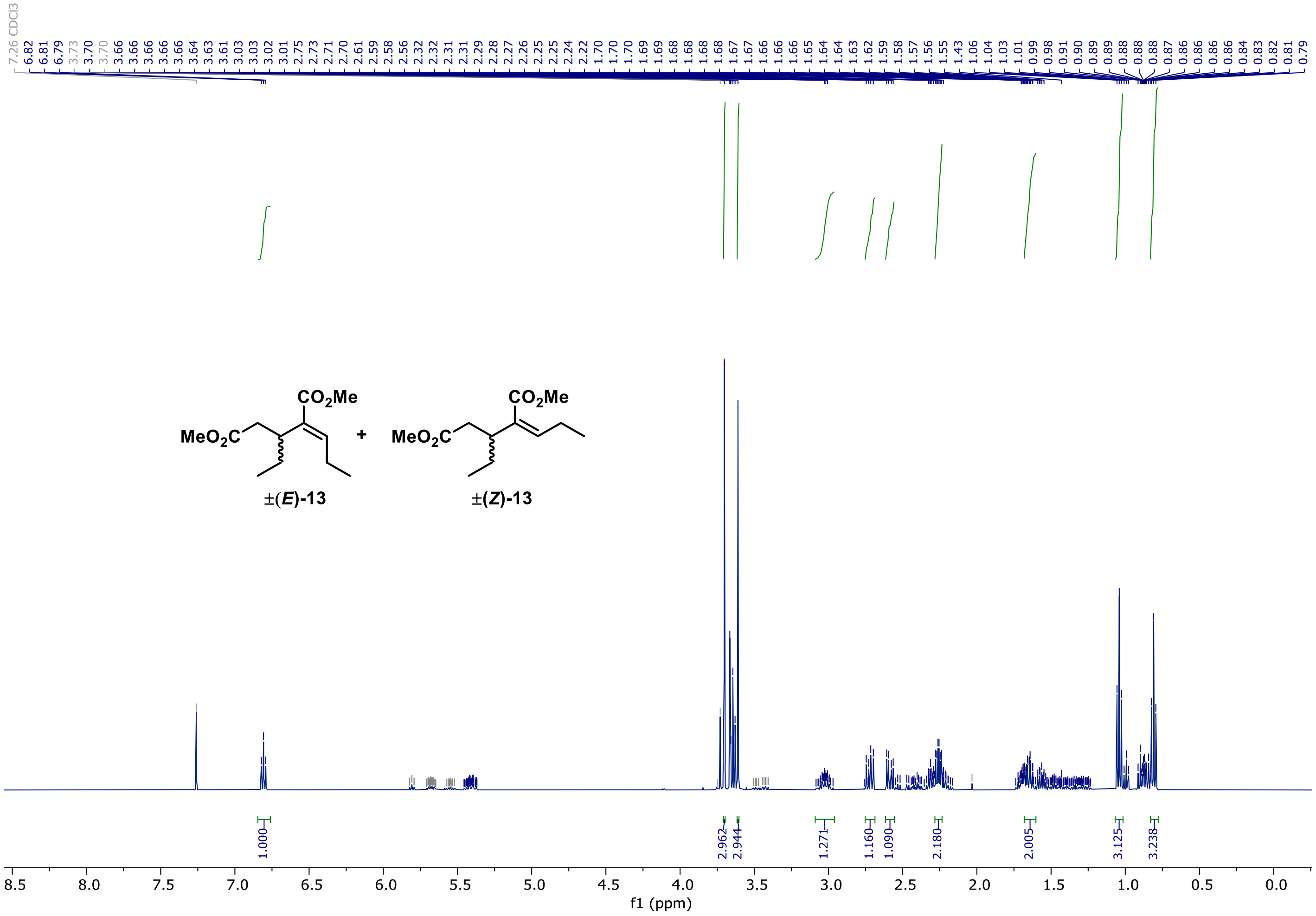

Supplementary Figure 37 – ^{1}H NMR (500 MHz, $CDCl_3$) spectrum of (*E*), (*Z*)-2-(1-propenyl)-3-ethylglutaric acid dimethyl ester **13**. Only the (*E*)-isomer is integrated; other peaks highlighted include (*Z*)- isomer and (*E*), (*Z*)- 2-(1-propenyl)-3-ethyl-glutaric acid dimethylester.

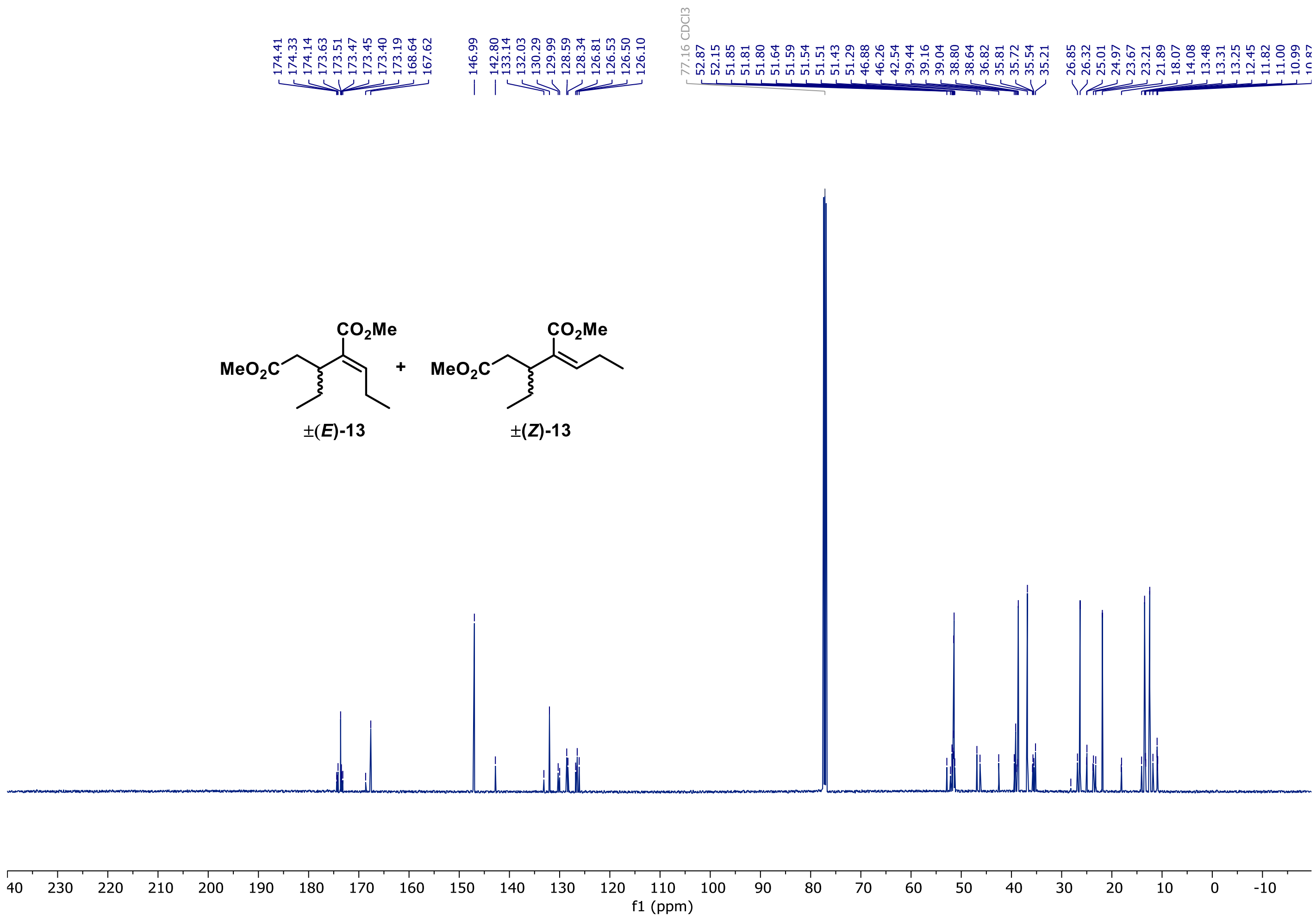

Supplementary Figure 38 – ^{13}C NMR (126 MHz, $CDCl_3$) spectrum of (*E*), (*Z*)-2-(1-propenyl)-3-ethylglutaric acid dimethyl ester **13**.

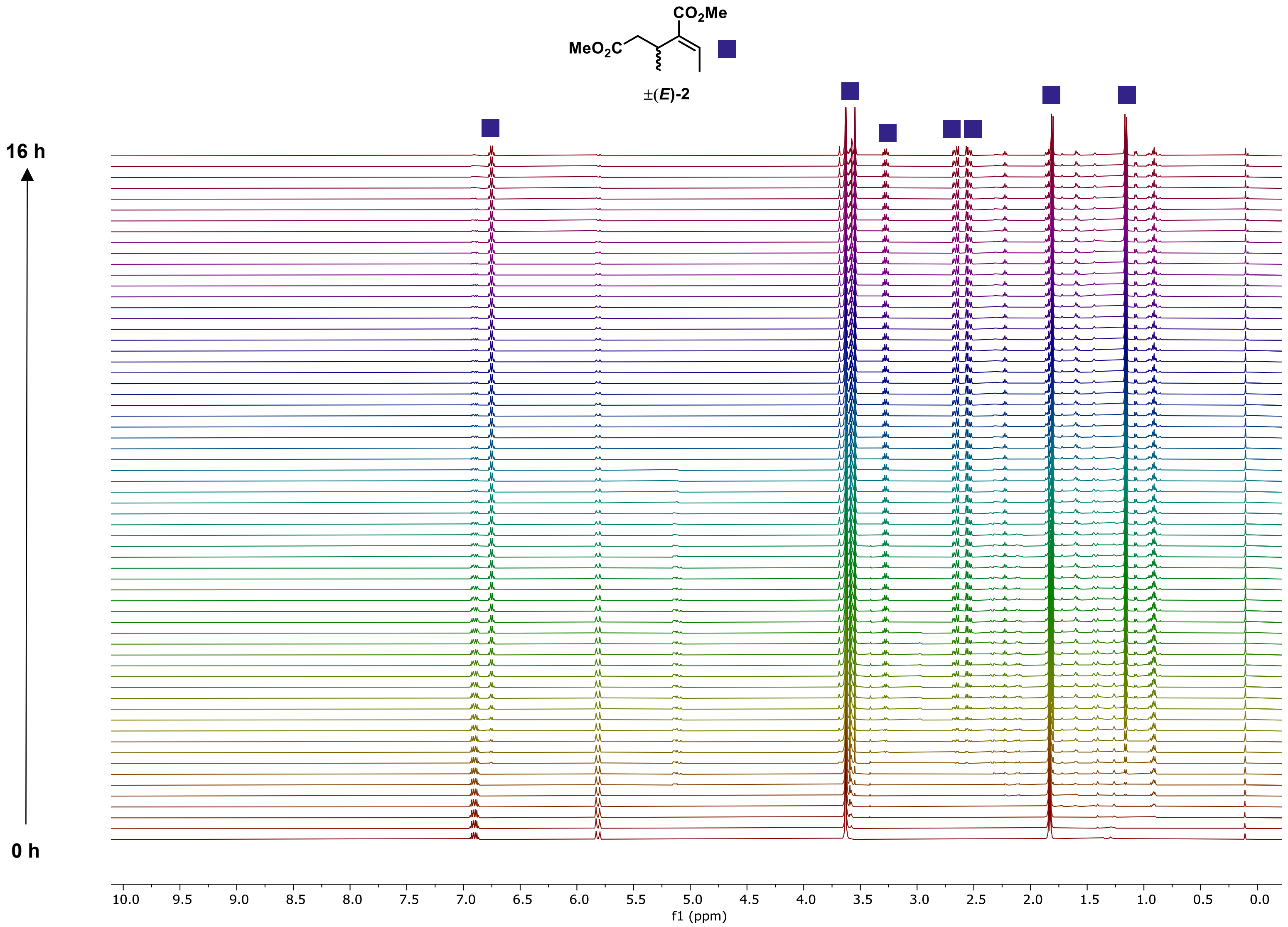

Supplementary Figure 39 – Stacked ^{1}H NMR (500 MHz, THF-d_8) spectra taken every 15 minutes of *in situ* monitoring under base-mediated *i.e.* dark conditions dimerising methyl crotonate **1** with [(dmpe)$_2$FeH$_2$] **3** (5 mol%) for 16 hours at 309 K.

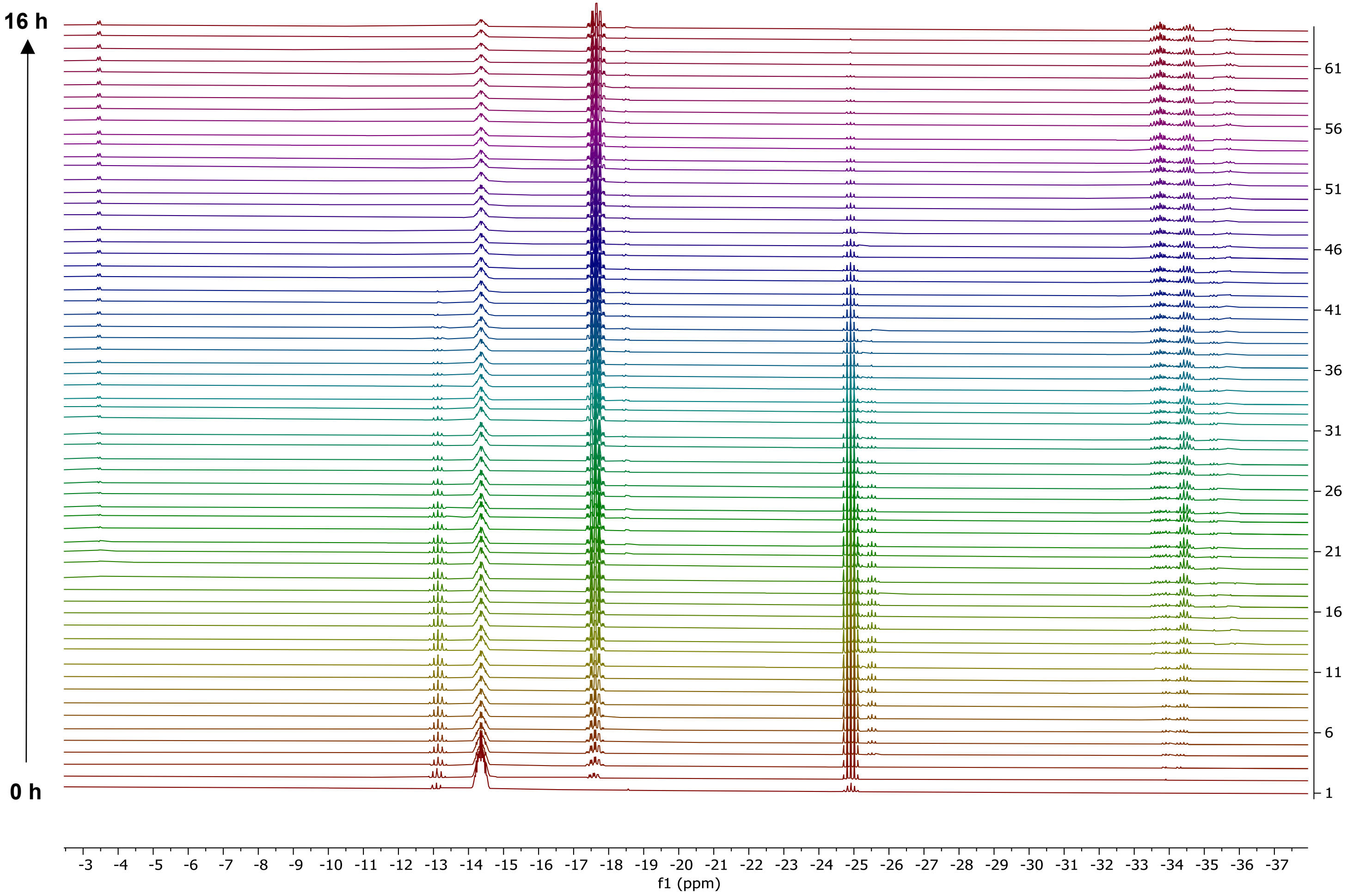

Supplementary Figure 40 – Stacked ^{1}H NMR (500 MHz, THF-d_8) spectra taken of the hydride region every 15 minutes of *in situ* monitoring under base-mediated *i.e.* dark conditions dimerising methyl crotonate **1** with [(dmpe)$_2$FeH$_2$] **3** (5 mol%) for 16 hours at 309 K.

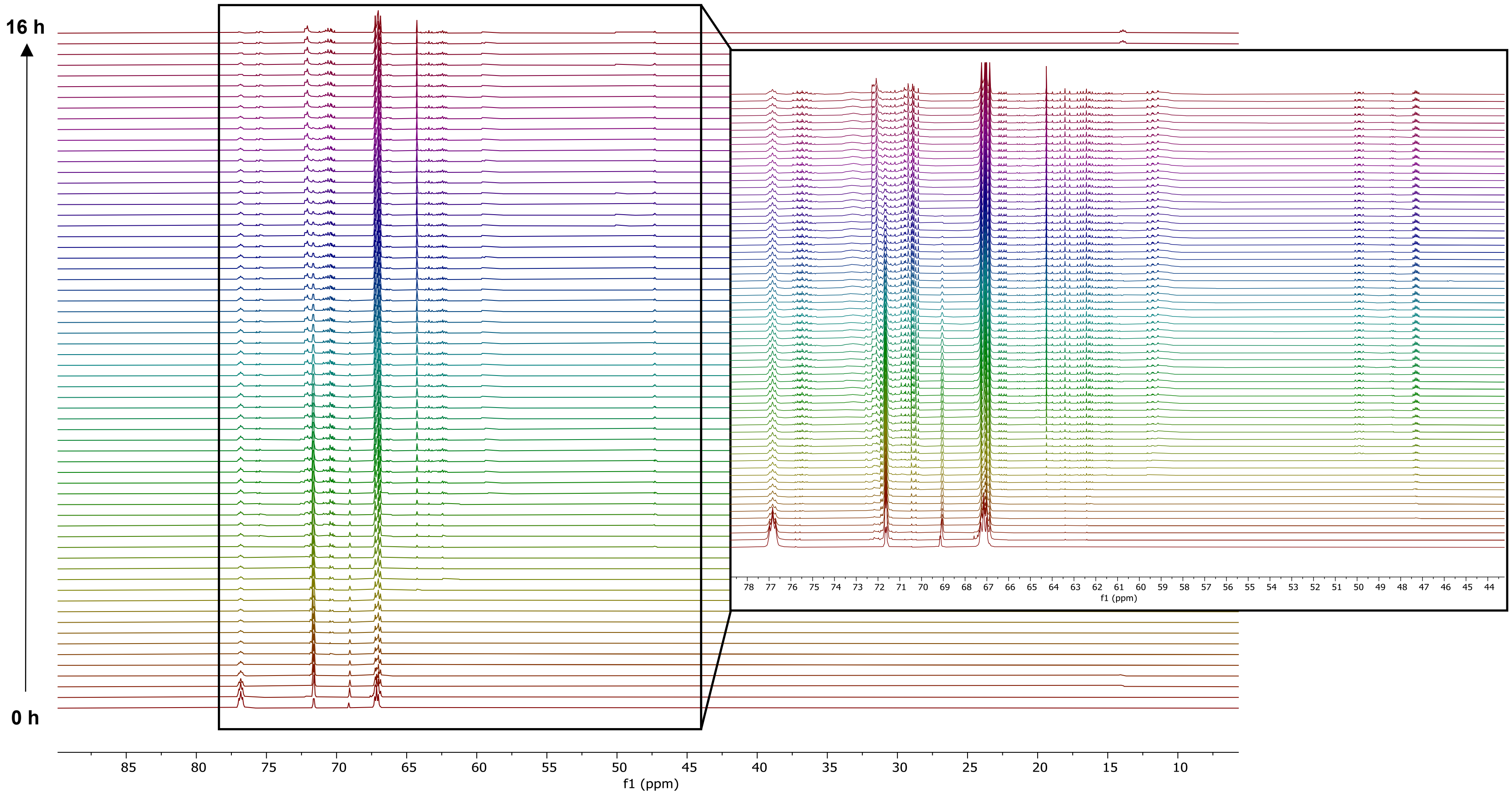

Supplementary Figure 41 – Stacked $^{31}P\{^{1}H\}$ NMR (202 MHz, THF-d_8) spectra taken every 15 minutes of *in situ* monitoring under base-mediated *i.e.* dark conditions dimerising methyl crotonate **1** with [(dmpe)$_2$FeH$_2$] **3** (5 mol%) for 16 hours at 309 K.

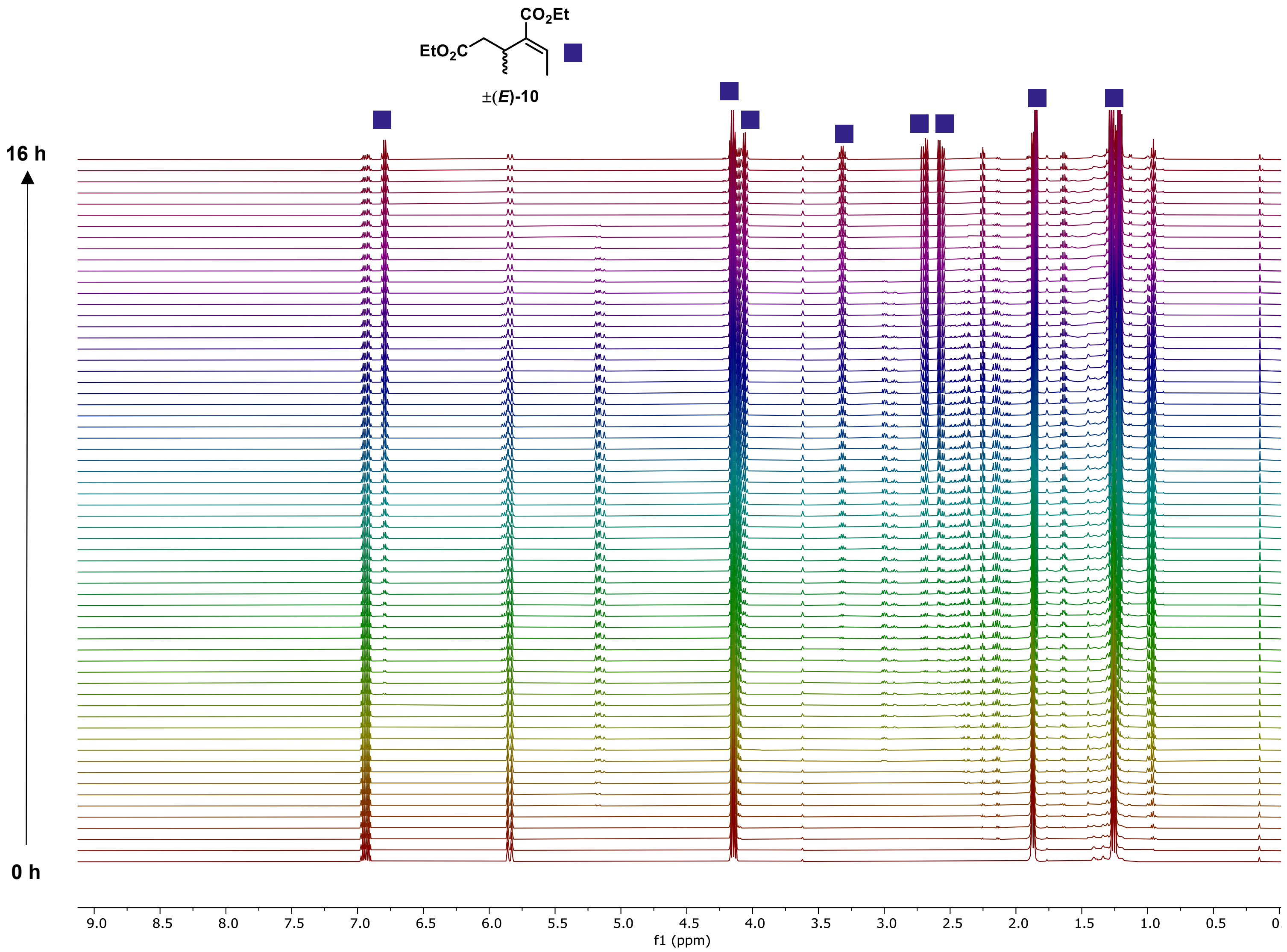

Supplementary Figure 42 – Stacked ^{1}H NMR (500 MHz, THF-d_8) spectra taken every 15 minutes of *in situ* monitoring under base-mediated *i.e.* dark conditions dimerising ethyl crotonate **9** with [(dmpe)$_2$FeH$_2$] **3** (5 mol%) for 16 hours at 309 K.

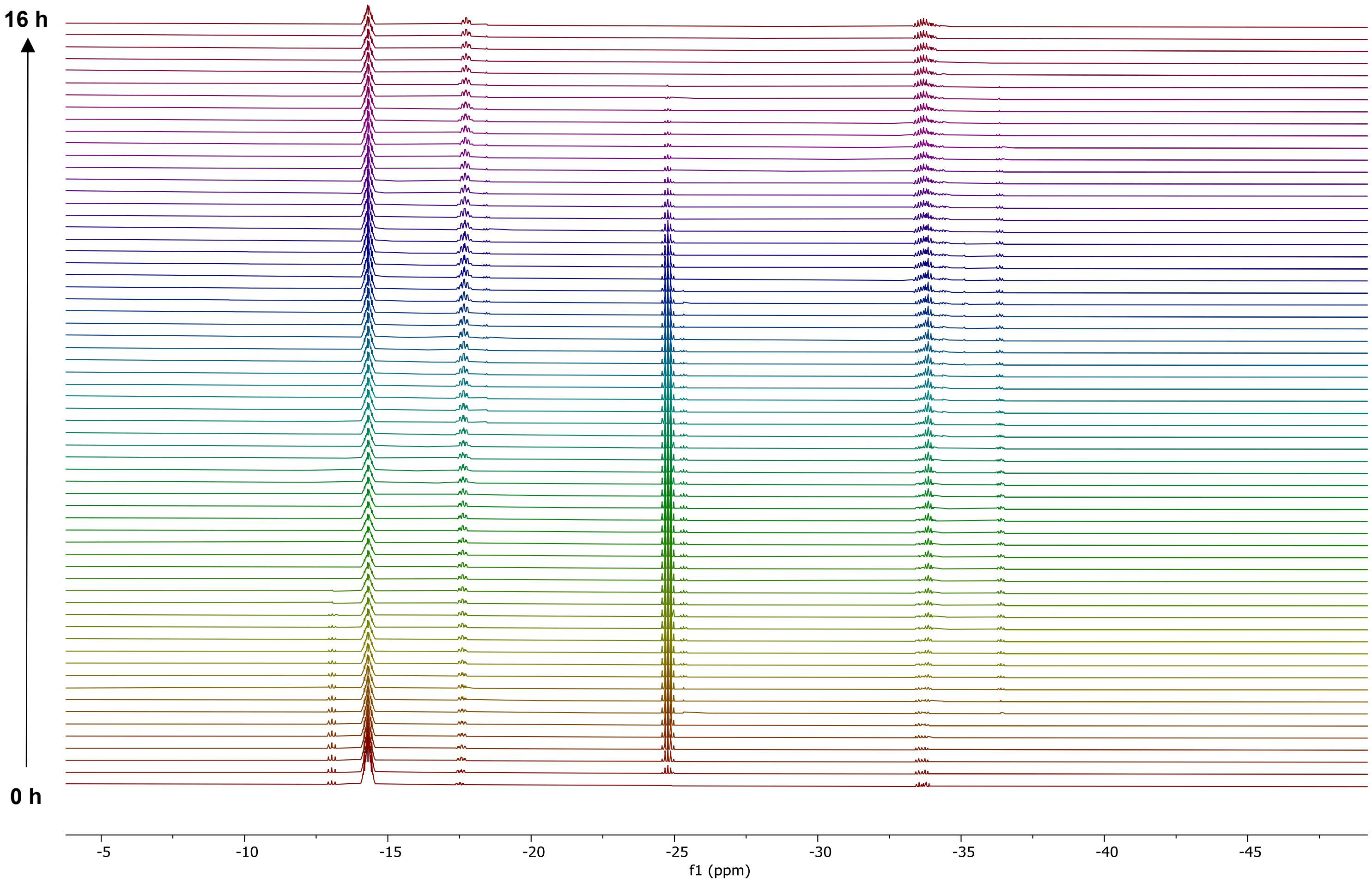

Supplementary Figure 43 – Stacked ^{1}H NMR (500 MHz, THF-d_8) spectra taken of the hydride region every 15 minutes of *in situ* monitoring under base-mediated *i.e.* dark conditions dimerising ethyl crotonate **9** with [$(dmpe)_2FeH_2$] **3** (5 mol%) for 16 hours at 309 K.

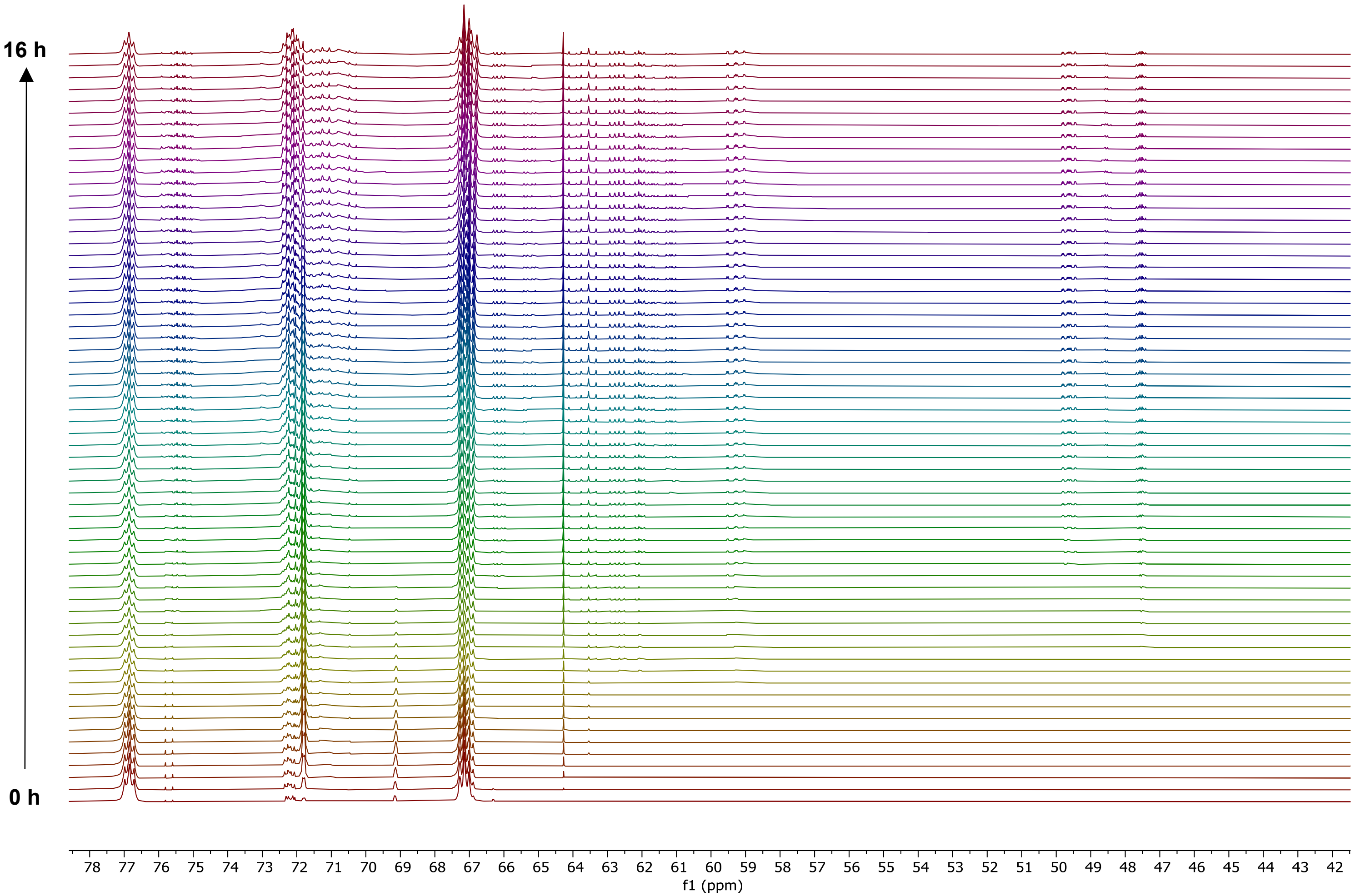

Supplementary Figure 44 – Stacked $^{31}P\{^{1}H\}$ NMR (202 MHz, THF-d_8) spectra taken every 15 minutes of *in situ* monitoring under base-mediated *i.e.* dark conditions dimerising ethyl crotonate **9** with $[(dmpe)_2FeH_2]$ **3** (5 mol%) for 16 hours at 309 K.

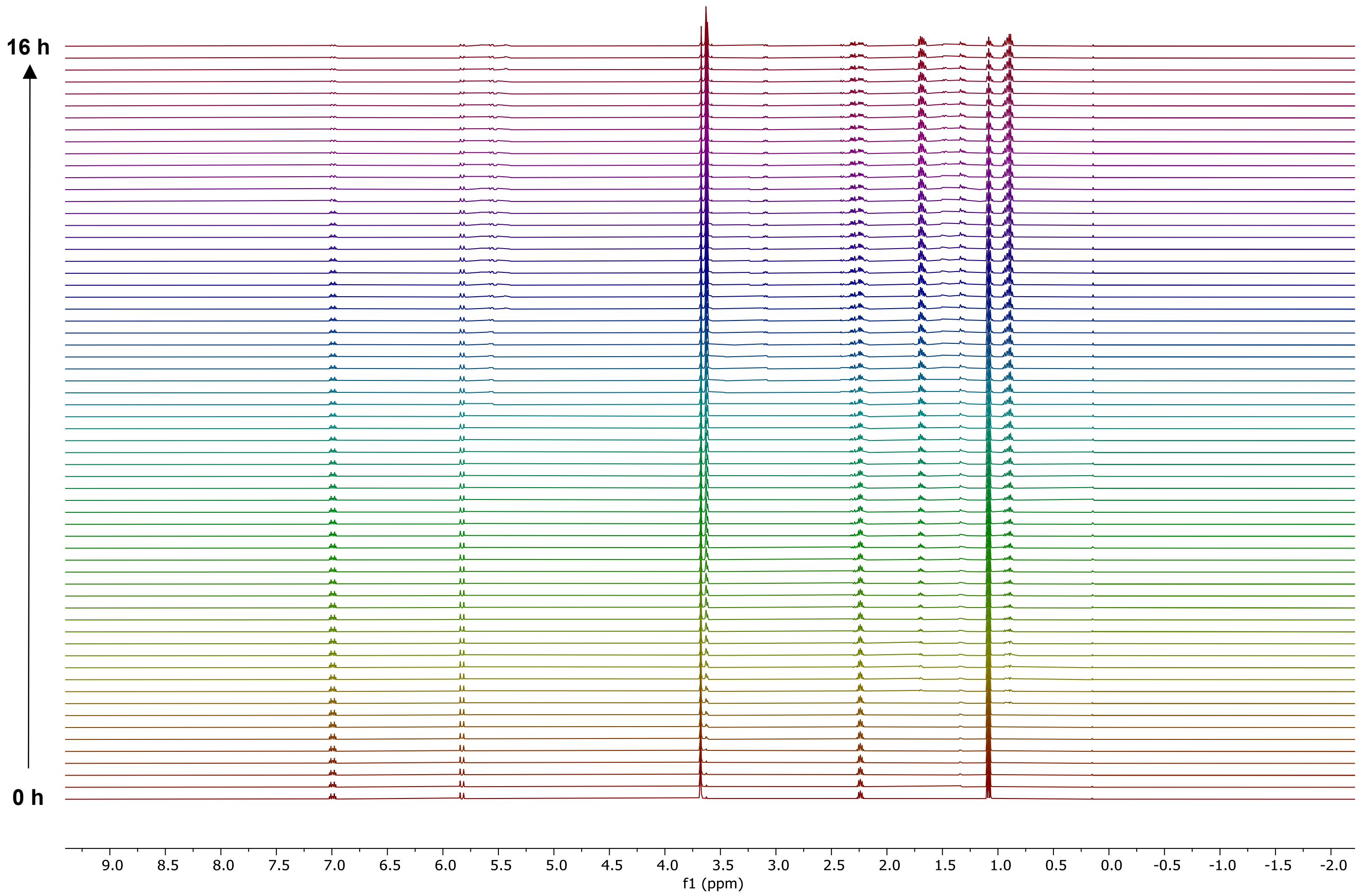

Supplementary Figure 45 – Stacked ^{1}H NMR (500 MHz, THF-d_8) spectra taken every 15 minutes of *in situ* monitoring under base-mediated *i.e.* dark conditions dimerising methyl (*E*)-2-pentenoate **11** with [(dmpe)$_2$FeH$_2$] **3** (5 mol%) for 16 hours at 309 K.

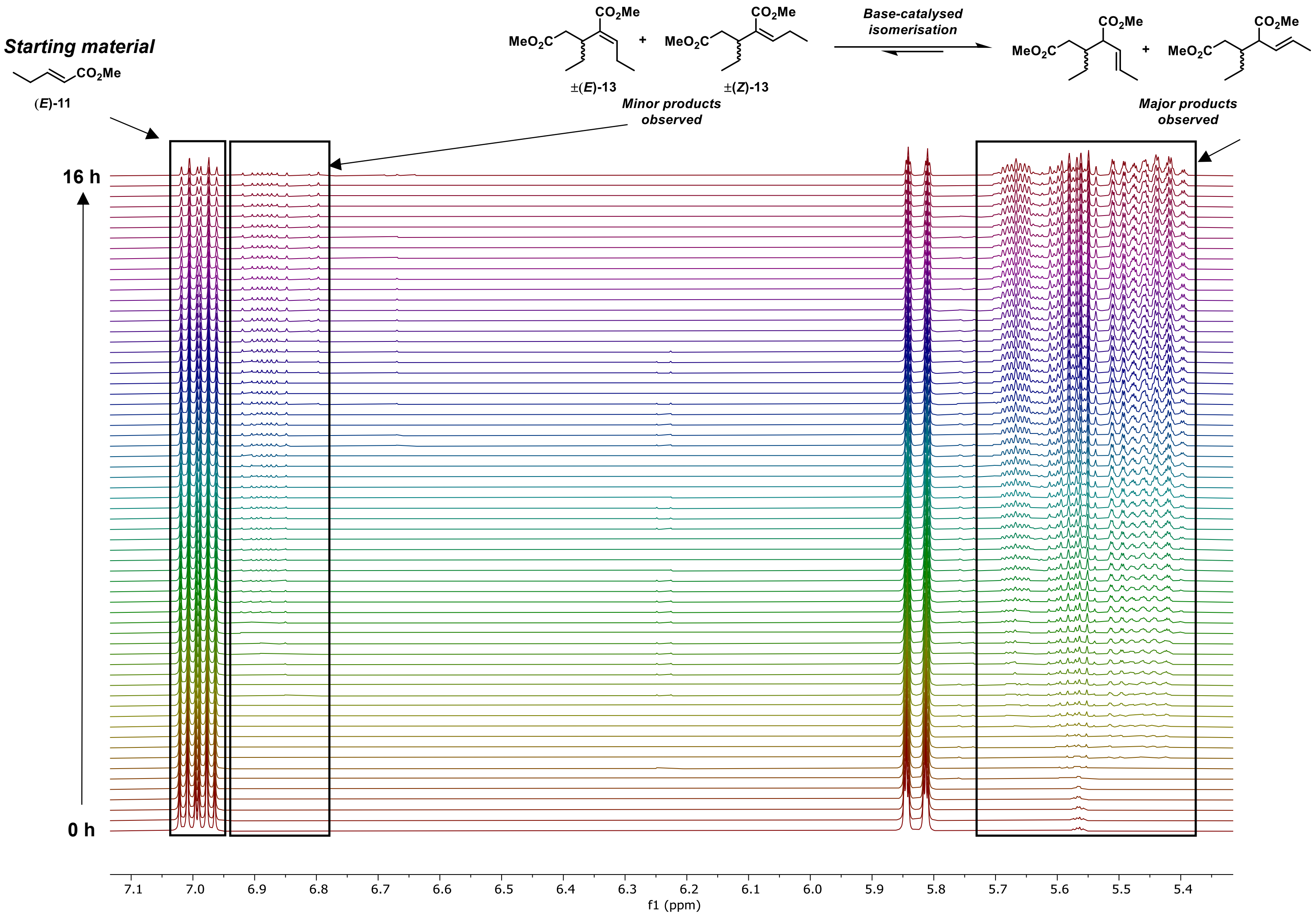

Supplementary Figure 46 – Stacked ^{1}H NMR (500 MHz, THF-d_8) spectra between δ 5.3− 7.1 ppm taken every 15 minutes of *in situ* monitoring under base-mediated *i.e.* dark conditions dimerising methyl (*E*)-2-pentenoate **11** with [(dmpe)$_2$FeH$_2$] **3** (5 mol%) for 16 hours at 309 K.

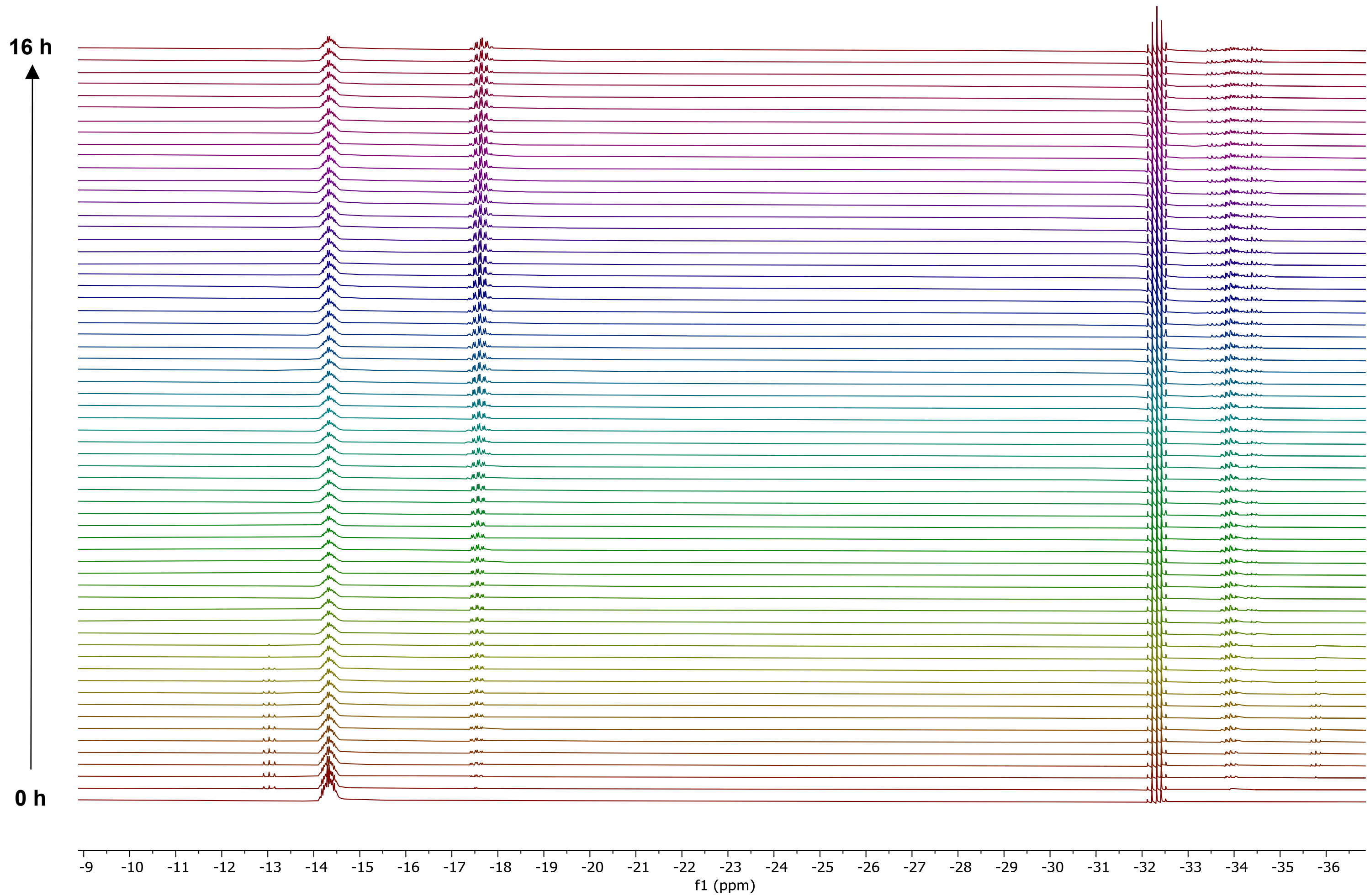

Supplementary Figure 47 – Stacked ^{1}H NMR (500 MHz, THF-d_8) spectra of the hydride region taken every 15 minutes of *in situ* monitoring under base-mediated *i.e.* dark conditions dimerising methyl (*E*)-2-pentenoate **11** with [$(dmpe)_2FeH_2$] **3** (5 mol%) for 16 hours at 309 K.

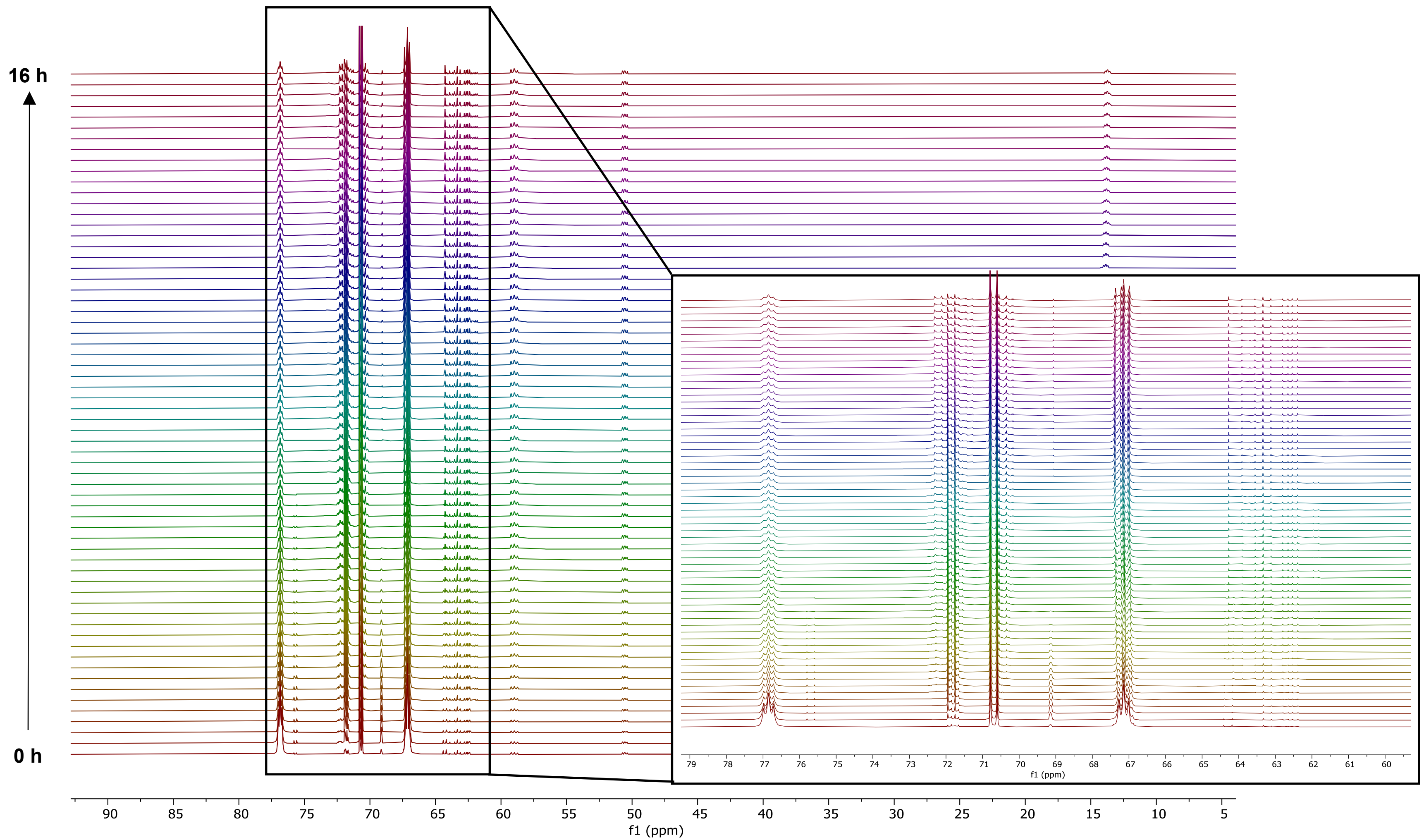

Supplementary Figure 48 – $^{31}P\{^1H\}$ (202 MHz, THF-d_8) stacked spectra of methyl (*E*)-2-pentenoate **11** dimerisation by $[(dmpe)_2FeH_2]$ **3** (5 mol%) taken every 15 minutes of *in situ* monitoring under base-mediated *i.e.* dark conditions for 16 hours at 309 K.

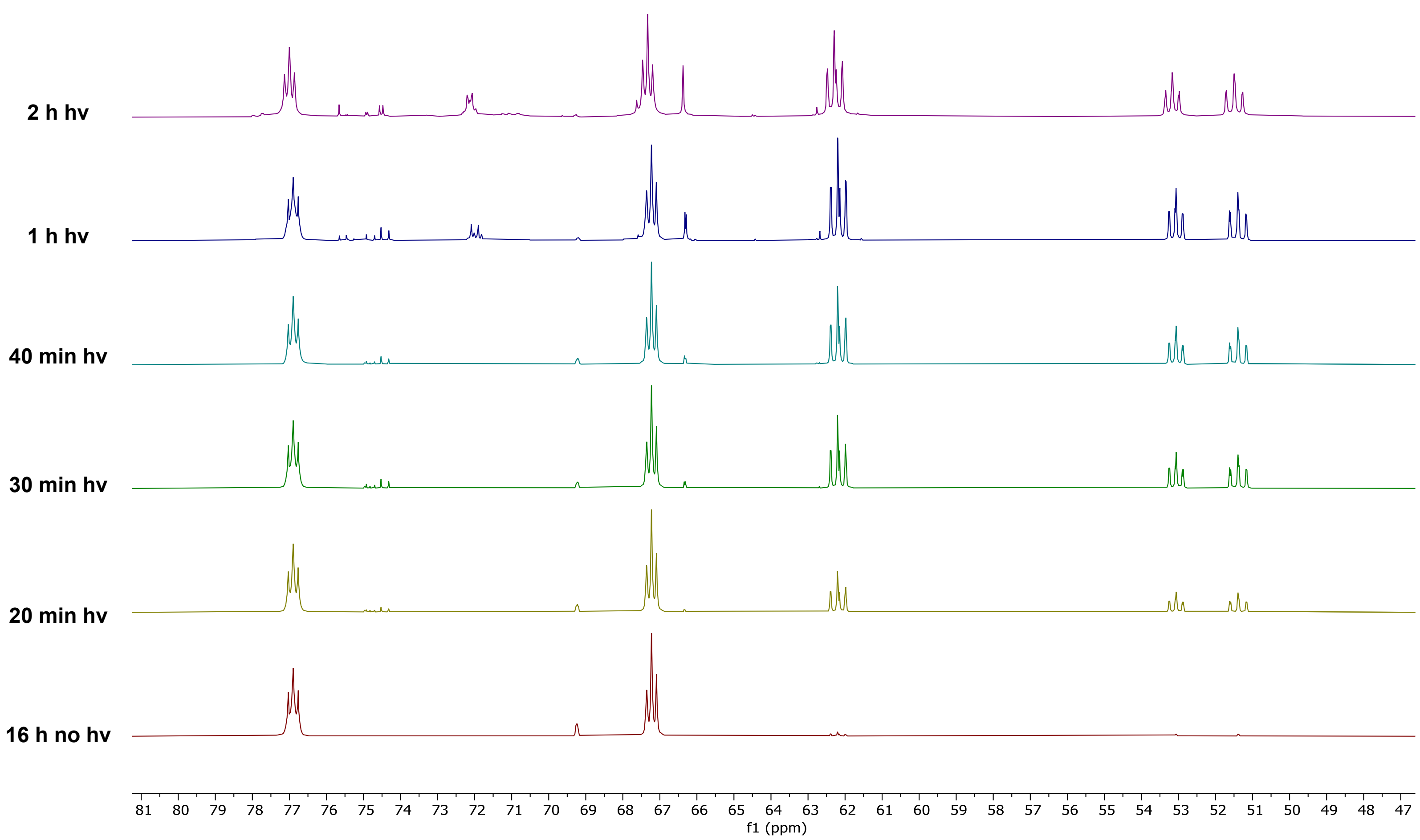

Supplementary Figure 49 – $^{31}P\{^{1}H\}$ (202 MHz, THF-*d*$_8$) stacked spectra of attempted methyl cinnamate **12** dimerisation by [(dmpe)$_2$FeH$_2$] **3** (5 mol%) after 16 hours under no-light conditions and then 2 hours of UV (365 nm) irradiation, forming [(dmpe)$_2$Fe(PhCH=CHCO$_2$Me)] **15** which was subsequently isolated by X-ray crystallography.

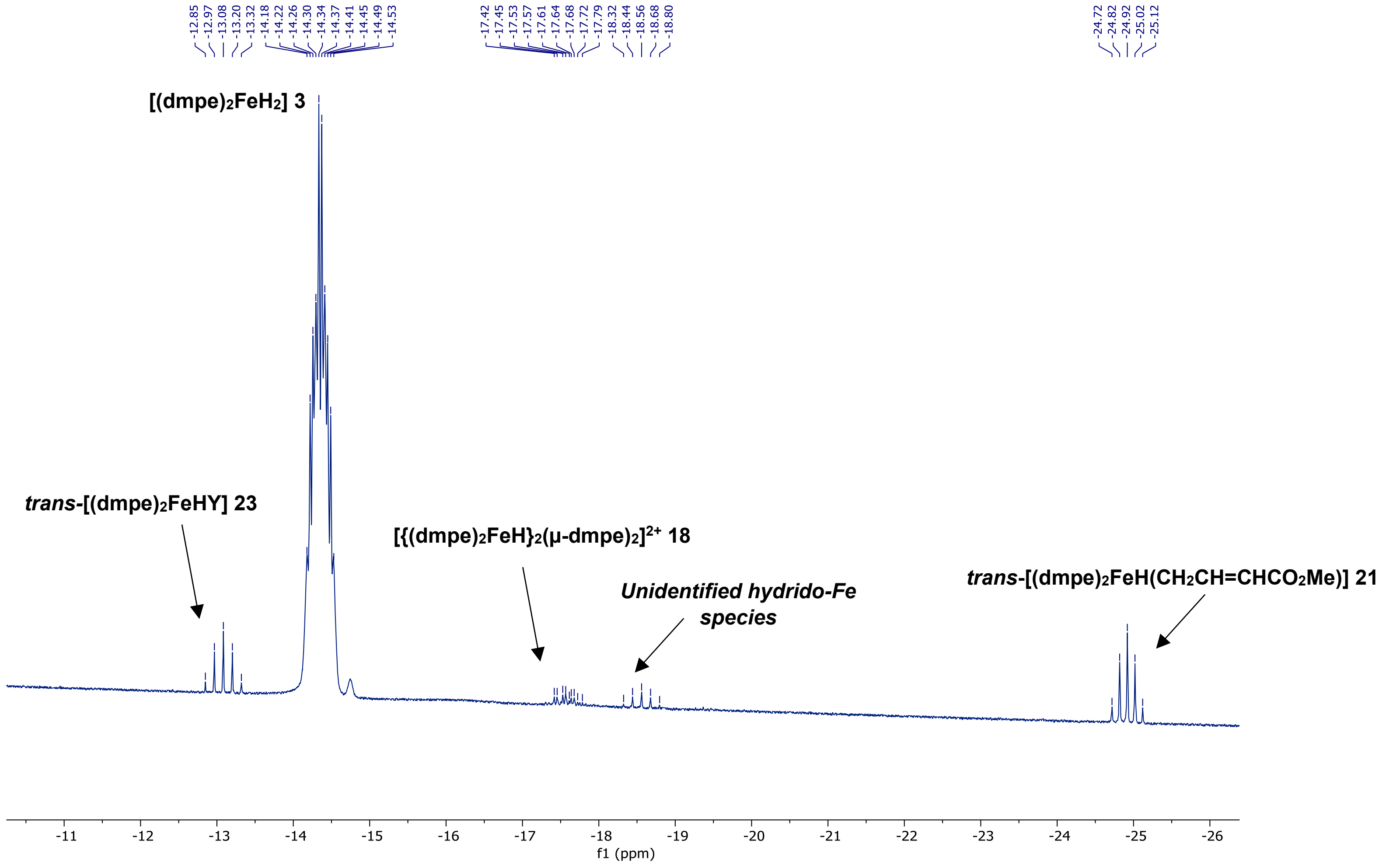

Supplementary Figure 50 – ^{1}H NMR (500 MHz, THF-d_8) spectrum *(only hydride region shown)* at the start of *in situ* monitoring under base-mediated *i.e.* dark conditions dimerising methyl crotonate **1** with $[(dmpe)_2FeH_2]$ **3** (5 mol%) for 16 hours at 309 K.

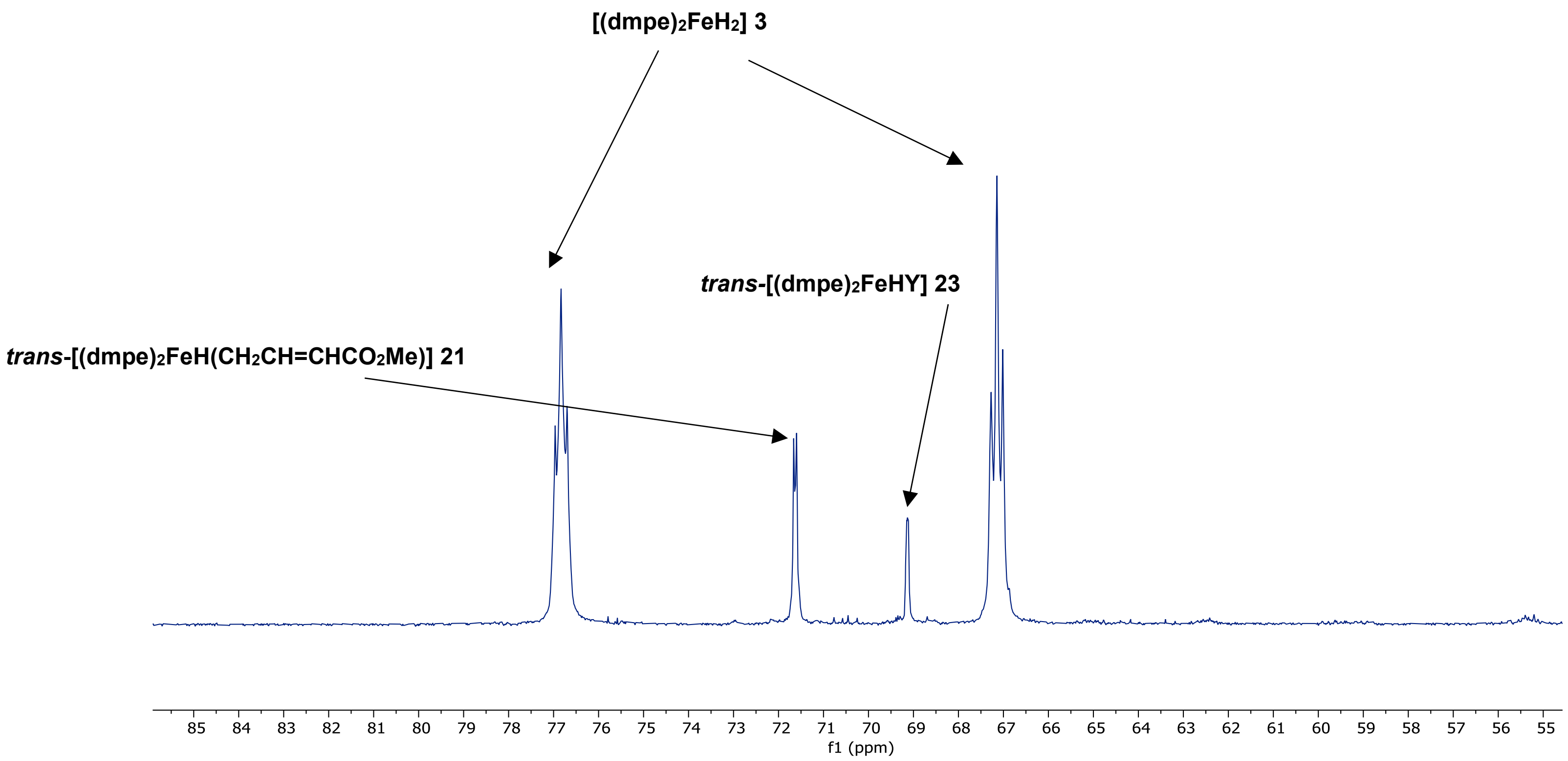

Supplementary Figure 51 – $^{31}P\{^1H\}$ NMR (202 MHz, THF-d_8) spectrum at the start of *in situ* monitoring under base-mediated *i.e.* dark conditions dimerising methyl crotonate **1** with $[(dmpe)_2FeH_2]$ **3** (5 mol%) for 16 hours at 309 K.

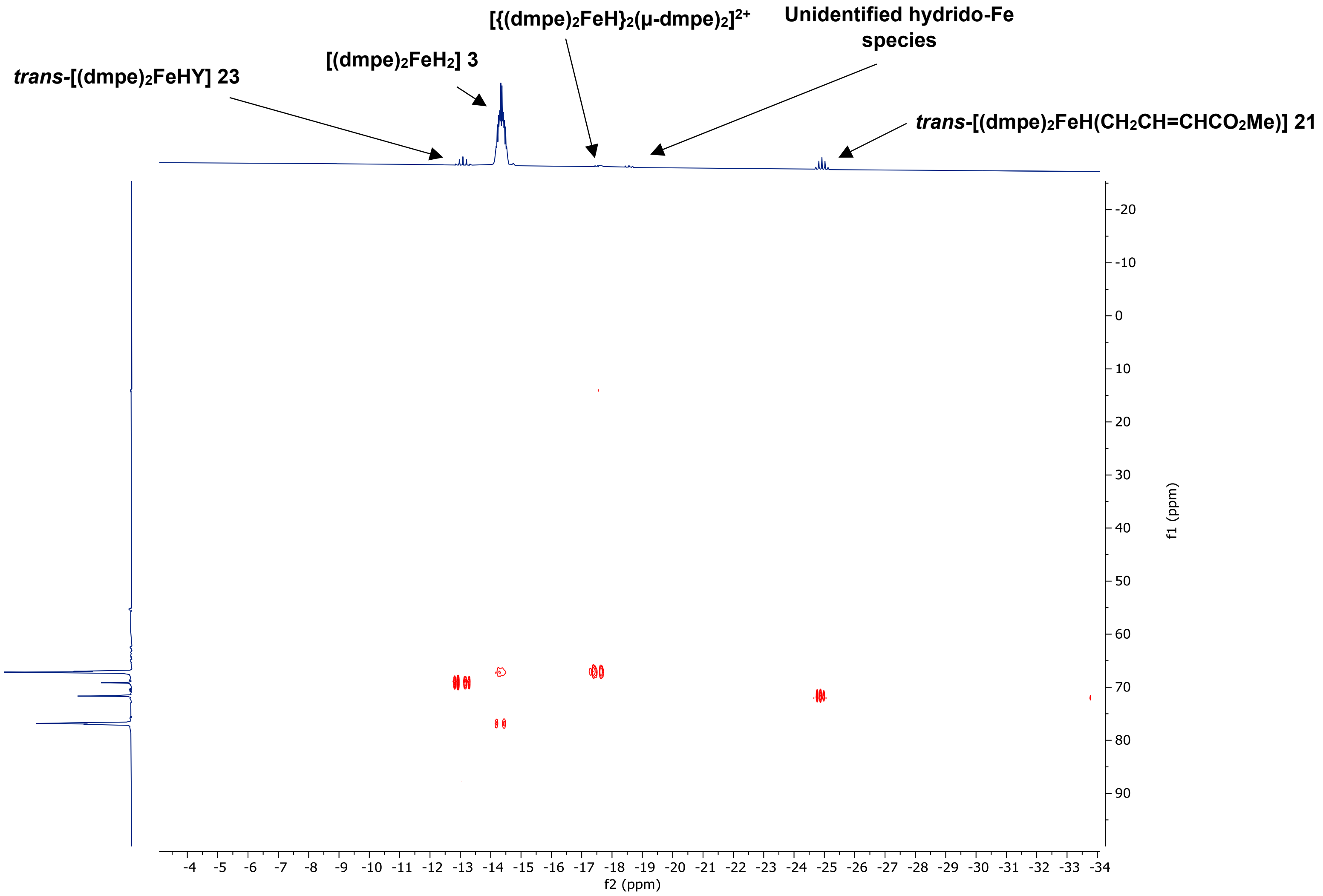

Supplementary Figure 52 – ^{1}H–^{31}P HMBC showing ^{1}H (*x*-axis) and ^{31}P (*y*-axis) NMR spectra at the start of *in situ* monitoring under base-mediated *i.e.* dark conditions dimerising methyl crotonate **1** with [(dmpe)$_2$FeH$_2$] **3** (5 mol%) for 16 hours at 309 K.

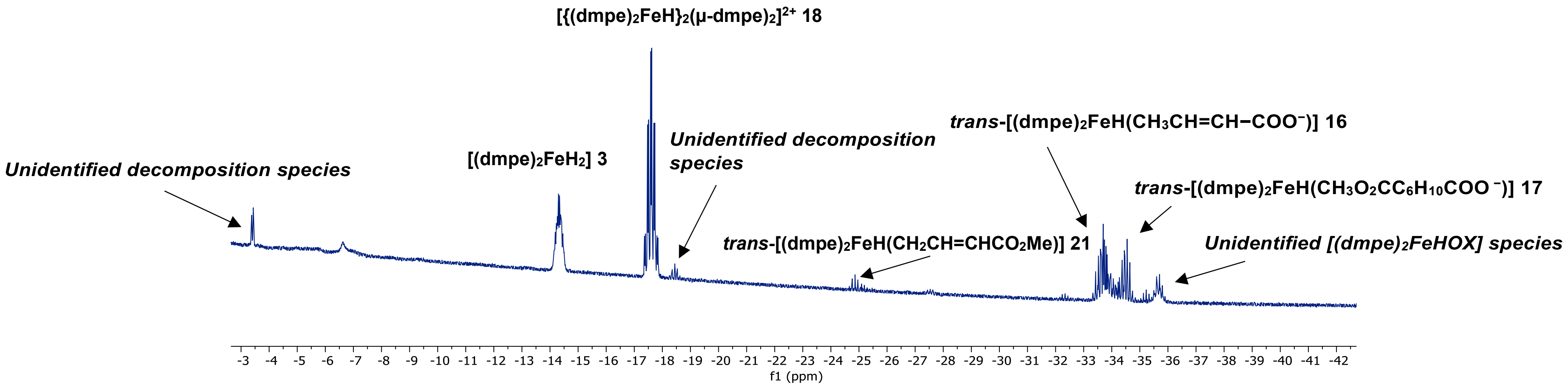

Supplementary Figure 53 – ^{1}H NMR (500 MHz, THF-d_8) spectrum (*hydride region only*) at the end of *in situ* monitoring under base-mediated *i.e.* dark conditions dimerising methyl crotonate **1** with [(dmpe)$_2$FeH$_2$] **3** (5 mol%) for 16 hours at 309 K.

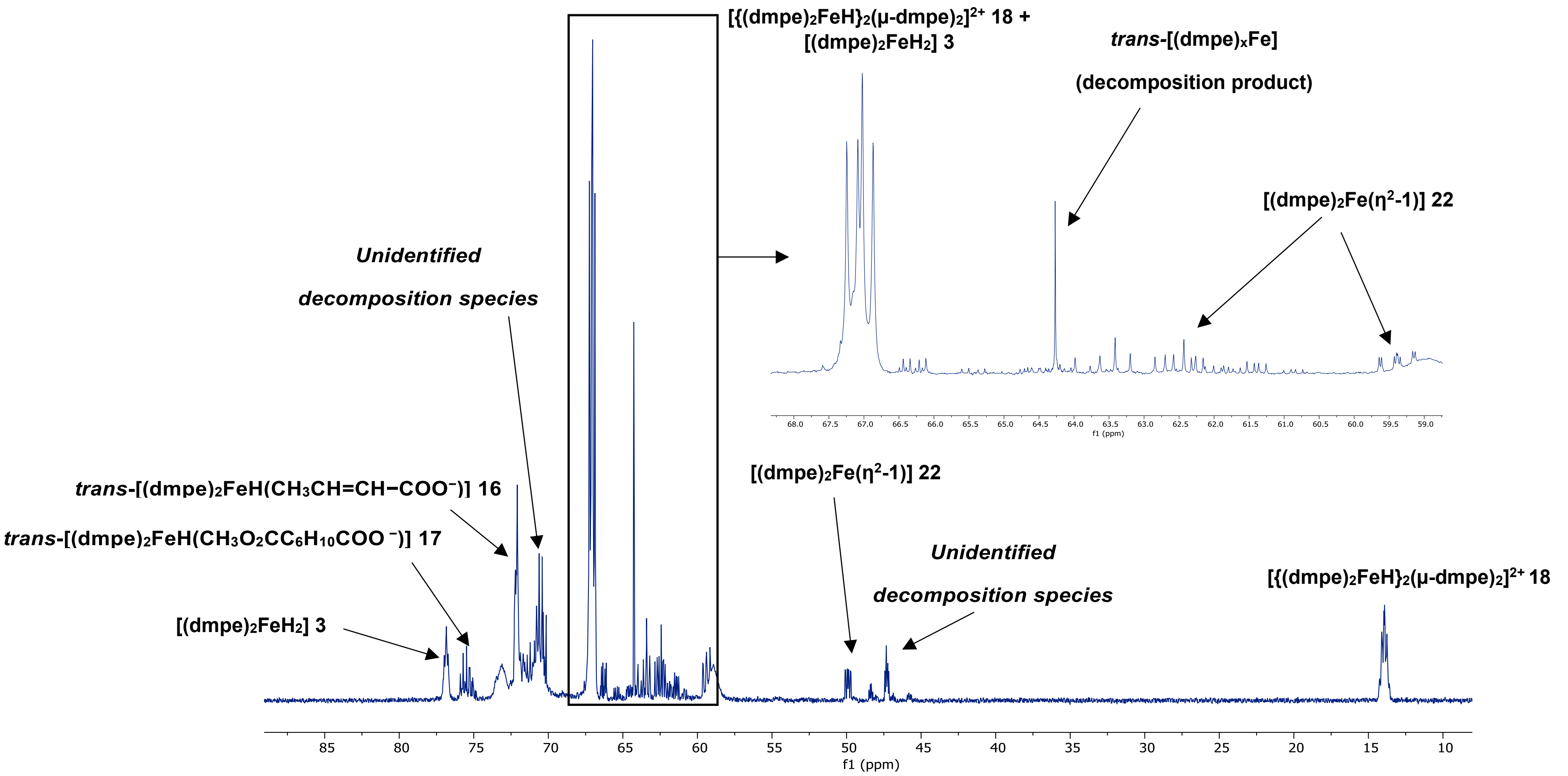

Supplementary Figure 54 – $^{31}P\{^{1}H\}$ NMR (202 MHz, THF-*d*$_8$) spectrum at the end of *in situ* monitoring under base-mediated *i.e.* dark conditions dimerising methyl crotonate **1** with [(dmpe)$_2$FeH$_2$] **3** (5 mol%) for 16 hours at 309 K.

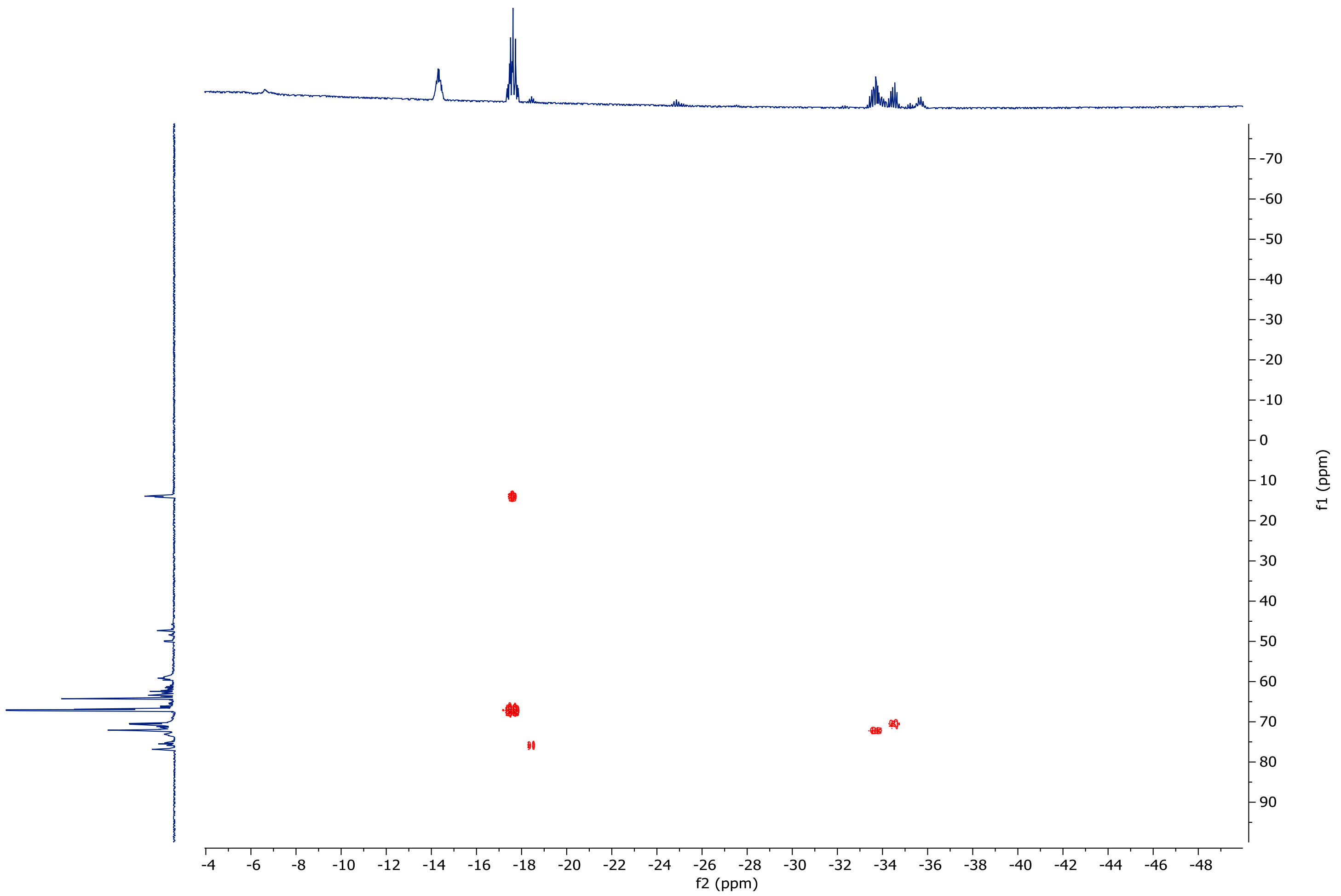

Supplementary Figure 55 – ^{1}H–^{31}P HMBC showing ^{1}H (x-axis) and $^{31}P\{^{1}H\}$ (y-axis) NMR spectra at the end of *in situ* monitoring under base-mediated *i.e.* dark conditions dimerising methyl crotonate **1** with $[(dmpe)_2FeH_2]$ **3** (5 mol%) for 16 hours at 309 K.

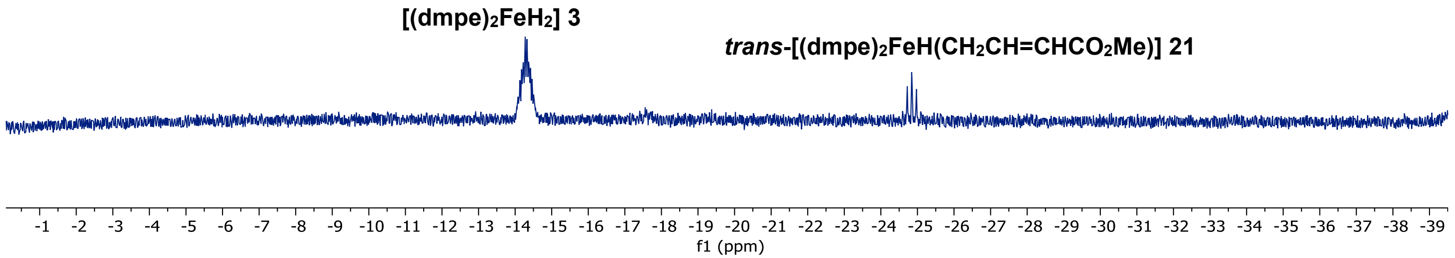

Supplementary Figure 56 – ^{1}H NMR (500 MHz, THF-d_8) spectrum (hydride region only) showing *in situ* monitoring under light-mediated conditions dimerising methyl crotonate **1** with [(dmpe)$_2$FeH$_2$] **3** (5 mol%) for approximately 2 hours at 300 K.

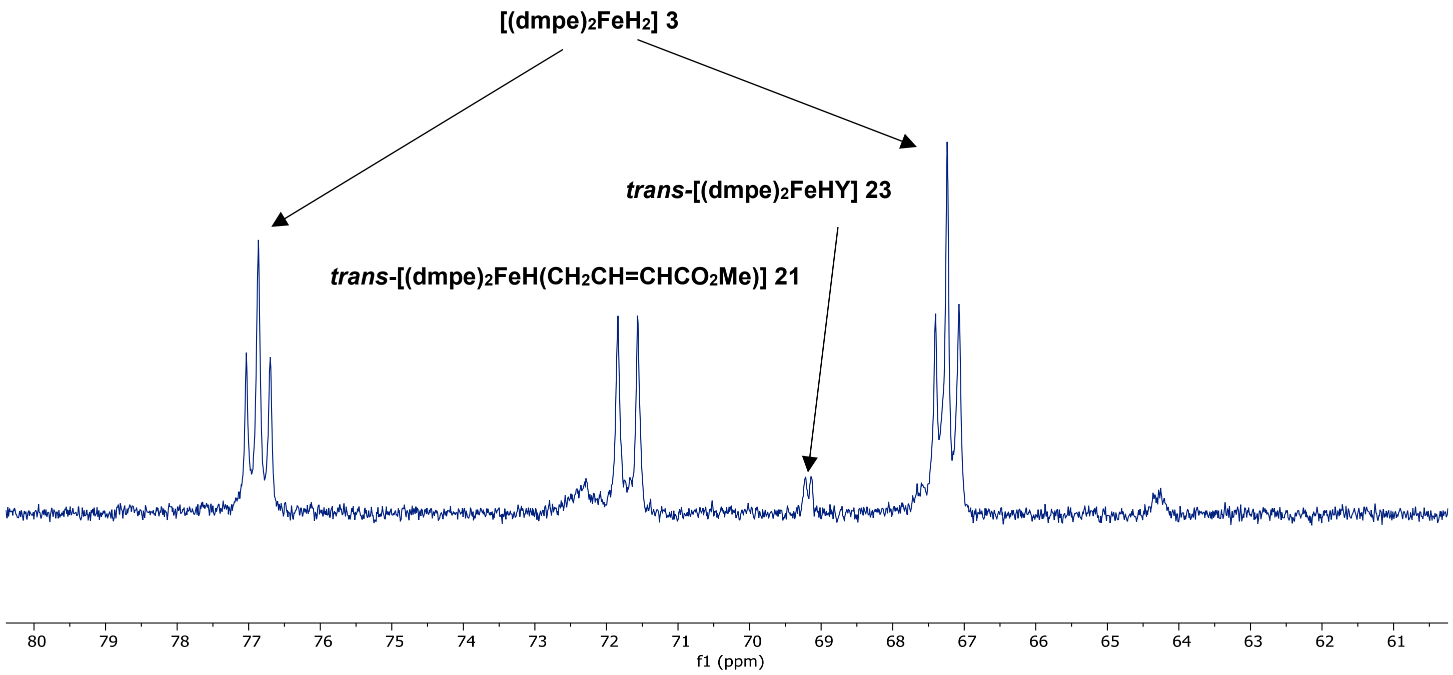

Supplementary Figure 57 – $^{31}P\{^{1}H\}$ NMR (202MHz, THF-d_8) spectrum showing *in situ* monitoring under light-mediated conditions dimerising methyl crotonate **1** with [(dmpe)$_2$FeH$_2$] **3** (5 mol%) for approximately 2 hours at 300 K.

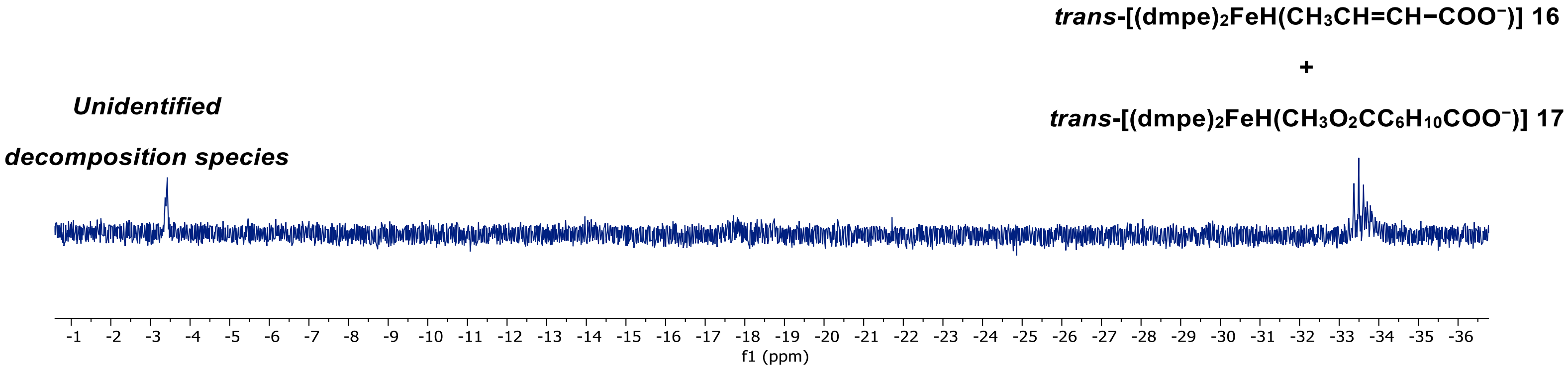

Supplementary Figure 58 – ^{1}H NMR (500MHz, THF-d_8) spectrum (hydride region only) showing end of *in situ* monitoring under light-mediated conditions dimerising methyl crotonate **1** with [(dmpe)$_2$FeH$_2$] **3** (5 mol%) for approximately 2 hours at 300 K.

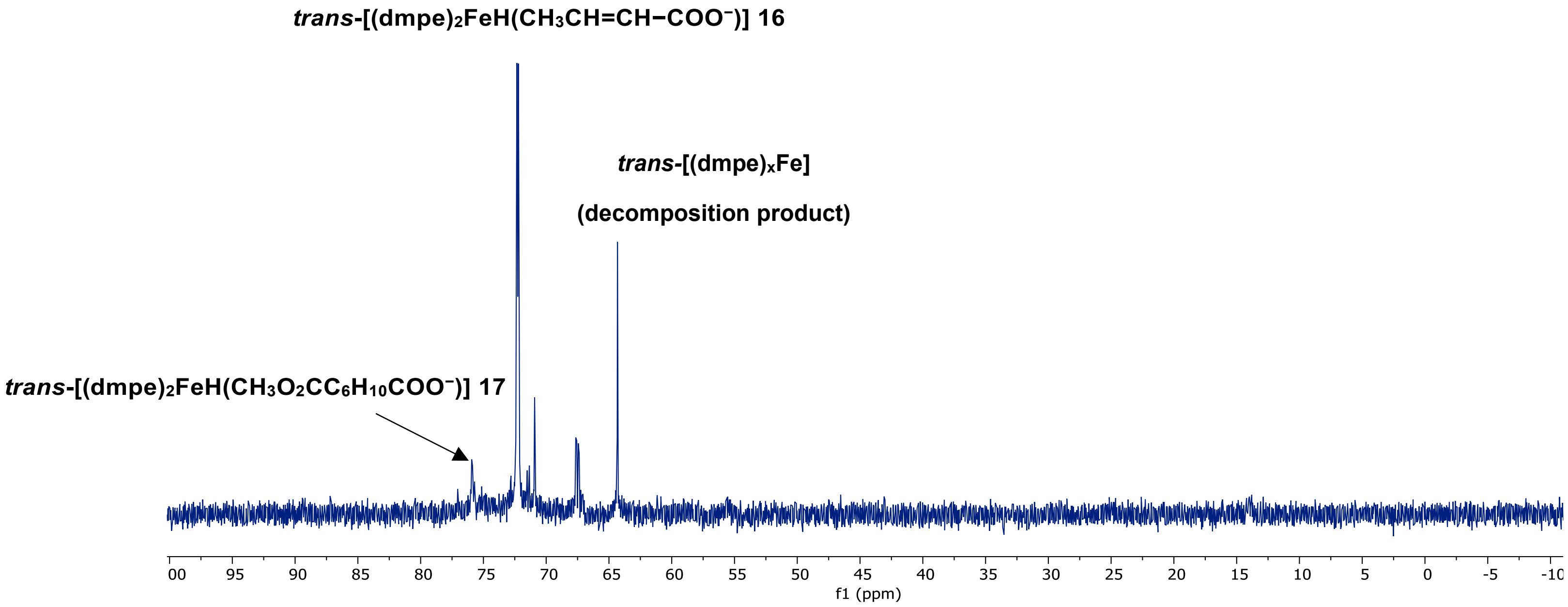

Supplementary Figure 59 – ^{31}P{^{1}H} NMR (202 MHz, THF-d_8) spectrum showing end of *in situ* monitoring under light-mediated conditions dimerising methyl crotonate **1** with [(dmpe)$_2$FeH$_2$] **3** (5 mol%) for approximately 2 hours at 300 K.

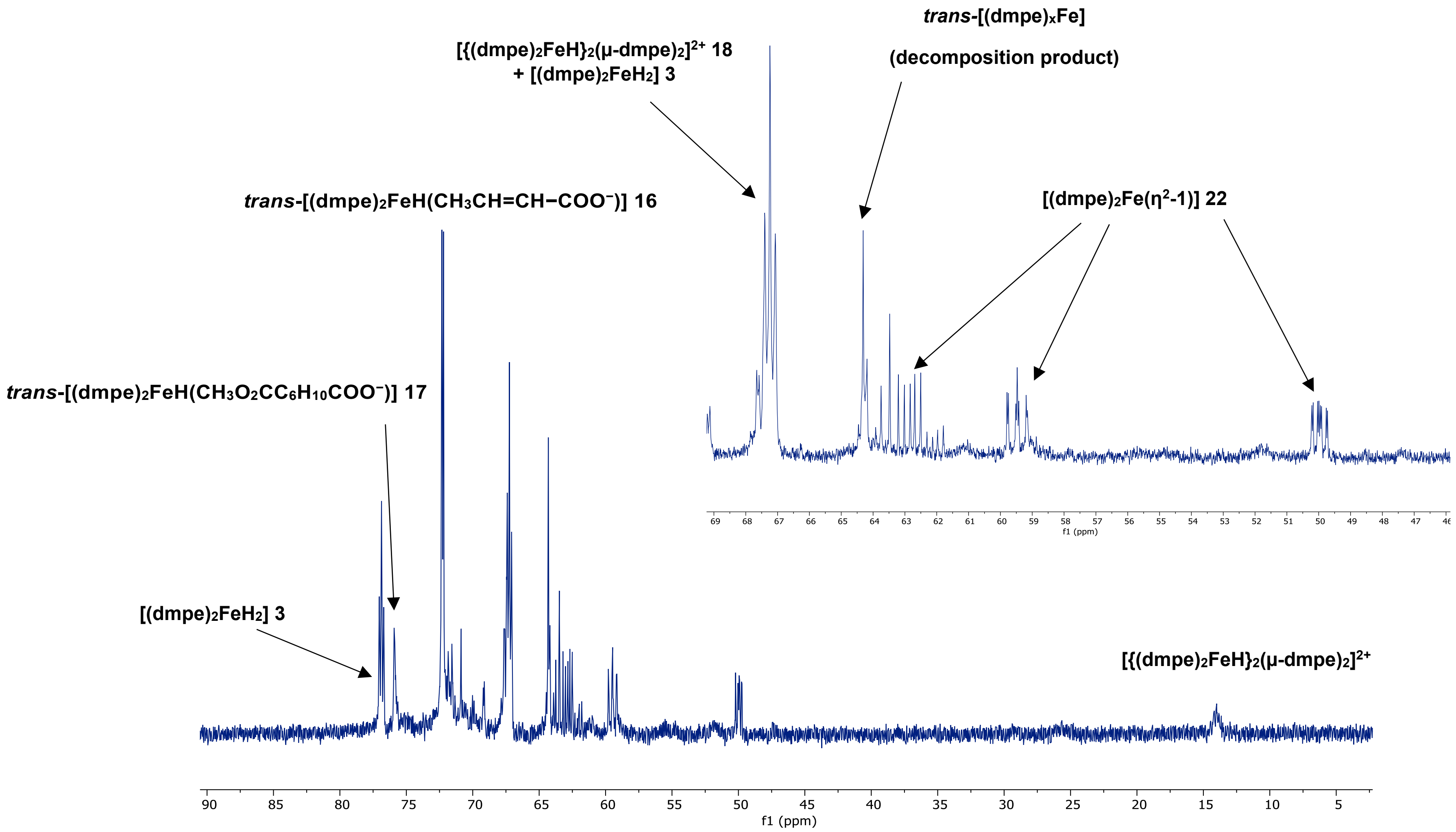

Supplementary Figure 60 – $^{31}P\{^{1}H\}$ NMR (202 MHz, THF-*d*$_8$) spectrum showing the middle of *in situ* monitoring showing the formation of [(dmpe)$_2$Fe(η^2-1)] **22** under light-mediated conditions dimerising methyl crotonate **1** with [(dmpe)$_2$FeH$_2$] **3** (5 mol%) for approximately 2 hours at 300 K.

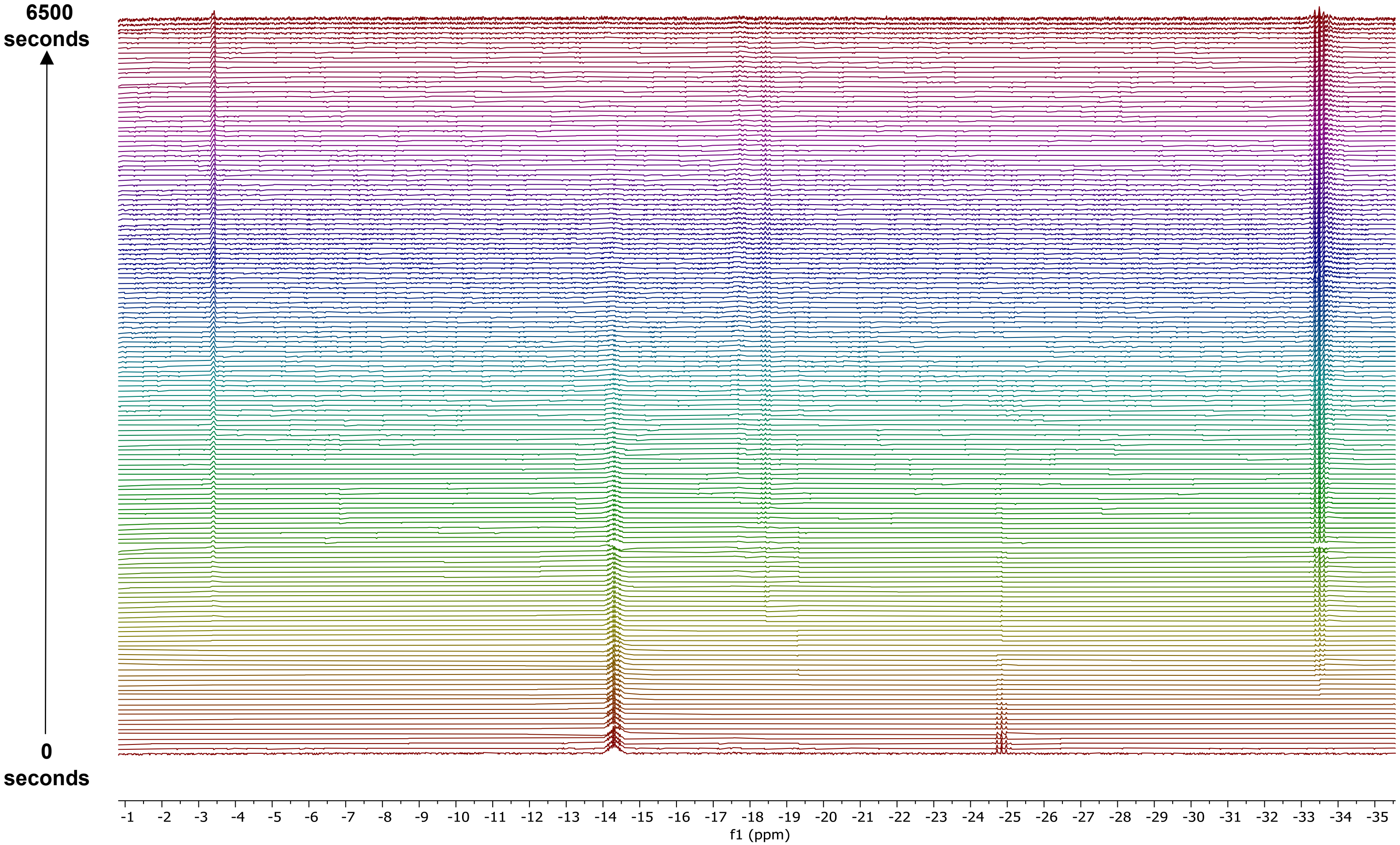

Supplementary Figure 61 – ^{1}H NMR (500 MHz, THF-d_8) stacked spectra (only hydride region shown) taken across approximately 2 hours under light-mediated conditions dimerising methyl crotonate **1** with [(dmpe)$_2$FeH$_2$] **3** (5 mol%) for approximately 2 hours at 300 K.

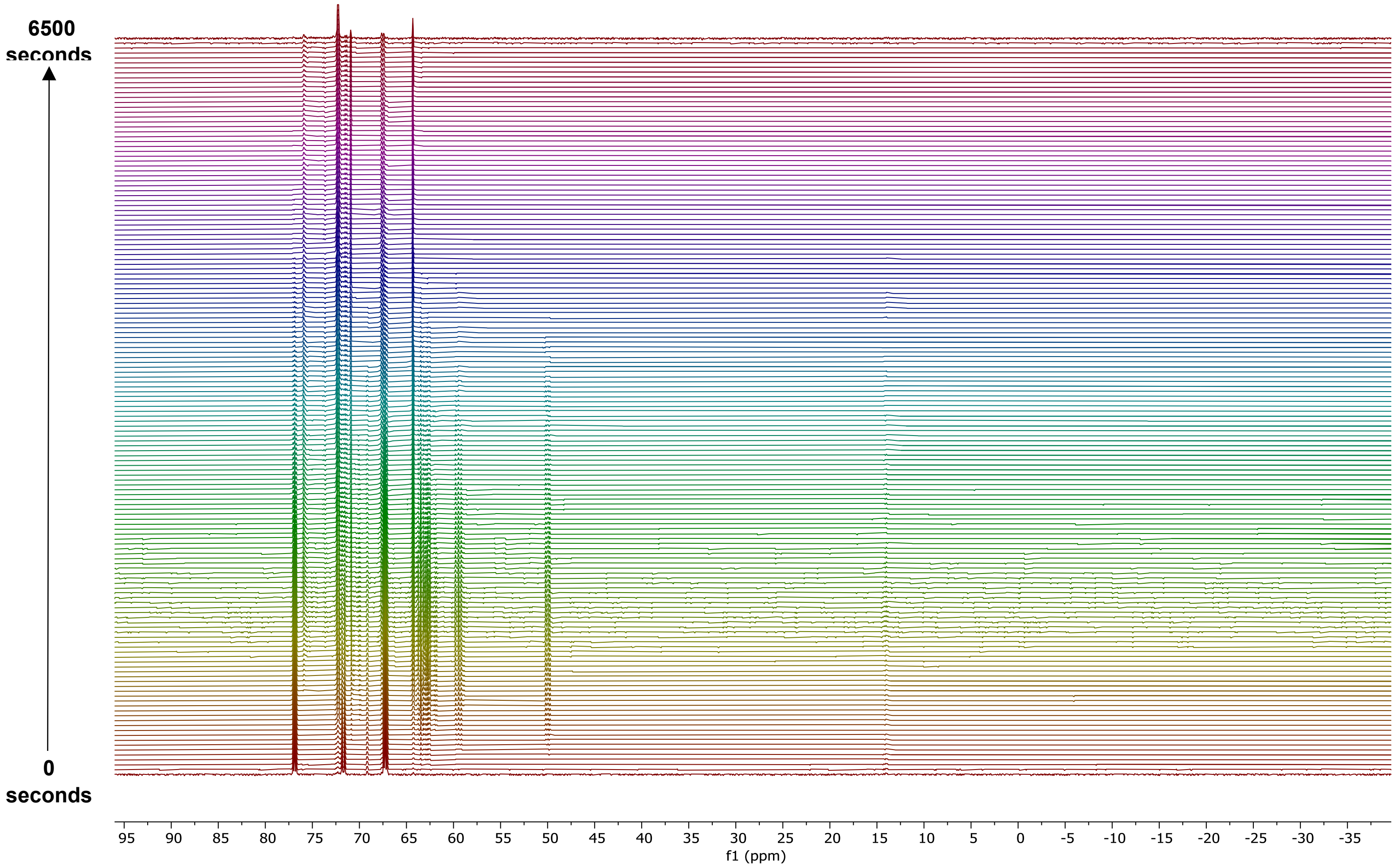

Supplementary Figure 62 – $^{31}P\{^{1}H\}$ NMR (202 MHz, THF-d_8) stacked spectra taken across approximately 2 hours under light-mediated conditions dimerising methyl crotonate **1** with $[(dmpe)_2FeH_2]$ **3** (5 mol%) for approximately 2 hours at 300 K.

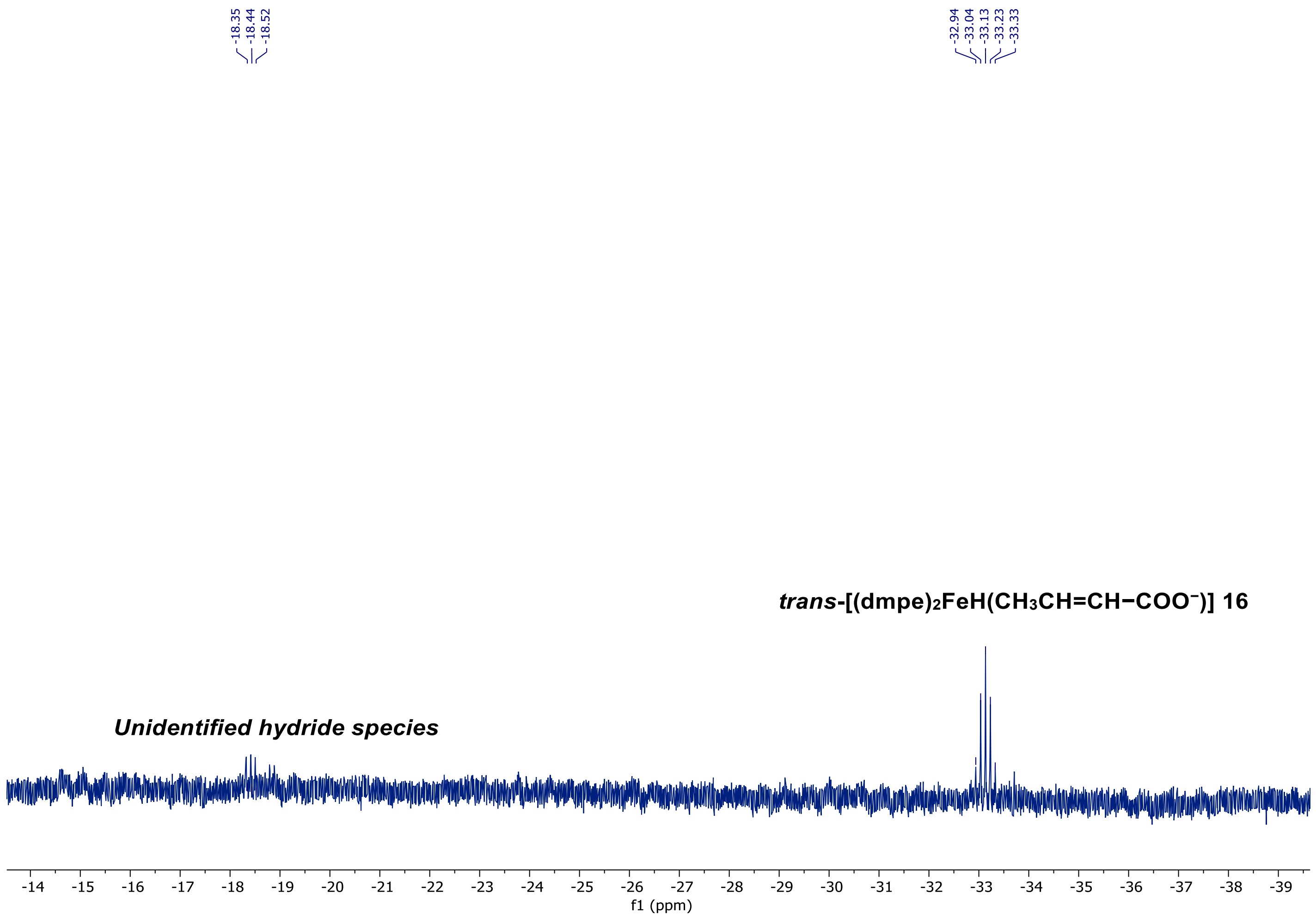

Supplementary Figure 63 – ^{1}H NMR (500 MHz, pentane) spectrum (hydride region shown) of a crystal [(dmpe)$_2$Fe(η^2-1)] **22**. The peak at δ −32.6 ppm (p, *J* = 50.3 Hz) is associated with *trans*-[(dmpe)$_2$FeH(CH$_3$CH=CH−COO$^-$)] **16**, with the peak at δ -17.8 ppm (ap t, *J*=45.8 Hz) was an unknown hydride species.

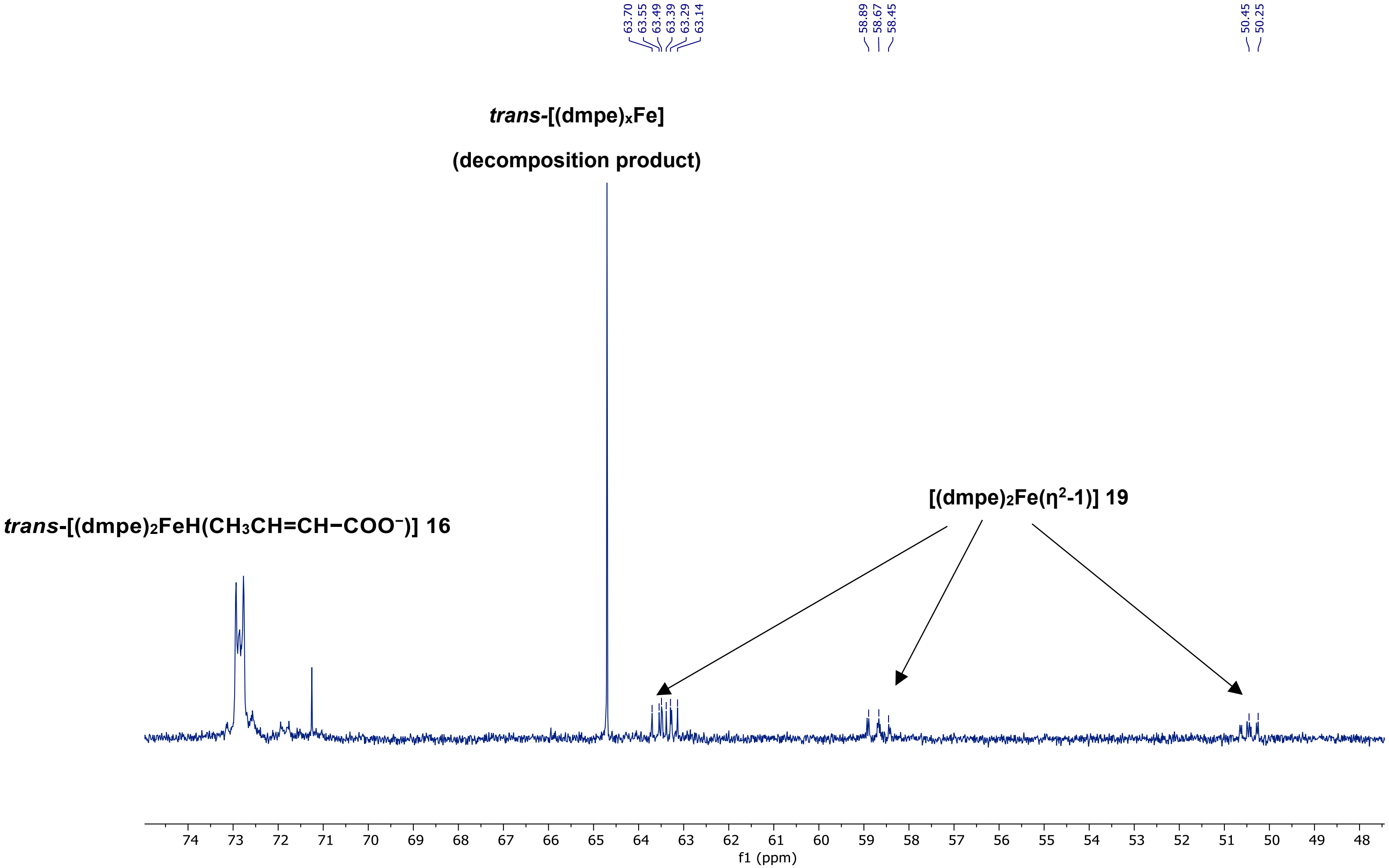

Supplementary Figure 64 – $^{31}P\{^{1}H\}$ NMR (202 MHz, pentane) spectra of the crystal of [(dmpe)$_2$Fe(η^2-1)] **22** with *trans*-[(dmpe)$_2$FeH(CH_3CH=CH−COO$^-$)] **16** and *trans*-[(dmpe)$_x$Fe] also present. It is unclear whether [(dmpe)$_2$Fe(η^2-1)] **22** directly decomposes to other species visible in this spectrum.

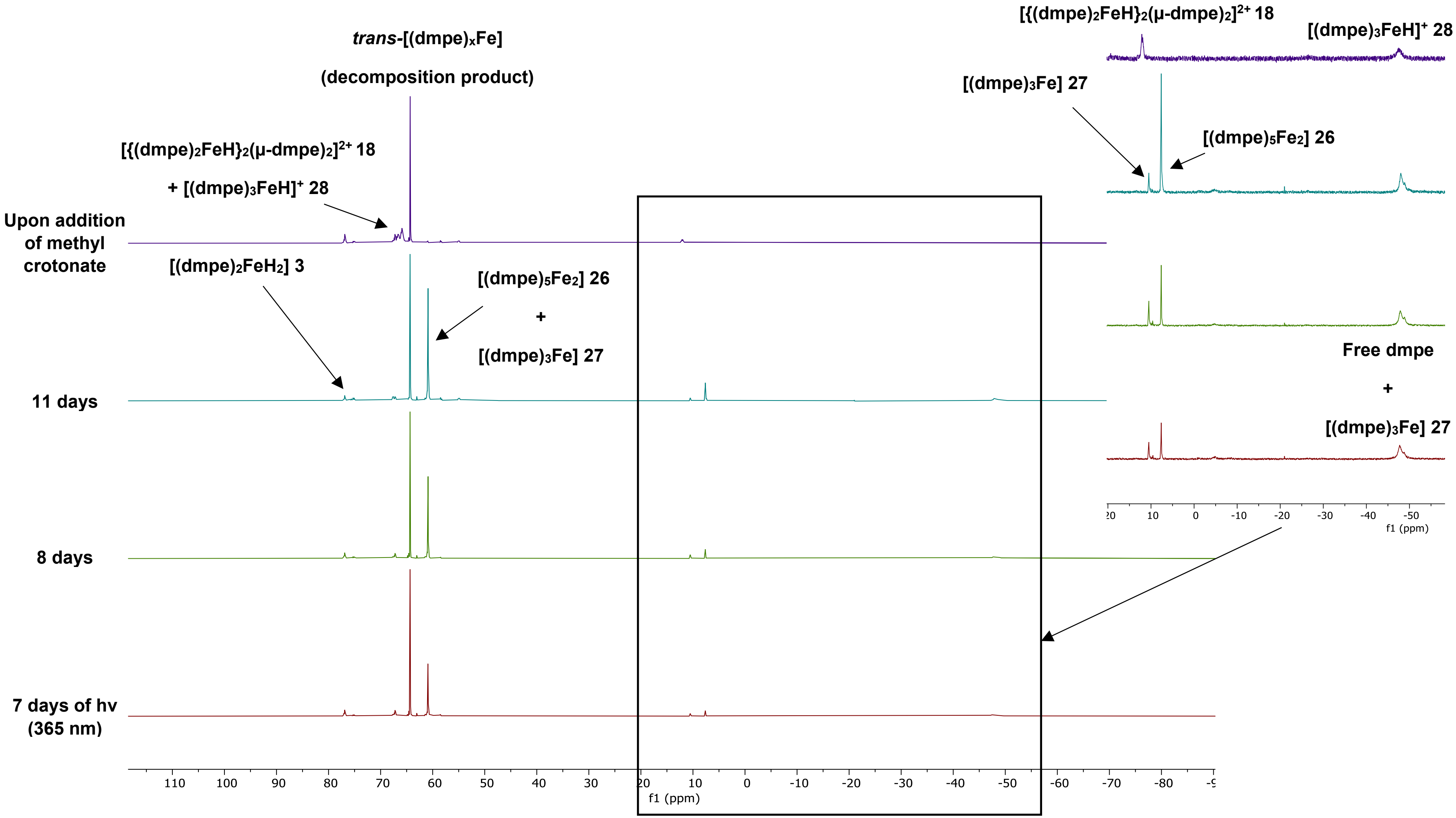

Supplementary Figure 65 – $^{31}P\{^{1}H\}$ NMR (202 MHz, THF-d_8) showing the irradiation of [(dmpe)$_2$FeH$_2$] **3** (5 mol%) and THF-d_8 over 11 days (red, green and blue lines) and then upon addition of methyl crotonate **1** (purple line).

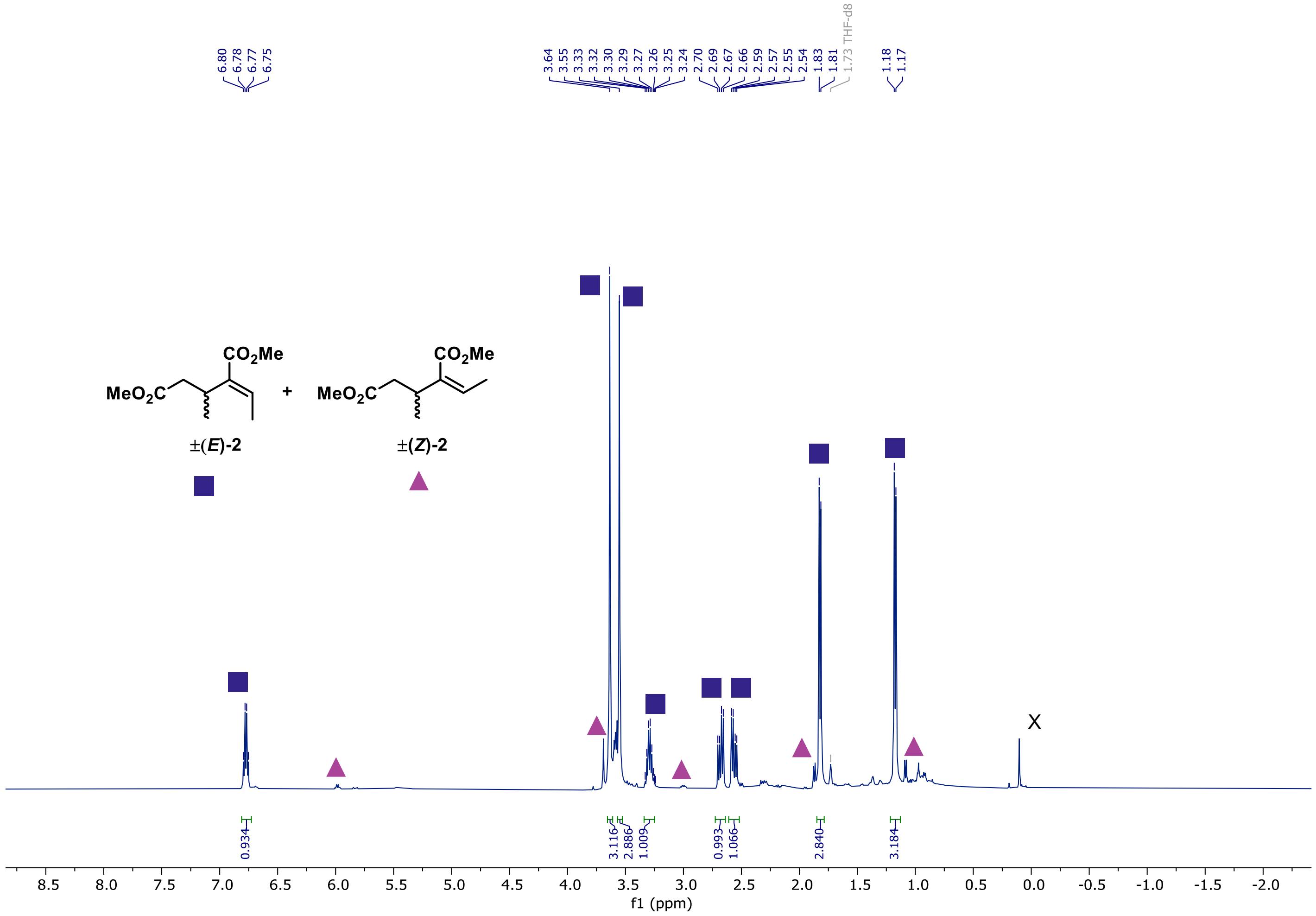

Supplementary Figure 66 – ^{1}H NMR (500MHz, THF-d_8) spectrum upon methyl crotonate addition to a [(dmpe)$_5$Fe$_2$] **26**/ [(dmpe)$_3$Fe] **27** mixture. The signal denoted by X is residual grease in the THF-d_8.

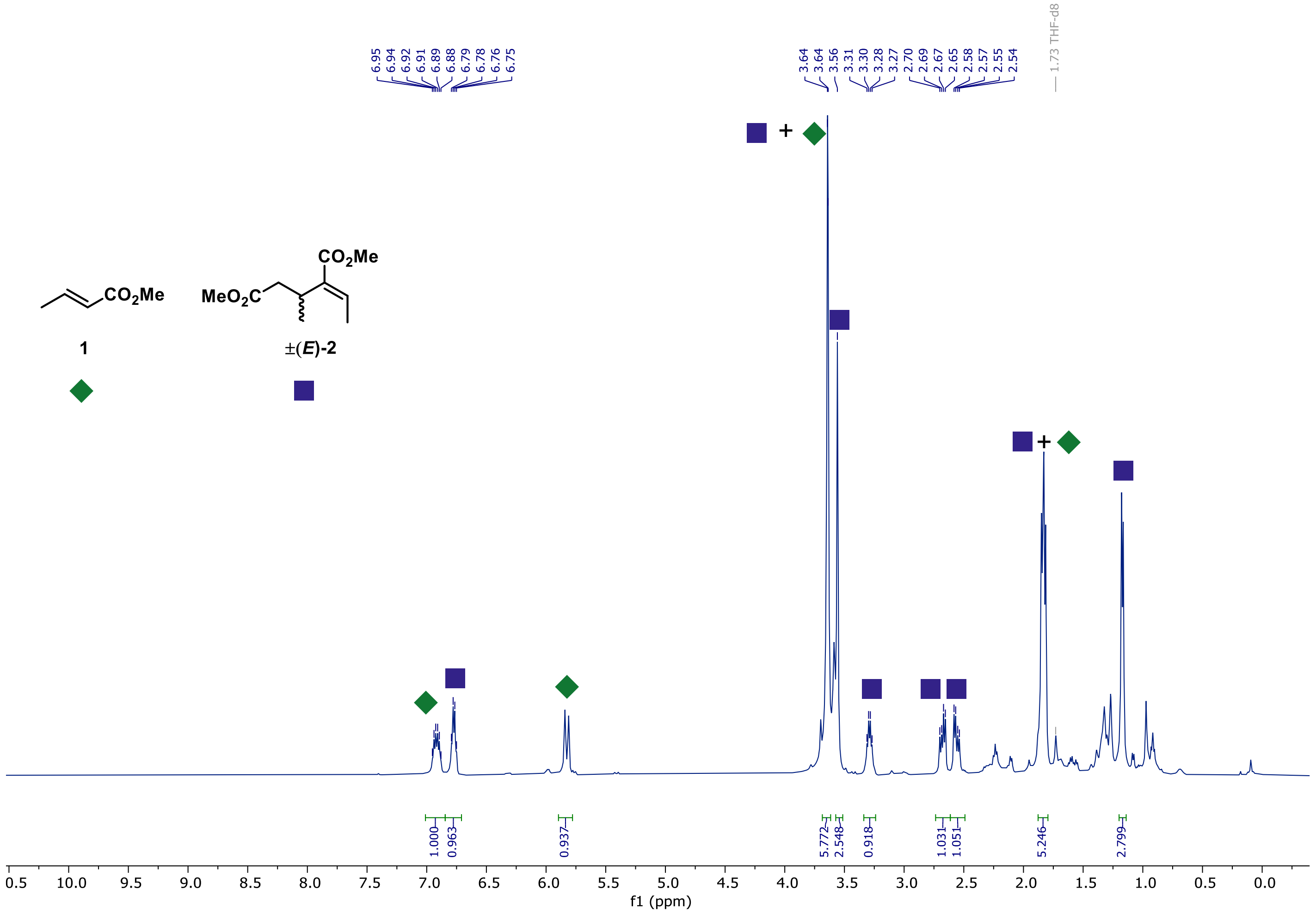

Supplementary Figure 67 – ^{1}H NMR (500 MHz, THF-d_8) spectrum of [(dmpe)$_2$FeH$_2$] **3** and methyl 3-butenoate **19** and irradiating (365 nm) for 2 hours, showing isomerisation to methyl crotonate **1** and subsequent dimerisation to (*E*),(*Z*)-2-ethylidene-3-methylpentanedioate **2**.

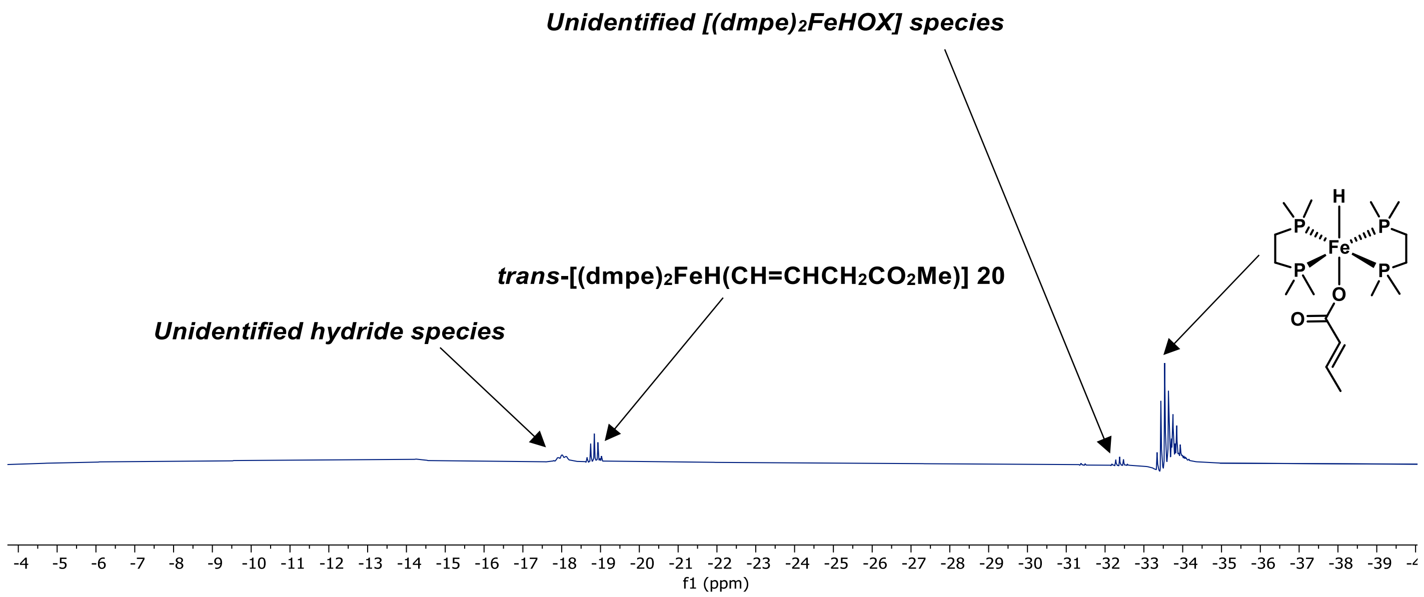

Supplementary Figure 68 – ^{1}H NMR (500 MHz, THF-d_8) spectrum of the hydride region of [(dmpe)$_2$FeH$_2$] **3** and methyl 3-butenoate **19** and irradiating (365 nm) for 2 hours.

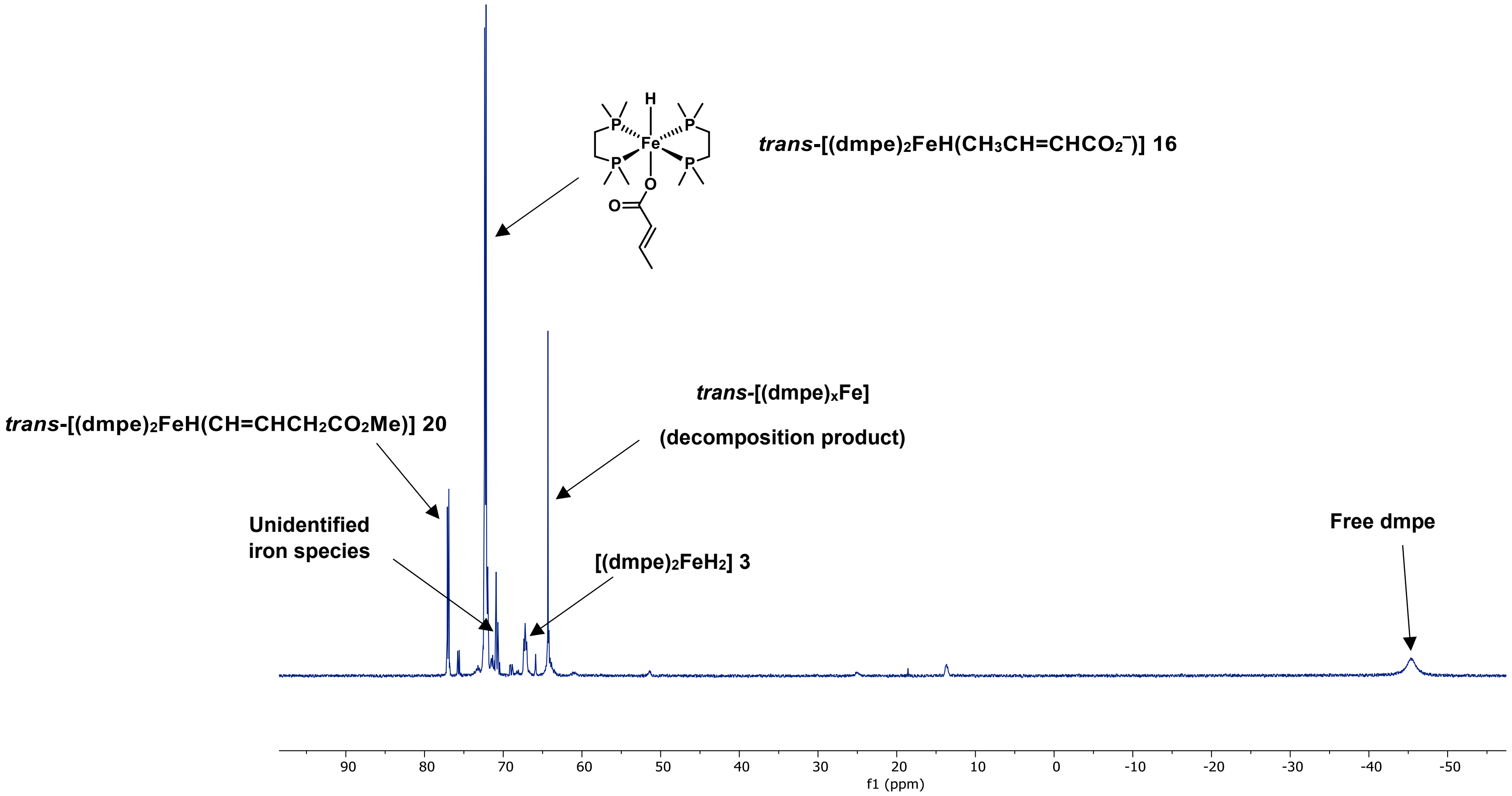

Supplementary Figure 69 – $^{31}P\{^1H\}$ NMR (202 MHz, THF-d_8) spectrum of [(dmpe)$_2$FeH$_2$] **3** and methyl 3-butenoate **19** and exposed to UV (365 nm) for 2 hours.

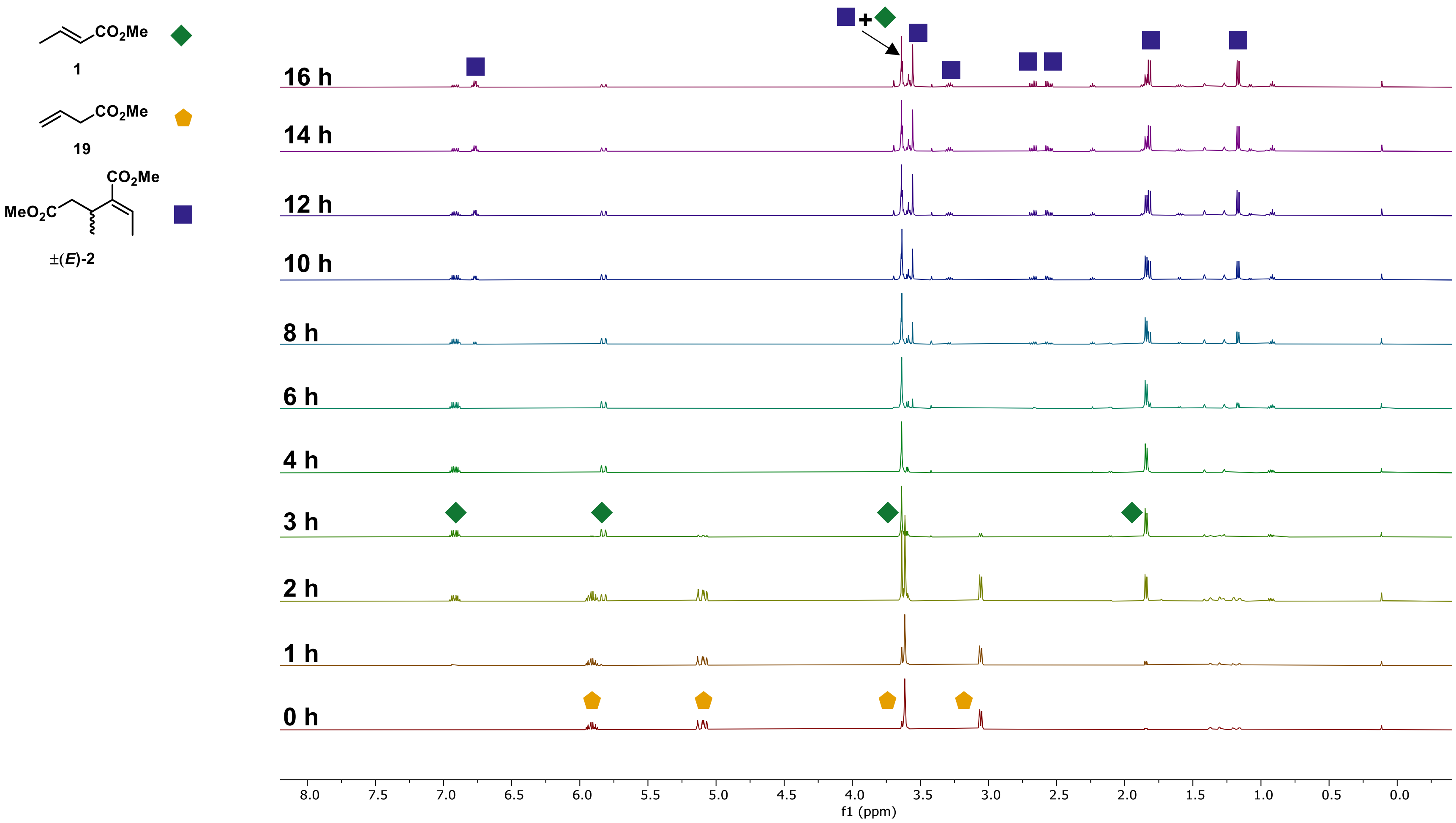

Supplementary Figure 70 – Stacked ^{1}H NMR (500 MHz, THF-d_8) spectra of [(dmpe)$_2$FeH$_2$] **3** and methyl 3-butenoate **19** under no-light conditions over 16 hours.

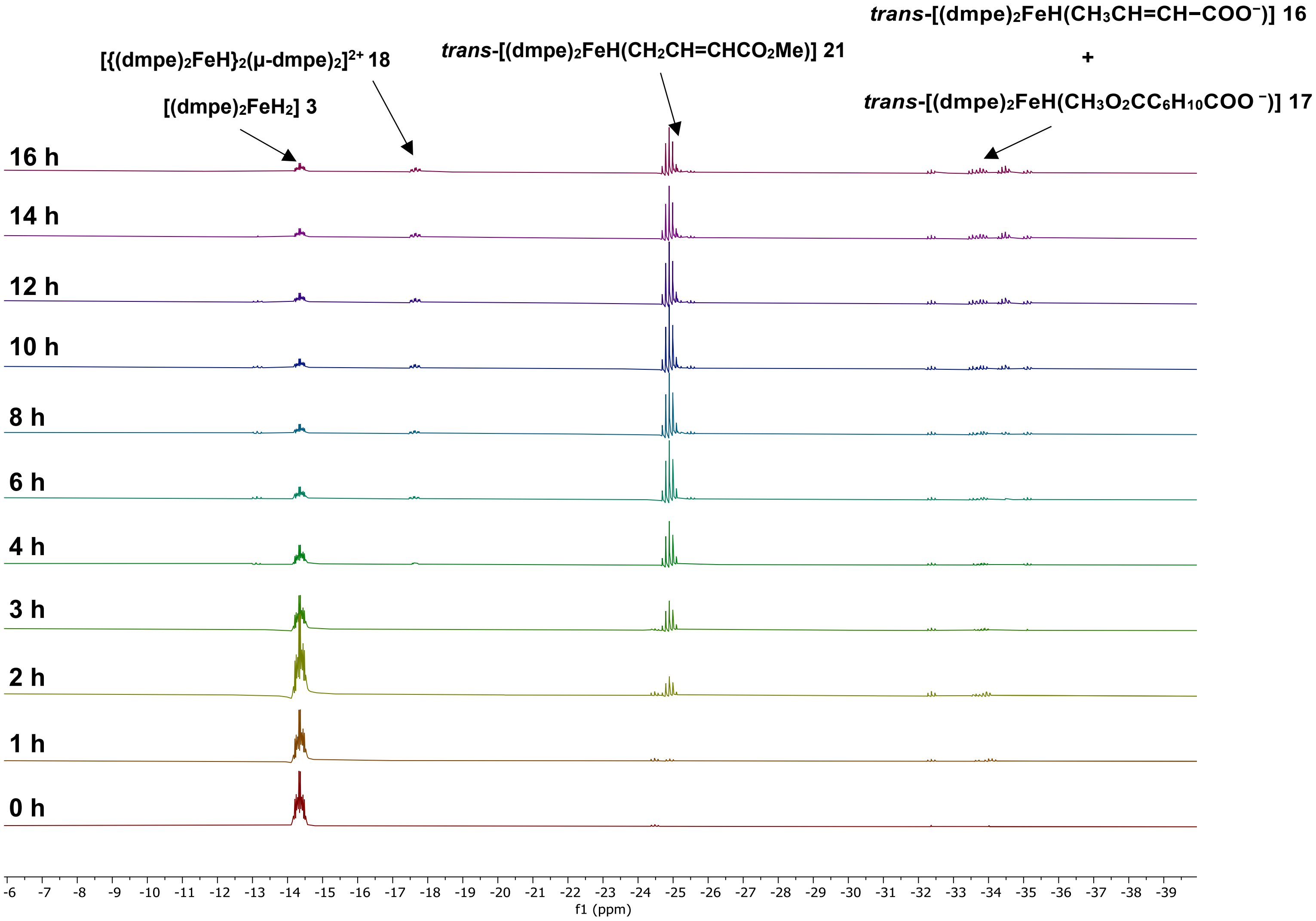

Supplementary Figure 71 – Stacked ^{1}H NMR (500 MHz, THF-d_8) spectra of the hydride region of [(dmpe)$_2$FeH$_2$] **3** and methyl 3-butenoate **19** under no-light conditions over 16 hours.

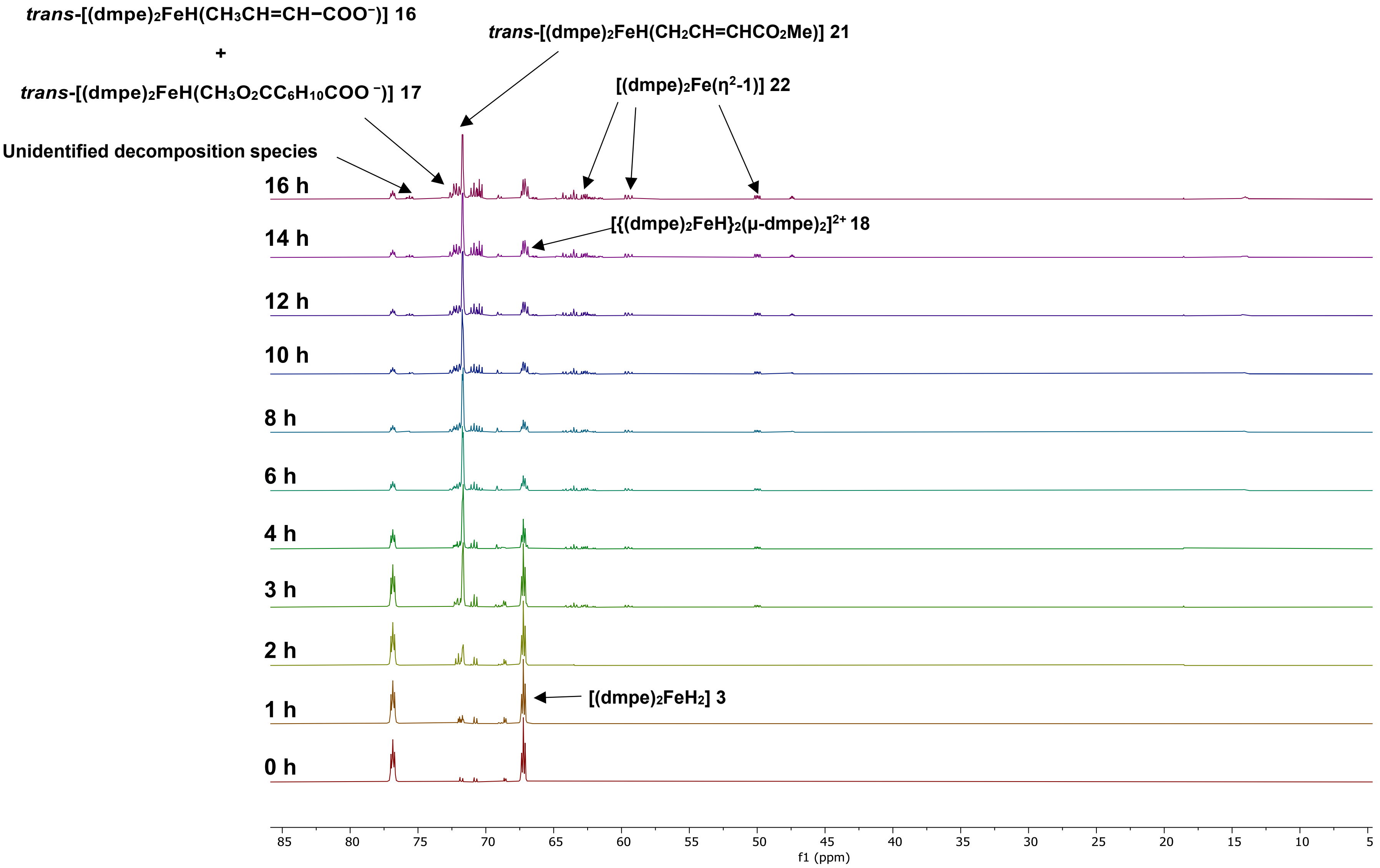

Supplementary Figure 72 – Stacked $^{31}P\{^{1}H\}$ NMR (500 MHz, THF-d_8) spectra of [(dmpe)$_2$FeH$_2$] **3** and methyl 3-butenoate **19** under no-light conditions over 16 hours.

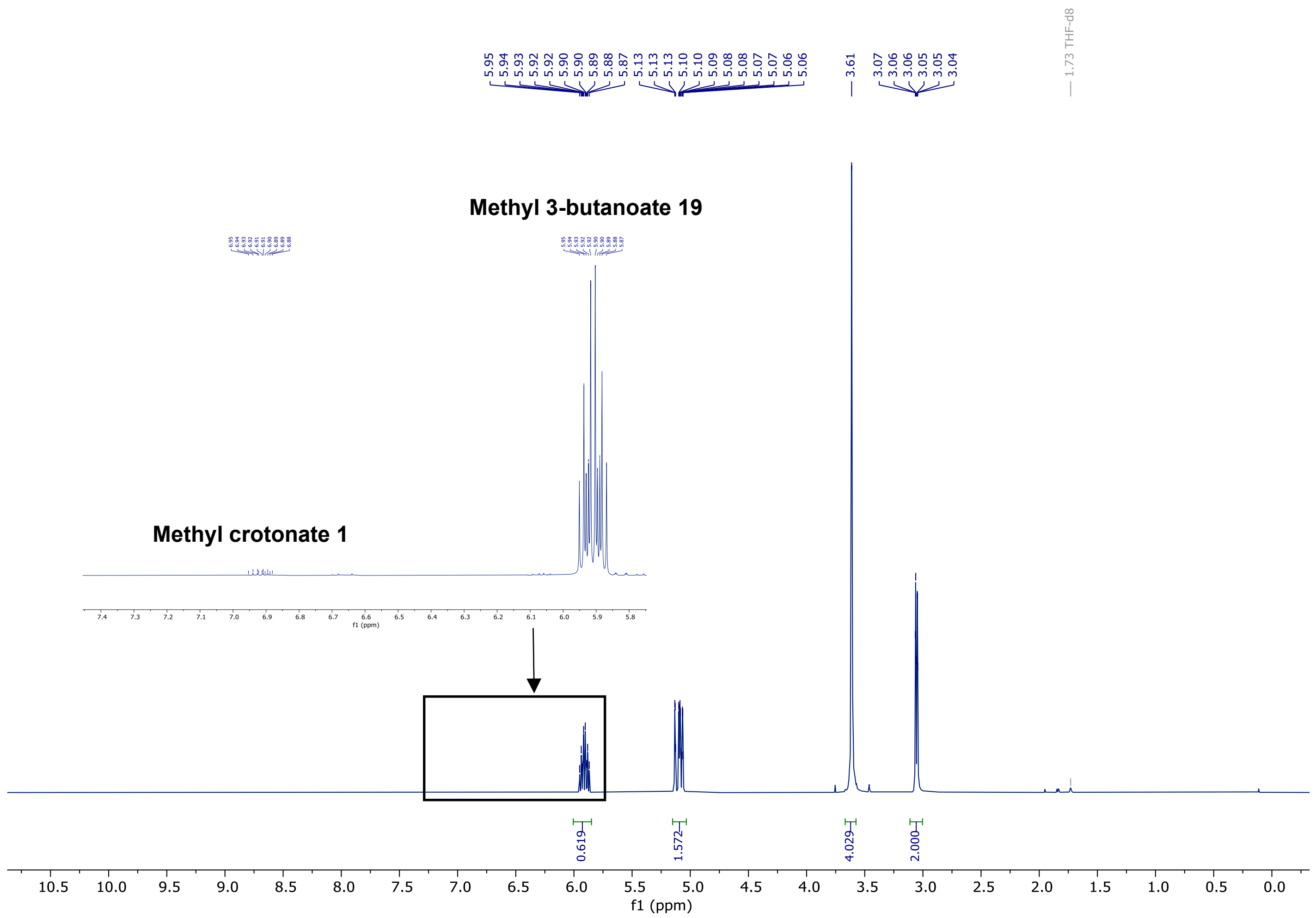

Supplementary Figure 73 – ^{1}H NMR (500 MHz, THF-d_8) spectrum showing methyl 3-butanoate **19** with sodium acetate (at 5 mol%) and <2% isomerisation to methyl crotonate **1** after 16 hours.

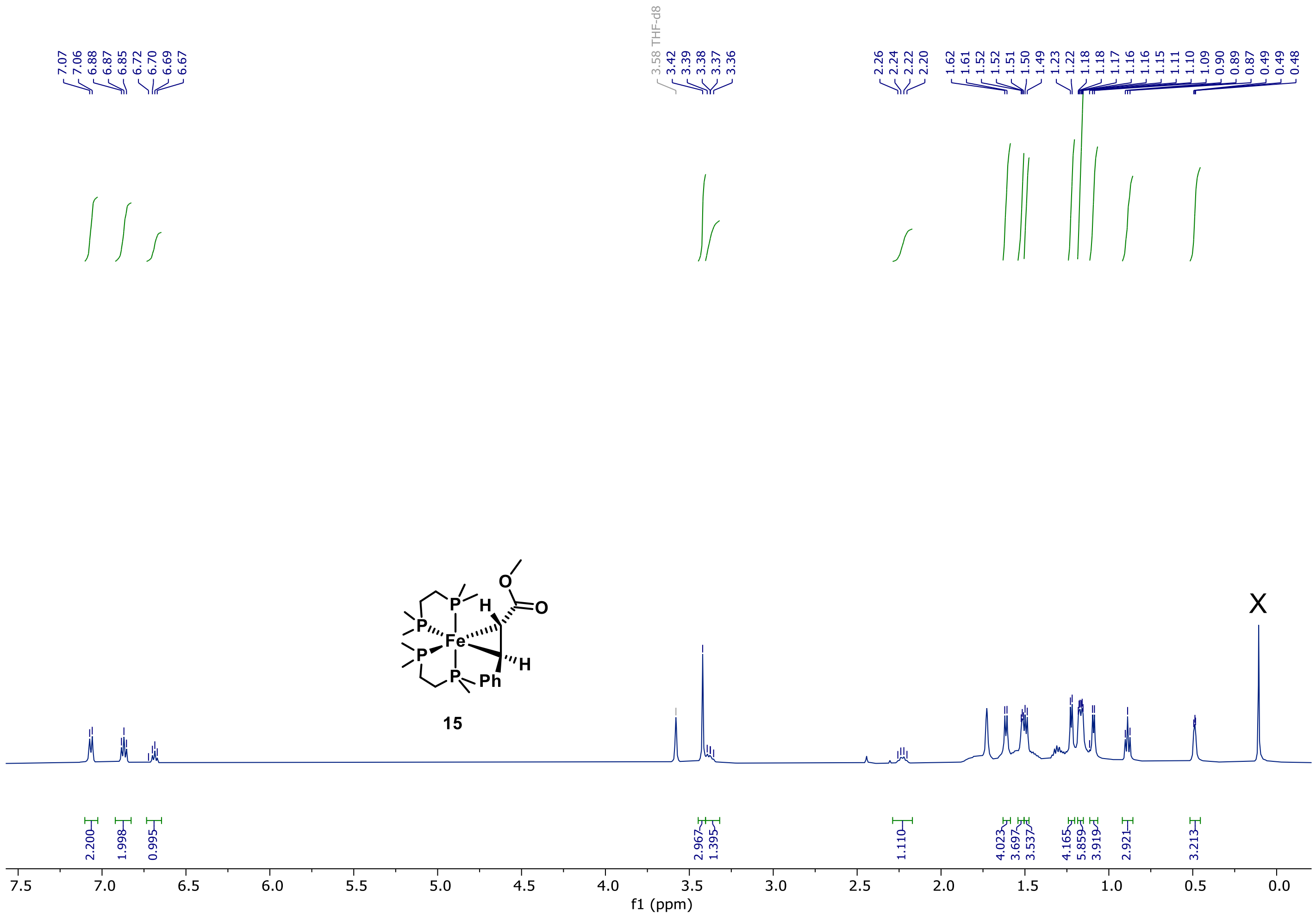

Supplementary Figure 74 – ^{1}H NMR (500 MHz, THF-d_8) spectrum of [(dmpe)$_2$Fe(PhCH=CHCO$_2$Me)] **15**. The signal denoted by X is residual grease from the solvent.

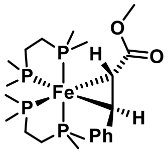

15

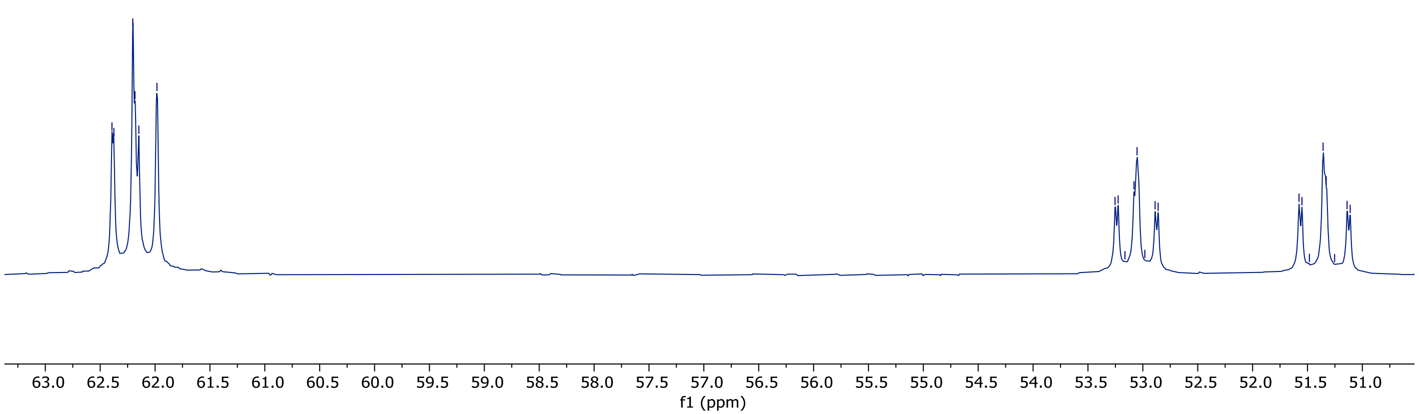

Supplementary Figure 75 – $^{31}P\{^{1}H\}$ NMR (202 MHz, THF-d_8) spectrum of [(dmpe)$_2$Fe(PhCH=CHCO$_2$Me)] **15**.

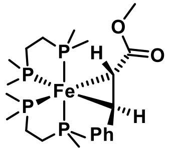

15

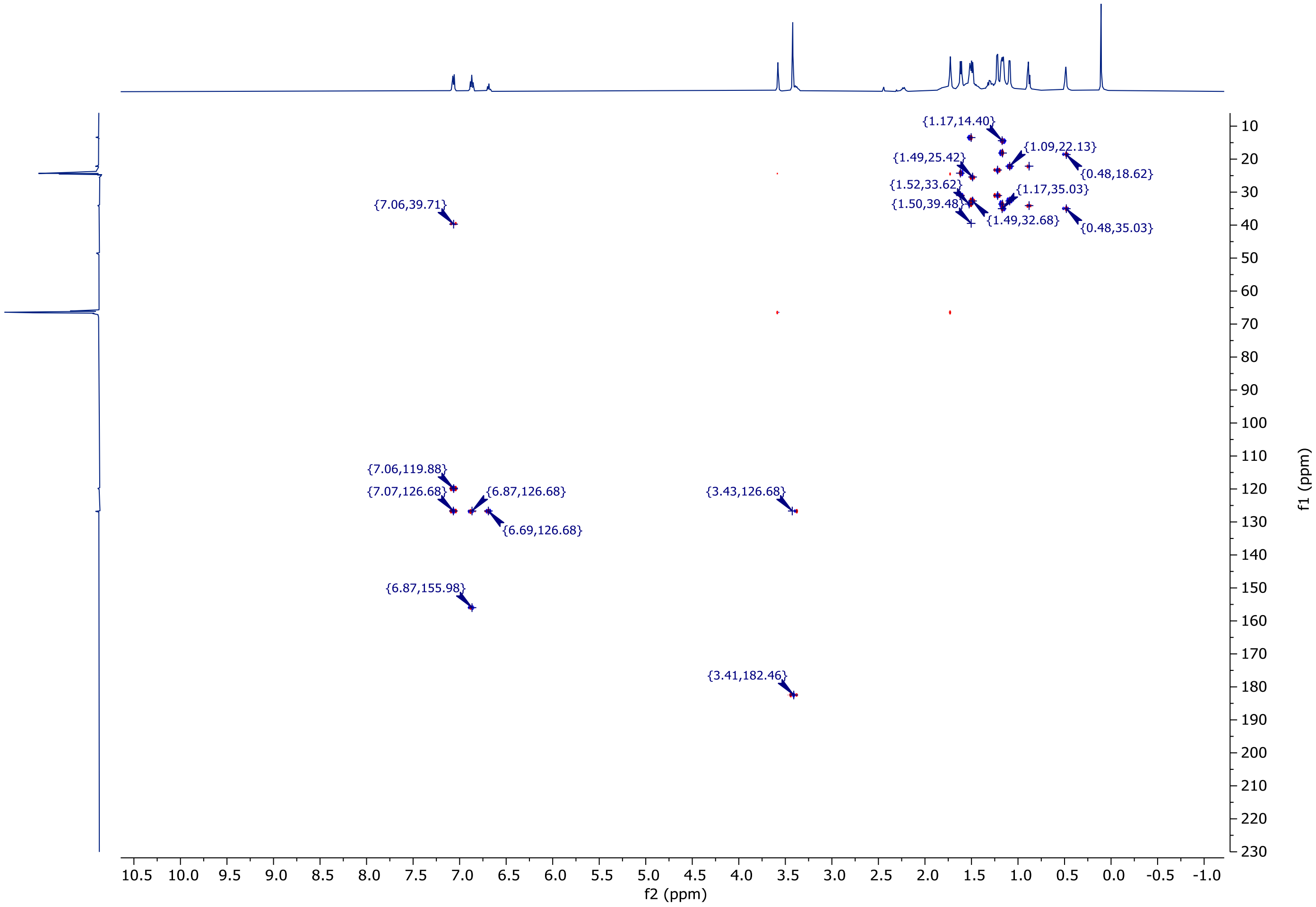

Supplementary Figure 76 – ^{1}H–^{13}C HMBC NMR spectrum of [(dmpe)$_2$Fe(PhCH=CHCO$_2$Me)] **15**.

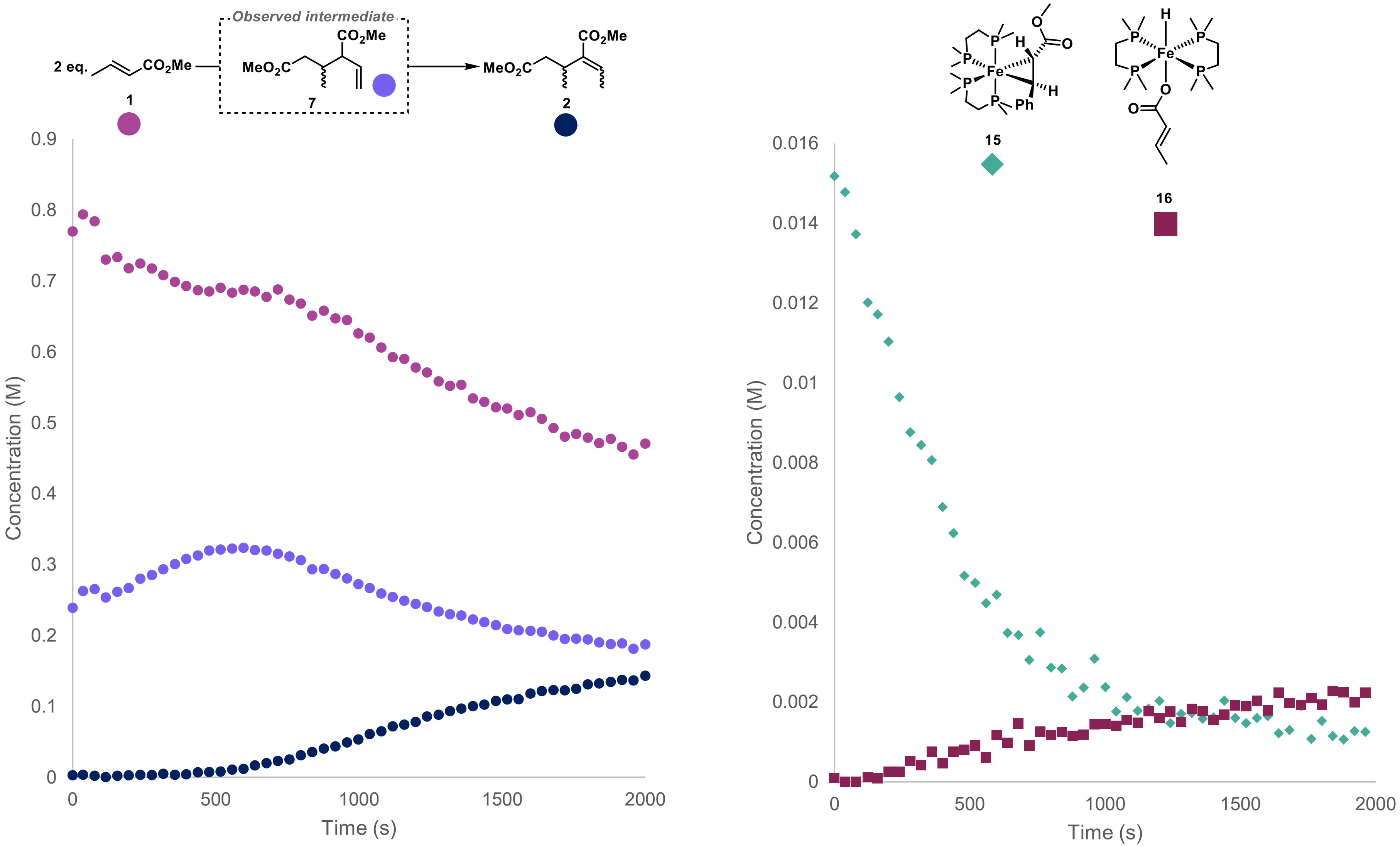

Supplementary Figure 77 – *in situ* Monitoring by ^{1}H NMR (left) and ^{31}P{^{1}H} NMR spectroscopy (right) showing that [(dmpe)$_2$Fe(PhCH=CHCO$_2$Me] **15** is catalytically active at 5 mol% loading *versus* methyl crotonate **1**.

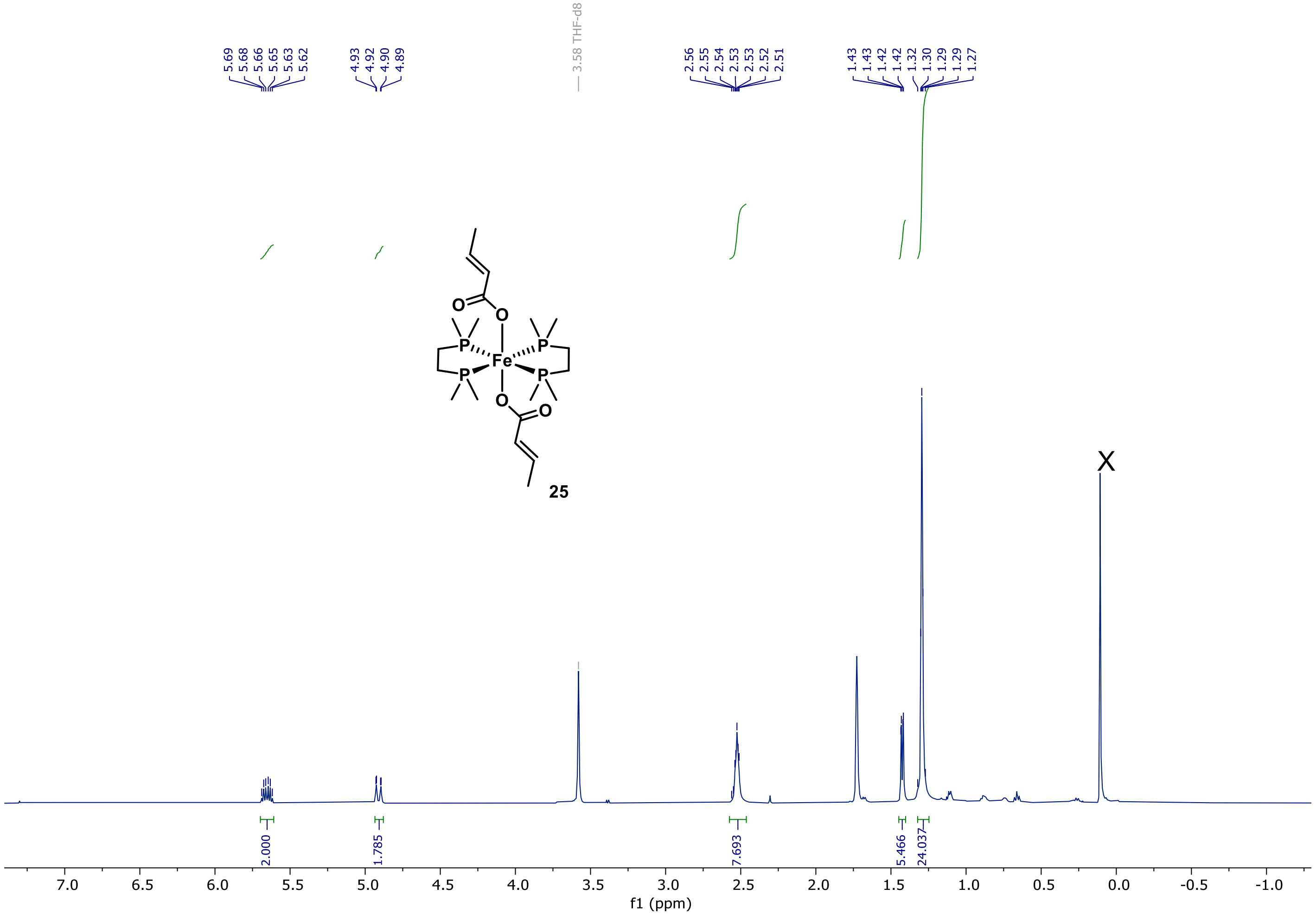

Supplementary Figure 78 –^{1}H NMR (500 MHz, THF-d_8) spectrum of *trans*-[(dmpe)$_2$Fe(CH$_3$CH=CH−COO$^-$)$_2$] **25**. The signal denoted by the symbol X is residual grease from the solvent.

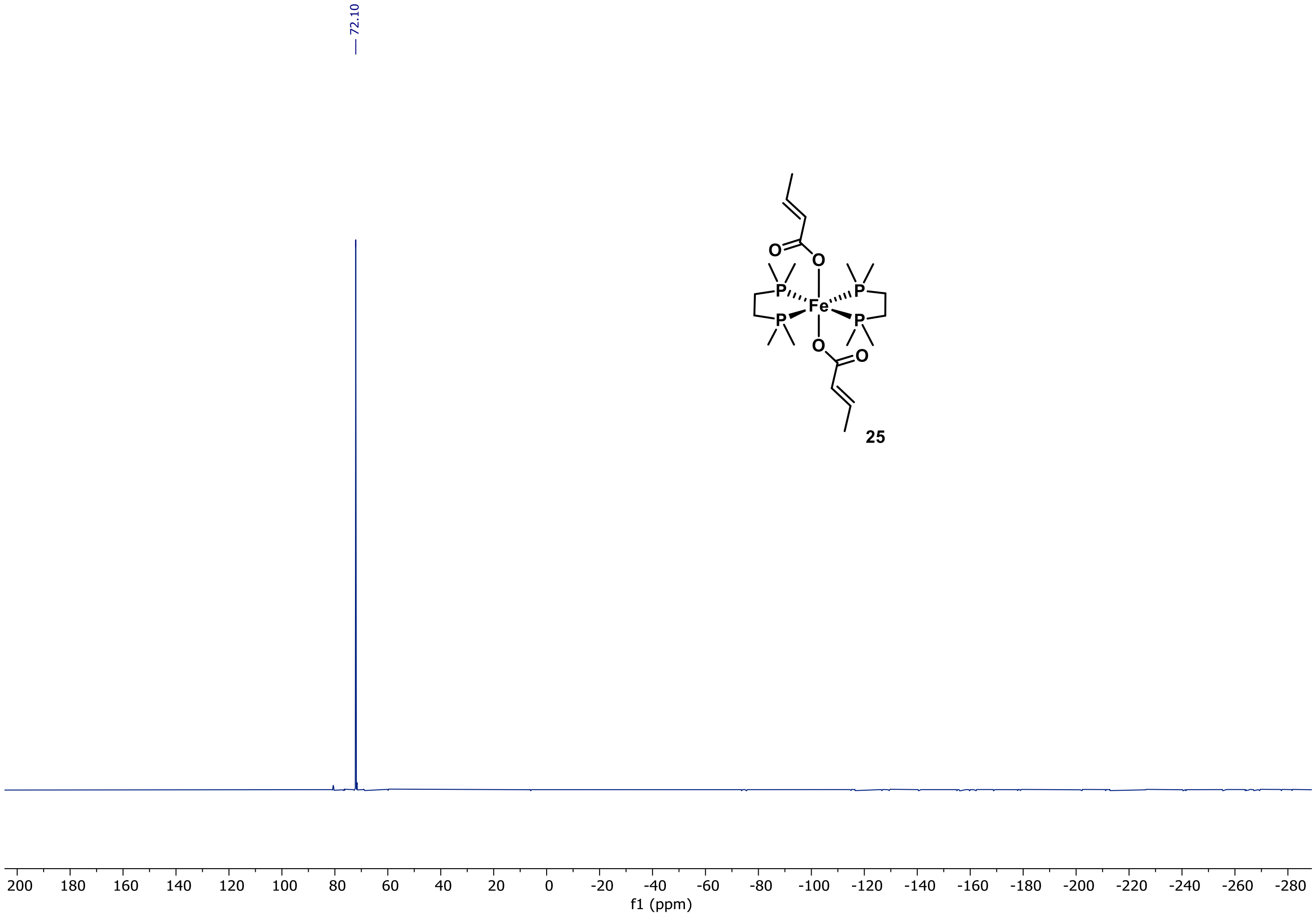

Supplementary Figure 79 –$^{31}P\{^1H\}$ NMR (202 MHz, THF-d_8) spectrum of *trans*-[(dmpe)$_2$Fe(CH$_3$CH=CH−COO$^-$)$_2$] **25**.

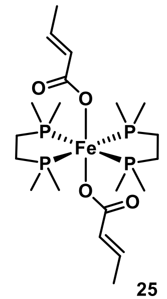

25

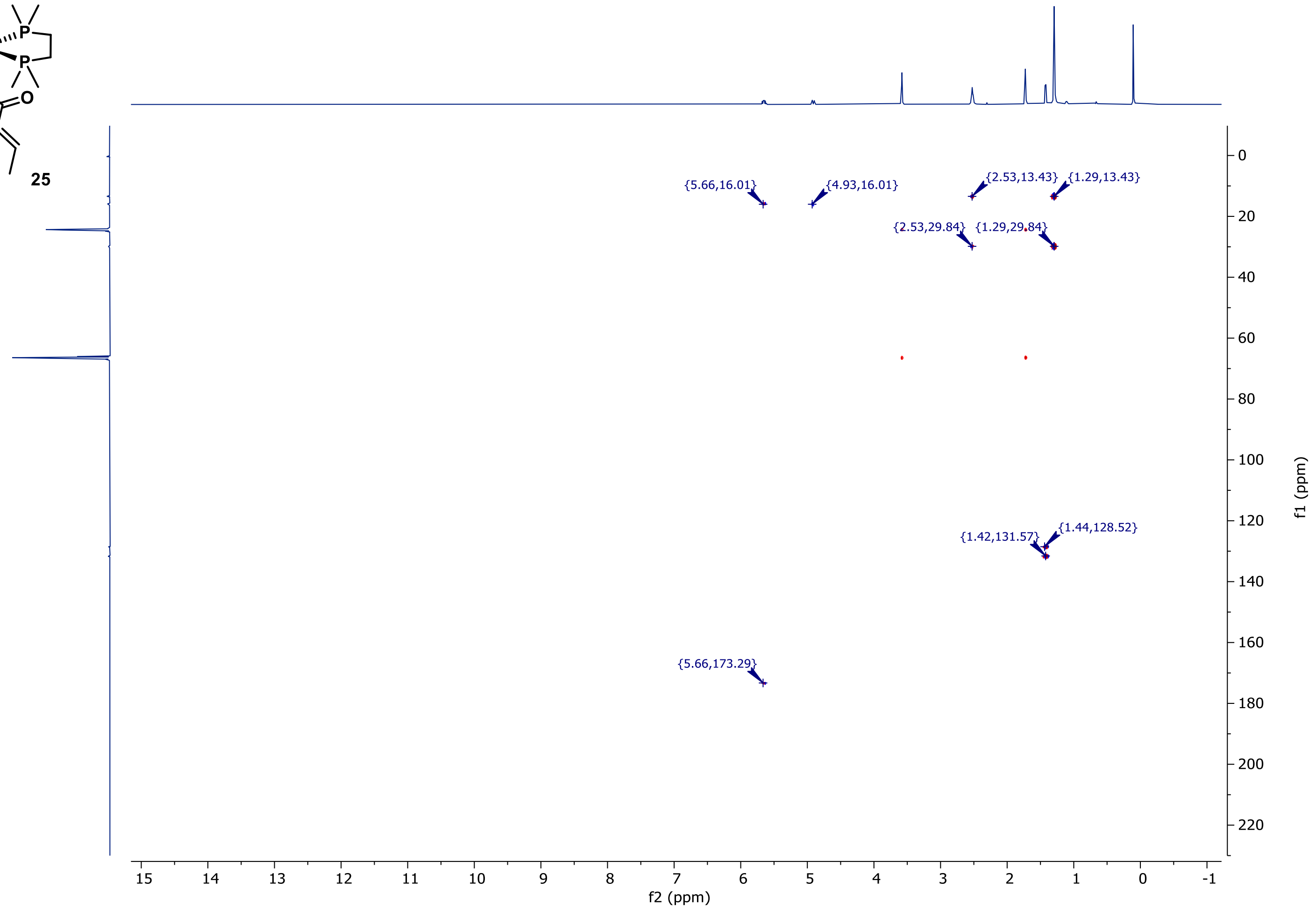

Supplementary Figure 80 – ^{1}H−^{13}C HMBC NMR (THF-d_8) spectrum of *trans*-[(dmpe)$_2$Fe(CH$_3$CH=CH−COO^{-})$_2$] **25**.

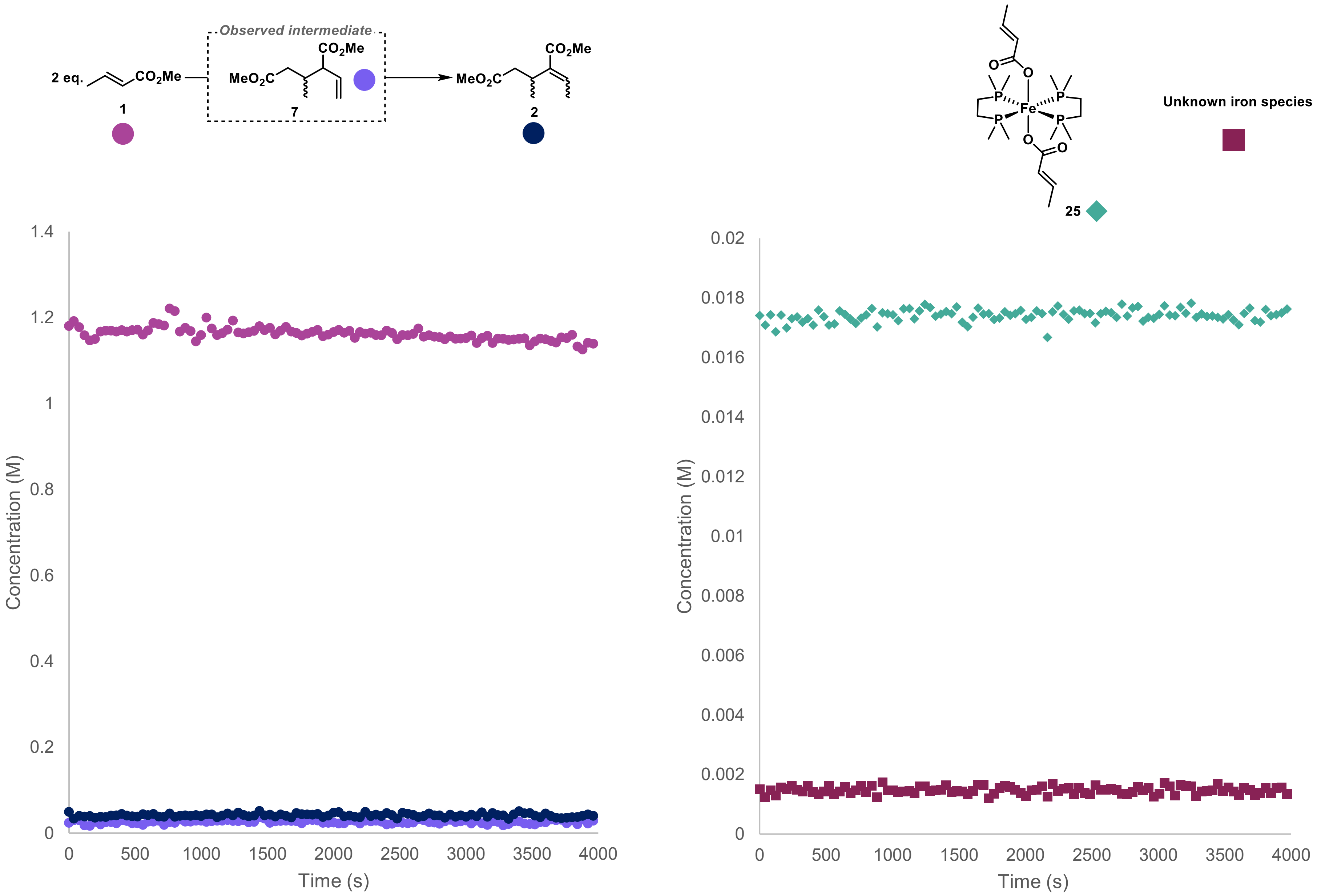

Supplementary Figure 81 – *in situ* Monitoring by ^{1}H NMR (left) and $^{31}P\{^{1}H\}$ NMR spectroscopy (right) showing that *trans*-[(dmpe)$_2$Fe($CH_3CH=CH-COO^-$)$_2$] **25** is catalytically inactive in the presence of methyl crotonate **1**.

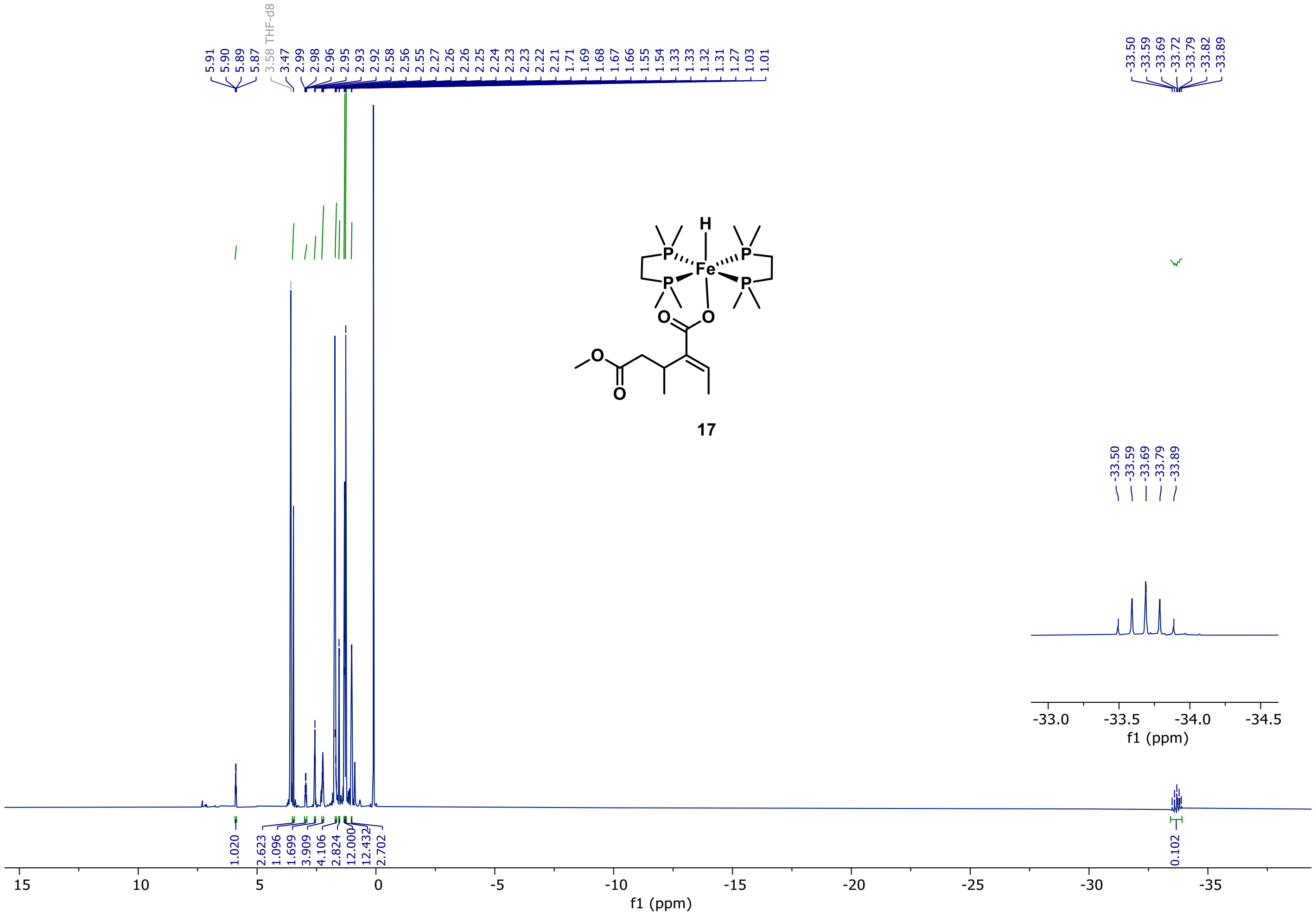

Supplementary Figure 82 – ^{1}H NMR (500 MHz, THF-d_8) spectrum of *trans*-[(dmpe)$_2$FeH(CH$_3$O$_2$CC$_6$H$_{10}$COO$^-$)] **17**.

.

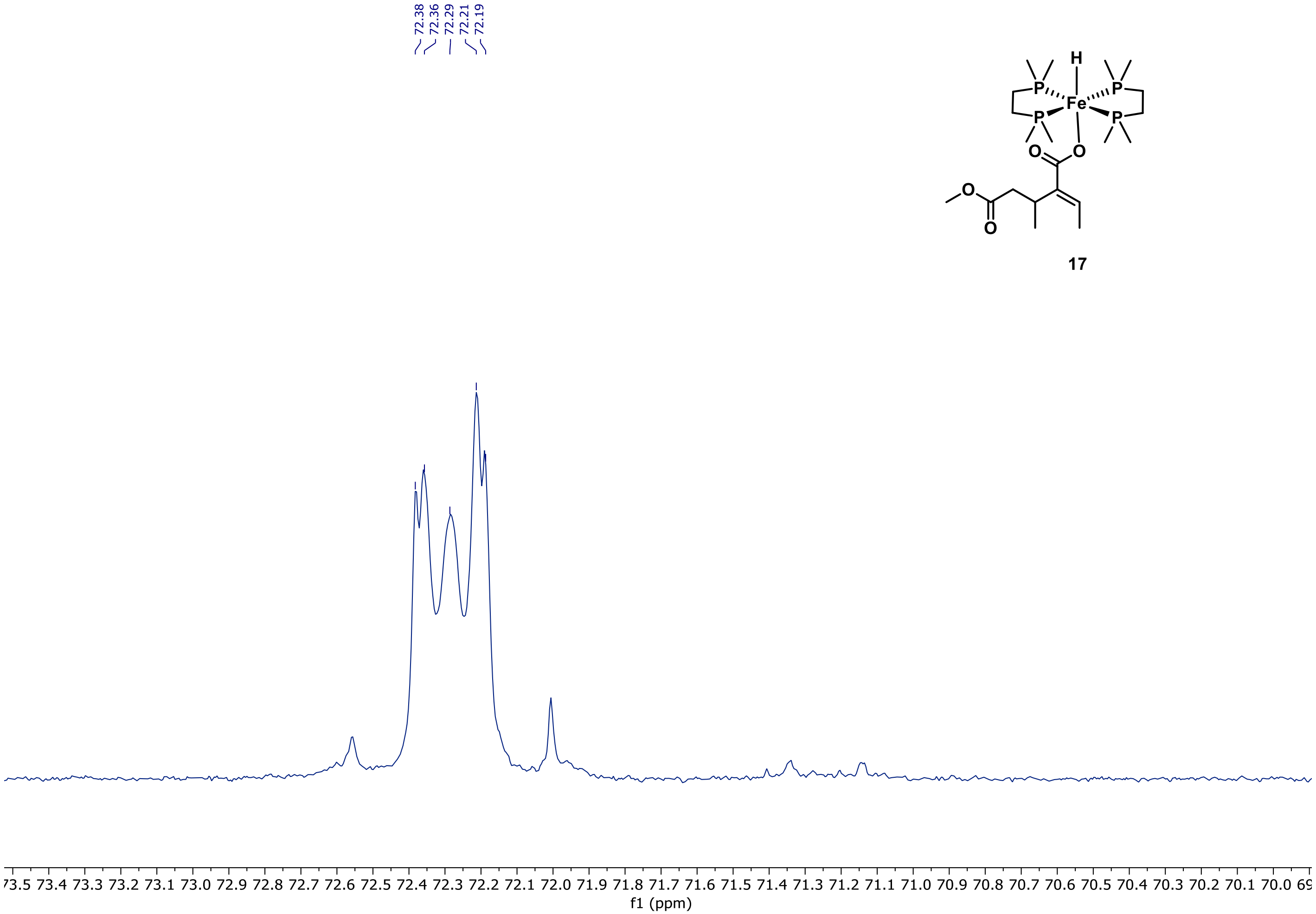

17

Supplementary Figure 83 – $^{31}P\{^1H\}$ NMR (202 MHz, THF-d_8) spectrum of *trans*-[(dmpe)$_2$FeH(CH$_3$O$_2$CC$_6$H$_{10}$COO$^-$)] **17**.

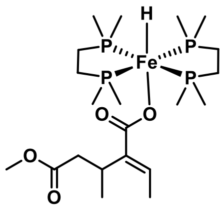

17

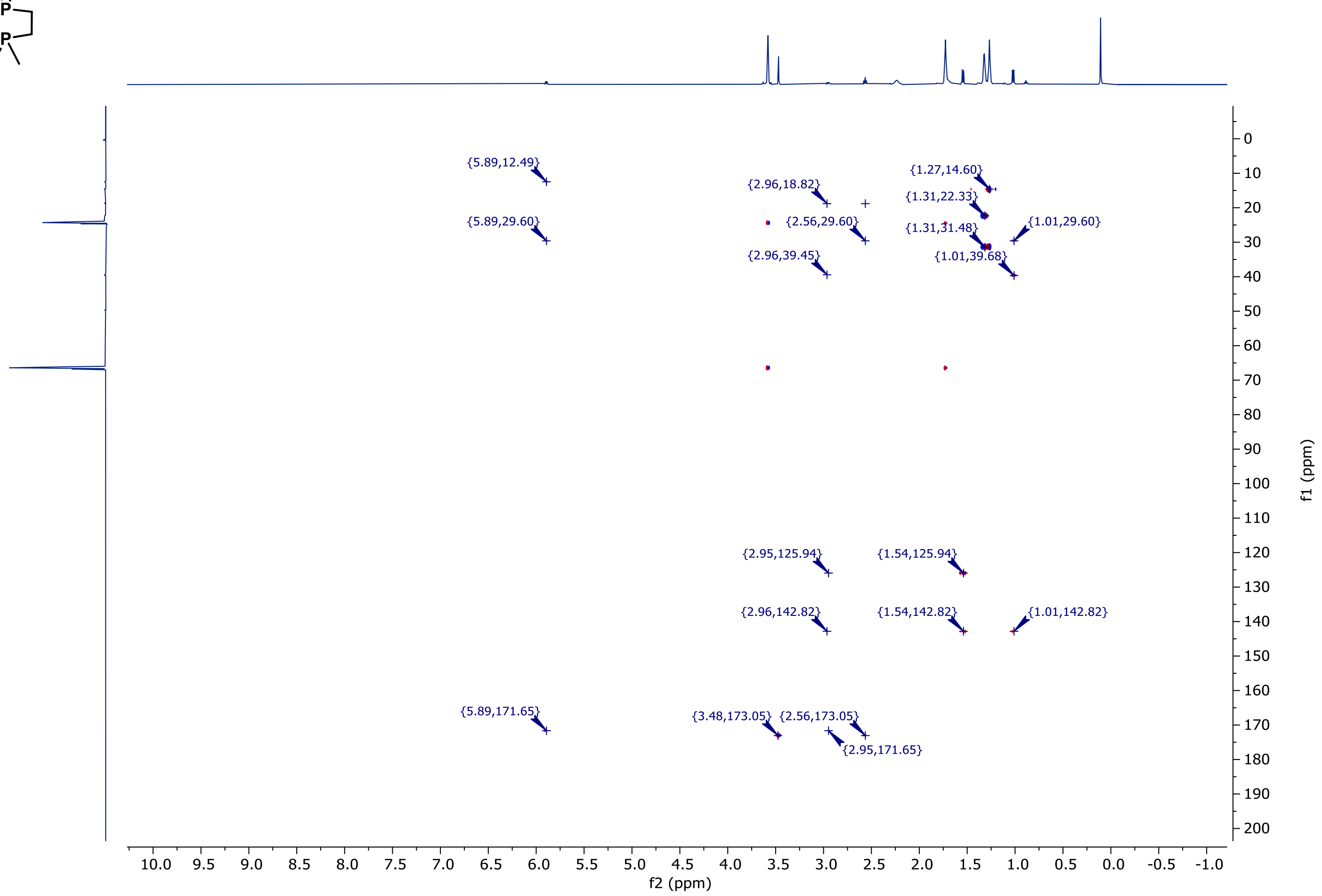

Supplementary Figure 84 – ^{1}H–^{13}C HMBC NMR (THF-*d*$_8$) spectrum of *trans*-[(dmpe)$_2$FeH($CH_3O_2CC_6H_{10}COO^-$)] **17**.

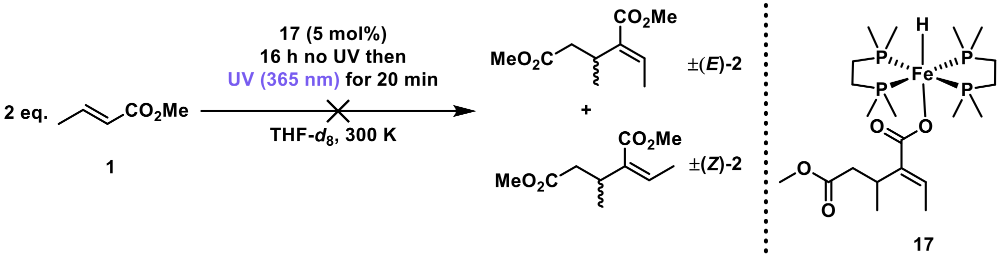

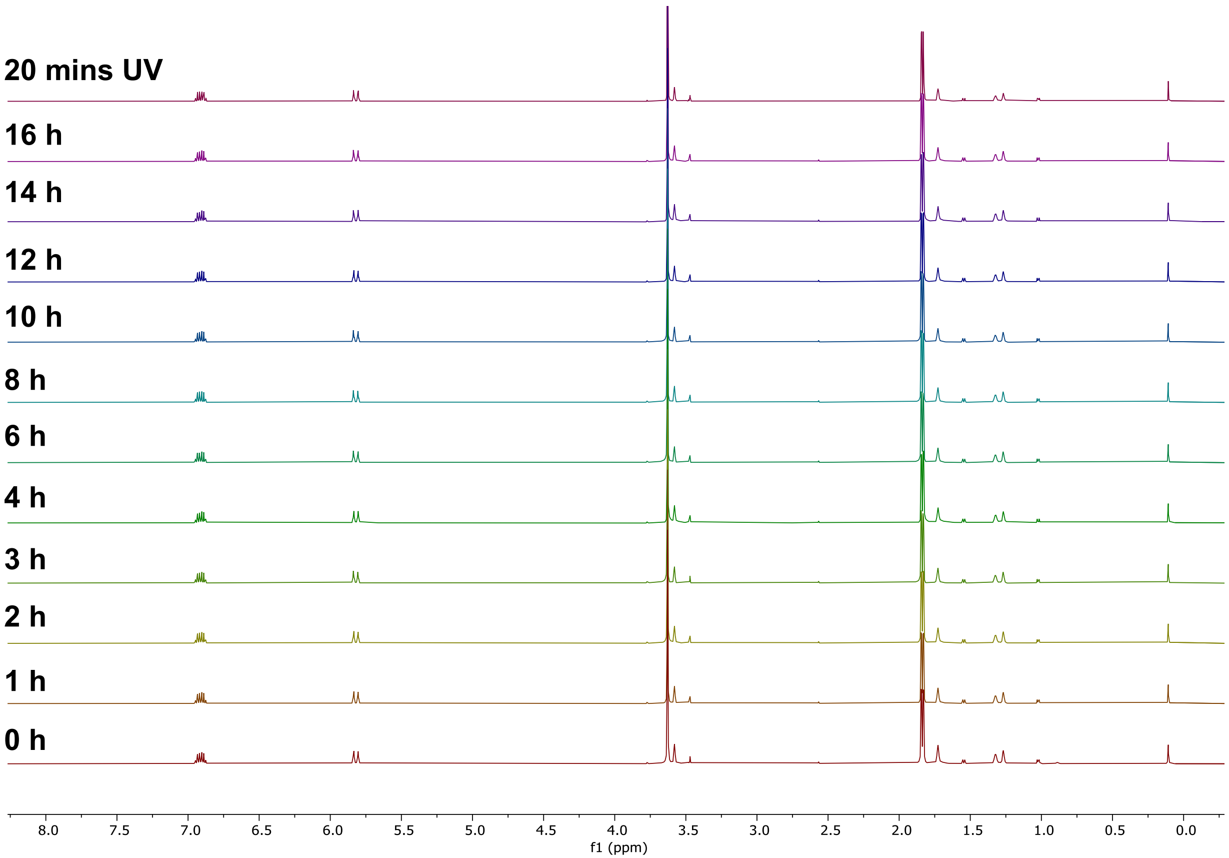

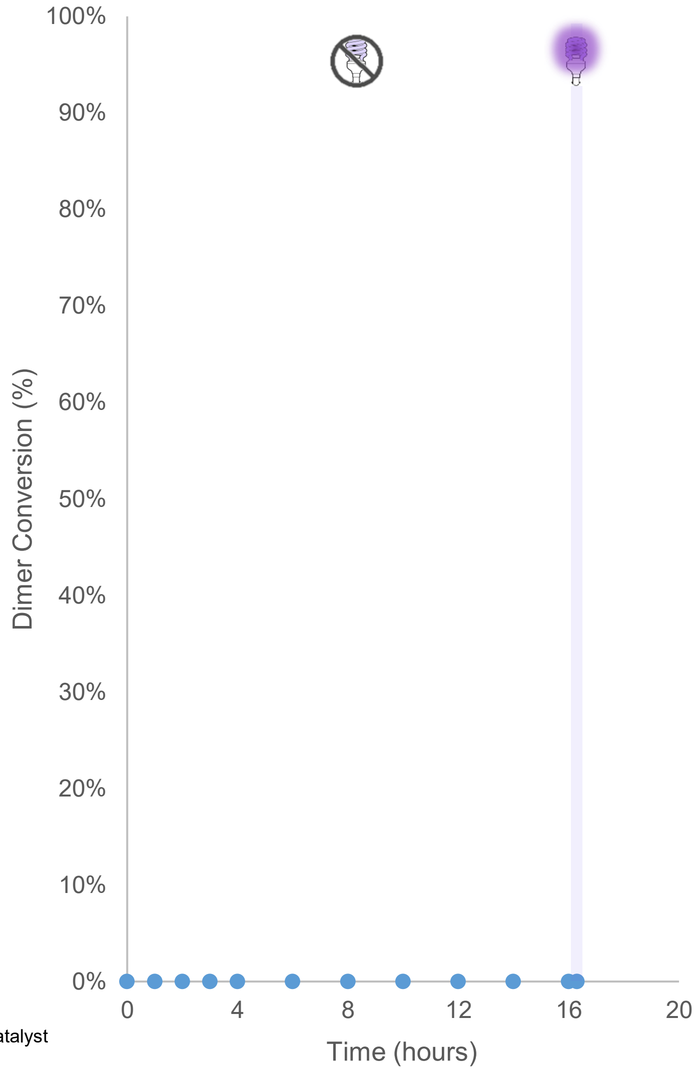

Supplementary Figure 85 – ^{1}H NMR (500 MHz, THF-d_8) stacked spectra (left) showing *trans*-[(dmpe)$_2$FeH($CH_3O_2CC_6H_{10}COO^-$)] **17** (at 5 mol% catalyst loading) is catalytically inactive to methyl crotonate **1** dimerisation under both light and no-light conditions (right).

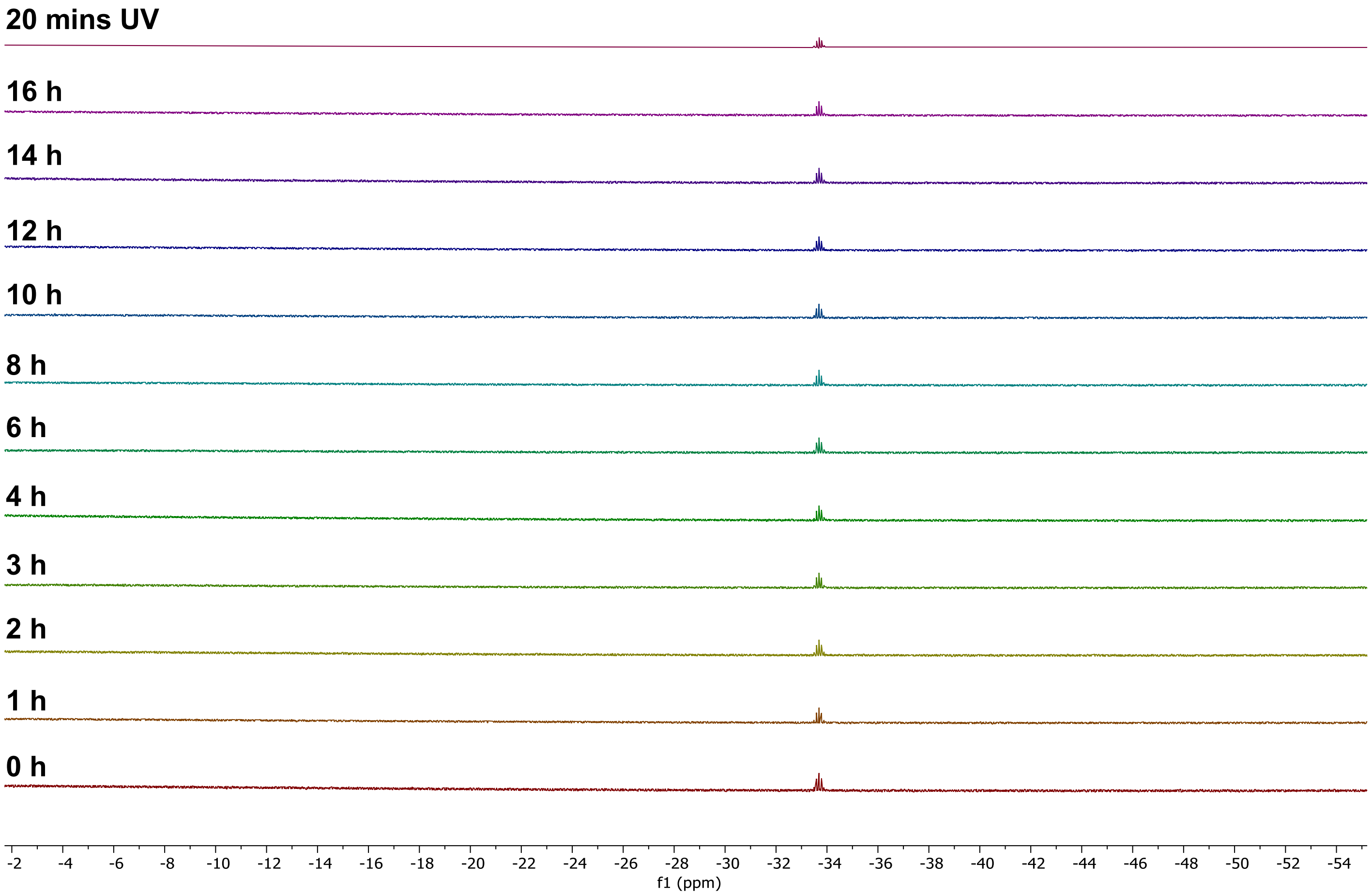

Supplementary Figure 86 – ^{1}H NMR (500 MHz, THF-d_8) stacked spectra of the hydride region showing no speciation in *trans*-[(dmpe)$_2$FeH(CH$_3$O$_2$CC$_6$H$_{10}$COO$^-$)] **17** (at 5 mol% catalyst loading).

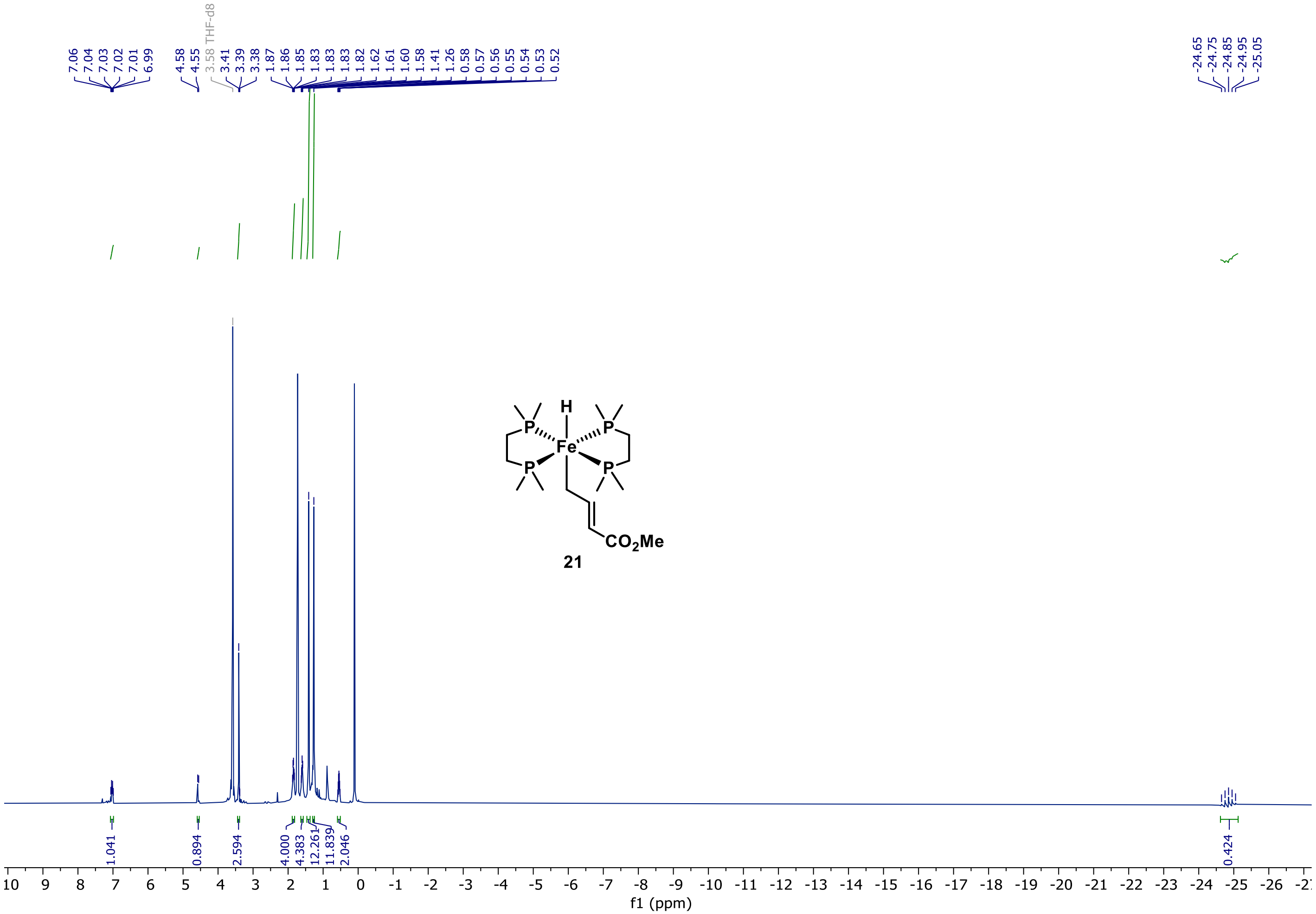

Supplementary Figure 87 – ^{1}H NMR (500 MHz, THF-d_8) of *trans*-[(dmpe)$_2$FeH(CH$_2$CH=CHCO$_2$Me)] **21**.

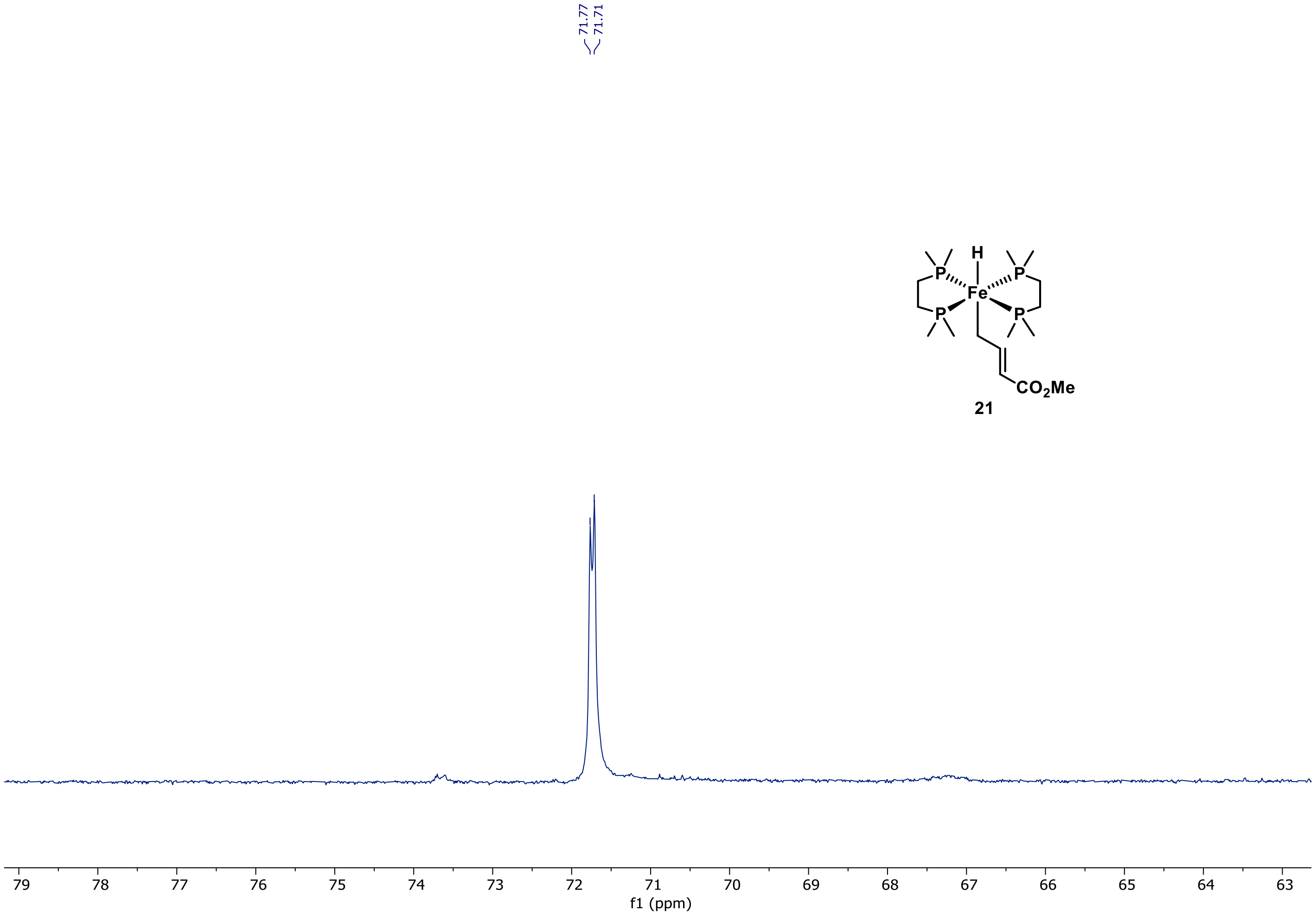

Supplementary Figure 88 – $^{31}P\{^1H\}$ NMR (202 MHz, THF-d_8) of *trans*-[(dmpe)$_2$FeH($CH_2CH=CHCO_2Me$)] **21**.

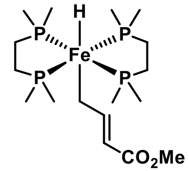

21

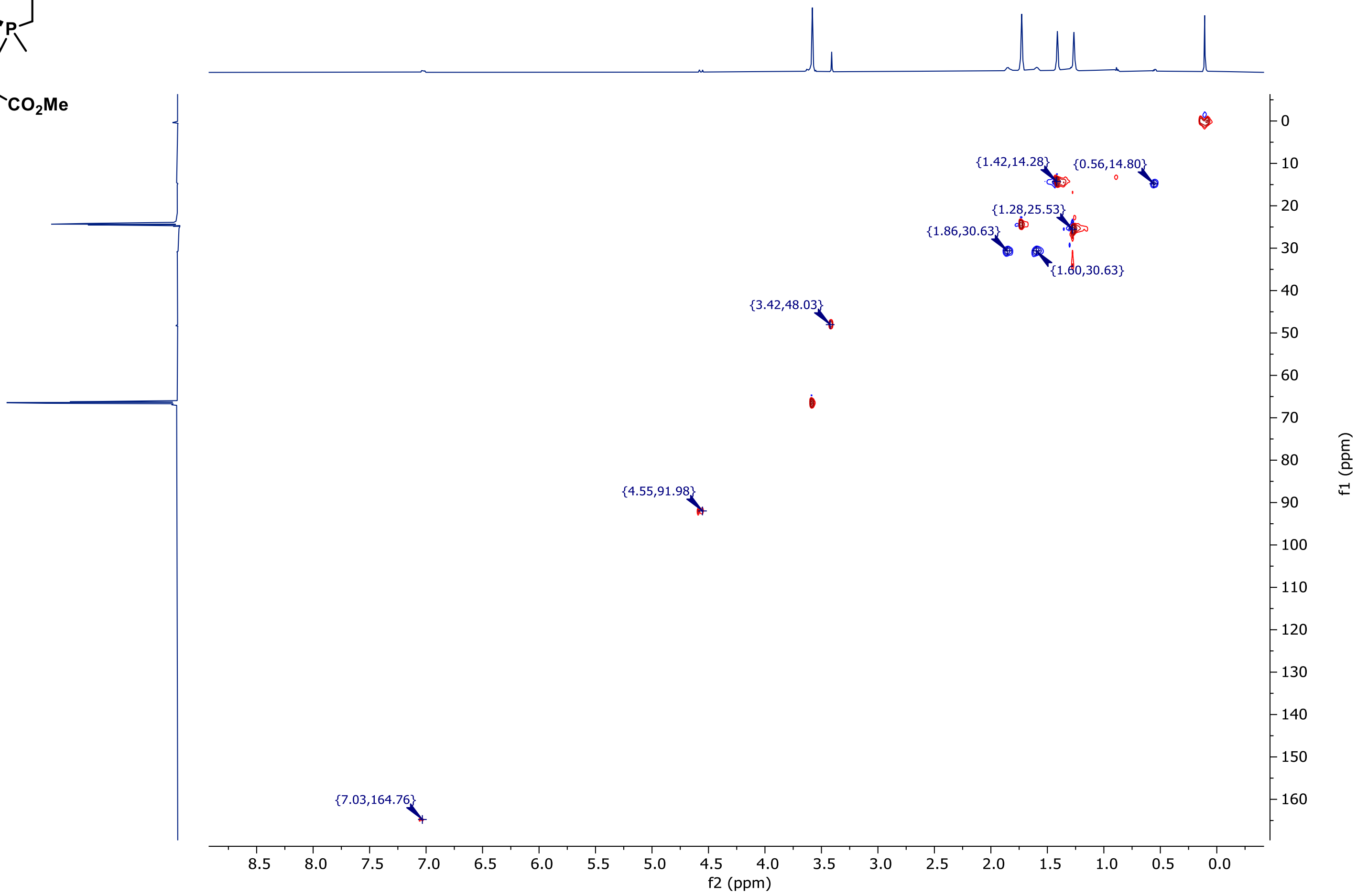

Supplementary Figure 89 –^{1}H–^{13}C HMBC NMR (THF-d_8) of *trans*-[(dmpe)$_2$FeH(CH$_2$CH=CHCO$_2$Me)] **21**.

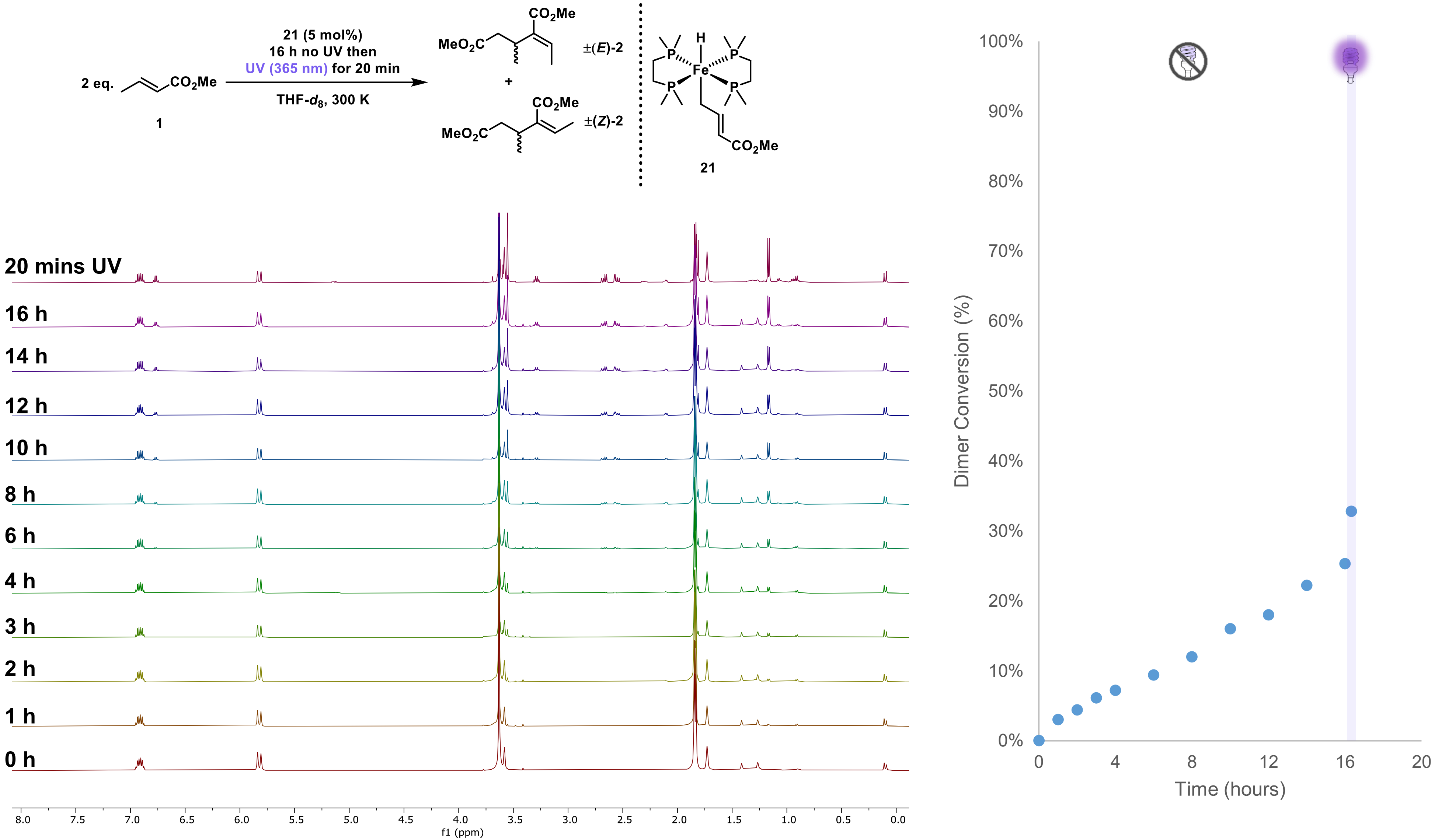

Supplementary Figure 90 – ^{1}H NMR (500 MHz, THF-d_8) stacked spectra (left) showing *trans*-[(dmpe)$_2$FeH($CH_2CH{=}CHCO_2Me$)] **21** (at 5 mol% catalyst loading) is catalytically active and can dimerise methyl crotonate **1** to (*E*),(*Z*)-2-ethylidene-3-methylpentanedioate **2** (right) under no-light conditions for 16 hours but also dimerises methyl crotonate **1** more rapidly under UV irradiation (365 nm) for 20 minutes.

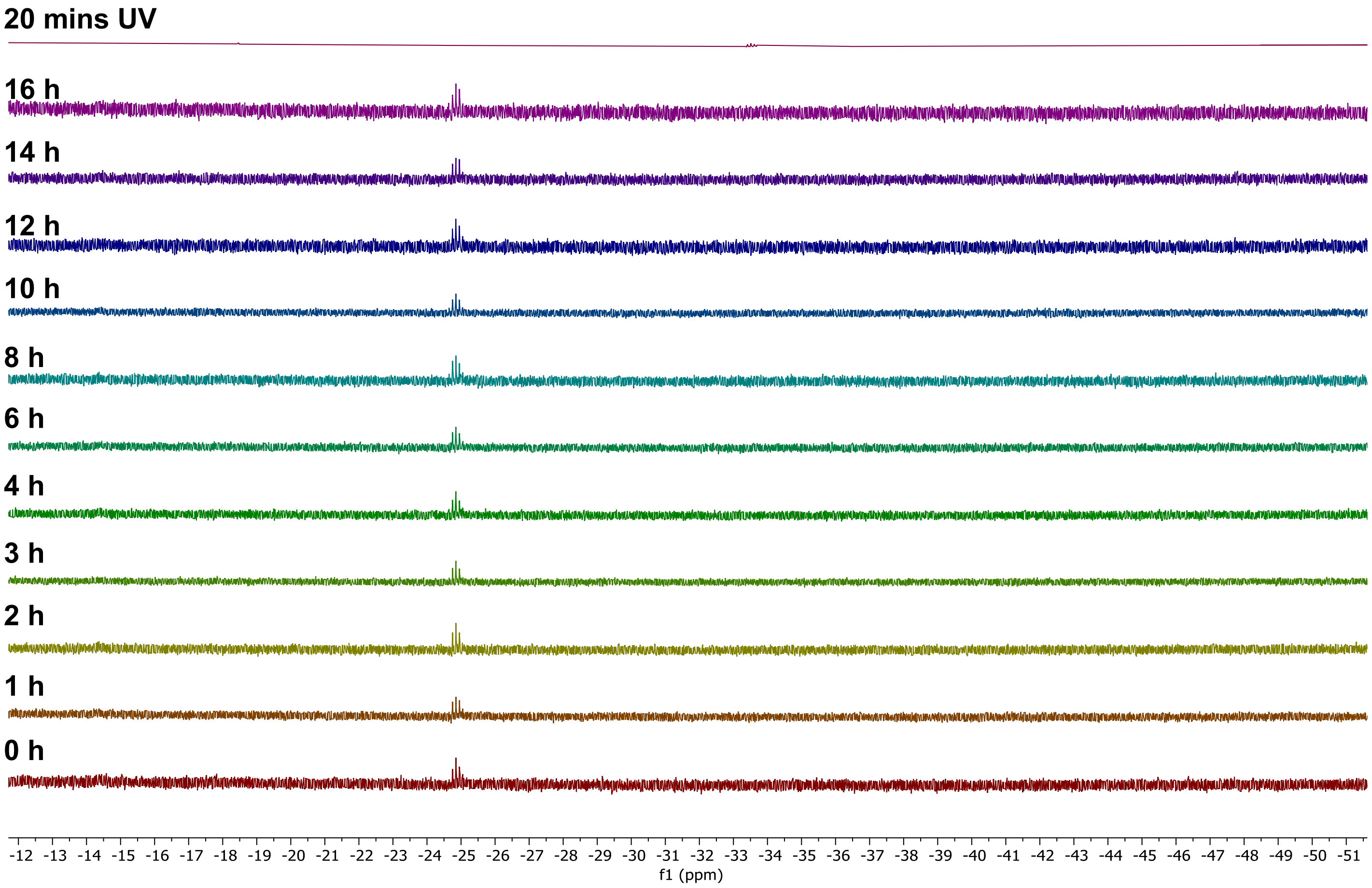

Supplementary Figure 91 – ^{1}H NMR (500 MHz, THF-d_8) stacked spectra of the hydride region showing *trans*-[(dmpe)$_2$FeH(CH$_2$CH=CHCO$_2$Me)] **21** (at 5 mol% catalyst loading), before being completely consumed when the sample was photoirradiated (365 nm) to form *trans*-[(dmpe)$_2$Fe(CH$_3$CH=CHCOO$^-$)] **16** (top spectrum).

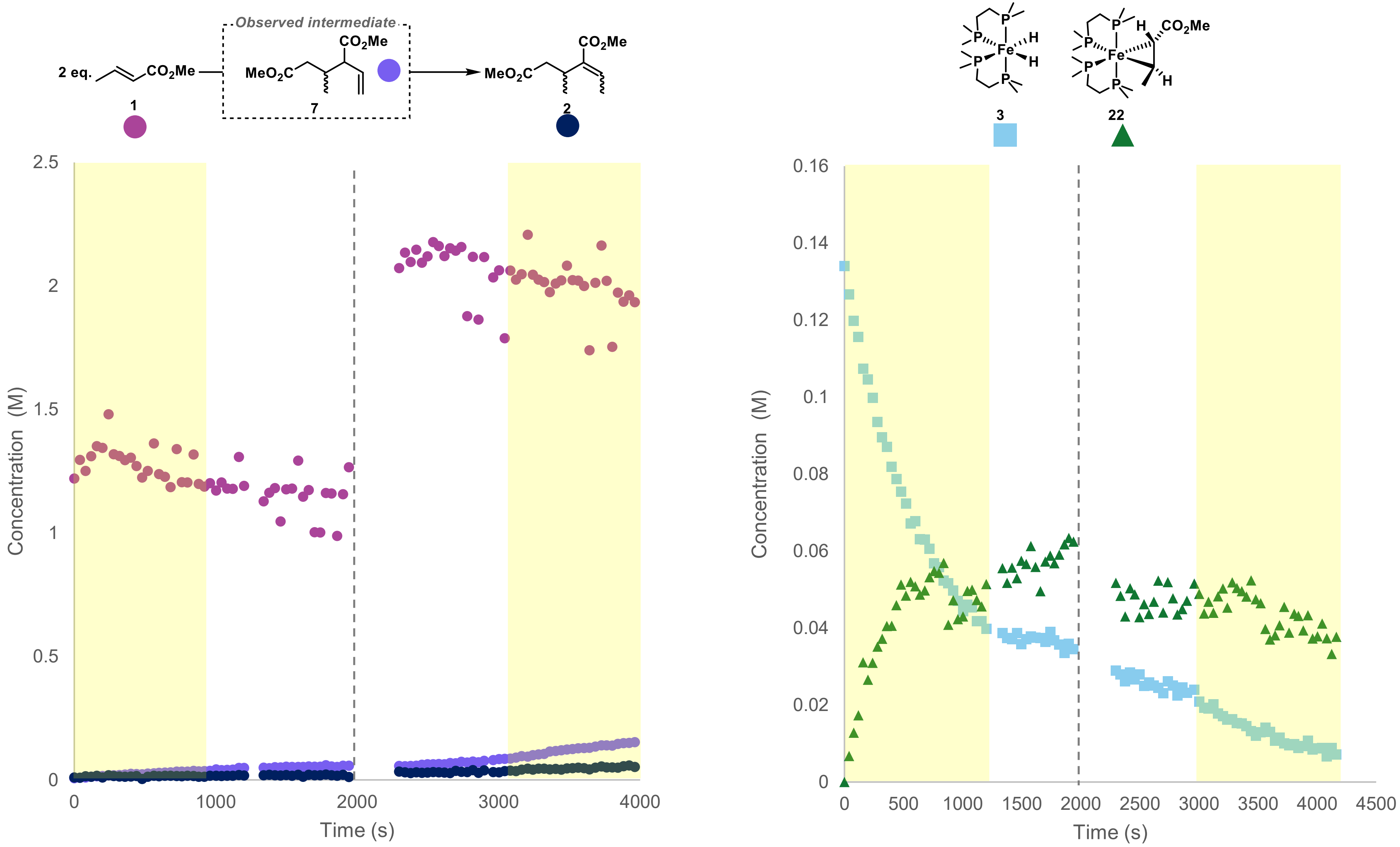

Supplementary Figure 92 – *in situ* Monitoring by ^{1}H NMR (left) and $^{31}P\{^{1}H\}$ NMR spectroscopy (right) showing that $[(dmpe)_2Fe(\eta^2\text{-}\mathbf{1})]$ **22** is catalytically active in the presence of methyl crotonate **1**. Yellow boxes indicate periods of sample irradiation.

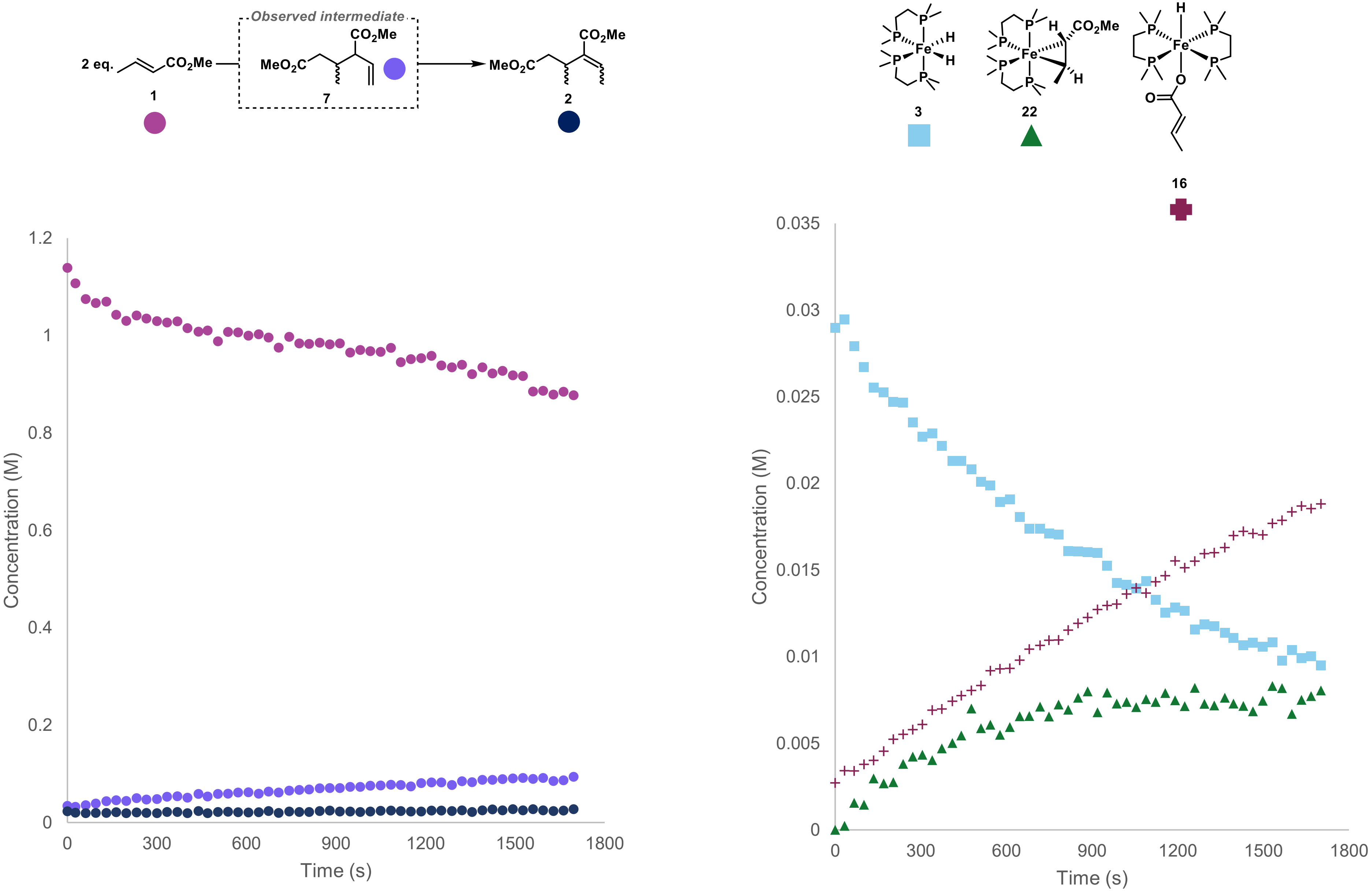

Supplementary Figure 93 – *in situ* Monitoring (see General Procedure in section **S4.5** for more details) by ^{1}H NMR (left) and $^{31}P\{^{1}H\}$ NMR spectroscopy (right) of a thermal control at 309 K of methyl crotonate **1** dimerisation by [(dmpe)$_2$FeH$_2$] **3**.

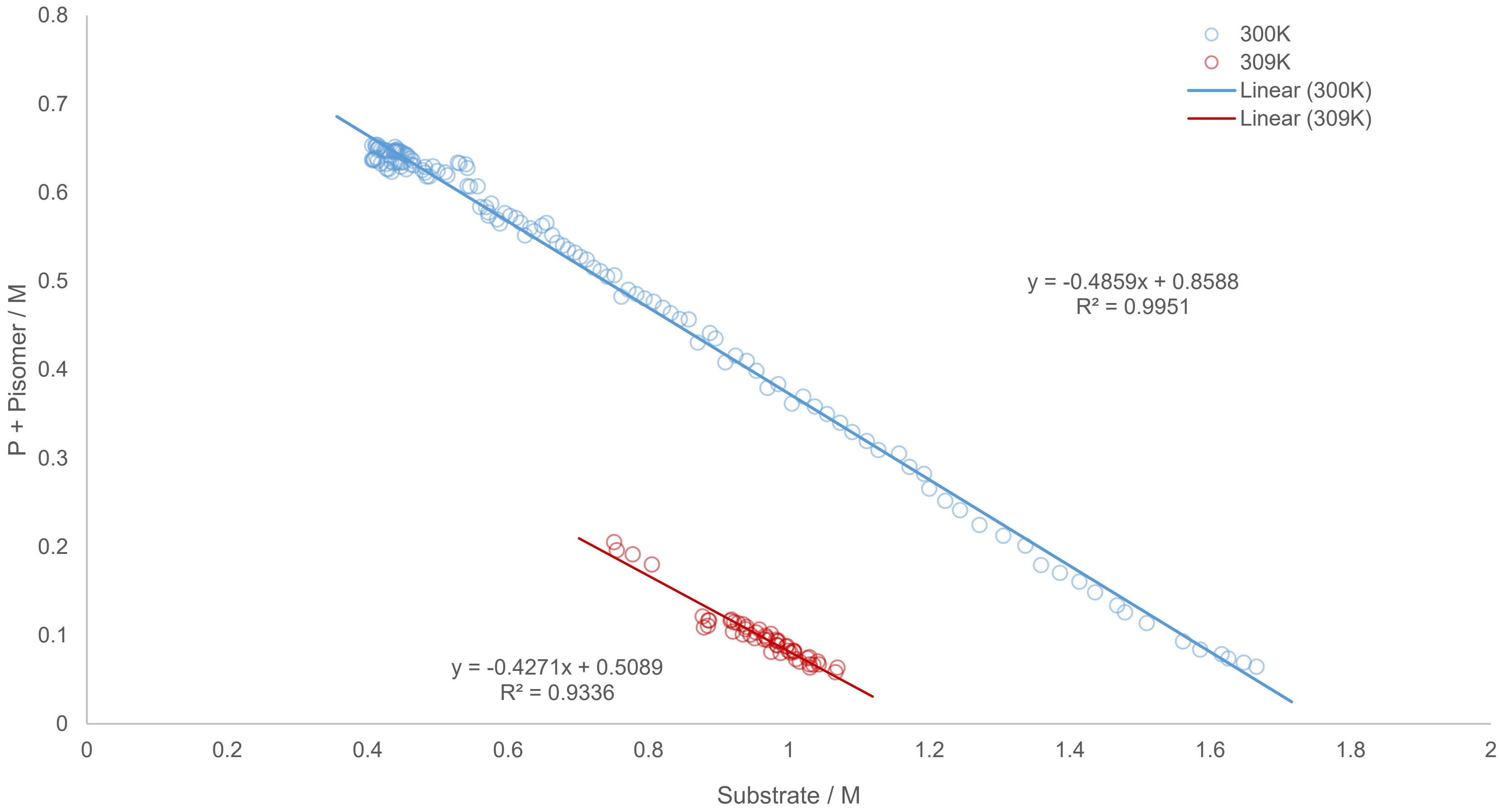

Supplementary Figure 94 – Product concentration versus substrate concentration at 300 K and 309 K showing little change in the rate of dimerisation despite the temperature difference.

S10 References

1. G. R. Fulmer, A. J. M. Miller, N. H. Sherden, H. E. Gottlieb, A. Nudelman, B. M. Stoltz, J. E. Bercaw and K. I. Goldberg, *Organometallics*, 2010, **29**, 2176-2179.
2. A. Flook and G. C. Lloyd-Jones, *J. Org. Chem.*, 2024, **89**, 16586-16593.
3. G. M. Sheldrick, *Acta Crystallogr., Sect. A: Found. Crystallogr.*, 2008, **64**, 112-122.
4. O. V. Dolomanov, L. J. Bourhis, R. J. Gildea, J. A. Howard and H. Puschmann, *J. Appl. Crystallogr.*, 2009, **42**, 339-341.
5. G. M. Sheldrick, *Acta Crystallogr., Sect A: Found. Adv.*, 2015, **71**, 3-8.
6. G. M. Sheldrick, *Acta Crystallogr., Sect. C: Struct. Chem.*, 2015, **71**, 3-8.
7. T. E. Elton, G. E. Ball, M. Bhadbhade, L. D. Field and S. B. Colbran, *Organometallics*, 2018, **37**, 3972-3982.
8. T. Dombray, C. G. Werncke, S. Jiang, M. Grellier, L. Vendier, S. Bontemps, J.-B. Sortais, S. Sabo-Etienne and C. Darcel, *J. Am. Chem. Soc.*, 2015, **137**, 4062-4065.
9. D. E. Seeger, P. M. Lahti, A. R. Rossi and J. A. Berson, *J. Am. Chem. Soc.*, 1986, **108**, 1251-1265.
10. T. M. Werkhoven, R. van Nispen and J. Lugtenburg, *Eur. J. Org. Chem.*, 1999, **1999**, 2909-2914.
11. Rutger B. Boers, Yolanda P. Randulfe, Hendrikus N. S. van d. Haas, Marleen v. Rossum-Baan and J. Lugtenburg, *Eur. J. Org. Chem.*, 2002, **2002**, 2094-2108.
12. M. Kishida, N. Yamauchi, K. Sawada, Y. Ohashi, T. Eguchi and K. Kakinuma, *J. Chem. Soc., Perkin Trans. 1.*, 1997, DOI: 10.1039/A606208C, 891-896.
13. D. G. I. Kingston and H. P. Tannenbaum, *Organic Mass Spectrometry*, 1975, **10**, 263-272.
14. J. C. A. Flanagan, E. J. Kang, N. I. Strong and R. M. Waymouth, *ACS Catal.*, 2015, **5**, 5328-5332.
15. C. Feldmeier, H. Bartling, E. Riedle and R. M. Gschwind, *J. Magn. Reson.*, 2013, **232**, 39-44.
16. M. V. Baker, L. D. Field and D. J. Young, *J. Chem. Soc. Chem. Comm.*, 1988, DOI: 10.1039/C39880000546, 546-548.
17. C. Tolman, S. Ittel, A. English and J. Jesson, *J. Am. Chem. Soc.*, 1978, **100**, 4080-4089.
18. L. R. Doyle, P. J. Hill, G. G. Wildgoose and A. E. Ashley, *Dalton Trans.*, 2016, **45**, 7550-7554.
